# Phylostratigraphic Analysis Shows the Earliest Origination of the Abiotic Stress Associated Genes in *A. thaliana*

**DOI:** 10.3390/genes10120963

**Published:** 2019-11-22

**Authors:** Zakhar S. Mustafin, Vladimir I. Zamyatin, Dmitrii K. Konstantinov, Aleksej V. Doroshkov, Sergey A. Lashin, Dmitry A. Afonnikov

**Affiliations:** 1 The Institute of Cytology and Genetics of the Siberian Branch of the Russian Academy of Sciences (IC & G SB RAS), 630090 Novosibirsk, Russia; mustafinzs@bionet.nsc.ru (Z.S.M.); zamyatin@bionet.nsc.ru (V.I.Z.); konstantinov@bionet.nsc.ru (D.K.K.); ad@bionet.nsc.ru (A.V.D.); 2 Kurchatov Genomics Center, Institute of Cytology and Genetics, SB RAS, 630090 Novosibirsk, Russia; 3 Faculty of Natural Sciences, Novosibirsk State University (NSU), 630090 Novosibirsk, Russia

**Keywords:** abiotic stress, *A. thaliana*, phylostratigraphic analysis, gene network, network structure, gene family evolution, divergence, multifunctional genes

## Abstract

Plants constantly fight with stressful factors as high or low temperature, drought, soil salinity and flooding. Plants have evolved a set of stress response mechanisms, which involve physiological and biochemical changes that result in adaptive or morphological changes. At a molecular level, stress response in plants is performed by genetic networks, which also undergo changes in the process of evolution. The study of the network structure and evolution may highlight mechanisms of plants adaptation to adverse conditions, as well as their response to stresses and help in discovery and functional characterization of the stress-related genes. We performed an analysis of *Arabidopsis thaliana* genes associated with several types of abiotic stresses (heat, cold, water-related, light, osmotic, salt, and oxidative) at the network level using a phylostratigraphic approach. Our results show that a substantial fraction of genes associated with various types of abiotic stress is of ancient origin and evolves under strong purifying selection. The interaction networks of genes associated with stress response have a modular structure with a regulatory component being one of the largest for five of seven stress types. We demonstrated a positive relationship between the number of interactions of gene in the stress gene network and its age. Moreover, genes of the same age tend to be connected in stress gene networks. We also demonstrated that old stress-related genes usually participate in the response for various types of stress and are involved in numerous biological processes unrelated to stress. Our results demonstrate that the stress response genes represent the ancient and one of the fundamental molecular systems in plants.

## 1. Introduction

Being sessile organisms, plants cannot avoid being exposed to stressful conditions. They constantly fight with stressful factors such as high or low temperature, drought, soil salinity, and flooding. Plants have evolved a set of stress tolerance mechanisms, which are different processes involving physiological and biochemical changes that result in adaptive or morphological changes. The study of the mechanisms of plants adaptation to adverse conditions, as well as their response to stresses, is of great interest in the selection of stress resistant varieties [1,2].

The response of plants to stress factors is complex, both in terms of the physiological and molecular systems involved [3,4]. For example, a turgor pressure in plant cells decreases under drought stress, and pH is changed as well as cell size. The pressure drop is captured by receptor kinases and they activate the abscisic acid signaling which regulate a series of effector genes [5]. This leads to a physiological reaction of the plant: stomata closure and osmoprotectant synthesis, which ensures the adaptation of the plant to stressful conditions. As a result of the heat stress action, the activation of a series of regulatory pathways occurs: kinase cascades, sumoylation, protein–protein interactions, Ca^2+^, etc. They eventually activate heat shock transcription factors [6,7], which leads to the expression of chaperones and enzymes that provide acclimation, the primary adaptation to stress. Thus, the response to stress has several stages involving different genetic components. These components are common and include stress sensing, hormonal signal transduction [8], specific transcription factors [7], and protein kinase cascades [9], leading to phenotypic and physiological changes in plant [5,10]. All these multilevel processes can be described using gene networks in which many genes are involved [11,12]. Analysis of the structure of stress gene networks reveals hub genes that may be important for the response to stress [13,14], thus greatly expediting the progress of discovery and functional characterization of the stress-tolerant genes/QTLs [15].

The stress response gene networks also change in the process of evolution; therefore, analysis of the systems of interacting genes, taking into account evolutionary patterns and expression data, allows the identification of features of the organization of response systems to stress factors and their most significant components promising for gene prioritization [16,17,18,19]. One promising approach in this regard is phylostratigraphic analysis. It is of great interest in relation to identification of important stages of genome evolution, where appearance of new genes took place, and to identification of lineage specific genes [20]. This analysis allows the determination of the time of occurrence of genes, assess their age and correlate these ages with the functional role of genes in an organism tissues [21]. On the other hand, a close relationship between the age of genes and the level of their expression in the process of embryogenesis (‘hourglass-like pattern’) was shown for both animals [22] and plants [23]. Interestingly, an hourglass-like pattern of gene expression by age was also identified in the response of tobacco plants to biotic stress [24]. One of the interesting tasks is to find functional features of genes that differ in age. In particular, several data suggest that genes associated with fundamental processes in cells usually are older than other genes. For instance, the study [20] reported that human genes referring to such phylostrata as Cellular organisms and Eukaryota are generally associated with basal cellular functions (metabolic processes, transcription regulation), while the genes originating in the later stages of evolution are associated with the genes of the immune response and reproduction.

Phylostratigraphic analysis is a promising method for analyzing the evolution of gene networks. In addition to the structural features of networks, it allows one to identify important details of their evolution, in particular to locate functional modules in networks [25]. This approach was used for large-scale analysis of the evolution of *A. thaliana* gene co-expression networks [26]. The authors showed that genes originating in the same evolutionary period tend to be connected, but extremely old and young genes tend to be disconnected.

As it was previously shown, the molecular mechanisms of response to stresses of different types are various and complex, both in the composition of genes involved in response to stress, and in the molecular mechanisms of this response. With this in mind, a more detailed study of phylostratigraphic indices for genes of stress response in plants and their relationship to the structure and functional role of gene networks is of great interest.

In this work, we used the Orthoscape application [27] to carry out phylostratigraphic analysis of genes of plant stress, including the assessment of the distributions of these genes according to their evolutionary age as well as the reconstruction and structural analysis of gene networks by the example of the network of the heat stress response. Our results show that the substantial fraction of genes associated with various types of abiotic stress is of ancient origin and evolves under strong purifying selection. The interaction networks of genes associated with stress response have modular structure with regulatory component being one of the largest for five of seven stress types. We demonstrated a positive relationship between the number of interactions of gene in the stress gene network and its age. Moreover, genes of the same age tend to be connected in gene networks. We also demonstrated that old stress-related genes usually participate in the response for various types of stress and involved in numerous biological processes unrelated to stress. Our results demonstrate that stress response genes represent one of the most ancient and fundamental molecular systems in plants.

## 2. Materials and Methods

### 2.1. Gene Sets Preparation

We analyzed sets of genes associated with seven types of abiotic stress for *Arabidopsis thaliana*: heat, cold, light, osmotic, salt, oxidative stress and water-related stress. Gene sets for each type of stress were formed on the basis of Gene Ontology (GO) terms [28], represented in the TAIR v. 10 [29] annotation. When selecting an annotation, only the terms of the following confidence levels were used: inferred from direct assay (IDA), inferred from mutant phenotype (IMP), inferred from genetic interaction (IGI), inferred from physical interaction (IPI).

In the first step, extended lists of GO terms associated with each type of stress were formed. To do this, we selected all the terms that contained the keyword “stress” in either title or description, as well as all their child terms. After the formation of the initial list, its refinement was carried out, the terms GO not associated with this type of stress were removed. Subsequent analysis showed that the lists of terms associated with the keyword ‘water’ and ‘drought’ were substantially overlapped: 25 terms were associated with the keyword ‘water’ and 10 with ‘drought’, 6 terms were common. Therefore, these two lists in our analysis were combined under the name “water stress”.

The similarity of gene lists was analyzed using the relation tree reconstructed with UPGMA method based on distances calculated using the Ochiai coefficients [30].

To avoid bias due to multiple occurrence of some genes in several gene lists we have used an additional non-redundant stress-related gene list, which includes the genes for all types of stress but in a single occurrence (‘all stresses nr ‘ dataset).

### 2.2. Network Reconstruction for Gene Sets

To reconstruct gene networks for the set of genes, interactions with a level of confidence above 0.7 were searched using the STRING database [31]. It should be noted that the search in the STRING database can change the composition of genes in the reconstructed gene network both by excluding genes from the input list for which no interactions were detected, and by adding new genes (in this paper, we made it possible to add no more than ten additional genes to the existing list). Generated STRING tables were then loaded into Cytoscape [32] for visual network reconstruction and analysis via Orthoscape application [27].

### 2.3. PAI/DI Calculation and Network Visualization

**Figure 1:**
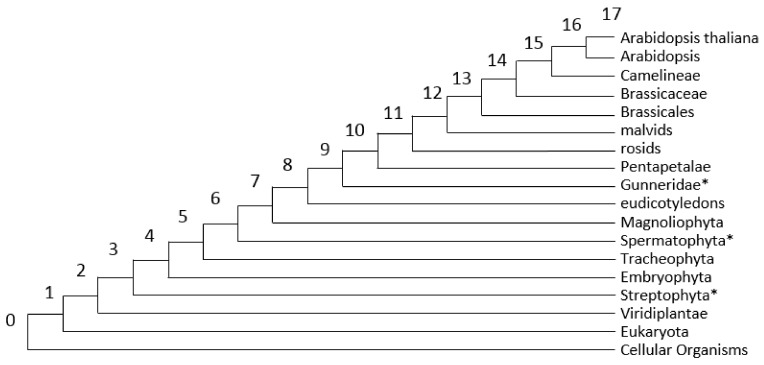
The phylostratigraphic map of *A. thaliana* and phylogeny used in the search for the evolutionary origin of *A. thaliana* genes, 18 genomic phylostrata that correspond to the phylogenetic internodes. An asterisk (*) indicates three phylostrata that were excluded from the statistical analysis.

We used the Orthoscape application [27] for analysis for gene sets of plant stress response and visualization of their reconstructed networks. Orthoscape loads lists of genes and their network relationships either from KEGG database or user-defined file. For each gene in the network, the Orthoscape calculates two evolutionary indices. First, the phylostratigraphic age index (PAI), order number of a phylostratum, indicating the evolutionary age of a gene based on the finding of the most basal taxon, common for the gene and every of its orthologs [21]. The lower PAI is, the lower the phylostratum number is, and the earlier the gene appeared in the course of the organismal evolution [21]. The Orthoscape uses the KEGG Organisms database [33] to get taxonomic trees. It performs a search of orthologous genes, populates the tree of species these genes belong to and then analyses the resulting tree [27]. In the KEGG database, the taxonomic tree for *A. thaliana* contains 18 taxa; they and their corresponding PAI values are shown in Figure 1. However, it was found that some of the taxonomic groups (Streptophyta, PAI = 3; Spermatophyta, PAI = 6; Gunneridae, PAI = 9) have only a single daughter taxon. For example, for Streptophyta it is Embryophyta, for Spermatophyta it is Magnoliophyta, for Gunneridae it is Pentapetalae. Therefore, these taxa were excluded from further analysis. However, we retained the PAI values for remained taxa unchanged. For example, we did not decrease the PAI for Embryophyta and younger taxa by 1 after removing Streptophyta with the PAI = 3, etc. An alternative is also possible: reducing the PAI values of all daughter taxa after excluding Streptophyta, Spermatophyta, and Gunneridae. In this case, for example, the PAI value for *A. thaliana* would be 17 − 3 = 14. Such a shift in PAI values can affect the evaluation of the significance of parametric distribution comparison tests (mean, variance, Pearson’s correlation coefficients, etc.). However, in our study, despite the fact that we provide estimates of the mean values for samples of stress genes, the main conclusions about the differences in the age of stress genes are made on the basis of nonparametric tests (see Section 2.4). In this case, the results will not depend on specific PAI values for taxa.

The *A. thaliana* gene list included 27,636 genes, and below it is assumed as the background *A. thaliana* genes list. 

Second evolutionary index is the divergence index (DI) of a gene indicating the influence of natural selection on gene evolution [23]. It is based on the estimation of the Ka/Ks ratio between the gene from the analyzed organism and the most similar ortholog from relative organism [34]. In this work, we use the closest *A. thaliana* relative, *Arabidopsis lyrata*. The DI value above 1 indicates the evolution of the gene under positive Darwinian selection. The DI close to 1 indicates that a gene evolves under neutral regime. The values of the DI close to 0 indicate strong purifying selection acting on a gene. The DI measure is often used to estimate the level of the selective pressure on the transcriptomes in phylostratigraphic transcriptome analysis [24,35,36,37,38].

The Orthoscape reports the following results: a graphical representation of a gene network graph in which each node of the network corresponding to a gene is colored according to PAI or DI; the PAI values for genes in the gene network, and the result of Ka/Ks ratio evaluation for genes. Orthoscape also provides its output in HTML format along with the generated R scripts that can be used for drawing violin plot for all the distributions obtained. HTML reports contain also the data of specific PAI calculated using weights according the node connectivity.

### 2.4. Significance of the Differences between PAI/DI Distributions in A. thaliana and Stress Gene Sets

To assess the age and the mode of evolution of the abiotic stress genes, we compared the distribution of the PAI and DI values for them with the distribution obtained for background list of protein-coding genes of *A. thaliana*. Differences in distributions were characterized by several parameters. First, we compared the mean PAI and DI values for all *A. thaliana* genes and stress genes. Secondly, we evaluated Chi-square statistics (ChiSqPAI/ChiSqDI) when comparing PAI/DI distributions of all *A. thaliana* genes and each of the stress gene sets. Third, for each PAI value, we evaluated the difference in proportion of genes corresponding to these values in the sets of stress genes and the complete set of genes of *A. thaliana*: dfPAI*_i_* = fPAI*_i_*_stress_ − fPAI*_i_*_At_, where fPAI*_i_*_stress_ is the proportion of genes with PAI*_i_* in the samples of stress genes, and fPAI*_i_*_At_ is the proportion of such genes in the sample of all genes. The parameters of dfDI*_i_*, differences in the frequency of genes in a certain range of values (bin) in the sets of stress genes and the complete set of *A. thaliana* genes, were calculated in the same way. As a null hypothesis, it was assumed that the estimated distributions of the sets of stress genes are identical to those in the sample of all *A. thaliana* genes. 

To assess the statistical significance of the differences between the obtained characteristics of stress gene distributions and the whole set of genes, we performed a shuffling test. For each type of stress, from the pool of all protein-coding genes, we randomly selected without replacement as many genes as were represented in the corresponding set. After that, PAI/DI distributions for this random set of genes were constructed and compared with the distribution for the complete set of genes according to the parameters described above (mean value, Chi-square statistics and the difference in frequencies in bins). Random sets of genes for each type of stress were generated 10^5^ times. The characteristics obtained for random samples were compared with those corresponding to the initial stress sample. For each characteristic, the number of n_rand_ samples was calculated, such that (1) the average value of PAI < PAI_rand_ and; (2) ChiSqPAI < ChiSqPAI_rand_; (3) for each PAI value dfPAI*_i_* < dfPAI*_i_*_rand_. The significance of deviations of the corresponding characteristics from those for the null hypothesis was estimated as *p* = n_rand_/10^5^. For example, if we obtained n_rand_ = 500 (*p* = 0.005) for the average PAI score, it would mean that the average age of the stress genes is significantly greater than the age for all *A. thaliana* genes (corresponding to lower PAI values). If a similar estimate is obtained for the ChiSq parameter, it means that the distribution of gene frequencies of different ages in the stress gene set is significantly different from that for a set of randomly selected genes. Hence, if similar estimates of *p* are obtained for some value *i* of the PAI, it means that genes with the age *i* are significantly more frequent in the sample of stress genes than can be expected for random choice for a set of randomly selected genes of *A. thaliana*.

A similar statistical test was applied to DI distributions in stress gene sets.

### 2.5. Gene Ontology Annotation Enrichment for Stress Gene Sets

The enrichment analysis of stress gene sets with the GO terms for dictionaries ‘Biological process’, ‘Cellular component’ and ‘Molecular function’ was carried out with the help of DAVID service v6.8 [39]. The significance of association of the term GO with any set of genes was determined at *p*-value < 0.05 with Benjamini correction for multiple comparison [40]. 

### 2.6. Relationship between Gene Network Structural and Evolutionary Characteristics

We distinguished clusters in the structure of gene networks on the basis of visual analysis. We evaluated the quality of the network graph partitioning into clusters using the internal degree of the node (deg_int_), the average value of the edges connecting nodes within cluster and external degree for the node (deg_ext_), the average value of the edges connecting nodes from the cluster to other nodes in the network [41]. The higher deg_int_ and the lower deg_ext_ are, the better is node selection for the cluster. For each cluster, we have separately identified the significantly associated GO terms as described in Section 2.5. 

To assess the relationship between the structure of gene networks of stress response and the evolutionary characteristics of genes, we used the following graphs characteristics for each networks: the degree of node, i.e., the number of other connected nodes, *k* [42]; list of pairs of interacting nodes; lists of pairs of nodes, the shortest path between which has two edges or more. To calculate the shortest path between nodes, we used the NetworkX [43] library’s method all_pairs_shortest_path() without limiting cutoff on the maximum length of the shortest path.

To assess the relationship between the structural characteristics of nodes and their evolutionary characteristics, we calculated: (1) the Pearson correlation coefficient between the degree of node *i*, *k_i_*, and its age PAI*_i_*; (2) the scalar assortativity coefficient [44] for PAI values in pairs of interacting genes. Assortativity coefficients, *r_a_*, were estimated using graph-tool package [https://graph-tool.skewed.de/], graph_tool.correlations function. The graph_tool.correlations function additionally returns the variance of the assortativity coefficient estimated by jack-knife test [44]. This allowed us to estimate the standard deviation of the coefficient, *σ*(*r_a_*) and to evaluate the significance of the *r_a_* deviation from 0. We also calculated the distributions of the absolute difference between PAI values, |dPAI| for gene pairs separated in the network structure by 1, 2 and 3 and more edges. 

### 2.7. Evolutionary Changes of Gene Functions in Stress-Related Gene Networks

The difference in the ages of genes within the network implies a change in the composition of genes in the process of its evolution. Together with the change in the composition of genes, the set of functions of genes (GO terms) involved in the network changes respectively. To track changes in the composition of GO terms during the evolution of the gene network, we conducted the following analysis. For each term associated with a stress gene network, we defined a set of genes whose annotations contained this term. For a set of these genes, we built a distribution of frequencies of occurrence by age. For example, if all genes in the gene network of stress annotated with the GO term ‘Seed development’, the most ancient was consistent with the phylostratum Magnoliophyta, which meant that at the stage of evolution corresponding to the emergence of flowering plants, some genes along with the response to stress became involved in the development process of seeds. Such distributions were constructed for all GO terms significantly associated with sets of genes of different types of stress (see Section 2.5).

## 3. Results

### 3.1. GO Terms and Genes Associated with Abiotic Stress

Using GO terms processing (see Section 2.1), we selected 161 terms that characterize particular types of stress. The list of GO terms associated with stress and the list of *A. thaliana* genes, annotations of which contain these terms, are presented in Supplementary file 1. It turned out that the number of GO terms that characterize stress varies among different types of stress. Among the GO terms we found 48 terms associated with light stress, and only 4 terms associated with cold stress. For other types of stress, the number of terms associated with them varied from 14 to 28.

Despite the strong difference in the number of terms associated with different types of stress, the number of genes in the TAIR database that were identified based on annotations for different types of stress differed by no more than 2.5 times (Table 1). The minimum number of genes (102) was associated with heat stress, the maximum (231) with salt stress. There was no significant correlation between the number of GO terms and the number of genes associated with these terms (Pearson correlation coefficient between these values was found to be 0.09).

To identify orthologs via the Orthoscape and to perform the subsequent analysis, we used data from the KEGG database [https://www.genome.jp/kegg/]. It was found that not all sequences of *A. thaliana* genes from the TAIR database, which served as the initial source of stress gene lists, are represented in KEGG. However, the differences in the number of genes for different types of stress were no more than 14 (the difference for light stress, 10% of the total list of genes for this stress, see Table 1). For other types of stress, the differences ranged from 0 to 6 genes.

There are genes common to different gene sets. For example, 13 genes of salt stress (5.6% of the total number, 231) included in the heat stress dataset (TAIR annotation). The number of genes in common between pairs of stress datasets provided in Table 2, along with the number of unique genes for each stress. It is apparent from the table that the gene set for osmotic stress shares largest fraction of genes with other datasets (40% with salt, 25% with water-related, 15% with cold and 10% with oxidative stresses). On the other hand, a large fraction of gene sets has several genes common with the salt stress dataset (5 out of 6 types have more than 10% of genes in common with this type of stress). However, the majority of comparisons yield less than 10% of common genes (28 out of 42). The fraction of unique genes for the datasets was lower than 50% for only one type of stress, osmotic (30%); for three datasets, it was greater than 70%, and for another three datasets it was greater than 50% (Table 2). The ratio of the number of common genes among all eight sets of genes is shown in the form of a tree, which was built with the UPGMA method using distances calculated on the basis of the Ochiai coefficients (Figure S1, Supplementary file 2). The tree reflects the above results properly: the proximity of gene sets of osmotic and salt, heat and light stress is well shown. These results demonstrate the polyfunctionality of stress response genes: some genes participate in the response to more than one stress.

The lists of genes from each specific stress type dataset are provided in Supplementary file 3. In our work, we will analyze the seven types of gene sets separately.

### 3.2. Analysis of PAI Distribution 

**Figure 2:**
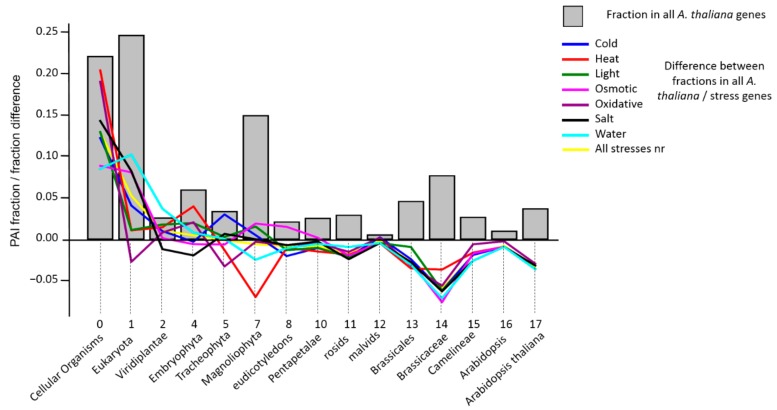
The distribution of frequencies of *A. thaliana* protein-coding genes (y-axis) by PAI (X-axis) is shown as grey bars. Solid lines indicate the values of the difference between the frequencies of occurrence of PAI values in stress dataset and all *A. thaliana* genes (dfPAI*_i_*). Correspondence of the line color and stress type is shown in the box in the upper right corner.

We calculated PAI indices for the background list of protein-coding genes in the *A. thaliana* genome and built their distribution (Figure 2, grey bars; see also Supplementary file 4 for the full data for *A. thaliana* genes). The Identity parameter for the identification of homologues in the Orthoscape program was set to 0.5. The PAI distribution for all *A. thaliana* genes is multimodal and has three distinguished peaks at Cellular Organisms-Eukaryota, Magnolyophyta and Brassicacea phylostrata. The first peak is the largest; almost 50% of genes in A. thaliana genome have PAI 0 or 1. The second peak comprises 15% of genes and the third one covers more than 6%. 

Similar PAI distributions were calculated for gene samples of each type of stress. Next, we calculated the difference between frequencies of occurrence of genes at each PAI values in the stress dataset and all *A. thaliana* genes (dfPAI). Figure 2 shows this difference as colored lines for each type of stress.

This diagram clearly demonstrates that PAI for genes from the analyzed stress datasets have a higher fraction of genes with lower PAI values in comparison to the distribution of the background genes. For instance, a large excess of genes from stress datasets is observed for Cellular organisms (all difference values in stress datasets are positive). For Eukaryota phylostratum, the values are positive for all types of stress except oxidative. For large PAI values (>12, malvids) all of the difference values are below zero. The combined list of genes ‘All stresses nr’ demonstrates a pattern of differences between background dataset similar to specific ones: positive values for earliest phylostrata and negative for the latest. It should be noted that similar patterns (the prevalence of positive values of the difference in frequencies in the samples of stress genes and all genes for small PAI values and negative for large PAI values) are typical for PAI estimates obtained with the values of Identity parameter of 0.7 and 0.6 (Figure S2, Supplementary file 2).

To assess the significance of deviations between PAI distributions in stress gene sets and in all *A. thaliana* genes, we performed a Monte Carlo randomization test (see Methods, Section 2.4). Its results are presented in Table 3 (PAI estimates at Identity = 0.5). The table shows that in all random samples of the same size as the stress gene samples, the average PAI value exceeded the values for stress genes, i.e., the average PAI values for stress genes are significantly (*p* < 10^−5^) less than would be expected for random samples from the whole set of *A. thaliana* genes. Table 3 also shows the Chi-squared values that characterize the degree of difference between PAI distributions in stress gene sets and PAI distributions of all *A. thaliana* genes. For stress genes, these values are higher than for random samples (the value of ChiSq_stress_ < ChiSq_rand_ is less than 5000 samples out of 10^5^, i.e., *p* < 0.05). This suggests that PAI distributions in stress genes are significantly different from those in random gene sets.

The randomization test also has shown that the number of genes represented in Cellular Organisms phylostratum in the sets of stress genes is significantly higher than expected by chance for randomized samples (Table 3). For heat, salt and oxidative stress, this number was exceeded in none of the random samples, and in the rest, the number of random samples with excess of the proportion of genes of this phylostratum ranged from 10 to 1051 (*p* < 0.05). A similar situation was observed in Eukaryota phylostratum for osmotic, salt and water stress genes, in Viridiplantae phylostratum for water stress, in Embryophyta for heat and in Tracheophyta for cold stress. All these facts are in good agreement with the data shown in Figure 2 and argues for the genes, the origins of which are associated with ancient phylostrata, being more often represented in sets of stress response than the average for the genome. 

Table 3 also shows that the genes of the young phylostrata are underrepresented in the lists of genes of stress response. In the vast majority of random samples, the proportion of such genes is higher for *A. thaliana* phylostratum (with *p* < 0.05 for all types of stress). For Brassicaceae phylostratum, it is observed in all types of stress except heat, for Camelineae phylostratum it is observed in light, water and salt stresses, for rosids it is salt stress, and for Brassicales phylostratum, it is oxidative, salt and water stresses.

Again, the combined list ‘All stresses nr’ demonstrates a similar pattern of *p*-values based on the randomization test: significant low values are observed for Cellular Organisms and for Eukaryota phylostrata, while high values are observed for phylostrata with higher PAI.

It should be noted that the results described above were obtained in determining orthologs with the threshold for the identity of sequences of 50%. We performed a similar analysis by establishing the identity thresholds for comparing sequences of 60% and 70%. The results are presented in Tables S1 and S2 (Supplementary file 2) and show a good agreement with the above: genes, whose origin is connected with ancient phylostrata, are overrepresented in stress samples of genes than in random samples, and the genes that occurred recently, on the contrary, are underrepresented in stress ones.

These data show that the frequency distribution representation of genes with different PAI values in genes associated with different types of stress and all genes are significantly different; additionally, stress response genes are characterized by elder age than for all *A. thaliana* proteome on average.

### 3.3. Analysis of DI Distribution

**Figure 3:**
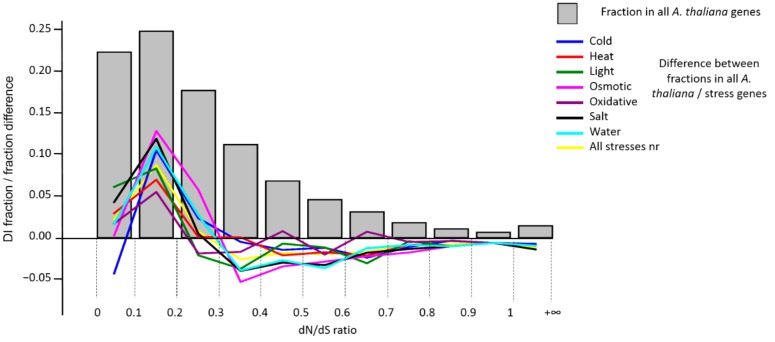
The distribution of frequencies of *A. thaliana* protein-coding genes (y-axis) by the DI value (x-axis) is shown by grey columns. Solid lines show the difference between the frequencies of occurrence of DI values in the stress dataset and all *A. thaliana* genes. Correspondence between the line color and the stress type is shown in the box in the upper right corner.

A similar analysis was conducted in order to compare the index of sequence divergence, the DI, for the stress genes and for all genes of *A. thaliana* (as described in Section 2.3). Those genes of *A. thaliana* that had no synonymous substitutions (Ks = 0, 1147 genes) were excluded from the analysis. 2679 genes had a zero value of the parameter DI (Ka = 0, Ks ≠ 0), which was approximately 10% of the total number of genes. 391 genes (1.4%) had a value of DI > 1, indicating evolution under positive Darwinian selection. Interestingly, we found only two genes with DI > 1 among the stress genes. These genes were RCI2A/AT3G05880 (low temperature and salt responsive protein family, associated with the cold stress, DI = 1.8019) and DREB2B/AT3G11020 (DRE/CRT-binding protein 2B, DI = 1.0176). This is a relative fraction of at least 2 times less than for all *A. thaliana* genes. The histogram of the distribution of all genes by the DI value and the difference in frequencies in the bins between all *A. thaliana* genes and the stress genes is shown in Figure 3.

The figure shows that the genes with lower values of DI (less than 0.3) in the sets of stress genes are overrepresented, and those with more are underrepresented. However, the peak of differences corresponds to the DI (0.1, 0.2] bin, not the [0.0, 0.1] bin, which is associated with a reduced proportion of genes with DI = 0 among stress genes. Thus, although stress genes are under strong purifying selection, their sequences still contain a small proportion of non-synonymous substitutions. To determine the significance of the differences in the distributions of DI stress genes and the complete set of *A. thaliana* genes, we carried out a randomization test similar to the test to check the significance of deviations of the PAI. The results are presented in Table 4. The table shows that the differences observed in Figure 3 in the DI distributions are significant.

In particular, the mean DI values in random gene samples exceeded those for stress gene sets in more than 95% of cases. Significant (*p* < 0.05) deviations in distributions estimated on the basis of Chi-square statistics are observed for the gene sets of light, osmotic, salt and water stress types. Excess of the proportion of genes with DI within the range 0–0.1 compared with the proportion for all *A. thaliana* genes was observed for all types of stress, except cold and salt, but was not significant. A significant excess was observed for all types of stress in the DI range of (0.1–0.2). Table 4 shows that a significant excess of the proportion of genes with values DI > 0.3 in random samples of genes compared to the stress-related is a frequent phenomenon (numerical values in the table are underlined), although not systematic.

We compared quantile values for DI distributions for stress gene sets and for all *A. thaliana* gene set. The results are shown in the Supplementary file, Table S3. The table demonstrates that for quantile 50 (median) and above (q75, q90, q100), all values for stress gene distributions are lower than those for all *A. thaliana* gene set. Quantile q25 have lower values in all stress cases except cold stress compared to the whole set of *A. thaliana* genes. This holds true also for ‘All stresses nr’ gene set. These results demonstrate that DI distributions for stress genes are shifted towards smaller values.

It should be noted that the analysis above uses lists of all *A. thaliana* protein-coding genes (including both annotated and non-annotated genes in the GO database) as the background lists. At the same time, it is known that among young genes there is a high proportion of non-annotated ones [27]. This may lead to a shift in our estimates, since the non-annotated portion of genes that are known to be younger is excluded from the stress gene sample. It turned out that the number of non-annotated genes in the TAIR database was 1738 (6% of the total number of genes). We built the age distributions for both GO-annotated and non-annotated genes in the TAIR database (Supplementary file 2, Figure S3). Indeed, the proportion of young genes (PAI > 10, Pentapetalae) in the list of genes without annotations is systematically higher than for the same PAI values among genes with GO annotation. It should be noted, however, that this proportion is generally comparable to the proportion of old genes. Thus, the highest proportions of genes for PAI = 14 (Brassicacea) and 17 (*A. thaliana*) occurring among young taxa are 0.14 and 0.16, respectively. These values differ insignificantly from the proportions of non-annotated genes for Cellular organisms (PAI = 0) and Eukaryota (PAI = 1) phylostrata, 0.13 and 0.16, respectively.

However, to account for this bias, we conducted randomization tests for PAI and DI values using a sample of *A. thaliana* protein coding genes of only annotated genes (25,898 of 276,364, 94%) as the background. Results for PAI (at Identity = 0.5) and DI are shown in Supplementary file 2, Tables S4 and S5. The data of the Table S4 show that the significance of deviations of PAI share on phylostrata from the sample of annotated *A. thaliana* genes at the level of *p* < 0.05 is unchanged compared to the results of Table 2. Significantly low values are observed for all types of stress in Cellular Organisms. For Eukaryota phylostratum, significant deviations are observed for osmotic, salt, water stresses as well as for All stresses nr stress. For Viridiplantae, the water stress is significant. For Embryophyta, it is the heat stress. Finally, for Tracheophyta it is the cold stress. As for young phylostrata, the results for them are also consistent for the two types of background lists (high values).

A similar pattern is observed for DI tests. All significant values in Table 4 correspond to significant values in Table S5; moreover, for the interval DI [0, 0.1], significantly low values were found for light and water stress genes.

The presented results allow us to conclude that the genes associated with the response to various types of stress in general are rather more frequently under purifying selection than the average for the genome of *A. thaliana*.

### 3.4. Network Clusters and Their Association with GO Terms

We reconstructed gene networks for genes represented in seven stress gene lists using STRING tool [https://string-db.org/] (see Section 2.2). The networks contain genes that have at least one relationship with other genes at a significance level of 0.7 (high confidence). For some genes, interactions of the required level of significance were not found and these were not included in the network structure. In addition, STRING added several genes to the network due to their connections with genes from the stress lists. In some cases, the graphs of networks of the same type of stress included genes, not presented in the original list, but presented in some of the lists of genes associated with other types of stress. The list of genes included in the particular type of the stress gene network is presented in Supplementary file 3.

To verify the clustering of the gene network nodes, for each cluster we calculated: the average edge number per node within cluster edges (deg_int_, see Section 2.6), the average edge number per node for edges connecting cluster and non-cluster nodes (deg_ext_), and the average degree of the node for all network, *k*_net_. The results for each network and each cluster are represented in Supplementary file 2, Table S6. The table demonstrated that the majority of clusters have deg_int_ > *k*_net_ and all clusters have deg_int_ > deg_ext_ (all deg_ext_ values are below 0.5). These data justify our choice of clusters within stress gene networks.

**Figure 4:**
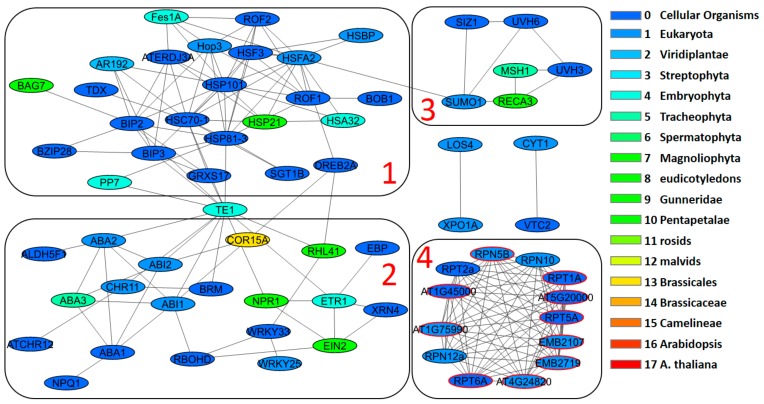
Gene network reconstructed for the heat associated gene set using the STRING tool. Node color corresponds to the PAI index of the gene from 0 (dark blue) to 17 (red). Nodes added to the gene set by the STRING procedure of network reconstruction are outlined in red color. The four clusters of genes are shown by rounded rectangles and numbered.

The network for heat stress genes was visualized using the Orthoscape application (Figure 4). In this network, four clusters were identified. Cluster 1 comprises 23 genes. 13 genes are coding for heat shock proteins performing chaperone functions (gene ID is shown after the slash): BOB1/AT5G53400, HSBP/AT4G15802, BAG7/AT5G62390, Fes1A/AT3G09350, HSP21/AT4G27670, HSF3/AT5G16820, BIP2/AT5G42020, AR192/AT4G26780, HSC70-1/AT5G02500, HSP101/AT1G74310, BIP3/AT1G09080, ATERDJ3A/AT3G08970, HSP81-3/AT5G56010. The 2 genes related to thioredoxin (GRXS17/AT4G04950, TDX/AT3G17880) and one gene, PP7/AT5G63870 is a housekeeping gene. Functions of other 8 genes in this cluster are less clear. It contains the majority of genes with low PAI and they have a dense network of interactions. Most of these genes are unique for the heat stress gene set. The GO terms (biological process), associated with this cluster in the largest number of genes, excluding ‘response to heat’ (Table S7, Supplementary file 2) are protein folding, response to hydrogen peroxide, cellular response to unfolded protein. The functions of this cluster are related to the mechanisms of cellular response to unfolded protein. Predominant cellular localization of genes for this cluster are cytosol and cytoplasm. Molecular function is protein binding, including Hsp70 (Table S7, Supplementary file 2).

Cluster 2 contains 20 genes. It includes transcription factors of WRKY (WRKY25/ AT2G30250, WRKY33/AT2G38470) and C2H2 (RHL41/AT5G59820) types, receptors for ethylene (ETR1/AT1G66340, XRN4/AT1G54490, EBP/AT3G16770), salicylic acid (NPR1/AT1G64280), abscisic acid (ABI1/ AT4G26080), hormone biosynthesis (ABA1/AT5G67030, ABA3/AT1G16540 are two enzymes controlling the first and the last steps of abscisic acid biosynthesis, respectively) and chromatin modifying protein ATCHR12/ AT3G06010. Cluster 2 contains more genes with higher PAI values and more genes shared with other stress types. The interaction network for this cluster is sparser in comparison with Cluster 1. The GO terms (biological process), associated with this cluster in the largest number of genes, excluding ‘response to heat’ (Table S7, Supplementary file 2) are response to abscisic acid, response to salt stress, response to water deprivation, response to osmotic stress, response to cold, cellular heat acclimation, response to ethylene, ethylene-activated signaling pathway. This set of genes is associated with a greater extent with the functions of the pathway of hormone signals for abscisic acid, ethylene and gene expression regulation. It significantly intersects with the functions of response to cold, salt, water, heat and osmotic stresses. No clear association with GO was found for cell localization of genes for this cluster. Molecular function was found to be protein binding (Table S7, Supplementary file 2).

Clusters 1 and 2 connected via hub gene, TE1 (ERECTA/AT2G26330), a receptor protein kinase, which is a pleiotropic regulator of developmental and physiological processes, as well as it is a modulator of responses to environmental stimuli [45], including heat stress [46].

Cluster 3 contains 6 genes. SUMO1/AT4G26840 [47] and SIZ1/ AT5G60410 [48] are related to ubiquitination. Two genes involved in the repair of strand breaks, and the excision repair in response to ultraviolet radiation: UVH6/AT1G03190 [49] and UVH3/AT3G28030 [50]. Two remaining genes from this cluster are involved in the mitochondrial genome stability, MSH1/AT3G24320 (a plant-specific protein involved in organellar genome stability in mitochondria and plastids [51]) and RECA3/AT3G10140 [52]. Like the other regulatory cluster, 2, cluster 3 has sparse interactions and large fraction of genes with medium/high PAI. The GO terms (biological process), associated with this cluster are mitochondrial genome maintenance, DNA repair. This cluster of genes is probably associated with maintaining the stability of the DNA structure during the response to heat stress. No clear association with GO was found for cell localization of genes in this cluster, as well as for molecular functions.

The fourth cluster contains 13 genes, 10 of which are the only genes added to the initial heat stress gene set by STRING. Genes from this cluster have no connections to other clusters via STRING interactions. They tightly interconnected within the cluster. All of genes included in this cluster are proteasomal genes. It is likely that the function of this cluster is related to the degradation of proteins unfolded due to the heat stress. It is confirmed by the GO terms for this cluster: ubiquitin-dependent protein catabolic process, ER-associated ubiquitin-dependent protein catabolic process, proteasome-mediated ubiquitin-dependent protein catabolic process. Cellular localization of genes for this cluster is primarily cytosol, cytoplasm, proteasome complex, etc. Molecular function annotation contains protein binding, hydrolase activity, proteasome-activating ATPase activity (Table S7, Supplementary file 2).

The remaining four genes outside clusters contain a pair of genes associated with the biosynthesis of ascorbic acid: CYT1/AT2G39770, GDP-D-mannose pyrophosphorylase VTC1 [53] и VTC2/AT4G26850 [54]. The two other genes, a DEAD box RNA helicase LOS4/AT3G53110 [55] and exprotin XPO1A/AT5G17020 [56], are associated with the export of mRNA. These four genes have low PAI values (less than 3).

Thus, the gene network of response to heat stress is segregated into several distinct clusters (modules), the genes in which perform quite specific functions. One of the clusters, “regulatory”, is quite large in size, and is characterized by genes of signal response, regulation associated with hormones (ABA, ethylene), and genes associated with other types of stress responses.

A similar structure of gene networks is typical for other types of stress (see Figures S4–S9, Supplementary file 2). We have also identified distinct clusters in them (up to 6 PCs), as well as several non-clustered genes. Perhaps the most remarkable feature of the considered networks is the isolation of the “regulatory” cluster of genes of response to cold, salt, osmotic and water stresses. These are the clusters with the number of 1, which include the greatest number of genes in all these networks except oxidative. The proportion of genes involved in these clusters is greater than that of a similar cluster in the heat stress network (Figure 4); they are linked by a large number of interactions. Interestingly, these clusters contain genes associated with known hormone response to abiotic stress such as ethylene, abscisic acid, jasmonate [57,58,59]. Among these can be mentioned the genes associated with the biosynthesis of abscisic acid (ABA1 zeaxanthin epoxidase), regulation of its biosynthesis (phosphatases ABI1, ABI2, molybdenum cofactor sulfurase ABA3), as well as transcription factors, which regulate this metabolite (abscisic acid receptor PYR1). Detailed information is presented in Tables S8–S13 (Supplementary file 2). These regulatory clusters also contain genes associated with ethylene signaling pathways and signal transduction genes, such as SnRK2 protein kinases, that are involved into the signal pathway of response to abiotic stress [60]. It is important to note that regulatory clusters contain mainly ancient genes.

It is interesting to note that the expressed regulatory clusters associated with stress hormones are not represented in the networks of response to light and oxidative stress. For example, the largest cluster in the light stress gene network contains the genes of response to light stimuli, regulatory genes, photomorphogenesis and circadian rhythm genes (Figure S5, Table S9, Supplementary file 2). A small cluster 2 containing only three genes is associated with auxin, and cluster 3 (6 genes) with abscisic acid-activated signaling pathway.

For oxidative stress, there is no clear ranking of clusters by the number of genes; they are all about the same, with rare connections (Figure S7, Table S11, Supplementary file 2). It is also difficult to distinguish a separate regulatory component. Genes associated with other types of stress are also rare. Only a small number of genes is associated with the terms “cellular response to highlight intensity” and “protein repair, protein folding”.

As for the functions of other clusters in gene networks, they are primarily related to the molecular mechanisms of stress response. For example, in a cold stress response network, cluster 2 includes cold shock proteins such as GRP2 (the cell wall glycine-rich protein), CSP3 (an RNA chaperone, cold shock domain protein 3 [61], GR-RBP2 (glycine-rich RNA-binding protein 2) which participate in DNA/RNA melting processes. There are also clusters of regulatory proteins and signal transduction, such as small-size clusters with the number 2 in the osmotic and salt stress networks that contain kinases (Figures S6 and S8, Supplementary file 2).

Thus, gene networks of response to different types of stress differ both in network topology and in functions associated with genes. In the networks of response to cold, salt, osmotic and water stress, the regulatory component is clearly distinguished, which includes a large number of genes, with a large number of links between them. This component is associated with known abiotic stress hormones, abscisic acid and ethylene, and contains many genes common to these types of stress, which is in good agreement with the clustering diagram of stress response gene lists (Figure S1, Supplementary file 2). In the network of heat stress, such a regulatory component is less apparent, and in the networks of light and oxidative stress it is almost absent.

### 3.5. Relationship between Structural and Evolutionary Characteristics of Gene Networks

**Figure 5:**
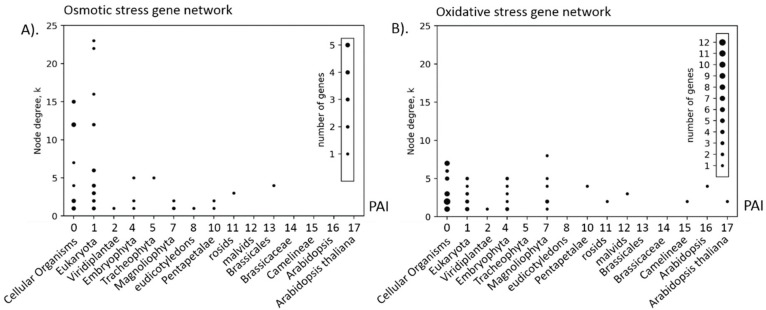
The PAI versus *k* scatterplots for osmotic (**A**) and oxidative (**B**) stress gene networks. The X-axis represents the PAI, the Y-axis shows node degree *k*.

We checked the relationship between structural characteristics of gene networks such as the connectivity degree of the node (*k*) and the evolutionary age of the gene (PAI). The results are shown in Table 5. The PAI versus *k* scatterplots for osmotic and oxidative stress are shown in Figure 5 (panels A and B, respectively).

Table 5 shows that significant negative correlations between the node degree of connectivity and the age of the corresponding gene are observed only for three types of stress. These are networks of heat, osmotic and salt stress. Such correlations are due to the presence of nodes in the network that correspond to ancient genes with a large number of connections, which is clearly seen in the scattering diagram for the osmotic stress network (Figure 5A, the upper left corner of the graph) and is not observed for the oxidative stress network (genes are distributed approximately equally by the age, regardless of the age value, see Figure 5B). In the network of osmotic stress, such nodes are ABI1/AT4G26080 gene (*k* = 23) and ABI2/AT5G57050 gene (*k* = 22), which belong to the Protein phosphatase 2C family (Figure S6, Supplementary file 2). They contain a large number of edges linked to the nodes of the PAI equal to 1 (Eukaryota phylostratum). The oxidative stress gene network does not contain nodes with such a high degree value (the maximum value of 8 is represented for the gene STZ/AT1G27730, salt tolerance zinc finger; see Figure S7, Supplementary file 2). It should be noted, however, that Table 5 does not contain significant positive correlation coefficients. Thus, it should be concluded that in the networks of stress response, young genes do not have node degrees significantly greater than that of old genes. This is clearly seen in the scatter plots shown in Figure 5 and Figure S10 (Supplementary file 2).

We evaluated the interrelation of the ages of the genes forming the gene networks of various types of stress. To do this, we calculated the distribution of the absolute value of the gene age difference (|dPAI|) for pairs of nodes of the gene network connected by edges. For comparison, we calculated similar distributions for pairs of nodes in a graph separated by the shortest path of 2 and 3 or more edges. The results are shown in Figure S11 (Supplementary file 2). It can be seen that for all types of stresses, except for light, for nodes separated by one edge, the most common is the pair with the same age (|dPAI| = 0). For light stress, these were pairs with a value of |dPAI| = 6. From the graph of the gene network of the light stress (Figure S5) one can see that it contains a large cluster (#1) including 34 genes, 17 of which are of PAI values equal to 0 (Cellular organisms) or 1 (Eukaryota). The remaining 17 genes have PAI values > 4 (Embryophyta) and have many interactions with ancient genes. Such a structure of the graph gives a high occurrence of pairs of interacting genes, the age difference in which is ranged from 4 to 9. Presumably, in this network, in the later stages of evolution, there was an intensive inclusion of new genes, due to the formation of interactions with the already existing backbone of the network, and not due to the addition of modules of age-homogeneous genes.

We estimated the scalar coefficient of assortativity for gene ages in the stress-related networks, as well as the variance of these estimates by using the package graph_tools [https://graph-tool.skewed.de/]. This coefficient reflects the correlation of PAI values between pairs of genes forming in interaction networks. The higher the coefficient is, the stronger is the relationship between the ages of the interacting genes. The results are shown in Table 6.

The assortativity coefficients occurring were positive for all types of stresses. For cold, heat, osmotic, oxidative and salt stress, they were high enough. The highest value, 0.567, was found for oxidative stress, in other types it was above 0.12. Thus, if the age of one of the genes in the interacting pair is higher, then the age of the second gene will also be higher (and vice versa, if the age of one gene is less, then the second will be less too). However, these correlation coefficients were comparable with the values of standard deviations, which makes it impossible to talk about their high significance.

### 3.6. Evolution of Gene Network Function

**Figure 6:**
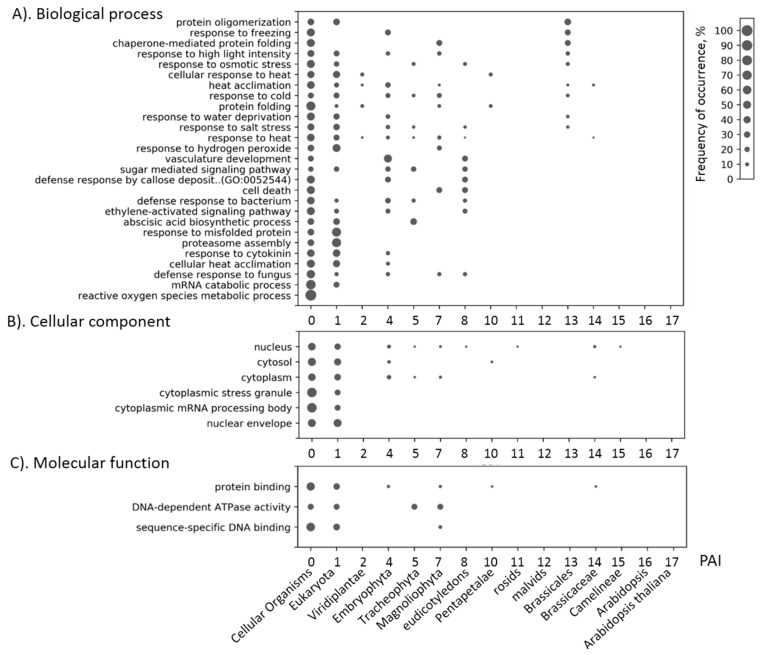
PAI distribution for genes associated with the heat stress and annotated by various GO terms: (**A**) GO terms for “biological process”; (**B**) GO terms for “cellular component”; GO terms for “molecular function”. The frequency of PAI occurrence is normalized to 100% for each GO term and shown by circles. The scale of circles shown on the right.

The difference in the ages of genes in gene networks suggests that in the process of evolution, new genes that could introduce new functions for this network were added to the “backbone” of the initial network, which was formed in the most ancient taxa. We decided to investigate how the functions of stress-related gene networks changed in the course of evolution, from phylostratum to phylostratum. To do this, we chose all GO terms significantly associated with the genes of each type of stress (Supplementary file 5). For each term, we have identified the associated genes. For these genes, we built a PAI distribution. An example of a diagram integrating such distributions for GO terms associated with the heat stress is shown in Figure 6. In this diagram, PAI (phylostratum) values are plotted along the X-axis. Vertically is a list of GO terms. Next to each term and age of the gene a circle is located, the size of which is proportional to the fraction of genes associated with the term and having the corresponding PAI value. For example, at the bottom of the diagram, for the term ‘reactive oxygen species metabolic process’ and PAI = 0 one can see a large circle. This means that in this gene network, all genes annotated with this term have the same value PAI = 0 (Cellular organism). For the keyword ‘response to salt stress’, it can be seen that genes associated with this term were added to the network of heat stress in the stages of formation of such phylostrata as Cellular organisms, Eukaryota, Embryophyta, Tracheophyta, Magnoliophyta and Brassicales, but the proportion of ancient genes (PAI = 0 and 1) is higher, which is reflected by the larger size of the corresponding circles.

A special feature of the data obtained (Figure 6) is that the gene networks of one type of stress contain genes that are associated with several other types of stress. For example, for a heat stress network, the terms related to cold stress (‘response to freezing’, ‘response to cold’) were observed for TIL/AT5G58070 gene encoding temperature-induced lipocalin, an essential component for thermotolerance probably acting against lipid peroxidation induced severe heat stress [62]. Its PAI is 4 (Embryophyta), and it is also annotated by GO terms ‘response to water deprivation’, ‘response to cytokinin’, ‘response to high light intensity’, ‘heat acclimation’, ‘response to heat’ (Supplementary file 3). TIL1 of Arabidopsis is localized in the plasma membrane [63] and its expression is increased in response to cold stress suggesting a cryoprotective role of the lipocalin-like protein under freeze-induced dehydration [64]. It was also shown that these proteins are translocated under salt stress and protect chloroplasts from ion toxicity [65]. Thus, the relationship of heat stress genes to several the above-mentioned biological processes can be explained by the existence of the multifunctional gene of the lipocalin family, TIL.

Another interesting feature of this chart is the fact that all the GO terms associated with the network response to heat stress, was presented in the annotation of genes of the most ancient phylostrata: Cellular organisms and Eukaryota. This is seen in the first two columns of circles that are observed for all GO terms.

A relatively small fraction of the terms were associated with genes whose age was small (PAI > 8). Thus, the obtained picture of changes in the GO composition depending on the age of genes in the network shows that the overwhelming number of functions associated with the work of the gene network is associated with ancient genes.

This result was expected because, as was shown above, among the genes associated with stress, there is a high proportion of ancient genes. However, for some terms this looks counterintuitive. For example, in Figure 6 the GO term ‘vasculature development’ matches not only the genes represented in phylostrata of higher plants (Embryophyta, eudicotyledons), but also those in ‘Cellular organism’ phylostratum, i.e., genes associated with the processes of development of vessels already existing at this stage of evolution, when organisms’ vessels had not yet formed. In the heat stress network, the term ‘vasculature development’ refers to the genes ETR1/AT1G66340 (Signal transduction histidine kinase), TE1/AT2G26330 (Leucine-rich receptor-like protein kinase family protein), XRN4/AT1G54490 (exoribonuclease 4), EIN2/AT5G03280 (NRAMP metal ion transporter family protein) (Supplementary file 3). Two of them, TE1 and ETR1 have the PAI = 4 (Embryophyta), one, EIN2, has PAI = 8 (eudicotyledons), and the XRN4 gene is the most ancient (PAI = 0, Cellular Organisms). The XRN4 gene (also known as EIN5) is an еndogenous suppressor of posttranscriptional gene silencing by preferential degradation of select substrates [66]. It is involved in the regulation of Ethylene Response Pathway [67]. This gene is also involved in regulating the response to heat stress. As was shown by Nguyen et al. [68] using the microarray analysis and mRNA decay assay, the loss of AtXRN4 function caused a reduction in the degradation of heat shock factor A2 (HSFA2) and ethylene response factor 1 (ERF1) mRNA, resulting in increased survival rate when plants subjected to a short-term severe heat stress. At the same time, the XRN4 is necessary for the thermotolerance of plants to long exposure to moderately high temperature. It is involved in the degradation of the *A. thaliana* transcriptome that occurs during the early steps of the heat stress response [69]. At the same time, the participation of this gene in the regulation of vascular cell division is shown [70]. This division is maintained by an interaction between the PXY and ethylene signaling [70]. Thus, the XRN4 gene is multifunctional, it takes part in a specific degradation of RNA, and is involved in several different biological processes related to both the response to stress and the development of plant tissues.

Similar diagrams were constructed for other types of stress. They are presented in Supplementary file 2 (Figures S12–S17). As in Figure 6, a special feature of these diagrams is that the vast majority of the GO terms have been associated with ancient genes (PAI = 0 and 1). For example, the ancient genes of the response to the light stress are involved in the regulation of flower development (Figure S13, Supplementary file 2), ancient genes for the response to the osmotic stress are involved in seed development (Figure S14, Supplementary file 2), and the salt stress genes—in the regulation of seed dormancy process (Figure S16, Supplementary file 2).

The results obtained (Figure 6, Figures S12–S17) indicate the multifunctionality of the ancient genes involved in the response to stress, up to their participation in processes that had already been formed in plants in the later stages of evolution.

## 4. Discussion

The study of the principles of evolution of the structure and function of gene networks is one of the most interesting and important tasks in biology [71]. The results of such analysis not only have theoretical interest, but also predictive power [72,73].

Such evolutionary characteristics of genes as phylostratigraphic age [21] and the index of divergence [23] reflect the most important properties of genes: the order of their appearance in the genome in relation to other genes and the degree of selection pressure in the process of evolution. These metrics allow us to characterize the dynamics of changes in the function of gene sets, including gene networks. In particular, a comparison of the ages of genes and the annotation of their functions (GO) allows one to explore the relationship between the function of genes/organisms and the age of genes [20,74]. As a rule, this is done by searching for associations of genes of different ages with the GO terms [26]. For example, Domazet-Loso and Tautz showed that genes related to human genetic diseases are significantly overrepresented among the genes that have emerged during the early evolution of the metazoan [20]. Ruprecht and coauthors [26] performed Mapman terms phylostrata enrichment analysis for three planta genomes, including *A. thaliana*, and showed that significant occurrence of the terms in different phylostrata mostly happens only once, suggesting that new biological features emerged in (or during) distinct evolutionary periods, without a significant addition of new genetic material during later stages of evolution.

In this paper, we selected the genes associated with plant response to different types of stress and analyzed these gene sets, including reconstruction of interaction networks, evaluation of phylostratigraphic age and selection pressure. We compared the evolutionary properties of these sets of genes with those of the complete set of *A. thaliana* genes and found their significant differences.

Our results show that the genes associated with the stress response generally contain a large number of ancient genes than would be expected from the distribution of such genes throughout the genome. In addition, stress genes are more conservative than would be expected from the conservatism of the whole set of genes. These data are in line with the concept that the more important genes in terms of function are older and are under strong pressure of stabilizing selection [75,76,77]. Genes with high connectivity in the interaction networks have similar evolutionary properties [78] and evolve under stabilizing selection [79,80,81,82,83]. The response of plants to stress, of course, involves the basic functions of the cell that were formed at the earliest stages of evolution, such as, for example, the system of heat shock proteins forming a noticeable cluster in the heat stress response network (Figure 4), or a cluster of genes of signaling pathways controlled by stress hormones (Supplementary file 2, Figures S4, S6, S8 and S9), where their fraction is significant. In response to stress factors of biotic and abiotic nature in animals and plants, many parallels can be found at the level of physiology [84], and especially at a cellular level [85,86,87]. It is known that stress response genes are homologous in plants and animals. For instance, stress associated proteins (SAPs) in plants contain A20/AN1 zinc finger domain homologous to proteins from the genomes of diverse organisms including protists, fungi, animals, and plants [88]. We found these genes among the genes associated with the response to osmotic and water types of stress (SAP5/AT3G12630, A20/AN1-like zinc finger family protein, Supplementary file 3).

Our analysis involves 15 phylostrata of organisms represented in KEGG database [33]. The distribution of genes across these phylostrata reveals an interesting feature: the presence of three peaks in the distribution (Figure 2). The most ancient peak (PAI < 2), contains about a half of all genes. The other peaks correspond to PAI = 7 (Magnoliophyta) and to PAI = 14 (Brassicacea). The position of these peaks does not change from the change in the value of the identity threshold when determining orthologs (see Supplementary file 2, Figure S2), although the ratio of gene fractions varies. We can assume that these two peaks correspond to the events of the whole genome duplication in *A. thaliana* lineage, an α-duplication that preceded the formation of the Brassecaceae clade [89,90,91,92] and more ancient γ-duplication, which corresponds to the angiosperms ancestor [93]. It is known that whole genome duplications are substantial events in organisms’ evolution, leading to the emergence of many novel genes [94,95]. The presence of two younger peaks in the PAI distribution for *A. thaliana* probably reflects this feature of evolution. It is interesting to note that for γ-duplication, the difference between the fractions of genes of this phylostratum in stress-related genes and in the whole genome of *A. thaliana* is close to zero or even positive (except for the genes of heat and water stress). This means that during the diversification of duplicated genes at this stage of evolution, new stress response genes have emerged. For α-duplication (Brassicaceae phylostratum) on the contrary, this difference is negative, and large in absolute value for all stress genes. We can speculate that at this stage, after the duplication, there was a sharp loss of duplicated stress genes (relative to other types of genes). Apparently, by that time, in general, the systems of response to various stresses in plants were mostly formed and they did not have a need for evolutionary innovations (compared to the increase in innovations in other gene systems). It should be noted; however, that Orthoscape in its current version cannot account for the possible bias in the phylostratigraphic assignment due to these duplications because we did not consider genome synteny when defining orthologous groups. The influence of such bias can be resolved in a future research. 

Stress genes are also under a strong pressure of purifying selection, which indicates their absolute importance for the organism (although few of them are absolutely conservative). Among them, very rarely (only two cases) are genes subject to positive Darwinian selection (DI > 1), which is very small compared to the proportion of such genes in the entire genome. This is generally consistent with data from Lei et al., according to which *A. thaliana* genes of DI > 1 are enriched in lipid localization, transport and binding, and the endomembrane system (i.e., stress unrelated terms) [37]. However, it should be noted that the use of the genome pair *A. thaliana* vs. *A. lyrata* for DI estimates (i.e., Ka/Ks) might provide insight into genes that evolved under different selection regimes only in the most recent past. Of course, in this case, it is hard to expect that any genes performing the basic functions of the organism can be affected by the positive selection. On the other hand, the results show that most stress genes at this stage of evolution are the subject to stabilizing selection, which is quite consistent with the hypothesis of their functional importance and the performance of their basic functions.

Analysis of the relationship between the structural characteristics of the reconstructed networks showed that for such networks as heat, osmotic and salt stress, the higher is the order of gene interactions in the network, the greater is the age of the gene. However, for other networks, we found no significant correlations. Apparently, these results are affected by the topology of the networks, which turned out to be different. For example, the oxidative stress network does not have a clearly defined large regulatory cluster; the light stress response network does not contain nodes with a large number of connections. It was also shown that a significant part of the interacting pairs of genes have the same age (except for light stress genes). This suggests that interactions in the network are preferable for genes of similar age (or that clusters of genes are generally homogeneous in age of genes). This trend is also indicated by the positive assortativity coefficients of the age of genes. Similar results were obtained in the study of Ruprecht [26], where it was shown that genes from the same evolutionary period tend to be connected, whereas old and young genes tend to be disconnected. This trend is not, however, a general rule, as confirmed by the structural analysis of the light stress network.

Our results show polyfunctionality of the stress-associated genes in agreement with the current knowledge [96]. A lot of GO terms, in addition to the stress terms we used, was found among the annotations of our gene lists. This can be explained by the fact that response of plants to stress of any nature affects a large number of molecular processes [3]. For example, the heat stress leads to the triggering of such processes in plant cells as change in membrane fluidity, increase of the reactive oxygen species (ROS), change in the transport of Ca^+^ ions and restructuring of the cytoskeleton, the denaturation of proteins and RNA, changing the structure of chromatin and the expression of miRNAs [97]. The heat stress activates heat shock proteins, sumoylation systems, chromatin remodeling, dehydration control [7]. The drought stress activates specific signaling pathways and transcription factors, detoxification enzymes, enzymes of the biosynthesis of osmolytes, the system of transporters and water channels, response to protein denaturation [98]. In response to the salt stress, genes of photosynthesis and carbon production, cell wall components, water channels, ion transport, ROS protection system, a detoxification system, signaling pathways and specific transcription factors are involved [99]. It should be noted that the system of response to the osmotic and the oxidative stress themselves are involved in responses to other types of abiotic stress [100]. Thus, the systems of response to abiotic stresses in plants are closely interconnected. Our analysis of annotations of the stress genes in *A. thaliana* indeed has shown that the involvement of some genes in several stress responses is one of the features of stress genes (Figure S1, Supplementary file 2). 

The presence of common and unique genes can be explained also by the multilevel structure of molecular systems of response to stress [3,101]: as a rule, these systems include stress sensors, signal transmission systems (including hormonal response), triggering transcription of stress response genes, molecular response to the occurrence of stress conditions to minimize its consequences. Systems of the first and second level, as well as partly the regulation of genes, are mainly specific for each type of abiotic stress. At the same time, the molecular response to cell stress for different types of stress has many common features: control of reactive oxygen species (ROS), change of ion transport, cell detoxification, control of protein denaturation. In our work, we demonstrated the existence of large regulatory cluster of genes common to cold, osmotic, heat, salt and water-related types of stress, which includes various regulatory, hormone-related and signal transduction genes. 

Another possible reason for the generality of genes for different types of stress is that in nature, stress factors often act together, and in the course of evolution plants develop shared responses which are common to individual stresses and stress combinations [102].

Several ancient genes of stress response were involved in biological processes that occurred at much later stages of evolution compared to the time of occurrence of these genes. The explanation for this may lie in the fact that during the evolution of plants the old genes were intensively involved for the formation of new functions, so they are involved in a new functional context [103].

Interestingly, our analysis shows that ancient stress genes have many properties in common with so-called multifunctional genes [104]: they are also highly conservative, involved in several biological processes, tend to form a large number of connections in the structure of gene networks and are involved in the performance of important functions in the life of organisms.

It should be noted that the analysis strongly depends on the results obtained at the stage of the formation of stress gene sets, as well as the reconstruction of gene networks, since it is completely based on these data. We had this in mind and chose rather strict criteria for the selection of genes by the GO terms and for the reconstruction of gene networks by the STRING method. Of course, these data may be incomplete and contain errors. However, a comparison of the composition of stress genes (Figure S1) demonstrates the commonality of several genes for different types of stress especially noticeable for stresses for which their hormonal control (ABA, ethylene and jasmonates) is known: salt, cold, osmotic, water stresses and to a lesser extent heat one. The structure of gene networks for these stresses also demonstrates the presence of a large cluster represented by genes associated with the perception and transmission of hormone signals (Figure 4, Figures S4, S6, S8 and S9, Supplementary file 2). This is in good agreement with the known role of hormones in regulating the response to abiotic stress [57,58,59]. Gene networks built on the basis of co-expression are definitely more common, if we talk about choosing a strict threshold to establish intergenic associations and can include a large number of genes, in comparison with networks built via STRING. However, the level of gene expression in response to stress strongly depends on the time elapsed after stress exposure, with many side processes that are secondary to the stress response itself, which complicates the interpretation of expression data. Therefore, we decided to use data based not only on co-expression, but also on broader information (GO annotation, which is-based, among other things, on expert data; STRING networks, which include information on co-expression along with protein-protein interactions and other additional information) in the selection of stress genes and reconstruction of their networks.

## 5. Conclusions

Our results demonstrate that the use of data on the structure of gene networks along with phylostratigraphic information allows us to describe the evolution of stress genes in plants and its relationship with the response to various abiotic stresses in the context of the structure of gene networks more fully.

## Figures and Tables

**Table 1:** Number of GO terms and genes that have identified associations with studied stress types.

Stress Type	Number of GO Terms	Number of Genes	KEGG Number of Genes
Cold	4	150	144
Heat	14	102	102
Light	48	155	141
Osmotic	23	116	114
Oxidative	28	154	152
Salt	17	231	230
Water	27	215	211

**Table 2:** The number of common genes between pairs in stress-related gene sets. Each cell in the table represent the fraction (and number, in parentheses) of genes from the set of the row common with the set of the column. The last column represents the number of unique genes for the stress in the row.

	Salt	Heat	Light	Water	Cold	Osmotic	Oxidative	Unique Genes
Salt	232	13	7 (0.03)	41 *	18	**47**	18	126
		−0.06		**(0.18)**	−0.08	**−0.2**	−0.08	−0.54
Heat	**13**	102	8	**11**	8	8	6	72
	**−0.13**		−0.08	**−0.11**	−0.08	−0.08	−0.06	−0.71
Light	7	8	155	12	9	3	6	120
	−0.05	−0.05		−0.08	−0.06	−0.02	−0.04	−0.77
Water	**41**	11	12	216	18	**29**	11	124
	**−0.2**	−0.05	−0.06		−0.09	**−0.14**	−0.05	−0.59
Cold	**18**	8	9 (0.06)	**18**	150	**17**	6	93
	**−0.12**	−0.05		**−0.12**		**−0.12**	−0.04	−0.64
Osmotic	**47**	8	3	**29**	**17**	117	12	35
	**−0.41**	−0.07	−0.03	**−0.25**	**−0.15**		−0.1	−0.3
Oxidative	**18**	6	6	11	6	12	154	118
	**−0.12**	−0.04	−0.04	−0.07	−0.04	−0.08		−0.77

* The cells with fraction of genes larger than 0.1 are shown in bold.

**Table 3:** The comparison of the PAI distribution of genes in the gene networks of *A. thaliana* stress response with the corresponding distribution of the complete set of *A. thaliana* genes according to the results of the permutation test. First line: types of stress. Second line: the proportion of random samples for which the average PAI_rand_ value for a set of genes, the same size as the stress network, exceeds the PAI_stress_ value for the corresponding stress network. Third row: fraction of random samples of genes in which the value of the quadratic deviation ChiSq_rand_ distribution of ages the distribution for all genes is higher than in the corresponding gene networks (ChiSq_stress_). The fifth and subsequent lines: the fraction of random samples of genes in which the difference between the proportions of genes of *i*-th phylostratum dfPAI*_i_* among stress genes exceeds the corresponding proportion among random sample formed from the whole gene set. All values in the cells must be multiplied by 10^−5^. PAI is calculated at the level of similarity of the sequences of ID = 0.5.

Stress	Cold	Heat	Llight	Osmotic	Oxidative	Salt	Water	All Stress nr
*p*(PAI_stress_ < PAI_rand_)	100,000 *	100,000	100,000	100,000	100,000	100,000	100,000	100,000
*p*(ChiSq_stress_ < ChiSq_rand_)	**163**	**153**	**303**	**2132**	**20**	**1**	**1**	0 **
*p* (dfPAI*_i_*_stress_ < dfPAI*_i_*_rand_) for specific phylostratum								
00_Cellular organisms	**28**	0 **	**11**	**1051**	0 **	0 **	**153**	0 **
01_Eukaryota	11,218	35,449	34,137	**2009**	75,372	**194**	**34**	**78**
02_Viridiplantae	15,729	11,571	6603	32,387	18,710	83,664	**96**	6394
04_Embryophyta	47,489	**3778**	12,708	51,099	11,320	87,444	26,046	25,790
05_Tracheophyta	**2181**	65,732	32,104	51,865	99,386	22,936	39,481	53,618
07_Magnoliophyta	38,170	97,432	26,294	23,881	49,013	45,825	82,207	100,000
08_eudicotyledons	94,830	62,021	78,520	8542	60,328	69,323	80,882	100,000
10_Pentapetalae	69,619	72,175	68,242	31,311	52,357	50,365	60,197	97,992
11_rosids	91,840	79,304	91,447	83,906	81,256	99,084	72,339	99,935
12_malvids	15,610	39,144	49,460	42,420	16,775	67,429	63,996	78,703
13_Brassicales	89,388	94,844	61,746	89,433	96,971	97,955	98,746	100,000
14_Brassicaceae	99,913	89,314	99,872	99,981	99,765	99,998	100,000	100,000
15_Camelineae	89,271	74,850	97,699	80,248	56,525	99,796	99,627	100,000
16_Arabidopsis	74,084	61,724	73,454	65,695	41,687	88,476	86,494	99,736
17_A. thaliana	99,520	97,657	99,446	98,525	97,458	99,811	99,948	100,000

* The values with *p* < 0.05 are bold and underlined; the values with *p* > 0.95 are underlined. ** *p* < 10^−5^.

**Table 4:** Comparison of the divergence index (DI) distribution of genes in the gene networks of *A. thaliana* stress response with the corresponding distribution of the complete set of *A. thaliana* genes according to the results of the permutation test. First line: types of stress. Second line: the proportion of random samples for which the average DI_rand_ value for a set of genes, the same size as the stress network, exceeds the DI_stress_ value for the corresponding stress network. Third line: fraction of random samples of genes in which the value of the quadratic deviation ChiSq_rand_ distribution of DI from such distribution for all genes is higher than in the corresponding gene networks (ChiSq_stress_). The fifth and subsequent lines: fraction of random samples of genes in which the difference between the proportion of genes of *i*-th phylostratum dfDI_i_ among stress genes exceeds the corresponding proportion among a random sample formed from the whole gene set. All values in the cells must be multiplied by 10^−5^.

Stress	Cold	Heat	Light	Osmotic	Oxidative	Salt	Water	All Stresses nr
*p*(DI_stress_ < DI_rand_)	96,272 *	99,700	99,993	99,993	98,140	100,000	99,999	100,000
*p*(ChiSq_stress_ < ChiSq_rand_)	7010	43,398	**4707**	**589**	52,740	**10**	**77**	0 **
*p*(N_DI stress_ < N_DI rand_) for specific DI bin								
[0, 0.1]	93,375	28,064	6137	53,230	38,498	10,701	38,527	**3109**
(0.1, 0.2]	**67**	**2567**	**440**	**25**	**2633**	0 **	**3**	0 **
(0.2, 0.3]	15,832	38,676	66,383	**3174**	64,490	32,404	9731	18,452
(0.3, 0.4]	49,562	40,493	90,569	96,058	69,027	97,027	96,333	99,395
(0.4, 0.5]	71,783	75,719	58,004	92,193	30,113	96,979	94,603	99,451
(0.5, 0.6]	70,023	73,560	68,603	92,023	86,198	99,567	99,808	99,973
(0.6, 0.7]	95,009	84,956	99,071	88,993	22,528	93,999	82,333	99,957
(0.7, 0.8]	76,327	58,418	51,318	88,964	55,802	93,241	77,350	98,435
(0.8, 0.9]	47,813	67,878	79,538	72,465	50,554	92,166	90,674	99,617
(0.9, 1]	61,938	49,902	61,515	53,707	64,025	78,219	75,853	99,712
(1, +∞)	62,703	78,089	87,708	81,801	89,300	96,544	82,213	99,985

* Values with *p* < 0.05 are bold and underlined; values with *p* > 0.95 are underlined. ** *p* < 10^−5^.

**Table 5:** Pearson correlation coefficients *r* (*k*, PAI) between the node degree *k* and its PAI value in gene networks of different stresses. The second column shows the value of the correlation coefficient, the third one shows the significance level of its difference from 0.

Stress	*r*(*k*, PAI)	*p*-Value
Сold	0.004	0.974
Heat	−0.361 *	**0.003**
Light	−0.125	0.248
Osmotic	**−0.379**	**0.006**
Oxidative	0.019	0.875
Salt	**−0.266**	**0.006**
Water	−0.061	0.524

* Values for *p* < 0.05 are shown in bold.

**Table 6:** The coefficients of assortativity *r_a_* for gene ages in stress-related gene networks and estimates of their standard deviation *σ*(*r_a_*). *Np*-number of pairs of interacting genes.

Stress	*Np*	*r_a_*	*σ(r_a_)*
Сold	148	0.251	0.345
Heat	196	0.126	0.294
Light	178	0.026	0.348
Osmotic	148	0.192	0.327
Oxidative	111	0.567	0.344
Salt	203	0.143	0.293
Water	213	0.031	0.307

## Supplementary Materials

The following are available online at https://www.mdpi.com/2073-4425/10/12/963/s1, ‘Supplementary file 1.xlsx’: List of GO terms characterizing seven types of plant stress response. ‘Supplementary file 2.pdf’: Supplementary Tables S1–S13, Supplementary Figures S1–S17. ‘Supplementary file 3.xls’: Lists of genes, associated with 7 types of abiotic stress. ‘Supplementary file 4.xls’: PAI and DI data for all protein coding genes of Arabidopsis thaliana. ‘Supplementary file 5.pdf’: Gene ontology terms associated with stress-associated genes obtained using DAVID server.
