# Supplementary material for: Phylostratigraphic Analysis Shows the Earliest Origination of the Abiotic Stress Associated Genes in A. thaliana
Source: Genes (Basel). 2019 Nov 22;10(12):963. doi: 10.3390/genes10120963 (PMC6947294; doi:10.3390/genes10120963)
Supplement: Supplementary file 1 [file genes-10-00963-s001.zip › Supplementary file 2_final.pdf]

Supplementary materials for Mustafin et al. "Phylostratigraphic Analysis Shows the Earliest Origination of the Abiotic Stress Associated Genes in A. thaliana"

File content:

Table S1……………………………………………………………………………………….2
Table S2……………………………………………………………………………………….3
Table S3……………………………………………………………………………………….3
Table S4 ……………………………………………………………………………………....4
Table S5……………………………………………………………………………………....5
Table S6……………………………………………………………………………………......6
Table S7……………………………………………………………………………………......7
Table S8……………………………………………………………………………………..10
Table S9……………………………………………………………………………………....13
Table S10…………………………………………………………………………………....16
Table S11…………………………………………………………………………………....18
Table S12…………………………………………………………………………………....20
Table S13…………………………………………………………………………………....23
Figure S1…………………………………………………………………………………….26
Figure S2…………………………………………………………………………………….27
Figure S3…………………………………………………………………………………….28
Figure S4…………………………………………………………………………………….29
Figure S5…………………………………………………………………………………….30
Figure S6…………………………………………………………………………………….31
Figure S7…………………………………………………………………………………….32
Figure S8…………………………………………………………………………………….33
Figure S9…………………………………………………………………………………….34
Figure S10…………………………………………………………………………………..35
Figure S11…………………………………………………………………………………..36
Figure S12…………………………………………………………………………………..37
Figure S13…………………………………………………………………………………..38
Figure S14…………………………………………………………………………………..39
Figure S15…………………………………………………………………………………..40
Figure S16…………………………………………………………………………………..41
Figure S17…………………………………………………………………………………..42

Table S1. The comparison of the PAI distribution of genes in the gene networks of *A. thaliana* stress response with the corresponding distribution of the complete set of *A. thaliana* genes according to the results of the permutation test. First line: types of stress. Second line: the proportion of random samples for which the average PAI_{rand} value for a set of genes, the same size as the stress network, exceeds the PAI_{stress} value for the corresponding stress network. Third row: fraction of random samples of genes in which the value of the quadratic deviation $ChiSq_{rand}$ distribution of ages the distribution for all genes is higher than in the corresponding gene networks ($ChiSq_{stress}$). The fifth and subsequent lines: the fraction of random samples of genes in which the number Ng_{rand} of genes of the corresponding phylostratum exceeds the number Ng_{stress} (the same phylostratum, taken from network). All values in the cells must be multiplied by 10^{-5}. The values with $p<0.05$ are bold and underlined; the values with $p>0.95$ are underlined. PAI is calculated at the level of similarity of the sequences of ID=0.6.

stress	cold	heat	light	osmotic	oxidative	salt	water
$p(PAI_{stress}<PAI_{rand})$	100000	100000	100000	100000	100000	100000	100000
$p(ChiSq_{stress}<ChiSq_{rand})$	**223**	**2**	**5**	**122**	**5**	**0***	**0***
$p(Ng_{stress}<Ng_{rand})$ for specific phylostratum							
00_Cellular Organisms	**132**	**4**	**3402**	**1227**	**0***	**1247**	42477
01_Eukaryota	**2881**	**4**	57925	**61**	21089	**0***	**0***
02_Viridiplantae	44198	71057	**2157**	51748	16205	49038	**38**
04_Embryophyta	5889	11686	5827	48195	**1919**	8738	7123
05_Tracheophyta	60878	**3456**	**8**	25283	**3905**	26062	**2170**
07_Magnoliophyta	6147	56463	**1713**	44252	40108	66387	63483
08_eudicotyledons	13366	60082	25812	96940	67407	92191	86742
10_Pentapetalae	57104	71741	40151	22362	79727	13170	31431
11_rosids	77197	69092	95858	41455	54024	25960	90845
12_malvids	68818	57219	69122	**1452**	13245	29992	8999
13_Brassicales	97137	99234	53067	22273	88899	82821	36253
14_Brassicaceae	63364	99595	92168	99048	97716	99995	99988
15_Camelineae	97687	77137	99950	98110	95371	99998	99855
16_Arabidopsis	95657	89963	95790	92154	96699	99425	98990
17_A.thaliana	99808	98839	99791	99274	95785	99742	99986

*$p<10^{-5}$

Table S2. The comparison of the PAI distribution of genes in the gene networks of *A. thaliana* stress response with the corresponding distribution of the complete set of *A. thaliana* genes according to the results of the permutation test. First line: types of stress. Second line: the proportion of random samples for which the average PAI_{rand} value for a set of genes, the same size as the stress network, exceeds the PAI_{stress} value for the corresponding stress network. Third row: fraction of random samples of genes in which the value of the quadratic deviation $ChiSq_{rand}$ distribution of ages the distribution for all genes is higher than in the corresponding gene networks ($ChiSq_{stress}$). The fifth and subsequent lines: the fraction of random samples of genes in which the number Ng_{rand} of genes of the corresponding phylostratum exceeds the number Ng_{stress} (the same phylostratum, taken from network). All values in the cells must be multiplied by 10^{-5}. The values with $p<0.05$ are bold and underlined; the values with $p>0.95$ are underlined. PAI is calculated at the level of similarity of the sequences of ID=0.7.

stress	cold	heat	light	osmotic	oxidative	salt	water
$p(PAI_{stress}<PAI_{rand})$	100000	100000	98000	99999	100000	100000	100000
$p(ChiSq_{stress}<ChiSq_{rand})$	**128**	**0***	**6**	**15**	**15**	**0***	**0***
$p(Ng_{stress}<Ng_{rand})$ for specific phylostratum							
00_Cellular Organisms	**2402**	**1817**	15755	**201**	**402**	32742	78546
01_Eukaryota	**266**	**0***	7165	**40**	**983**	**0***	**0***
02_Viridiplantae	11414	**179**	33139	61574	**3895**	13032	24915
04_Embryophyta	6658	46739	**2307**	82832	**229**	14991	**483**
05_Tracheophyta	40530	23601	**51**	11696	45746	**52**	12297
07_Magnoliophyta	17302	**4322**	**1542**	15336	56599	75894	15184
08_eudicotyledons	39648	41449	10404	92622	**821**	60611	84851
10_Pentapetalae	**1143**	29632	**2846**	37337	**1956**	30749	18990
11_rosids	7611	55235	52327	63862	85947	87331	65249
12_malvids	14913	62038	74049	66203	76620	17744	13169
13_Brassicales	94785	95288	79677	**2525**	63724	10530	42477
14_Brassicaceae	69311	99794	32578	51116	88415	95994	86623
15_Camelineae	81494	87892	99391	99178	78710	99952	99412
16_Arabidopsis	99027	95289	99874	99552	99333	99996	99927
17_A.thaliana	99999	98713	99998	99987	97809	99999	100000

*$p<10^{-5}$

Table S3. Quantile values for DI distributions for *A.thaliana* and stress genes. The quantile values for stress gene sets lower than for *A.thaliana* gene set are shown in bold.

Gene set/quantile	10	25	50	75	90	100
A.thaliana	0	0.1018	0.2014	0.3562	0.5639	5.0118
All stresses nr	0.0433	**0.0948**	**0.1639**	**0.2602**	**0.4022**	**1.8019**
Cold	0.0639	0.1122	**0.174**	**0.279**	**0.416**	**1.8019**
Heat	0.0422	**0.0829**	**0.1672**	**0.2754**	**0.3975**	**0.798**
Light	0.0435	**0.0848**	**0.1531**	**0.2591**	**0.4094**	**0.7462**
Osmotic	0.0557	0.1038	**0.1651**	**0.2297**	**0.3266**	**0.6255**
Oxidative	0.0462	**0.0997**	**0.1668**	**0.309**	**0.4725**	**0.8395**
Salt	0.05	**0.0948**	**0.1551**	**0.2265**	**0.3402**	**0.7283**
Water	0.0539	**0.0997**	**0.164**	**0.2441**	**0.36**	**1.0176**

Table S4. The comparison of the PAI distribution of genes in the gene networks of *A. thaliana* stress response with the corresponding distribution of the set of *A. thaliana* genes with GO annotations only according to the results of the permutation test. First line: types of stress. Second line: the proportion of random samples for which the average PAI_{rand} value for a set of genes, the same size as the stress network, exceeds the PAI_{stress} value for the corresponding stress network. Third row: fraction of random samples of genes in which the value of the quadratic deviation $ChiSq_{rand}$ distribution of ages the distribution for all genes is higher than in the corresponding gene networks ($ChiSq_{stress}$). The fifth and subsequent lines: the fraction of random samples of genes in which the difference between the proportions of genes of *i*-th phylostratum $dfPAI_i$ among stress genes exceeds the corresponding proportion among random sample formed from the whole gene set. All values in the cells must be multiplied by 10^{-5}. The values with $p<0.05$ are bold and underlined; the values with $p>0.95$ are underlined. PAI is calculated at the level of similarity of the sequences of ID=0.5.

Stress	Cold	Heat	Llight	Osmotic	Oxidative	Salt	Water	All stress
$p(PAI_{stress}<PAI_{rand})$	100000	100000	100000	100000	100000	100000	100000	100000
$p(ChiSq_{stress}<ChiSq_{rand})$	**383**	**331**	**800**	**4493**	**65**	**1**	**5**	**0***
$p(dfPAI_{i\ stress} < dfPAI_{i\ rand})$ for specific phylostratum								
00_Cellular Organisms	**52**	**0***	**27**	**1611**	**0***	**0***	**294**	**0***
01_Eukaryota	14473	39934	39772	**2842**	79931	**403**	**78**	**68**
02_Viridiplantae	16852	12233	7182	33775	19632	84540	**102**	3491
04_Embryophyta	50791	**4378**	14719	53768	13271	89596	29436	32900
05_Tracheophyta	**2519**	67453	34339	54486	99491	25153	42304	59527
07_Magnoliophyta	42072	97829	29295	26648	52702	50569	84905	75369
08_eudicotyledons	95404	64025	79964	9366	62625	71922	82719	97286
10_Pentapetalae	68730	71710	67429	30664	51379	50032	59369	93257
11_rosids	91798	79025	90986	84048	80939	99017	71540	99966
12_malvids	14002	37435	47453	40978	15228	65209	62179	74895
13_Brassicales	89041	94694	60951	88962	96918	97851	98654	99999
14_Brassicaceae	99833	86333	99795	99977	99561	99996	100000	100000
15_Camelineae	85947	70032	96709	75897	48976	99641	99376	99985
16_Arabidopsis	69073	56559	68163	60183	35051	84847	81810	99255
17_A.thaliana	98228	94191	98080	95937	92420	98847	99726	100000

*$p<10^{-5}$

Table S5. Comparison of the divergence index (DI) distribution of genes in the gene networks of *A. thaliana* stress response with the corresponding distribution of the set of *A. thaliana* genes with GO annotations only according to the results of the permutation test. First line: types of stress. Second line: the proportion of random samples for which the average DI_{rand} value for a set of genes, the same size as the stress network, exceeds the DI_{stress} value for the corresponding stress network. Third line: fraction of random samples of genes in which the value of the quadratic deviation $ChiSq_{rand}$ distribution of DI from such distribution for all genes is higher than in the corresponding gene networks ($ChiSq_{stress}$). The fifth and subsequent lines: fraction of random samples of genes in which the difference between the proportion of genes of *i*-th phylostratum $dfDI_i$ among stress genes exceeds the corresponding proportion among random sample formed from the whole gene set. All values in the cells must be multiplied by 10^{-5}. Values with $p<0.05$ are bold and underlined; values with $p>0.95$ are underlined.

Stress	cold	heat	light	osmotic	oxidative	salt	water	All stresses nr
$p(DI_{stress}<DI_{rand})$	97074	99787	99996	99993	98604	100000	100000	100000
$p(ChiSq_{stress}<ChiSq_{rand})$	12845	48023	**4856**	**981**	56693	**12**	**154**	**0***
$p(N_{DI\ stress}<N_{DI\ rand})$ for specific DI bin								
[0,0.1]	88469	19711	**3203**	42161	26898	**4897**	24835	**4861**
(0.1,0.2]	**132**	**3921**	**806**	**61**	**4646**	**1**	**10**	**0***
(0.2,0.3]	19309	42975	71119	**4174**	69421	38369	13149	25209
(0.3,0.4]	51357	41815	91179	96457	70437	97499	96664	99469
(0.4,0.5]	69825	74331	55547	91495	28092	96485	93985	98761
(0.5,0.6]	68516	72536	67177	91395	85348	99532	99787	99852
(0.6,0.7]	94787	84728	99028	88730	21792	93593	81924	99935
(0.7,0.8]	75239	57231	49665	88216	54304	92786	75816	97855
(0.8,0.9]	46403	67365	78796	71542	49134	91682	90210	99506
(0.9,1]	62165	49801	61444	54063	63996	78471	76039	99687
(1,+∞)	63107	78326	88040	81972	89724	96673	82373	99969

*$p<10^{-5}$

Table S6. The characteristics of the gene clusters in the stress gene networks (deg_{int}, the average edge number per node for within cluster edges; deg_{ext}, the average edge number per node for edges connecting cluster and non-cluster nodes; k_{net}, the average degree of the node for all network.

Stress type	Cluster	deg_{int}	deg_{ext}
Cold	1	5.04	0.29
	2	4.92	0.15
	3	4.20	0.60
	4	4.06	0.31
	5	2.67	0.33
	k_{net}	1.83	
Heat	1	4.77	0.02
	2	2.83	0.17
	3	12.00	0
	k_{net}	2.88	
Light	1	6.30	0.26
	2	1.75	0.25
	3	3.17	0.50
	4	4.00	0.27
	5	4.00	0.40
	6	2.80	0
	k_{net}	2.05	
Osmotic	1	1.33	0.17
	2	0.75	0
	3	4.41	0.03
	k_{net}	2.90	
Oxidative	1	3.00	0.17
	2	4.00	0.33
	3	5.50	0.10
	4	2.60	0.13
	5	3.71	0
	6	3.20	0
	k_{net}	1.56	
Salt	1	7.30	0.03
	2	4.21	0.14
	3	3.00	0.25
	4	2.00	0
	5	2.67	0
	k_{net}	1.92	
Water	1	4.52	0.02
	2	3.20	0.20
	3	5.63	0.13
	4	4.00	0
	k_{net}	1.88	

Table S7. Gene ontology terms associated with specific gene clusters in the heat stress associated gene network obtained using DAVID server. The table includes the following information: the cluster number (Cluster); the GO ID and the name of term separated by ~ (Term); the number of genes associated with term (Count); - the proportion (in %) of genes associated with this GO in the cluster (%);the threshold of EASE Score returned by DAVID (PValue); list of gene IDs associated with the GO term (Genes); the number of genes in cluster (List Total); how many have the function name in gene list of interest (Pop Hits); how many genes in the GO dictionary (on the top of table) has that function name in the background genome (Pop Total); the magnitude of enrichment (Fold Enrichment); the Bonferroni correction's of the enrichment's P-values (Bonferroni); the Benjamini correction's of the enrichment's P-values (Benjamini); false discovery rate, the percentages of test which might be false positive (FDR). P-values equal or smaller than 0.05 considered strongly enriched in the annotation categories.

Cluster	Term	Count	%	PValue	Genes	List Total	Pop Hits	Pop Total	Fold Enrichment	Bonferroni	Benjamini	FDR
Biological process												
1	GO:0034620~cellular response to unfolded protein	2	8.33	2.49E-03	AT2G26150, AT5G62390	24	2	18499	770.7917	1.64E-01	2.21E-02	2.56E+00
1	GO:0034605~cellular response to heat	3	12.50	4.72E-04	AT2G26150, AT5G62390, AT3G08970	24	26	18499	88.9375	3.34E-02	4.84E-03	4.90E-01
1	GO:0070370~cellular heat acclimation	3	12.50	6.61E-05	AT4G15802, AT3G25230, AT5G48570	24	10	18499	231.2375	4.75E-03	7.93E-04	6.88E-02
1	GO:0009644~response to high light intensity	4	16.67	4.22E-05	AT2G26150, AT4G27670, AT1G74310, AT4G12400	24	55	18499	56.05758	3.04E-03	6.08E-04	4.39E-02
1	GO:0042542~response to hydrogen peroxide	5	20.83	5.51E-07	AT2G26150, AT4G27670, AT1G74310, AT5G05410, AT4G12400	24	54	18499	71.3696	3.97E-05	9.92E-06	5.73E-04
1	GO:0006457~protein folding	10	41.67	3.20E-11	AT5G42020, AT5G56010, AT4G26780, AT5G53400, AT3G17880, AT5G62390, AT1G09080, AT3G08970, AT3G25230, AT5G02500	24	288	18499	26.7636	2.31E-09	7.69E-10	3.33E-08
1	GO:0010286~heat acclimation	7	29.17	4.89E-12	AT2G26150, AT4G26780, AT5G53400, AT3G17880, AT4G21320, AT5G05410, AT4G12400	24	38	18499	141.9879	3.52E-10	1.76E-10	5.09E-09
1	GO:0009408~response to heat	21	87.50	2.78E-39	AT5G56010, AT4G11260, AT4G27670, AT3G25230, AT5G63870, AT4G12400, AT2G26150, AT4G04950, AT5G42020, AT3G09350, AT4G26780, AT5G53400, AT4G21320, AT1G09080, AT1G74310, AT3G08970, AT4G15802, AT5G16820, AT3G10800, AT5G05410, AT5G02500	24	160	18499	101.1664	2.00E-37	2.00E-37	2.89E-36
2	GO:0009737~response to abscisic acid	4	20.00	7.21E-03	AT5G57050, AT1G66340, AT4G26080, AT2G42540	20	394	18499	9.390355	6.12E-01	4.87E-02	8.09E+00
2	GO:0043044~ATP-dependent chromatin remodeling	2	10.00	5.13E-03	AT3G06400, AT2G46020	20	5	18499	369.98	4.90E-01	3.67E-02	5.82E+00
2	GO:0009871~jasmonic acid and ethylene-dependent systemic resistance, ethylene mediated signaling pathway	2	10.00	4.10E-03	AT1G66340, AT5G03280	20	4	18499	462.475	4.16E-01	3.12E-02	4.68E+00
2	GO:0016569~covalent chromatin modification	3	15.00	3.63E-03	AT3G06400, AT3G06010, AT2G46020	20	88	18499	31.53239	3.79E-01	2.93E-02	4.15E+00
2	GO:0042742~defense response to bacterium	4	20.00	2.67E-03	AT1G66340, AT1G16540, AT2G38470, AT5G03280	20	276	18499	13.40507	2.95E-01	2.31E-02	3.07E+00
2	GO:0008219~cell death	3	15.00	9.21E-04	AT5G03280, AT1G64280, AT3G16770	20	44	18499	63.06477	1.14E-01	9.25E-03	1.07E+00
2	GO:0010119~regulation of stomatal movement	3	15.00	9.64E-04	AT1G66340, AT4G26080, AT5G03280	20	45	18499	61.66333	1.19E-01	8.98E-03	1.12E+00
2	GO:0009873~ethylene-activated signaling pathway	4	20.00	7.70E-04	AT1G66340, AT1G54490, AT5G03280, AT3G16770	20	179	18499	20.66927	9.60E-02	8.38E-03	8.94E-01
2	GO:0010286~heat acclimation	3	15.00	6.87E-04	AT1G54490, AT2G42540, AT3G16770	20	38	18499	73.02237	8.61E-02	8.15E-03	7.99E-01
2	GO:0001944~vasculature development	3	15.00	3.20E-04	AT1G66340, AT1G54490, AT5G03280	20	26	18499	106.725	4.11E-02	4.18E-03	3.73E-01
2	GO:0009723~response to ethylene	4	20.00	2.63E-04	AT1G66340, AT1G54490, AT5G03280, AT3G16770	20	124	18499	29.8371	3.39E-02	3.83E-03	3.07E-01
2	GO:0009688~abscisic acid biosynthetic process	3	15.00	1.04E-04	AT1G16540, AT5G67030, AT1G52340	20	15	18499	184.99	1.35E-02	1.70E-03	1.21E-01
2	GO:0070370~cellular heat acclimation	3	15.00	4.48E-05	AT2G30250, AT2G38470, AT1G54490	20	10	18499	277.485	5.85E-03	8.37E-04	5.22E-02
2	GO:0009409~response to cold	6	30.00	1.03E-05	AT5G59820, AT1G16540, AT2G30250, AT4G26080, AT2G38470, AT2G42540	20	299	18499	18.56087	1.35E-03	2.25E-04	1.20E-02
2	GO:0009651~response to salt stress	7	35.00	6.31E-06	AT1G66340, AT1G16540, AT2G30250, AT3G06010, AT2G38470, AT5G03280, AT2G42540	20	484	18499	13.37738	8.27E-04	2.07E-04	7.36E-03
2	GO:0009414~response to water deprivation	6	30.00	7.36E-06	AT5G57050, AT5G67030, AT3G06010, AT2G38470, AT2G42540, AT1G52340	20	279	18499	19.8914	9.64E-04	1.93E-04	8.58E-03
2	GO:0010182~sugar mediated signaling pathway	5	25.00	2.14E-08	AT1G66340, AT1G16540, AT5G67030, AT5G03280, AT1G52340	20	30	18499	154.1583	2.80E-06	9.35E-07	2.50E-05
2	GO:0006970~response to osmotic stress	7	35.00	1.84E-09	AT5G57050, AT1G16540, AT2G30250, AT5G67030, AT2G38470, AT5G03280, AT2G42540	20	122	18499	53.0709	2.41E-07	1.20E-07	2.14E-06
2	GO:0009408~response to heat	14	70.00	2.39E-23	AT1G16540, AT5G59820, AT5G47910, AT3G06010, AT5G03280, AT1G64280, AT5G57050, AT1G66340, AT1G79440, AT5G67030, AT1G08550, AT4G26080, AT2G38470, AT1G52340	20	160	18499	80.93313	3.13E-21	3.13E-21	2.78E-20

3	GO:0000002~mitochondrial genome maintenance	2	33.33	8.11E-04	AT3G10140, AT3G24320	6	3	18499	2055.444	3.66E-02	1.85E-02	7.64E-01
3	GO:0006281~DNA repair	3	50.00	1.07E-03	AT1G03190, AT3G10140, AT3G28030	6	194	18499	47.67784	4.81E-02	1.63E-02	1.01E+00
3	GO:0009408~response to heat	5	83.33	2.68E-08	AT1G03190, AT3G10140, AT4G26840, AT3G24320, AT3G28030	6	160	18499	96.34896	1.23E-06	1.23E-06	2.53E-05
4	GO:0048528~post-embryonic root development	2	15.38	6.47E-03	AT1G64520, AT4G38630	13	10	18499	284.6	2.77E-01	2.91E-02	6.06E+00
4	GO:0009408~response to heat	3	23.08	4.64E-03	AT4G29040, AT1G64520, AT4G38630	13	160	18499	26.68125	2.07E-01	2.30E-02	4.38E+00
4	GO:0042176~regulation of protein catabolic process	2	15.38	3.89E-03	AT1G20200, AT1G75990	13	6	18499	474.3333	1.77E-01	2.14E-02	3.68E+00
4	GO:0080129~proteasome core complex assembly	2	15.38	1.30E-03	AT4G29040, AT4G38630	13	2	18499	1423	6.28E-02	8.08E-03	1.24E+00
4	GO:0043248~proteasome assembly	3	23.08	5.22E-05	AT4G29040, AT1G64520, AT4G38630	13	17	18499	251.1176	2.61E-03	3.73E-04	5.02E-02
4	GO:0051788~response to misfolded protein	3	23.08	1.16E-06	AT4G29040, AT1G64520, AT4G38630	13	3	18499	1423	5.78E-05	9.64E-06	1.11E-03
4	GO:0043161~proteasome-mediated ubiquitin-dependent protein catabolic process	6	46.15	3.57E-07	AT4G29040, AT4G24820, AT1G64520, AT5G09900, AT4G38630, AT5G64760	13	256	18499	33.35156	1.79E-05	3.57E-06	3.44E-04
4	GO:0030433~ER-associated ubiquitin-dependent protein catabolic process	6	46.15	5.17E-12	AT5G20000, AT4G29040, AT1G53750, AT1G45000, AT5G19990, AT3G05530	13	29	18499	294.4138	2.59E-10	6.47E-11	4.98E-09
4	GO:0045899~positive regulation of RNA polymerase II transcriptional preinitiation complex assembly	6	46.15	1.10E-14	AT5G20000, AT4G29040, AT1G53750, AT1G45000, AT5G19990, AT3G05530	13	10	18499	853.8	5.50E-13	1.83E-13	1.06E-11
4	GO:0006511~ubiquitin-dependent protein catabolic process	11	84.62	7.84E-18	AT4G29040, AT1G53750, AT4G24820, AT5G19990, AT1G64520, AT5G09900, AT1G20200, AT4G38630, AT1G75990, AT5G64760, AT3G05530	13	242	18499	64.68182	3.92E-16	1.96E-16	7.55E-15
4	GO:0030163~protein catabolic process	10	76.92	1.70E-18	AT5G20000, AT4G29040, AT1G53750, AT4G24820, AT1G45000, AT5G19990, AT1G64520, AT4G38630, AT1G75990, AT5G64760	13	112	18499	127.0536	8.52E-17	8.52E-17	1.64E-15
**Cellular component**												
1	GO:0005788~endoplasmic reticulum lumen	3	12.50	6.11E-04	AT5G42020, AT1G09080, AT3G08970	24	40	25147	78.58438	1.88E-02	6.30E-03	5.24E-01
1	GO:0005829~cytosol	10	41.67	1.13E-04	AT5G42020, AT5G56010, AT4G04950, AT4G11260, AT5G53400, AT5G62390, AT1G74310, AT4G15802, AT3G25230, AT5G02500	24	2309	25147	4.537859	3.50E-03	1.75E-03	9.71E-02
1	GO:0005737~cytoplasm	14	58.33	2.85E-05	AT2G26150, AT5G56010, AT3G09350, AT4G04950, AT4G11260, AT5G53400, AT3G17880, AT1G74310, AT5G16820, AT3G25230, AT5G63870, AT3G10800, AT5G02500, AT4G12400	24	4407	25147	3.328587	8.82E-04	8.82E-04	2.44E-02
4	GO:0005886~plasma membrane	6	46.15	2.21E-02	AT1G53750, AT4G24820, AT1G45000, AT5G19990, AT1G20200, AT1G75990	13	3702	25147	3.135145	3.01E-01	3.52E-02	1.47E+01
4	GO:0016020~membrane	5	38.46	8.08E-03	AT4G29040, AT4G24820, AT1G45000, AT4G38630, AT1G75990	13	1797	25147	5.382261	1.22E-01	1.43E-02	5.60E+00
4	GO:0005737~cytoplasm	9	69.23	2.23E-04	AT5G20000, AT4G29040, AT1G53750, AT1G45000, AT5G19990, AT5G09900, AT4G38630, AT5G64760, AT3G05530	13	4407	25147	3.950411	3.57E-03	4.47E-04	1.59E-01
4	GO:0005634~nucleus	13	100.00	1.22E-05	AT5G20000, AT4G29040, AT1G53750, AT4G24820, AT1G45000, AT5G19990, AT1G64520, AT5G09900, AT1G20200, AT4G38630, AT1G75990, AT5G64760, AT3G05530	13	9796	25147	2.567068	1.95E-04	2.78E-05	8.64E-03
4	GO:0005829~cytosol	12	92.31	4.21E-11	AT5G20000, AT4G29040, AT1G53750, AT4G24820, AT1G45000, AT5G19990, AT1G64520, AT5G09900, AT1G20200, AT4G38630, AT1G75990, AT3G05530	13	2309	25147	10.0531	6.73E-10	1.12E-10	2.99E-08
4	GO:0008541~proteasome regulatory particle, lid subcomplex	6	46.15	4.12E-14	AT4G24820, AT1G64520, AT5G09900, AT1G20200, AT1G75990, AT5G64760	13	16	25147	725.3942	6.59E-13	1.32E-13	2.93E-11
4	GO:0031597~cytosolic proteasome complex	6	46.15	4.36E-15	AT5G20000, AT4G29040, AT1G53750, AT1G45000, AT5G19990, AT3G05530	13	11	25147	1055.119	6.93E-14	1.73E-14	3.08E-12
4	GO:0008540~proteasome regulatory particle, base subcomplex	7	53.85	4.87E-17	AT5G20000, AT4G29040, AT1G53750, AT1G45000, AT5G19990, AT4G38630, AT3G05530	13	18	25147	752.2607	7.80E-16	2.60E-16	3.46E-14
4	GO:0031595~nuclear proteasome complex	8	61.54	4.97E-22	AT5G20000, AT4G29040, AT1G53750, AT1G45000, AT5G19990, AT5G09900, AT5G64760, AT3G05530	13	12	25147	1289.59	7.95E-21	3.98E-21	3.53E-19
4	GO:0000502~proteasome complex	13	100.00	2.47E-32	AT5G20000, AT4G29040, AT1G53750, AT4G24820, AT1G45000, AT5G19990, AT1G64520, AT5G09900, AT1G20200, AT4G38630, AT1G75990, AT5G64760, AT3G05530	13	64	25147	392.9219	3.95E-31	3.95E-31	1.75E-29
**Molecular function**												
1	GO:0051082~unfolded protein binding	3	12.50	4.92E-03	AT5G56010, AT4G26780, AT5G53400	23	87	18171	27.24288	1.50E-01	5.28E-02	4.21E+00
1	GO:0030544~Hsp70 protein binding	2	8.33	2.42E-03	AT3G09350, AT3G17880	23	2	18171	790.0435	7.68E-02	3.92E-02	2.09E+00
1	GO:0005515~protein binding	10	41.67	2.06E-04	AT2G26150, AT5G56010, AT4G04950, AT4G11260, AT3G17880, AT5G62390, AT1G74310, AT3G10800, AT5G05410, AT5G02500	23	1901	18171	4.155936	6.77E-03	6.77E-03	1.80E-01
2	GO:0005515~protein binding	11	55.00	5.82E-06	AT5G57050, AT1G66340, AT3G06400, AT5G47910, AT2G30250, AT4G26080, AT2G38470, AT1G54490, AT1G64280, AT2G46020, AT3G16770	20	1901	18171	5.257259	3.03E-04	3.03E-04	5.66E-03
4	GO:0030234~enzyme regulator activity	2	15.38	6.42E-03	AT1G20200, AT1G75990	10	13	18171	279.5538	6.24E-02	1.07E-02	3.79E+00

4	GO:0005524~ATP binding	6	46.15	3.01E-03	AT5G20000, AT4G29040, AT1G53750, AT1G45000, AT5G19990, AT3G05530	10	2372	18171	4.596374	2.97E-02	6.02E-03	1.80E+00
4	GO:0016787~hydrolase activity	5	38.46	5.77E-05	AT5G20000, AT4G29040, AT1G53750, AT1G45000, AT5G19990	10	487	18171	18.65606	5.77E-04	1.44E-04	3.46E-02
4	GO:0016887~ATPase activity	6	46.15	4.75E-08	AT5G20000, AT4G29040, AT1G53750, AT1G45000, AT5G19990, AT3G05530	10	241	18171	45.239	4.75E-07	1.58E-07	2.85E-05
4	GO:0017025~TBP-class protein binding	6	46.15	6.14E-13	AT5G20000, AT4G29040, AT1G53750, AT1G45000, AT5G19990, AT3G05530	10	27	18171	403.8	6.14E-12	3.07E-12	3.69E-10
4	GO:0036402~proteasome-activating ATPase activity	6	46.15	1.92E-15	AT5G20000, AT4G29040, AT1G53750, AT1G45000, AT5G19990, AT3G05530	10	10	18171	1090.26	1.89E-14	1.89E-14	1.13E-12
1	GO:0051082~unfolded protein binding	3	12.50	4.92E-03	AT5G56010, AT4G26780, AT5G53400	23	87	18171	27.24288	1.50E-01	5.28E-02	4.21E+00
1	GO:0030544~Hsp70 protein binding	2	8.33	2.42E-03	AT3G09350, AT3G17880	23	2	18171	790.0435	7.68E-02	3.92E-02	2.09E+00
1	GO:0005515~protein binding	10	41.67	2.06E-04	AT2G26150, AT5G56010, AT4G04950, AT4G11260, AT3G17880, AT5G62390, AT1G74310, AT3G10800, AT5G05410, AT5G02500	23	1901	18171	4.155936	6.77E-03	6.77E-03	1.80E-01
2	GO:0005515~protein binding	11	55.00	5.82E-06	AT5G57050, AT1G66340, AT3G06400, AT5G47910, AT2G30250, AT4G26080, AT2G38470, AT1G54490, AT1G64280, AT2G46020, AT3G16770	20	1901	18171	5.257259	3.03E-04	3.03E-04	5.66E-03
4	GO:0030234~enzyme regulator activity	2	15.38	6.42E-03	AT1G20200, AT1G75990	10	13	18171	279.5538	6.24E-02	1.07E-02	3.79E+00
4	GO:0005524~ATP binding	6	46.15	3.01E-03	AT5G20000, AT4G29040, AT1G53750, AT1G45000, AT5G19990, AT3G05530	10	2372	18171	4.596374	2.97E-02	6.02E-03	1.80E+00
4	GO:0016787~hydrolase activity	5	38.46	5.77E-05	AT5G20000, AT4G29040, AT1G53750, AT1G45000, AT5G19990	10	487	18171	18.65606	5.77E-04	1.44E-04	3.46E-02
4	GO:0016887~ATPase activity	6	46.15	4.75E-08	AT5G20000, AT4G29040, AT1G53750, AT1G45000, AT5G19990, AT3G05530	10	241	18171	45.239	4.75E-07	1.58E-07	2.85E-05
4	GO:0017025~TBP-class protein binding	6	46.15	6.14E-13	AT5G20000, AT4G29040, AT1G53750, AT1G45000, AT5G19990, AT3G05530	10	27	18171	403.8	6.14E-12	3.07E-12	3.69E-10
4	GO:0036402~proteasome-activating ATPase activity	6	46.15	1.92E-15	AT5G20000, AT4G29040, AT1G53750, AT1G45000, AT5G19990, AT3G05530	10	10	18171	1090.26	1.89E-14	1.89E-14	1.13E-12

Table S8. Gene ontology terms associated with specific gene clusters in the cold stress associated gene network obtained using DAVID server. The description of the columns as in Table S7.

Cluster	Term	Count	%	PValue	Genes	List Total	Pop Hits	Pop Total	Fold Enrichment	Bonferroni	Benjamini	FDR
Biological process												
1	GO:0048509~regulation of meristem development	2	9.52	9.69E-03	AT1G27320, AT5G35750	21	9	18499	195.7566	5.59E-01	4.70E-02	9.92E+00
1	GO:1902456~regulation of stomatal opening	2	9.52	8.62E-03	AT5G57050, AT4G33950	21	8	18499	220.2262	5.17E-01	4.44E-02	8.87E+00
1	GO:0048831~regulation of shoot system development	2	9.52	8.62E-03	AT1G27320, AT5G35750	21	8	18499	220.2262	5.17E-01	4.44E-02	8.87E+00
1	GO:2001295~malonyl-CoA biosynthetic process	2	9.52	5.39E-03	AT1G36160, AT1G36180	21	5	18499	352.3619	3.65E-01	3.19E-02	5.64E+00
1	GO:0006470~protein dephosphorylation	3	14.29	5.46E-03	AT5G57050, AT1G72770, AT4G26080	21	103	18499	25.65742	3.69E-01	3.02E-02	5.71E+00
1	GO:0080117~secondary growth	2	9.52	4.32E-03	AT1G27320, AT5G35750	21	4	18499	440.4524	3.05E-01	2.76E-02	4.54E+00
1	GO:0010271~regulation of chlorophyll catabolic process	2	9.52	3.24E-03	AT1G27320, AT5G35750	21	3	18499	587.2698	2.39E-01	2.25E-02	3.42E+00
1	GO:0006096~glycolytic process	3	14.29	2.64E-03	AT3G55440, AT1G79550, AT2G36530	21	71	18499	37.22133	1.99E-01	2.00E-02	2.80E+00
1	GO:0034757~negative regulation of iron ion transport	2	9.52	2.16E-03	AT1G27320, AT5G35750	21	2	18499	880.9048	1.66E-01	1.80E-02	2.30E+00
1	GO:0009408~response to heat	4	19.05	6.50E-04	AT5G57050, AT1G16540, AT1G79550, AT4G26080	21	160	18499	22.02262	5.31E-02	6.05E-03	6.95E-01
1	GO:0009631~cold acclimation	4	19.05	2.18E-05	AT1G74960, AT5G54590, AT1G36160, AT5G66400	21	51	18499	69.09057	1.83E-03	2.29E-04	2.34E-02
1	GO:0009409~response to cold	6	28.57	1.36E-05	AT1G16540, AT5G54590, AT4G26080, AT1G27320, AT5G65940, AT2G36530	21	299	18499	17.67702	1.14E-03	1.63E-04	1.45E-02
1	GO:0010029~regulation of seed germination	4	19.05	1.04E-05	AT1G27320, AT3G50500, AT5G35750, AT5G66880	21	40	18499	88.09048	8.74E-04	1.46E-04	1.12E-02
1	GO:0009651~response to salt stress	8	38.10	4.64E-07	AT1G16540, AT3G55440, AT1G27320, AT4G33950, AT3G50500, AT5G35750, AT5G66880, AT2G36530	21	484	18499	14.56041	3.89E-05	7.79E-06	4.98E-04
1	GO:0009414~response to water deprivation	7	33.33	3.62E-07	AT5G57050, AT5G66400, AT1G27320, AT4G33950, AT3G50500, AT5G35750, AT5G66880	21	279	18499	22.10155	3.04E-05	7.60E-06	3.89E-04
1	GO:0009737~response to abscisic acid	8	38.10	1.15E-07	AT5G57050, AT5G66400, AT4G26080, AT4G33950, AT3G50500, AT5G35750, AT5G66880, AT2G36530	21	394	18499	17.88639	9.66E-06	3.22E-06	1.23E-04
1	GO:0006970~response to osmotic stress	7	33.33	2.61E-09	AT5G57050, AT1G16540, AT1G27320, AT4G33950, AT3G50500, AT5G35750, AT5G66880	21	122	18499	50.54372	2.19E-07	1.10E-07	2.80E-06
1	GO:0009738~abscisic acid-activated signaling pathway	8	38.10	8.96E-10	AT5G57050, AT4G17870, AT1G72770, AT5G46790, AT4G26080, AT4G33950, AT3G50500, AT5G66880	21	195	18499	36.13968	7.53E-08	7.53E-08	9.62E-07
2	GO:0016070~RNA metabolic process	2	15.38	5.93E-03	AT3G14080, AT1G19120	12	10	18499	308.3167	2.26E-01	2.53E-02	5.39E+00
2	GO:0006397~mRNA processing	3	23.08	4.68E-03	AT3G14080, AT1G19120, AT4G24770	12	176	18499	26.27699	1.83E-01	2.22E-02	4.27E+00
2	GO:0000290~deadenylation-dependent decapping of nuclear-transcribed mRNA	2	15.38	4.16E-03	AT3G14080, AT1G19120	12	7	18499	440.4524	1.64E-01	2.21E-02	3.80E+00
2	GO:0000244~spliceosomal tri-snRNP complex assembly	2	15.38	2.97E-03	AT4G03430, AT1G60170	12	5	18499	616.6333	1.20E-01	1.81E-02	2.73E+00
2	GO:0000398~mRNA splicing, via spliceosome	3	23.08	2.00E-03	AT1G06960, AT4G03430, AT1G60170	12	114	18499	40.56798	8.24E-02	1.42E-02	1.84E+00
2	GO:0009845~seed germination	3	23.08	9.18E-04	AT4G03430, AT4G13850, AT1G60170	12	77	18499	60.06169	3.87E-02	7.87E-03	8.51E-01
2	GO:0032508~DNA duplex unwinding	3	23.08	7.38E-05	AT4G38680, AT4G36020, AT2G17870	12	22	18499	210.2159	3.17E-03	7.93E-04	6.86E-02
2	GO:0009414~response to water deprivation	5	38.46	1.54E-05	AT3G14080, AT4G13850, AT1G19120, AT4G38680, AT4G36020	12	279	18499	27.62694	6.61E-04	2.20E-04	1.43E-02
2	GO:0009409~response to cold	9	69.23	6.71E-13	AT2G33800, AT1G56070, AT4G03430, AT4G13850, AT1G60170, AT4G38680, AT4G24770, AT4G36020, AT2G17870	12	299	18499	46.40217	2.89E-11	1.44E-11	6.25E-10
2	GO:0009631~cold acclimation	8	61.54	2.58E-16	AT3G14080, AT1G56070, AT4G13850, AT1G19120, AT4G38680, AT4G24770, AT4G36020, AT2G17870	12	51	18499	241.817	9.55E-15	9.55E-15	2.11E-13
3	GO:0009651~response to salt stress	3	60.00	3.96E-03	AT2G21660, AT1G01060, AT2G46830	5	484	18499	22.93264	2.21E-01	4.08E-02	3.94E+00
3	GO:0042752~regulation of circadian rhythm	2	40.00	5.18E-03	AT1G22770, AT1G01060	5	24	18499	308.3167	2.79E-01	4.01E-02	5.12E+00
3	GO:2000028~regulation of photoperiodism, flowering	2	40.00	4.32E-03	AT1G22770, AT2G18790	5	20	18499	369.98	2.39E-01	3.82E-02	4.29E+00
3	GO:0006355~regulation of transcription, DNA-templated	4	80.00	5.49E-03	AT1G22770, AT1G01060, AT2G18790, AT2G46830	5	2119	18499	6.984049	2.93E-01	3.78E-02	5.42E+00
3	GO:0048574~long-day photoperiodism, flowering	2	40.00	2.59E-03	AT1G01060, AT2G46830	5	12	18499	616.6333	1.51E-01	3.22E-02	2.59E+00
3	GO:0046686~response to cadmium ion	3	60.00	2.00E-03	AT2G21660, AT1G01060, AT2G46830	5	342	18499	32.45439	1.18E-01	3.10E-02	2.00E+00
3	GO:0042754~negative regulation of circadian rhythm	2	40.00	6.49E-04	AT1G01060, AT2G46830	5	3	18499	2466.533	4.00E-02	1.35E-02	6.55E-01
3	GO:0007623~circadian rhythm	4	80.00	5.57E-07	AT1G22770, AT2G21660, AT1G01060, AT2G46830	5	97	18499	152.5691	3.51E-05	1.75E-05	5.64E-04
3	GO:0009409~response to cold	5	100.00	6.69E-08	AT1G22770, AT2G21660, AT1G01060, AT2G18790, AT2G46830	5	299	18499	61.86957	4.22E-06	4.22E-06	6.77E-05

4	GO:0035066~positive regulation of histone acetylation	2	12.50	6.47E-03	AT4G16420, AT3G07740	16	8	18499	289.0469	3.00E-01	3.19E-02	6.18E+00
4	GO:0050832~defense response to fungus	4	25.00	5.70E-03	AT1G20440, AT2G22300, AT3G50970, AT2G42530	16	464	18499	9.967134	2.70E-01	3.09E-02	5.47E+00
4	GO:0010200~response to chitin	3	18.75	5.07E-03	AT5G59820, AT3G23250, AT3G49530	16	133	18499	26.07942	2.44E-01	3.06E-02	4.87E+00
4	GO:0010150~leaf senescence	3	18.75	2.46E-03	AT2G22300, AT2G42530, AT2G42540	16	92	18499	37.70177	1.27E-01	1.92E-02	2.40E+00
4	GO:0006351~transcription, DNA-templated	7	43.75	2.47E-03	AT4G16420, AT5G59820, AT4G25480, AT4G25470, AT4G25490, AT3G49530, AT3G07740	16	1886	18499	4.291258	1.27E-01	1.69E-02	2.41E+00
4	GO:0006950~response to stress	3	18.75	1.40E-03	AT1G20450, AT1G20440, AT3G50970	16	69	18499	50.26902	7.39E-02	1.27E-02	1.36E+00
4	GO:0009737~response to abscisic acid	5	31.25	2.30E-04	AT1G20450, AT1G20440, AT3G50970, AT2G42530, AT2G42540	16	394	18499	14.67243	1.26E-02	2.52E-03	2.26E-01
4	GO:0009415~response to water	3	18.75	4.03E-05	AT1G20450, AT1G20440, AT3G50970	16	12	18499	289.0469	2.21E-03	5.54E-04	3.96E-02
4	GO:0009414~response to water deprivation	7	43.75	4.98E-08	AT1G20450, AT4G25480, AT4G25490, AT1G20440, AT3G50970, AT2G42540, AT3G55990	16	279	18499	29.00829	2.74E-06	9.13E-07	4.90E-05
4	GO:0009409~response to cold	13	81.25	1.11E-19	AT1G20450, AT5G59820, AT4G25480, AT4G25470, AT2G39810, AT4G25490, AT1G20440, AT2G22300, AT3G50970, AT2G42530, AT3G07740, AT2G42540, AT3G55990	16	299	18499	50.26902	6.09E-18	3.05E-18	1.09E-16
4	GO:0009631~cold acclimation	10	62.50	2.15E-20	AT4G16420, AT1G20450, AT5G59820, AT4G25480, AT4G25470, AT4G25490, AT1G20440, AT3G50970, AT2G42530, AT2G42540	16	51	18499	226.7034	1.18E-18	1.18E-18	2.12E-17
5	GO:0009651~response to salt stress	4	66.67	1.71E-04	AT4G01370, AT4G29810, AT2G43790, AT4G08500	6	484	18499	25.48072	1.00E-02	3.36E-03	1.71E-01
5	GO:0000165~MAPK cascade	3	50.00	1.10E-04	AT4G01370, AT4G29810, AT4G08500	6	62	18499	149.1855	6.46E-03	3.23E-03	1.10E-01
5	GO:0009409~response to cold	6	100.00	1.07E-09	AT4G01370, AT4G10030, AT4G29810, AT1G74710, AT2G43790, AT4G08500	6	299	18499	61.86957	6.30E-08	6.30E-08	1.07E-06
Cellular Component												
1	GO:0008287~protein serine/threonine phosphatase complex	2	9.52	3.18E-03	AT5G57050, AT4G26080	21	4	25147	598.7381	7.06E-02	1.81E-02	2.49E+00
1	GO:0005829~cytosol	9	42.86	2.29E-04	AT3G55440, AT1G79550, AT1G36160, AT1G36180, AT5G66400, AT4G33950, AT3G50500, AT5G66880, AT2G36530	21	2309	25147	4.667512	5.25E-03	1.75E-03	1.81E-01
1	GO:0005737~cytoplasm	13	61.90	2.57E-05	AT1G16540, AT3G55440, AT4G17870, AT1G79550, AT1G36160, AT1G72770, AT1G36180, AT5G46790, AT4G26080, AT4G33950, AT3G50500, AT5G66880, AT2G36530	21	4407	25147	3.532378	5.91E-04	5.91E-04	2.04E-02
1	GO:0005886~plasma membrane	12	57.14	3.20E-05	AT3G55440, AT4G17870, AT5G54590, AT1G79550, AT1G36160, AT5G46790, AT4G26080, AT1G27320, AT3G50500, AT5G35750, AT1G16670, AT2G36530	21	3702	25147	3.881608	7.35E-04	3.68E-04	2.53E-02
4	GO:0005634~nucleus	13	81.25	1.46E-03	AT4G16420, AT1G20450, AT5G59820, AT4G25480, AT4G25470, AT3G23250, AT2G39810, AT4G25490, AT1G20440, AT2G22300, AT3G07740, AT3G49530, AT3G55990	16	9796	25147	2.085743	1.74E-02	1.74E-02	9.38E-01
Molecular Function												
1	GO:0003989~acetyl-CoA carboxylase activity	2	9.52	7.30E-03	AT1G36160, AT1G36180	20	7	18171	259.5857	2.48E-01	4.65E-02	6.44E+00
1	GO:0004722~protein serine/threonine phosphatase activity	3	14.29	1.06E-02	AT5G57050, AT1G72770, AT4G26080	20	150	18171	18.171	3.39E-01	4.49E-02	9.20E+00
1	GO:0005524~ATP binding	8	38.10	7.70E-03	AT5G54590, AT1G79550, AT1G36160, AT1G36180, AT4G33950, AT3G50500, AT1G16670, AT5G66880	20	2372	18171	3.06425	2.60E-01	4.22E-02	6.79E+00
1	GO:0004674~protein serine/threonine kinase activity	5	23.81	8.66E-03	AT5G54590, AT4G33950, AT3G50500, AT1G16670, AT5G66880	20	804	18171	5.650187	2.88E-01	4.15E-02	7.61E+00
1	GO:0004075~biotin carboxylase activity	2	9.52	5.22E-03	AT1G36160, AT1G36180	20	5	18171	363.42	1.85E-01	4.00E-02	4.65E+00
1	GO:0005034~osmosensor activity	2	9.52	4.18E-03	AT1G27320, AT5G35750	20	4	18171	454.275	1.51E-01	4.00E-02	3.73E+00
1	GO:0009884~cytokinin receptor activity	2	9.52	3.13E-03	AT1G27320, AT5G35750	20	3	18171	605.7	1.15E-01	4.00E-02	2.81E+00
1	GO:0004721~phosphoprotein phosphatase activity	3	14.29	1.77E-03	AT4G26080, AT1G27320, AT5G35750	20	60	18171	45.4275	6.67E-02	3.39E-02	1.60E+00
1	GO:0005515~protein binding	11	52.38	5.82E-06	AT5G57050, AT4G17870, AT5G54590, AT1G72770, AT5G46790, AT4G26080, AT1G27320, AT4G33950, AT3G50500, AT5G35750, AT5G66880	20	1901	18171	5.257259	2.27E-04	2.27E-04	5.29E-03
2	GO:0000166~nucleotide binding	4	30.77	2.96E-03	AT1G06960, AT3G52150, AT4G13850, AT4G24770	12	504	18171	12.01786	6.04E-02	1.03E-02	2.27E+00
2	GO:0000339~RNA cap binding	2	15.38	3.02E-03	AT3G14080, AT1G19120	12	5	18171	605.7	6.16E-02	9.04E-03	2.31E+00
2	GO:0003690~double-stranded DNA binding	3	23.08	8.55E-04	AT4G13850, AT4G38680, AT4G36020	12	73	18171	62.22945	1.78E-02	3.59E-03	6.58E-01
2	GO:0003697~single-stranded DNA binding	3	23.08	5.79E-04	AT4G13850, AT4G38680, AT4G36020	12	60	18171	75.7125	1.21E-02	3.03E-03	4.46E-01
2	GO:0003729~mRNA binding	5	38.46	4.06E-05	AT1G56070, AT4G38680, AT4G24770, AT4G36020, AT2G17870	12	351	18171	21.57051	8.52E-04	2.84E-04	3.13E-02
2	GO:0003676~nucleic acid binding	6	46.15	1.70E-05	AT3G52150, AT4G13850, AT4G38680, AT4G24770, AT4G36020, AT2G17870	12	615	18171	14.77317	3.58E-04	1.79E-04	1.32E-02

2	GO:0003723~RNA binding	8	61.54	6.73E-08	AT1G06960, AT3G52150, AT3G14080, AT4G13850, AT1G19120, AT4G24770, AT4G36020, AT2G17870	12	769	18171	15.75293	1.41E-06	1.41E-06	5.20E-05
4	GO:0005534~galactose binding	2	12.50	1.43E-03	AT2G42530, AT2G42540	14	2	18171	1297.929	2.96E-02	1.49E-02	1.10E+00
4	GO:0003677~DNA binding	7	43.75	1.72E-03	AT4G16420, AT4G25480, AT4G25470, AT3G23250, AT4G25490, AT3G49530, AT3G07740	14	2047	18171	4.438447	3.56E-02	1.20E-02	1.32E+00
4	GO:0003700~transcription factor activity, sequence-specific DNA binding	8	50.00	6.67E-05	AT4G16420, AT5G59820, AT4G25480, AT4G25470, AT3G23250, AT4G25490, AT3G49530, AT3G07740	14	1711	18171	6.068632	1.40E-03	1.40E-03	5.15E-02
5	GO:0005515~protein binding	4	66.67	9.72E-03	AT4G01370, AT4G29810, AT2G43790, AT4G08500	6	1901	18171	6.372436	1.19E-01	4.14E-02	6.26E+00
5	GO:0004707~MAP kinase activity	2	33.33	6.31E-03	AT4G01370, AT2G43790	6	23	18171	263.3478	7.90E-02	4.03E-02	4.11E+00
5	GO:0016301~kinase activity	4	66.67	1.71E-03	AT4G01370, AT4G29810, AT2G43790, AT4G08500	6	1039	18171	11.65929	2.20E-02	2.20E-02	1.13E+00

Table S9. Gene ontology terms associated with specific gene clusters in the light stress associated gene network obtained using DAVID server. The description of the columns as in Table S7.

Cluster	Term	Count	%	PValue	Genes	List Total	Pop Hits	Pop Total	Fold Enrichment	Bonferroni	Benjamini	FDR
Biological Process												
1	GO:1902347~response to strigolactone	2	5.88	1.03E-02	AT4G08920, AT1G04400	33	6	18499	186.8586	7.85E-01	4.55E-02	1.16E+01
1	GO:0031048~chromatin silencing by small RNA	2	5.88	1.03E-02	AT2G37678, AT5G02200	33	6	18499	186.8586	7.85E-01	4.55E-02	1.16E+01
1	GO:0009649~entrainment of circadian clock	2	5.88	8.62E-03	AT2G18790, AT2G40080	33	5	18499	224.2303	7.22E-01	3.93E-02	9.80E+00
1	GO:0051457~maintenance of protein location in nucleus	2	5.88	5.18E-03	AT2G37678, AT5G02200	33	3	18499	373.7172	5.36E-01	2.45E-02	6.00E+00
1	GO:0010362~negative regulation of anion channel activity by blue light	2	5.88	3.46E-03	AT3G45780, AT5G58140	33	2	18499	560.5758	4.01E-01	1.69E-02	4.04E+00
1	GO:1901371~regulation of leaf morphogenesis	2	5.88	3.46E-03	AT4G08920, AT1G04400	33	2	18499	560.5758	4.01E-01	1.69E-02	4.04E+00
1	GO:0072387~flavin adenine dinucleotide metabolic process	2	5.88	3.46E-03	AT4G08920, AT1G04400	33	2	18499	560.5758	4.01E-01	1.69E-02	4.04E+00
1	GO:0010029~regulation of seed germination	3	8.82	2.17E-03	AT2G37678, AT2G20180, AT2G18790	33	40	18499	42.04318	2.75E-01	1.10E-02	2.56E+00
1	GO:0010118~stomatal movement	3	8.82	1.14E-03	AT4G08920, AT5G58140, AT1G04400	33	29	18499	57.9906	1.56E-01	6.03E-03	1.35E+00
1	GO:0009646~response to absence of light	3	8.82	9.18E-04	AT2G37678, AT4G08920, AT1G04400	33	26	18499	64.68182	1.27E-01	5.02E-03	1.09E+00
1	GO:0045893~positive regulation of transcription, DNA-templated	5	14.71	5.12E-04	AT5G24120, AT2G37678, AT4G15090, AT5G02200, AT4G25560	33	217	18499	12.91649	7.30E-02	2.91E-03	6.08E-01
1	GO:0009908~flower development	5	14.71	3.91E-04	AT1G22770, AT1G68050, AT5G57360, AT4G34530, AT2G40080	33	202	18499	13.87564	5.62E-02	2.31E-03	4.64E-01
1	GO:0009704~de-etiolation	3	8.82	1.58E-04	AT5G61270, AT2G43010, AT1G09530	33	11	18499	152.8843	2.31E-02	9.73E-04	1.88E-01
1	GO:0000160~phosphorelay signal transduction system	4	11.76	9.25E-05	AT1G10470, AT3G45780, AT1G59940, AT5G58140	33	51	18499	43.96673	1.36E-02	5.95E-04	1.10E-01
1	GO:0009911~positive regulation of flower development	4	11.76	4.12E-05	AT1G68050, AT1G26260, AT4G34530, AT1G04400	33	39	18499	57.49495	6.08E-03	2.77E-04	4.91E-02
1	GO:0009639~response to red or far red light	4	11.76	3.23E-05	AT2G37678, AT1G09530, AT4G15090, AT4G25560	33	36	18499	62.2862	4.77E-03	2.28E-04	3.85E-02
1	GO:0017006~protein-tetrapyrrole linkage	3	8.82	2.89E-05	AT4G16250, AT2G18790, AT1G09570	33	5	18499	336.3455	4.27E-03	2.14E-04	3.44E-02
1	GO:0009584~detection of visible light	3	8.82	2.89E-05	AT4G16250, AT2G18790, AT1G09570	33	5	18499	336.3455	4.27E-03	2.14E-04	3.44E-02
1	GO:0009416~response to light stimulus	6	17.65	1.66E-05	AT5G64330, AT1G26945, AT2G37678, AT4G08920, AT5G61270, AT1G04400	33	188	18499	17.89072	2.45E-03	1.29E-04	1.97E-02
1	GO:0010617~circadian regulation of calcium ion oscillation	3	8.82	8.69E-06	AT4G08920, AT2G18790, AT1G04400	33	3	18499	560.5758	1.28E-03	7.14E-05	1.03E-02
1	GO:2000028~regulation of photoperiodism, flowering	4	11.76	5.25E-06	AT1G22770, AT2G46340, AT2G18790, AT1G04400	33	20	18499	112.1152	7.77E-04	4.57E-05	6.26E-03
1	GO:0046777~protein autophosphorylation	6	17.65	3.30E-06	AT1G10470, AT4G08920, AT3G45780, AT4G28860, AT5G58140, AT1G04400	33	135	18499	24.91448	4.89E-04	3.05E-05	3.93E-03
1	GO:0010244~response to low fluence blue light stimulus by blue low-fluence system	4	11.76	3.92E-07	AT4G08920, AT2G43010, AT2G18790, AT1G04400	33	9	18499	249.1448	5.80E-05	3.87E-06	4.67E-04
1	GO:0006351~transcription, DNA-templated	16	47.06	1.33E-07	AT1G26945, AT2G37678, AT1G26260, AT2G20180, AT1G02340, AT4G16250, AT2G43010, AT1G09530, AT5G02200, AT1G09570, AT4G34530, AT4G25560, AT1G10470, AT5G61270, AT1G59940, AT2G18790	33	1886	18499	4.75568	1.96E-05	1.40E-06	1.58E-04
1	GO:0006355~regulation of transcription, DNA-templated	17	50.00	8.25E-08	AT1G26945, AT1G26260, AT2G20180, AT4G16250, AT1G02340, AT2G43010, AT1G09530, AT4G34530, AT1G09570, AT4G25560, AT1G10470, AT5G24120, AT1G22770, AT1G68050, AT5G61270, AT1G59940, AT2G18790	33	2119	18499	4.497304	1.22E-05	9.39E-07	9.82E-05
1	GO:0042752~regulation of circadian rhythm	6	17.65	4.64E-10	AT1G22770, AT1G10470, AT5G57360, AT4G08920, AT1G59940, AT1G04400	33	24	18499	140.1439	6.86E-08	5.72E-09	5.52E-07
1	GO:0009640~photomorphogenesis	7	20.59	3.97E-10	AT4G28880, AT2G37678, AT4G08920, AT4G28860, AT2G46340, AT2G18790, AT1G09570	33	54	18499	72.66723	5.88E-08	5.34E-09	4.73E-07
1	GO:0010114~response to red light	8	23.53	6.48E-12	AT5G24120, AT1G10470, AT2G37678, AT5G57360, AT4G08920, AT1G59940, AT2G46340, AT2G40080	33	58	18499	77.32079	9.60E-10	9.60E-11	7.73E-09
1	GO:0007623~circadian rhythm	9	26.47	4.04E-12	AT1G22770, AT1G10470, AT1G68050, AT5G57360, AT4G08920, AT3G45780, AT1G59940, AT5G58140, AT1G04400	33	97	18499	52.01218	5.98E-10	6.64E-11	4.81E-09
1	GO:0010017~red or far-red light signaling pathway	7	20.59	2.82E-12	AT1G10470, AT4G16250, AT2G43010, AT1G09530, AT2G46340, AT4G15090, AT2G40080	33	25	18499	156.9612	4.17E-10	5.22E-11	3.36E-09
1	GO:0010161~red light signaling pathway	6	17.65	1.40E-12	AT2G20180, AT2G43010, AT1G59940, AT2G18790, AT1G09570, AT5G49230	33	9	18499	373.7172	2.07E-10	2.96E-11	1.67E-09
1	GO:0009638~phototropism	7	20.59	1.99E-13	AT5G64330, AT4G08920, AT3G45780, AT5G58140, AT2G18790, AT1G09570, AT1G04400	33	17	18499	230.8253	2.94E-11	4.91E-12	2.37E-10
1	GO:0018298~protein-chromophore linkage	9	26.47	6.36E-15	AT1G68050, AT5G57360, AT4G08920, AT3G45780, AT4G16250, AT5G58140, AT2G18790, AT1G09570, AT1G04400	33	45	18499	112.1152	9.37E-13	1.87E-13	7.54E-12
1	GO:0010218~response to far red light	10	29.41	7.86E-17	AT1G22770, AT5G24120, AT2G37678, AT4G08920, AT1G02340, AT2G46340,	33	49	18499	114.4032	1.64E-14	4.11E-15	1.33E-13

					AT4G15090, AT5G02200, AT2G18790, AT1G09570							
1	GO:0009785~blue light signaling pathway	9	26.47	1.34E-18	AT5G64330, AT4G28880, AT4G08920, AT3G45780, AT1G02340, AT4G28860, AT5G63870, AT1G04400, AT5G49230	33	18	18499	280.2879	1.98E-16	6.60E-17	1.59E-15
1	GO:0009585~red, far-red light phototransduction	12	35.29	2.85E-20	AT1G22770, AT4G16250, AT1G02340, AT5G61270, AT2G43010, AT1G09530, AT2G46340, AT4G15090, AT2G18790, AT1G09570, AT2G40080, AT4G25560	33	62	18499	108.4985	4.22E-18	2.11E-18	3.40E-17
1	GO:0009637~response to blue light	13	38.24	1.34E-23	AT4G28880, AT1G26260, AT4G08920, AT4G28860, AT5G02200, AT4G34530, AT5G24120, AT1G22770, AT1G68050, AT3G45780, AT5G58140, AT2G46340, AT1G04400	33	52	18499	140.1439	1.98E-21	1.98E-21	1.59E-20
2	GO:0009637~response to blue light	2	50.00	8.41E-03	AT2G36910, AT3G28860	4	52	18499	177.875	2.75E-01	2.12E-02	7.35E+00
2	GO:0009640~photomorphogenesis	2	50.00	8.73E-03	AT2G36910, AT3G28860	4	54	18499	171.287	2.83E-01	2.06E-02	7.62E+00
2	GO:0048527~lateral root development	2	50.00	7.60E-03	AT5G20730, AT3G28860	4	47	18499	196.7979	2.52E-01	2.05E-02	6.66E+00
2	GO:0009639~response to red or far red light	2	50.00	5.83E-03	AT2G36910, AT3G28860	4	36	18499	256.9306	1.99E-01	1.83E-02	5.14E+00
2	GO:0010540~basipetal auxin transport	2	50.00	5.18E-03	AT2G36910, AT3G28860	4	32	18499	289.0469	1.79E-01	1.78E-02	4.59E+00
2	GO:0009630~gravitropism	2	50.00	5.99E-03	AT5G20730, AT1G70940	4	37	18499	249.9865	2.04E-01	1.74E-02	5.28E+00
2	GO:0048443~stamen development	2	50.00	3.73E-03	AT2G36910, AT3G28860	4	23	18499	402.1522	1.32E-01	1.41E-02	3.32E+00
2	GO:0010315~auxin efflux	2	50.00	3.24E-03	AT2G36910, AT1G70940	4	20	18499	462.475	1.16E-01	1.36E-02	2.89E+00
2	GO:0008361~regulation of cell size	2	50.00	2.43E-03	AT2G36910, AT3G28860	4	15	18499	616.6333	8.83E-02	1.31E-02	2.18E+00
2	GO:0010541~acropetal auxin transport	2	50.00	2.59E-03	AT2G36910, AT3G28860	4	16	18499	578.0938	9.39E-02	1.23E-02	2.32E+00
2	GO:0009733~response to auxin	3	75.00	7.27E-04	AT2G36910, AT5G20730, AT3G28860	4	290	18499	47.84224	2.73E-02	5.51E-03	6.55E-01
2	GO:0043481~anthocyanin accumulation in tissues in response to UV light	2	50.00	8.11E-04	AT2G36910, AT3G28860	4	5	18499	1849.9	3.03E-02	5.12E-03	7.30E-01
2	GO:0055085~transmembrane transport	3	75.00	3.89E-04	AT2G36910, AT3G28860, AT1G70940	4	212	18499	65.44458	1.47E-02	3.69E-03	3.51E-01
2	GO:0009926~auxin polar transport	3	75.00	2.69E-05	AT2G36910, AT3G28860, AT1G70940	4	56	18499	247.7545	1.02E-03	3.41E-04	2.44E-02
2	GO:0009958~positive gravitropism	3	75.00	9.25E-06	AT2G36910, AT3G28860, AT1G70940	4	33	18499	420.4318	3.51E-04	1.76E-04	8.36E-03
2	GO:0009734~auxin-activated signaling pathway	4	100.00	1.07E-06	AT2G36910, AT5G20730, AT3G28860, AT1G70940	4	190	18499	97.36316	4.05E-05	4.05E-05	9.64E-04
3	GO:0072593~reactive oxygen species metabolic process	2	33.33	4.32E-03	AT4G34460, AT2G26300	6	16	18499	385.3958	1.98E-01	2.42E-02	4.10E+00
3	GO:0009094~L-phenylalanine biosynthetic process	2	33.33	2.43E-03	AT2G26300, AT1G48270	6	9	18499	685.1481	1.17E-01	1.76E-02	2.33E+00
3	GO:0007186~G-protein coupled receptor signaling pathway	2	33.33	2.70E-03	AT2G26300, AT1G48270	6	10	18499	616.6333	1.29E-01	1.71E-02	2.58E+00
3	GO:0006571~tyrosine biosynthetic process	2	33.33	1.35E-03	AT2G26300, AT1G48270	6	5	18499	1233.267	6.66E-02	1.14E-02	1.30E+00
3	GO:0009845~seed germination	3	50.00	1.70E-04	AT4G34460, AT2G26300, AT1G48270	6	77	18499	120.1234	8.61E-03	1.73E-03	1.64E-01
3	GO:0005834~heterotrimeric G-protein complex	3	50.00	7.82E-05	AT4G34460, AT1G53090, AT2G26300	6	71	25147	177.0915	1.09E-03	1.09E-03	5.31E-02
3	GO:0009788~negative regulation of abscisic acid-activated signaling pathway	3	50.00	4.31E-05	AT4G34460, AT2G26300, AT1G48270	6	39	18499	237.1667	2.20E-03	5.50E-04	4.17E-02
3	GO:0009785~blue light signaling pathway	3	50.00	8.93E-06	AT2G26300, AT3G59220, AT1G48270	6	18	18499	513.8611	4.55E-04	2.28E-04	8.64E-03
3	GO:0009738~abscisic acid-activated signaling pathway	4	66.67	1.14E-05	AT4G34460, AT2G26300, AT3G59220, AT1G48270	6	195	18499	63.24444	5.79E-04	1.93E-04	1.10E-02
3	GO:0010244~response to low fluence blue light stimulus by blue low-fluence system	3	50.00	2.10E-06	AT2G26300, AT3G59220, AT1G48270	6	9	18499	1027.722	1.07E-04	1.07E-04	2.03E-03
4	GO:0007049~cell cycle	4	40.00	2.28E-05	AT1G18040, AT5G27620, AT1G66750, AT4G30820	10	122	18499	60.65246	1.21E-03	1.21E-03	2.23E-02
5	GO:0009637~response to blue light	3	60.00	4.63E-05	AT1G42550, AT5G38150, AT1G66840	5	52	18499	213.45	1.20E-03	4.01E-04	3.80E-02
5	GO:0009904~chloroplast accumulation movement	3	60.00	6.65E-06	AT5G38150, AT1G75100, AT1G66840	5	20	18499	554.97	1.73E-04	8.65E-05	5.46E-03
5	GO:0009903~chloroplast avoidance movement	4	80.00	7.67E-09	AT1G42550, AT5G38150, AT1G75100, AT1G66840	5	24	18499	616.6333	1.99E-07	1.99E-07	6.29E-06
6	GO:0009231~riboflavin biosynthetic process	5	83.33	5.07E-13	AT2G44050, AT3G47390, AT5G64300, AT5G59750, AT2G22450	6	12	18499	1284.653	6.59E-12	6.59E-12	3.36E-10
**Cellular Component**												
1	GO:0009898~cytoplasmic side of plasma membrane	2	5.88	1.05E-02	AT3G45780, AT5G58140	34	8	25147	184.9044	2.06E-01	3.25E-02	7.89E+00
1	GO:0005622~intracellular	7	20.59	1.55E-04	AT1G10470, AT3G45780, AT4G16250, AT1G59940, AT5G58140, AT2G18790, AT1G09570	34	634	25147	8.166125	3.41E-03	5.70E-04	1.22E-01
1	GO:0005737~cytoplasm	17	50.00	4.34E-05	AT4G28880, AT1G26945, AT2G37678, AT5G57360, AT4G08920, AT2G47700, AT4G28860, AT5G02200, AT5G63870, AT1G09570, AT1G22770, AT1G10470, AT1G68050, AT3G45780, AT1G59940, AT5G58140, AT1G04400	34	4407	25147	2.853075	9.55E-04	1.91E-04	3.40E-02
1	GO:0016605~PML body	3	8.82	1.00E-05	AT4G08920, AT2G46340, AT1G04400	34	4	25147	554.7132	2.20E-04	5.50E-05	7.83E-03

1	GO:0016607~nuclear speck	6	17.65	3.23E-08	AT5G61270, AT2G46340, AT5G02200, AT2G18790, AT1G09570, AT4G25560	34	70	25147	63.3958	7.10E-07	2.37E-07	2.53E-05
1	GO:0005634~nucleus	32	94.12	4.05E-11	AT4G28880, AT1G26945, AT1G26260, AT4G08920, AT2G20180, AT4G16250, AT4G28860, AT2G43010, AT5G63870, AT1G09570, AT1G22770, AT1G68050, AT3G45780, AT1G59940, AT5G58140, AT2G46340, AT2G18790, AT1G04400, AT2G40080, AT5G49230, AT2G37678, AT5G57360, AT1G02340, AT1G09530, AT5G02200, AT4G34530, AT4G25560, AT1G10470, AT5G64330, AT5G61270, AT5G61230, AT4G15090	34	9796	25147	2.416064	8.90E-10	4.45E-10	3.17E-08
1	GO:0016604~nuclear body	6	17.65	1.73E-11	AT2G37678, AT4G08920, AT2G46340, AT2G18790, AT1G09570, AT1G04400	34	17	25147	261.0415	3.81E-10	3.81E-10	1.36E-08
5	GO:0005623~cell	3	60.00	3.44E-04	AT1G42550, AT5G38150, AT1G66840	5	192	25147	78.58438	3.10E-03	3.10E-03	1.98E-01
6	GO:0009570~chloroplast stroma	3	50.00	6.39E-03	AT1G79440, AT2G44050, AT5G64300	6	653	25147	19.25498	3.77E-02	1.91E-02	3.02E+00
6	GO:0009507~chloroplast	6	100.00	8.45E-05	AT1G79440, AT2G44050, AT3G47390, AT5G64300, AT5G59750, AT2G22450	6	3855	25147	6.523217	5.07E-04	5.07E-04	4.04E-02
Molecular Function												
1	GO:0042802~identical protein binding	3	8.82	7.11E-03	AT4G08920, AT3G45780, AT5G58140	33	72	18171	22.94318	2.59E-01	2.47E-02	6.39E+00
1	GO:0031516~far-red light photoreceptor activity	2	5.88	3.52E-03	AT2G18790, AT1G09570	33	2	18171	550.6364	1.38E-01	1.34E-02	3.21E+00
1	GO:0003700~transcription factor activity, sequence-specific DNA binding	10	29.41	2.16E-03	AT5G24120, AT1G26260, AT2G20180, AT1G02340, AT5G61270, AT2G43010, AT1G09530, AT4G15090, AT4G34530, AT4G25560	33	1711	18171	3.218214	8.68E-02	9.04E-03	1.98E+00
1	GO:0004871~signal transducer activity	5	14.71	1.34E-04	AT5G64330, AT4G16250, AT2G46340, AT2G18790, AT1G09570	33	150	18171	18.35455	5.61E-03	6.25E-04	1.24E-01
1	GO:0008020~G-protein coupled photoreceptor activity	3	8.82	1.80E-05	AT4G16250, AT2G18790, AT1G09570	33	4	18171	412.9773	7.55E-04	9.44E-05	1.66E-02
1	GO:0009883~red or far-red light photoreceptor activity	3	8.82	9.00E-06	AT4G16250, AT2G18790, AT1G09570	33	3	18171	550.6364	3.78E-04	5.40E-05	8.33E-03
1	GO:0046983~protein dimerization activity	8	23.53	1.26E-06	AT1G26945, AT1G26260, AT2G20180, AT1G02340, AT5G61270, AT2G43010, AT1G09530, AT4G34530	33	325	18171	13.55413	5.28E-05	8.81E-06	1.16E-03
1	GO:0000155~phosphorelay sensor kinase activity	5	14.71	3.01E-08	AT3G45780, AT4G16250, AT5G58140, AT2G18790, AT1G09570	33	19	18171	144.9043	1.27E-06	2.53E-07	2.79E-05
1	GO:0042803~protein homodimerization activity	8	23.53	2.19E-08	AT2G37678, AT4G08920, AT4G16250, AT5G02200, AT2G18790, AT1G09570, AT1G04400, AT2G40080	33	179	18171	24.60945	9.19E-07	2.30E-07	2.03E-05
1	GO:0009881~photoreceptor activity	5	14.71	3.88E-09	AT1G68050, AT5G57360, AT4G16250, AT2G18790, AT1G09570	33	12	18171	229.4318	1.63E-07	5.43E-08	3.59E-06
1	GO:0009882~blue light photoreceptor activity	5	14.71	3.95E-11	AT5G57360, AT4G08920, AT3G45780, AT5G58140, AT1G04400	33	5	18171	550.6364	1.66E-09	8.30E-10	3.66E-08
1	GO:0005515~protein binding	22	64.71	9.61E-14	AT4G28880, AT1G26260, AT5G57360, AT4G08920, AT2G20180, AT4G16250, AT1G02340, AT4G28860, AT2G43010, AT1G09530, AT5G02200, AT4G34530, AT1G09570, AT4G25560, AT1G22770, AT1G10470, AT5G64330, AT1G68050, AT3G45780, AT2G46340, AT2G18790, AT1G04400	33	1901	18171	6.372436	4.03E-12	4.03E-12	8.89E-11
2	GO:0010329~auxin efflux transmembrane transporter activity	3	75.00	7.90E-06	AT2G36910, AT3G28860, AT1G70940	4	30	18171	454.275	7.11E-05	7.11E-05	4.54E-03
3	GO:0004871~signal transducer activity	3	50.00	6.66E-04	AT4G34460, AT1G53090, AT2G26300	6	150	18171	60.57	9.28E-03	4.65E-03	4.52E-01
3	GO:0005515~protein binding	6	100.00	1.25E-05	AT4G34460, AT1G53090, AT1G20090, AT2G26300, AT3G59220, AT1G48270	6	1901	18171	9.558653	1.75E-04	1.75E-04	8.47E-03
4	GO:0008353~RNA polymerase II carboxy-terminal domain kinase activity	2	20.00	5.93E-03	AT1G18040, AT1G66750	10	12	18171	302.85	1.23E-01	4.27E-02	4.55E+00
4	GO:0004693~cyclin-dependent protein serine/threonine kinase activity	3	30.00	1.86E-04	AT1G18040, AT5G27620, AT1G66750	10	42	18171	129.7929	4.08E-03	2.04E-03	1.45E-01
4	GO:0005515~protein binding	8	80.00	4.04E-06	AT1G18040, AT1G03190, AT3G61600, AT5G27620, AT1G66750, AT4G14110, AT1G26830, AT4G30820	10	1901	18171	7.646923	8.88E-05	8.88E-05	3.16E-03
6	GO:0005525~GTP binding	3	50.00	2.49E-03	AT5G64300, AT5G59750, AT2G22450	6	292	18171	31.11473	4.15E-02	2.10E-02	1.79E+00
6	GO:0008686~3,4-dihydroxy-2-butanone-4-phosphate synthase activity	3	50.00	3.63E-07	AT5G64300, AT5G59750, AT2G22450	6	4	18171	2271.375	6.18E-06	6.18E-06	2.63E-04
6	GO:0003935~GTP cyclohydrolase II activity	3	50.00	3.63E-07	AT5G64300, AT5G59750, AT2G22450	6	4	18171	2271.375	6.18E-06	6.18E-06	2.63E-04

Table S10. Gene ontology terms associated with specific gene clusters in the osmotic stress associated gene network obtained using DAVID server. The description of the columns as in Table S7.

Cluster	Term	Count	%	PValue	Genes	List Total	Pop Hits	Pop Total	Fold Enrichment	Bonferroni	Benjamini	FDR
Biological Process												
1	GO:0009631~cold acclimation	3	10.34	2.69E-03	AT5G15960, AT1G20440, AT2G42540	29	51	18499	37.52333	2.17E-01	1.62E-02	2.89E+00
1	GO:0010119~regulation of stomatal movement	3	10.34	2.10E-03	AT2G40220, AT4G26080, AT4G33950	29	45	18499	42.52644	1.74E-01	1.36E-02	2.27E+00
1	GO:0009408~response to heat	4	13.79	1.77E-03	AT5G57050, AT1G16540, AT5G67030, AT4G26080	29	160	18499	15.94741	1.49E-01	1.24E-02	1.92E+00
1	GO:0010118~stomatal movement	3	10.34	8.75E-04	AT1G16540, AT4G33950, AT4G17615	29	29	18499	65.9893	7.65E-02	7.21E-03	9.49E-01
1	GO:0010182~sugar mediated signaling pathway	3	10.34	9.36E-04	AT1G16540, AT2G40220, AT5G67030	29	30	18499	63.78966	8.17E-02	7.08E-03	1.02E+00
1	GO:0009688~abscisic acid biosynthetic process	3	10.34	2.29E-04	AT1G16540, AT5G67030, AT3G14440	29	15	18499	127.5793	2.06E-02	2.08E-03	2.50E-01
1	GO:0009409~response to cold	7	24.14	4.73E-06	AT1G16540, AT5G52310, AT5G15960, AT1G20440, AT4G26080, AT4G17615, AT2G42540	29	299	18499	14.93403	4.31E-04	4.79E-05	5.16E-03
1	GO:0080163~regulation of protein serine/threonine phosphatase activity	4	13.79	2.96E-06	AT4G27920, AT4G17870, AT5G46790, AT2G26040	29	19	18499	134.294	2.69E-04	3.37E-05	3.23E-03
1	GO:0006468~protein phosphorylation	12	41.38	2.40E-08	AT5G08590, AT5G57630, AT4G40010, AT2G23030, AT5G63650, AT4G33950, AT1G60940, AT3G50500, AT1G10940, AT3G17510, AT5G66880, AT1G78290	29	870	18499	8.798573	2.19E-06	3.13E-07	2.62E-05
1	GO:0009737~response to abscisic acid	11	37.93	1.60E-10	AT5G57050, AT5G52310, AT5G15960, AT1G20440, AT4G26080, AT4G33950, AT3G50500, AT1G10940, AT3G17510, AT5G66880, AT2G42540	29	394	18499	17.80929	1.45E-08	2.42E-09	1.74E-07
1	GO:0009651~response to salt stress	14	48.28	6.01E-14	AT1G16540, AT5G08590, AT5G52310, AT4G40010, AT5G63650, AT4G33950, AT1G60940, AT3G50500, AT4G17615, AT1G10940, AT3G17510, AT5G66880, AT4G01420, AT2G42540	29	484	18499	18.45155	5.47E-12	1.09E-12	6.55E-11
1	GO:0035556~intracellular signal transduction	12	41.38	1.72E-15	AT5G08590, AT5G57630, AT4G40010, AT2G23030, AT5G63650, AT4G33950, AT1G60940, AT3G50500, AT1G10940, AT3G17510, AT5G66880, AT1G78290	29	189	18499	40.50137	1.52E-13	7.57E-14	1.82E-12
1	GO:0009738~abscisic acid-activated signaling pathway	12	41.38	2.43E-15	AT4G27920, AT5G57050, AT4G17870, AT2G40220, AT1G72770, AT5G46790, AT4G26080, AT4G33950, AT3G50500, AT2G26040, AT4G17615, AT5G66880	29	195	18499	39.25517	2.22E-13	7.41E-14	2.66E-12
1	GO:0009414~response to water deprivation	13	44.83	2.64E-15	AT5G57050, AT5G52310, AT2G40220, AT5G15960, AT5G67030, AT1G20440, AT3G14440, AT4G33950, AT3G50500, AT4G17615, AT5G66880, AT4G01420, AT2G42540	29	279	18499	29.72278	2.42E-13	6.06E-14	2.91E-12
1	GO:0006970~response to osmotic stress	22	75.86	2.96E-41	AT1G16540, AT5G08590, AT5G15960, AT1G20440, AT4G33950, AT1G60940, AT4G17615, AT1G10940, AT5G66880, AT4G01420, AT2G42540, AT5G57050, AT5G52310, AT4G40010, AT2G40220, AT5G67030, AT2G23030, AT5G63650, AT3G14440, AT3G50500, AT3G17510, AT1G78290	29	122	18499	115.0305	2.70E-39	2.70E-39	3.23E-38
2	GO:0007165~signal transduction	3	50.00	5.62E-03	AT4G01370, AT3G45640, AT2G43790	6	450	18499	20.55444	2.95E-01	3.81E-02	5.53E+00
2	GO:0010229~inflorescence development	2	33.33	4.86E-03	AT3G45640, AT2G43790	6	18	18499	342.5741	2.61E-01	3.70E-02	4.79E+00
2	GO:0010120~camalexin biosynthetic process	2	33.33	2.70E-03	AT3G45640, AT2G43790	6	10	18499	616.6333	1.54E-01	2.37E-02	2.69E+00
2	GO:2000037~regulation of stomatal complex patterning	2	33.33	1.62E-03	AT3G45640, AT2G43790	6	6	18499	1027.722	9.57E-02	1.66E-02	1.62E+00
2	GO:2000038~regulation of stomatal complex development	2	33.33	1.62E-03	AT3G45640, AT2G43790	6	6	18499	1027.722	9.57E-02	1.66E-02	1.62E+00
2	GO:0009555~pollen development	3	50.00	1.06E-03	AT4G01370, AT3G45640, AT2G43790	6	193	18499	47.92487	6.37E-02	1.31E-02	1.07E+00
2	GO:0080136~priming of cellular response to stress	2	33.33	5.41E-04	AT3G45640, AT2G43790	6	2	18499	3083.167	3.30E-02	8.35E-03	5.44E-01
2	GO:0009651~response to salt stress	4	66.67	1.71E-04	AT3G08730, AT4G01370, AT2G43790, AT4G08500	6	484	18499	25.48072	1.06E-02	3.53E-03	1.73E-01
2	GO:0009409~response to cold	5	83.33	3.30E-07	AT3G08730, AT4G01370, AT3G45640, AT2G43790, AT4G08500	6	299	18499	51.55797	2.05E-05	1.02E-05	3.33E-04
2	GO:0006970~response to osmotic stress	5	83.33	8.96E-09	AT3G08730, AT3G45640, AT1G43700, AT2G43790, AT4G08500	6	122	18499	126.3593	5.55E-07	5.55E-07	9.04E-06
Cellular Component												
1	GO:0005886~plasma membrane	10	34.48	1.22E-02	AT4G27920, AT4G17870, AT5G46790, AT4G26080, AT1G60940, AT3G50500, AT2G26040, AT4G17615, AT3G17510, AT4G01420	28	3702	25147	2.426005	1.69E-01	3.62E-02	8.20E+00
1	GO:0008287~protein serine/threonine phosphatase complex	2	6.90	4.29E-03	AT5G57050, AT4G26080	28	4	25147	449.0536	6.24E-02	1.60E-02	2.94E+00
1	GO:0005829~cytosol	9	31.03	2.25E-03	AT5G57630, AT4G40010, AT2G23030, AT1G20440, AT4G33950, AT1G60940, AT3G50500, AT1G10940, AT5G66880	28	2309	25147	3.500634	3.32E-02	1.12E-02	1.56E+00

1	GO:0005634~nucleus	22	75.86	4.60E-05	AT5G08590, AT1G20440, AT1G72770, AT4G33950, AT2G26040, AT1G60940, AT1G10940, AT4G01420, AT5G66880, AT4G27920, AT5G52310, AT4G17870, AT4G40010, AT5G57630, AT2G40220, AT2G23030, AT5G63650, AT5G46790, AT4G26080, AT3G50500, AT3G17510, AT1G78290	28	9796	25147	2.016982	6.90E-04	3.45E-04	3.20E-02
1	GO:0005737~cytoplasm	20	68.97	2.15E-09	AT1G16540, AT5G08590, AT1G72770, AT4G33950, AT2G26040, AT4G17615, AT4G01420, AT5G66880, AT4G27920, AT5G52310, AT4G17870, AT4G40010, AT5G57630, AT2G23030, AT5G63650, AT5G46790, AT4G26080, AT3G50500, AT3G17510, AT1G78290	28	4407	25147	4.075821	3.23E-08	3.23E-08	1.50E-06
Molecular Function												
1	GO:0070300~phosphatidic acid binding	2	6.90	1.23E-02	AT1G60940, AT1G10940	26	9	18171	155.3077	3.52E-01	4.25E-02	1.04E+01
1	GO:0042803~protein homodimerization activity	4	13.79	1.84E-03	AT4G27920, AT4G17870, AT5G46790, AT2G26040	26	179	18171	15.61753	6.25E-02	7.15E-03	1.62E+00
1	GO:0004672~protein kinase activity	6	20.69	4.40E-04	AT5G08590, AT4G33950, AT1G60940, AT3G50500, AT1G10940, AT5G66880	26	482	18171	8.699808	1.53E-02	1.93E-03	3.89E-01
1	GO:0005524~ATP binding	12	41.38	1.40E-04	AT5G08590, AT5G57630, AT4G40010, AT2G23030, AT5G63650, AT4G33950, AT1G60940, AT3G50500, AT1G10940, AT3G17510, AT5G66880, AT1G78290	26	2372	18171	3.535673	4.90E-03	7.01E-04	1.24E-01
1	GO:0004872~receptor activity	4	13.79	2.20E-05	AT4G27920, AT4G17870, AT5G46790, AT2G26040	26	40	18171	69.88846	7.69E-04	1.28E-04	1.95E-02
1	GO:0010427~abscisic acid binding	4	13.79	2.20E-06	AT4G27920, AT4G17870, AT5G46790, AT2G26040	26	19	18171	147.1336	7.69E-05	1.92E-05	1.95E-03
1	GO:0005515~protein binding	13	44.83	2.34E-06	AT4G27920, AT5G57050, AT4G17870, AT5G57630, AT2G40220, AT1G72770, AT5G46790, AT4G26080, AT4G33950, AT3G50500, AT2G26040, AT3G17510, AT5G66880	26	1901	18171	4.779327	8.20E-05	1.64E-05	2.08E-03
1	GO:0004864~protein phosphatase inhibitor activity	4	13.79	1.27E-06	AT4G27920, AT4G17870, AT5G46790, AT2G26040	26	16	18171	174.7212	4.46E-05	1.49E-05	1.13E-03
1	GO:0016301~kinase activity	12	41.38	4.30E-08	AT5G08590, AT5G57630, AT4G40010, AT2G23030, AT5G63650, AT4G33950, AT1G60940, AT3G50500, AT1G10940, AT3G17510, AT5G66880, AT1G78290	26	1039	18171	8.071815	1.51E-06	7.53E-07	3.81E-05
1	GO:0004674~protein serine/threonine kinase activity	12	41.38	3.00E-09	AT5G08590, AT5G57630, AT4G40010, AT2G23030, AT5G63650, AT4G33950, AT1G60940, AT3G50500, AT1G10940, AT3G17510, AT5G66880, AT1G78290	26	804	18171	10.43111	1.05E-07	1.05E-07	2.66E-06
2	GO:0005524~ATP binding	5	83.33	1.30E-03	AT3G08730, AT4G01370, AT3G45640, AT2G43790, AT4G08500	6	2372	18171	6.383853	1.80E-02	3.63E-03	8.78E-01
2	GO:0004672~protein kinase activity	4	66.67	1.78E-04	AT3G08730, AT4G01370, AT3G45640, AT2G43790	6	482	18171	25.13278	2.49E-03	6.24E-04	1.21E-01
2	GO:0016301~kinase activity	5	83.33	5.07E-05	AT3G08730, AT4G01370, AT3G45640, AT2G43790, AT4G08500	6	1039	18171	14.57411	7.10E-04	2.37E-04	3.45E-02
2	GO:0005515~protein binding	6	100.00	1.25E-05	AT3G08730, AT4G01370, AT3G45640, AT1G43700, AT2G43790, AT4G08500	6	1901	18171	9.558653	1.75E-04	1.75E-04	8.47E-03
2	GO:0004707~MAP kinase activity	3	50.00	1.53E-05	AT4G01370, AT3G45640, AT2G43790	6	23	18171	395.0217	2.14E-04	1.07E-04	1.04E-02

Table S11. Gene ontology terms associated with specific gene clusters in the oxidative stress associated gene network obtained using DAVID server. The description of the columns as in Table S7.

Cluster	Term	Count	%	PValue	Genes	List Total	Pop Hits	Pop Total	Fold Enrichment	Bonferroni	Benjamini	FDR
Biological Process												
1	GO:0009611~response to wounding	5	41.67	2.59E-06	AT5G59820, AT3G45640, AT1G27730, AT1G19180, AT1G32640	11	199	18499	42.25445	1.74E-04	5.79E-05	2.66E-03
1	GO:0010200~response to chitin	5	41.67	5.19E-07	AT3G46090, AT5G59820, AT3G45640, AT1G27730, AT1G32640	11	133	18499	63.22283	3.47E-05	1.74E-05	5.32E-04
1	GO:0006979~response to oxidative stress	9	75.00	1.49E-13	AT3G46090, AT2G40000, AT5G59820, AT3G45640, AT1G27730, AT3G22380, AT1G09970, AT3G06110, AT1G19020	11	291	18499	52.01218	1.00E-11	1.00E-11	1.53E-10
2	GO:0006379~mRNA cleavage	2	33.33	2.70E-03	AT5G23880, AT5G51660	6	10	18499	616.6333	4.49E-02	2.27E-02	1.94E+00
2	GO:0006378~mRNA polyadenylation	3	50.00	1.11E-05	AT5G23880, AT1G30460, AT5G51660	6	20	18499	462.475	1.88E-04	1.88E-04	8.02E-03
3	GO:0000302~response to reactive oxygen species	2	20.00	1.59E-02	AT4G35000, AT3G09640	10	33	18499	112.1152	4.57E-01	3.99E-02	1.35E+01
3	GO:0010193~response to ozone	2	20.00	1.59E-02	AT4G25100, AT1G08830	10	33	18499	112.1152	4.57E-01	3.99E-02	1.35E+01
3	GO:0010039~response to iron ion	2	20.00	8.73E-03	AT2G28190, AT1G08830	10	18	18499	205.5444	2.83E-01	2.35E-02	7.61E+00
3	GO:0071486~cellular response to high light intensity	2	20.00	3.89E-03	AT5G18100, AT2G28190	10	8	18499	462.475	1.38E-01	1.13E-02	3.46E+00
3	GO:0071329~cellular response to sucrose stimulus	2	20.00	3.40E-03	AT2G28190, AT1G08830	10	7	18499	528.5429	1.21E-01	1.07E-02	3.03E+00
3	GO:0006801~superoxide metabolic process	2	20.00	9.73E-04	AT2G28190, AT1G08830	10	2	18499	1849.9	3.63E-02	3.36E-03	8.76E-01
3	GO:0042744~hydrogen peroxide catabolic process	3	30.00	8.06E-04	AT4G35090, AT4G35000, AT3G09640	10	89	18499	62.35618	3.02E-02	3.06E-03	7.26E-01
3	GO:0071472~cellular response to salt stress	3	30.00	1.10E-04	AT5G18100, AT2G28190, AT1G08830	10	33	18499	168.1727	4.18E-03	4.65E-04	9.96E-02
3	GO:0046688~response to copper ion	3	30.00	4.40E-05	AT4G25100, AT2G28190, AT1G08830	10	21	18499	264.2714	1.67E-03	2.09E-04	3.97E-02
3	GO:0071493~cellular response to UV-B	3	30.00	4.41E-06	AT5G18100, AT2G28190, AT1G08830	10	7	18499	792.8143	1.68E-04	2.40E-05	3.99E-03
3	GO:0034599~cellular response to oxidative stress	4	40.00	3.27E-06	AT4G35000, AT3G09640, AT2G28190, AT1G08830	10	64	18499	115.6188	1.24E-04	2.07E-05	2.95E-03
3	GO:0071484~cellular response to light intensity	3	30.00	2.10E-06	AT5G18100, AT2G28190, AT1G08830	10	5	18499	1109.94	7.99E-05	1.60E-05	1.90E-03
3	GO:0071457~cellular response to ozone	3	30.00	6.31E-07	AT5G18100, AT2G28190, AT1G08830	10	3	18499	1849.9	2.40E-05	6.00E-06	5.70E-04
3	GO:0055114~oxidation-reduction process	8	80.00	3.10E-07	AT4G35090, AT1G63460, AT4G25100, AT4G35000, AT5G18100, AT3G09640, AT2G28190, AT1G08830	10	1330	18499	11.12722	1.18E-05	3.92E-06	2.80E-04
3	GO:0019430~removal of superoxide radicals	4	40.00	1.75E-08	AT4G25100, AT5G18100, AT2G28190, AT1G08830	10	12	18499	616.6333	6.64E-07	3.32E-07	1.58E-05
3	GO:0006979~response to oxidative stress	10	100.00	5.22E-17	AT4G35090, AT1G63460, AT4G39640, AT4G25100, AT4G35000, AT5G18100, AT3G09640, AT1G02930, AT2G28190, AT1G08830	10	291	18499	63.57045	1.98E-15	1.98E-15	4.71E-14
4	GO:0043086~negative regulation of catalytic activity	2	25.00	2.36E-02	AT1G03680, AT3G15360	8	63	18499	73.40873	4.23E-01	4.47E-02	1.72E+01
4	GO:0006464~cellular protein modification process	2	25.00	1.50E-02	AT5G07460, AT4G25130	8	40	18499	115.6188	2.94E-01	3.12E-02	1.13E+01
4	GO:0006109~regulation of carbohydrate metabolic process	2	25.00	2.27E-03	AT1G03680, AT3G15360	8	6	18499	770.7917	5.09E-02	5.21E-03	1.78E+00
4	GO:0006457~protein folding	4	50.00	1.25E-04	AT1G76080, AT4G03520, AT1G03680, AT3G15360	8	288	18499	32.11632	2.87E-03	3.19E-04	9.89E-02
4	GO:0043085~positive regulation of catalytic activity	3	37.50	1.29E-05	AT4G03520, AT1G03680, AT3G15360	8	15	18499	462.475	2.96E-04	4.22E-05	1.02E-02
4	GO:0030091~protein repair	3	37.50	1.47E-05	AT5G07460, AT4G04800, AT4G25130	8	16	18499	433.5703	3.38E-04	4.22E-05	1.16E-02
4	GO:0000103~sulfate assimilation	4	50.00	6.86E-07	AT1G76080, AT4G03520, AT1G03680, AT3G15360	8	51	18499	181.3627	1.58E-05	2.63E-06	5.43E-04
4	GO:0006662~glycerol ether metabolic process	4	50.00	2.78E-07	AT1G76080, AT4G03520, AT1G03680, AT3G15360	8	38	18499	243.4079	6.40E-06	1.28E-06	2.21E-04
4	GO:0045454~cell redox homeostasis	5	62.50	1.63E-07	AT1G76080, AT3G06050, AT4G03520, AT1G03680, AT3G15360	8	155	18499	74.59274	3.74E-06	9.36E-07	1.29E-04
4	GO:0006979~response to oxidative stress	6	75.00	1.91E-08	AT1G76080, AT5G07460, AT3G06050, AT4G03520, AT1G03680, AT3G15360	8	291	18499	47.67784	4.38E-07	1.46E-07	1.51E-05
4	GO:0055114~oxidation-reduction process	8	100.00	9.78E-09	AT1G76080, AT5G07460, AT3G06050, AT4G04800, AT4G25130, AT4G03520, AT1G03680, AT3G15360	8	1330	18499	13.90902	2.25E-07	1.13E-07	7.76E-06
4	GO:0034599~cellular response to oxidative stress	7	87.50	9.41E-15	AT1G76080, AT5G07460, AT4G04800, AT4G25130, AT4G03520, AT1G03680, AT3G15360	8	64	18499	252.916	2.17E-13	2.17E-13	7.48E-12
5	GO:0006120~mitochondrial electron transport, NADH to ubiquinone	2	15.38	6.47E-03	AT5G08530, AT4G02580	13	10	18499	284.6	1.66E-01	4.44E-02	5.28E+00
5	GO:0006097~glyoxylate cycle	2	15.38	6.47E-03	AT2G05710, AT4G26970	13	10	18499	284.6	1.66E-01	4.44E-02	5.28E+00
5	GO:0006102~isocitrate metabolic process	2	15.38	7.11E-03	AT2G05710, AT4G26970	13	11	18499	258.7273	1.81E-01	3.92E-02	5.80E+00
5	GO:0006101~citrate metabolic process	2	15.38	3.24E-03	AT2G05710, AT4G26970	13	5	18499	569.2	8.68E-02	2.98E-02	2.68E+00
5	GO:0006099~tricarboxylic acid cycle	4	30.77	4.80E-06	AT2G05710, AT2G47510, AT5G55070, AT4G26970	13	53	18499	107.3962	1.34E-04	6.72E-05	4.01E-03

5	GO:0006979~response to oxidative stress	9	69.23	1.60E-12	AT5G08670, AT4G02580, AT1G32220, AT2G05710, AT2G47510, AT5G37510, AT5G20140, AT5G55070, AT4G26970	13	291	18499	44.01031	4.47E-11	4.47E-11	1.34E-09
6	GO:0006979~response to oxidative stress	5	100.00	6.00E-08	AT2G22080, AT2G44240, AT1G50290, AT1G64360, AT2G19310	5	291	18499	63.57045	3.60E-07	3.60E-07	2.87E-05

Cellular Component

2	GO:0005847~mRNA cleavage and polyadenylation specificity factor complex	2	33.33	1.27E-03	AT5G23880, AT1G30460	5	8	25147	1257.35	8.87E-03	8.87E-03	6.54E-01
3	GO:0009514~glyoxysome	2	20.00	5.00E-03	AT4G35090, AT4G35000	10	14	25147	359.2429	1.22E-01	4.25E-02	4.03E+00
3	GO:0009507~chloroplast	6	60.00	6.19E-03	AT4G35090, AT4G25100, AT4G35000, AT5G18100, AT3G09640, AT2G28190	10	3855	25147	3.91393	1.49E-01	3.95E-02	4.96E+00
3	GO:0005777~peroxisome	3	30.00	2.78E-03	AT4G35090, AT4G35000, AT5G18100	10	226	25147	33.38097	6.97E-02	3.55E-02	2.25E+00
3	GO:0005829~cytosol	7	70.00	3.92E-05	AT4G35090, AT1G63460, AT4G25100, AT4G35000, AT3G09640, AT1G02930, AT1G08830	10	2309	25147	7.623603	1.02E-03	1.02E-03	3.21E-02
4	GO:0009507~chloroplast	6	75.00	1.35E-03	AT1G76080, AT5G07460, AT4G25130, AT4G03520, AT1G03680, AT3G15360	8	3855	25147	4.892412	2.01E-02	5.06E-03	9.35E-01
4	GO:0009579~thylakoid	3	37.50	1.36E-03	AT4G03520, AT1G03680, AT3G15360	8	206	25147	45.77731	2.03E-02	4.09E-03	9.45E-01
4	GO:0009941~chloroplast envelope	4	50.00	3.28E-04	AT1G76080, AT4G25130, AT1G03680, AT3G15360	8	543	25147	23.15562	4.92E-03	1.64E-03	2.28E-01
4	GO:0009570~chloroplast stroma	5	62.50	1.48E-05	AT1G76080, AT4G25130, AT4G03520, AT1G03680, AT3G15360	8	653	25147	24.06872	2.22E-04	1.11E-04	1.03E-02
4	GO:0005623~cell	5	62.50	1.13E-07	AT1G76080, AT3G06050, AT4G03520, AT1G03680, AT3G15360	8	192	25147	81.85872	1.70E-06	1.70E-06	7.87E-05
5	GO:0009507~chloroplast	7	53.85	5.18E-03	AT5G08670, AT5G64940, AT1G32220, AT2G05710, AT5G37510, AT5G20140, AT4G26970	13	3855	25147	3.512501	1.17E-01	4.07E-02	4.08E+00
5	GO:0005739~mitochondrion	11	84.62	1.04E-07	AT5G08670, AT5G08530, AT4G02580, AT1G32220, AT1G79010, AT1G16700, AT2G05710, AT2G47510, AT5G37510, AT5G55070, AT4G26970	13	3406	25147	6.247279	2.50E-06	1.25E-06	8.37E-05
5	GO:0005747~mitochondrial respiratory chain complex I	6	46.15	4.67E-11	AT5G08670, AT5G08530, AT4G02580, AT1G79010, AT1G16700, AT5G37510	13	59	25147	196.7171	1.12E-09	1.12E-09	3.75E-08

Molecular Function

1	GO:0043565~sequence-specific DNA binding	4	33.33	1.60E-03	AT3G46090, AT5G59820, AT1G27730, AT1G32640	8	676	18171	13.44009	2.85E-02	2.85E-02	1.18E+00
3	GO:0020037~heme binding	3	30.00	1.47E-02	AT4G35090, AT4G35000, AT3G09640	10	386	18171	14.12254	2.34E-01	4.34E-02	1.03E+01
3	GO:0004130~cytochrome-c peroxidase activity	2	20.00	3.96E-03	AT4G35000, AT3G09640	10	8	18171	454.275	6.89E-02	1.77E-02	2.88E+00
3	GO:0016688~L-ascorbate peroxidase activity	2	20.00	4.45E-03	AT4G35000, AT3G09640	10	9	18171	403.8	7.71E-02	1.59E-02	3.23E+00
3	GO:0046872~metal ion binding	6	60.00	4.99E-04	AT4G35090, AT4G25100, AT4G35000, AT3G09640, AT2G28190, AT1G08830	10	1606	18171	6.788667	8.94E-03	2.99E-03	3.67E-01
3	GO:0005507~copper ion binding	5	50.00	3.61E-06	AT4G25100, AT5G18100, AT1G02930, AT2G28190, AT1G08830	10	241	18171	37.69917	6.50E-05	3.25E-05	2.66E-03
3	GO:0004784~superoxide dismutase activity	4	40.00	4.70E-09	AT4G25100, AT5G18100, AT2G28190, AT1G08830	10	8	18171	908.55	8.46E-08	8.46E-08	3.46E-06
4	GO:0008047~enzyme activator activity	3	37.50	1.73E-05	AT4G03520, AT1G03680, AT3G15360	8	17	18171	400.8309	2.59E-04	4.31E-05	1.20E-02
4	GO:0015035~protein disulfide oxidoreductase activity	4	50.00	4.19E-06	AT1G76080, AT4G03520, AT1G03680, AT3G15360	8	91	18171	99.84066	6.29E-05	1.57E-05	2.91E-03
4	GO:0008113~peptide-methionine (S)-S-oxide reductase activity	3	37.50	4.57E-06	AT5G07460, AT4G04800, AT4G25130	8	9	18171	757.125	6.86E-05	1.37E-05	3.18E-03
4	GO:0016671~oxidoreductase activity, acting on a sulfur group of donors, disulfide as acceptor	4	50.00	4.61E-07	AT1G76080, AT4G03520, AT1G03680, AT3G15360	8	44	18171	206.4886	6.91E-06	3.45E-06	3.20E-04
4	GO:0004791~thioredoxin-disulfide reductase activity	4	50.00	6.40E-07	AT1G76080, AT4G03520, AT1G03680, AT3G15360	8	49	18171	185.4184	9.60E-06	3.20E-06	4.45E-04
4	GO:0047134~protein-disulfide reductase activity	4	50.00	1.27E-07	AT1G76080, AT4G03520, AT1G03680, AT3G15360	8	29	18171	313.2931	1.91E-06	1.91E-06	8.86E-05
5	GO:0005507~copper ion binding	3	23.08	1.06E-02	AT5G08670, AT2G05710, AT4G26970	13	241	18171	17.39962	2.00E-01	4.37E-02	7.89E+00
5	GO:0003994~aconitate hydratase activity	2	15.38	1.98E-03	AT2G05710, AT4G26970	13	3	18171	931.8462	4.08E-02	1.04E-02	1.52E+00
5	GO:0003954~NADH dehydrogenase activity	3	23.08	3.11E-05	AT5G08530, AT1G32220, AT5G37510	13	13	18171	322.5621	6.52E-04	2.17E-04	2.40E-02
5	GO:0008137~NADH dehydrogenase (ubiquinone) activity	5	38.46	7.12E-09	AT5G08530, AT4G02580, AT1G79010, AT1G16700, AT5G37510	13	37	18171	188.8877	1.49E-07	7.47E-08	5.49E-06
5	GO:0051539~4 iron, 4 sulfur cluster binding	6	46.15	3.59E-10	AT5G08530, AT1G79010, AT1G16700, AT2G05710, AT5G37510, AT4G26970	13	64	18171	131.0409	7.54E-09	7.54E-09	2.77E-07

Table S12. Gene ontology terms associated with specific gene clusters in the salt stress associated gene network obtained using DAVID server. The description of the columns as in Table S7.

Cluster	Term	Count	%	PValue	Genes	List Total	Pop Hits	Pop Total	Fold Enrichment	Bonferroni	Benjamini	FDR
**Biological Process**												
1	GO:0010119~regulation of stomatal movement	3	8.82	2.91E-03	AT4G23650, AT4G26080, AT4G33950	34	45	18499	36.273	2.51E-01	1.91E-02	3.18E+00
1	GO:0009789~positive regulation of abscisic acid-activated signaling pathway	3	8.82	1.67E-03	AT3G50500, AT5G66880, AT3G17980	34	34	18499	48.008	1.53E-01	1.18E-02	1.84E+00
1	GO:0006814~sodium ion transport	3	8.82	1.39E-03	AT5G27150, AT2G01980, AT4G10310	34	31	18499	52.654	1.29E-01	1.05E-02	1.53E+00
1	GO:0051453~regulation of intracellular pH	3	8.82	1.13E-03	AT5G27150, AT2G01980, AT3G47950	34	28	18499	58.295	1.06E-01	1.02E-02	1.25E+00
1	GO:0010118~stomatal movement	3	8.82	1.22E-03	AT1G16540, AT4G33950, AT4G17615	34	29	18499	56.285	1.13E-01	9.99E-03	1.34E+00
1	GO:0006470~protein dephosphorylation	4	11.76	8.10E-04	AT5G57050, AT2G33700, AT1G72770, AT4G26080	34	103	18499	21.130	7.71E-02	7.99E-03	8.94E-01
1	GO:2000377~regulation of reactive oxygen species metabolic process	3	8.82	6.34E-04	AT2G01980, AT4G33950, AT1G32230	34	21	18499	77.727	6.09E-02	6.96E-03	7.01E-01
1	GO:0080163~regulation of protein serine/threonine phosphatase activity	3	8.82	5.18E-04	AT4G17870, AT5G46790, AT2G26040	34	19	18499	85.909	5.00E-02	6.39E-03	5.72E-01
1	GO:0009737~response to abscisic acid	10	29.41	2.02E-08	AT5G57050, AT3G61890, AT3G46930, AT2G33700, AT4G26080, AT4G33950, AT3G50500, AT1G10940, AT3G17510, AT5G66880	34	394	18499	13.809	2.00E-06	2.86E-07	2.24E-05
1	GO:0006468~protein phosphorylation	14	41.18	1.20E-09	AT5G08590, AT5G35410, AT4G33950, AT1G60940, AT5G26751, AT1G10940, AT5G66880, AT3G46930, AT4G23650, AT4G40010, AT5G57630, AT5G63650, AT3G50500, AT3G17510	34	870	18499	8.755	1.19E-07	1.99E-08	1.33E-06
1	GO:0009414~response to water deprivation	12	35.29	1.09E-12	AT5G57050, AT2G26650, AT3G61890, AT3G46930, AT2G33700, AT1G15690, AT4G33950, AT3G50500, AT4G17615, AT4G01420, AT5G66880, AT1G32230	34	279	18499	23.402	1.08E-10	2.15E-11	1.21E-09
1	GO:0009738~abscisic acid-activated signaling pathway	12	35.29	2.13E-14	AT5G57050, AT4G17870, AT4G23650, AT1G72770, AT5G46790, AT4G26080, AT4G33950, AT3G50500, AT2G26040, AT4G17615, AT5G66880, AT3G17980	34	195	18499	33.482	2.11E-12	5.28E-13	2.36E-11
1	GO:0035556~intracellular signal transduction	12	35.29	1.50E-14	AT5G08590, AT5G57630, AT4G40010, AT4G23650, AT5G63650, AT5G35410, AT4G33950, AT1G60940, AT3G50500, AT1G10940, AT3G17510, AT5G66880	34	189	18499	34.545	1.48E-12	4.95E-13	1.66E-11
1	GO:0006970~response to osmotic stress	17	50.00	4.88E-27	AT1G16540, AT5G08590, AT4G33950, AT4G10310, AT1G60940, AT4G17615, AT1G10940, AT5G66880, AT4G01420, AT1G32230, AT5G57050, AT3G46930, AT3G61890, AT4G40010, AT5G63650, AT3G50500, AT3G17510	34	122	18499	75.816	4.83E-25	2.41E-25	5.40E-24
1	GO:0009651~response to salt stress	27	79.41	1.33E-35	AT4G10310, AT1G60940, AT5G66880, AT1G32230, AT3G47950, AT2G26650, AT3G46930, AT3G61890, AT2G33700, AT5G63650, AT3G50500, AT3G17510, AT3G17980, AT1G16540, AT5G08590, AT1G06040, AT5G35410, AT4G33950, AT5G26751, AT1G10940, AT4G17615, AT4G01420, AT4G23650, AT4G40010, AT5G27150, AT2G01980, AT1G15690	34	484	18499	30.352	1.32E-33	1.32E-33	1.48E-32
2	GO:0070370~cellular heat acclimation	2	14.29	5.93E-03	AT2G30250, AT2G38470	12	10	18499	308.317	3.64E-01	4.90E-02	6.07E+00
2	GO:0010337~regulation of salicylic acid metabolic process	2	14.29	4.75E-03	AT1G28380, AT2G41010	12	8	18499	385.396	3.04E-01	4.42E-02	4.88E+00
2	GO:0010200~response to chitin	3	21.43	2.70E-03	AT3G23250, AT1G27730, AT2G38470	12	133	18499	34.773	1.86E-01	2.90E-02	2.81E+00
2	GO:0006970~response to osmotic stress	3	21.43	2.28E-03	AT2G30250, AT2G38470, AT2G43790	12	122	18499	37.908	1.59E-01	2.85E-02	2.38E+00
2	GO:0009620~response to fungus	3	21.43	8.03E-04	AT4G01370, AT1G73500, AT2G43790	12	72	18499	64.233	5.92E-02	1.21E-02	8.42E-01
2	GO:0009414~response to water deprivation	4	28.57	5.12E-04	AT1G27730, AT2G38470, AT3G50310, AT2G41010	12	279	18499	22.102	3.82E-02	9.68E-03	5.37E-01
2	GO:0010120~camalexin biosynthetic process	3	21.43	1.44E-05	AT2G38470, AT1G73500, AT2G43790	12	10	18499	462.475	1.10E-03	3.65E-04	1.52E-02
2	GO:0009409~response to cold	7	50.00	7.32E-09	AT4G01370, AT4G29810, AT2G30250, AT1G27730, AT2G38470, AT2G43790, AT3G50310	12	299	18499	36.091	5.56E-07	2.78E-07	7.70E-06
2	GO:0009651~response to salt stress	12	85.71	3.52E-18	AT4G01370, AT1G28380, AT3G23250, AT4G29810, AT3G55270, AT2G30250, AT1G27730, AT2G38470, AT1G73500, AT2G43790, AT3G50310, AT2G41010	12	484	18499	38.221	2.67E-16	2.67E-16	3.70E-15
3	GO:0006950~response to stress	3	37.50	2.84E-04	AT5G56030, AT2G04030, AT4G24190	8	69	18499	100.538	1.08E-02	2.16E-03	2.57E-01
3	GO:0009414~response to water deprivation	4	50.00	1.14E-04	AT5G56030, AT2G04030, AT4G24190, AT3G50310	8	279	18499	33.152	4.31E-03	1.08E-03	1.03E-01
3	GO:0006457~protein folding	5	62.50	1.94E-06	AT3G44110, AT1G59860, AT5G56030, AT2G04030, AT4G24190	8	288	18499	40.145	7.38E-05	2.46E-05	1.75E-03

3	GO:0009408~response to heat	5	62.50	1.85E-07	AT3G09350, AT3G44110, AT1G59860, AT5G56030, AT2G04030	8	160	18499	72.262	7.03E-06	3.51E-06	1.67E-04
3	GO:0009651~response to salt stress	8	100.00	8.04E-12	AT3G09350, AT3G44110, AT1G59860, AT1G53300, AT5G56030, AT2G04030, AT4G24190, AT3G50310	8	484	18499	38.221	3.06E-10	3.06E-10	7.27E-09
4	GO:0006355~regulation of transcription, DNA-templated	4	66.67	1.26E-02	AT1G66350, AT2G01570, AT3G03450, AT1G14920	6	2119	18499	5.820	3.41E-01	2.93E-02	1.04E+01
4	GO:0010218~response to far red light	2	33.33	1.32E-02	AT2G01570, AT1G14920	6	49	18499	125.844	3.54E-01	2.88E-02	1.09E+01
4	GO:0006351~transcription, DNA-templated	4	66.67	9.03E-03	AT1G66350, AT2G01570, AT3G03450, AT1G14920	6	1886	18499	6.539	2.59E-01	2.28E-02	7.61E+00
4	GO:0009737~response to abscisic acid	4	66.67	9.29E-05	AT1G66350, AT2G01570, AT3G03450, AT1G14920	6	394	18499	31.301	3.06E-03	2.55E-04	8.10E-02
4	GO:0010029~regulation of seed germination	3	50.00	4.54E-05	AT2G01570, AT3G03450, AT1G14920	6	40	18499	231.238	1.50E-03	1.36E-04	3.96E-02
4	GO:0009723~response to ethylene	4	66.67	2.91E-06	AT1G66350, AT2G01570, AT3G03450, AT1G14920	6	124	18499	99.457	9.61E-05	9.61E-06	2.54E-03
4	GO:0009651~response to salt stress	5	83.33	2.27E-06	AT1G66350, AT2G01570, AT1G60220, AT3G03450, AT1G14920	6	484	18499	31.851	7.48E-05	8.31E-06	1.98E-03
4	GO:0009739~response to gibberellin	4	66.67	1.48E-06	AT1G66350, AT3G05120, AT3G03450, AT1G14920	6	99	18499	124.572	4.87E-05	6.09E-06	1.29E-03
4	GO:0042538~hyperosmotic salinity response	4	66.67	2.76E-07	AT1G66350, AT2G01570, AT3G03450, AT1G14920	6	57	18499	216.363	9.11E-06	1.30E-06	2.41E-04
4	GO:0009867~jasmonic acid mediated signaling pathway	4	66.67	2.09E-07	AT1G66350, AT2G01570, AT3G03450, AT1G14920	6	52	18499	237.167	6.89E-06	1.15E-06	1.82E-04
4	GO:0009863~salicylic acid mediated signaling pathway	4	66.67	2.46E-08	AT1G66350, AT2G01570, AT3G03450, AT1G14920	6	26	18499	474.333	8.12E-07	1.62E-07	2.15E-05
4	GO:2000377~regulation of reactive oxygen species metabolic process	4	66.67	1.26E-08	AT1G66350, AT2G01570, AT3G03450, AT1G14920	6	21	18499	587.270	4.15E-07	1.04E-07	1.10E-05
4	GO:0010187~negative regulation of seed germination	4	66.67	1.26E-08	AT1G66350, AT2G01570, AT3G03450, AT1G14920	6	21	18499	587.270	4.15E-07	1.04E-07	1.10E-05
4	GO:2000033~regulation of seed dormancy process	4	66.67	7.96E-10	AT1G66350, AT2G01570, AT3G03450, AT1G14920	6	9	18499	1370.296	2.63E-08	2.63E-08	6.95E-07
4	GO:0009740~gibberellic acid mediated signaling pathway	5	83.33	1.11E-09	AT1G66350, AT2G01570, AT3G05120, AT3G03450, AT1G14920	6	73	18499	211.176	3.67E-08	1.84E-08	9.71E-07
4	GO:0009938~negative regulation of gibberellic acid mediated signaling pathway	4	66.67	1.56E-09	AT1G66350, AT2G01570, AT3G03450, AT1G14920	6	11	18499	1121.152	5.16E-08	1.72E-08	1.36E-06
5	GO:0006979~response to oxidative stress	3	60.00	1.45E-03	AT3G47450, AT2G16500, AT4G34710	5	291	18499	38.142	4.12E-02	1.05E-02	1.22E+00
5	GO:0008295~spermidine biosynthetic process	2	40.00	1.73E-03	AT2G16500, AT4G34710	5	8	18499	924.950	4.89E-02	9.99E-03	1.45E+00
5	GO:0006596~polyamine biosynthetic process	2	40.00	1.94E-03	AT2G16500, AT4G34710	5	9	18499	822.178	5.49E-02	9.36E-03	1.63E+00
5	GO:0006527~arginine catabolic process	2	40.00	6.49E-04	AT2G16500, AT4G34710	5	3	18499	2466.533	1.86E-02	9.36E-03	5.46E-01
5	GO:0033388~putrescine biosynthetic process from arginine	2	40.00	8.65E-04	AT2G16500, AT4G34710	5	4	18499	1849.900	2.48E-02	8.33E-03	7.28E-01
5	GO:0009651~response to salt stress	4	80.00	6.98E-05	AT3G47450, AT3G51920, AT2G16500, AT4G34710	5	484	18499	30.577	2.02E-03	2.02E-03	5.89E-02
Cellular Component												
1	GO:0009705~plant-type vacuole membrane	3	8.82	8.69E-03	AT5G57630, AT1G15690, AT5G35410	34	107	25147	20.737	1.89E-01	3.43E-02	6.76E+00
1	GO:0008287~protein serine/threonine phosphatase complex	2	5.88	5.24E-03	AT5G57050, AT4G26080	34	4	25147	369.809	1.18E-01	2.49E-02	4.13E+00
1	GO:0005829~cytosol	11	32.35	5.36E-04	AT5G57630, AT4G40010, AT4G23650, AT1G15690, AT4G33950, AT1G60940, AT3G50500, AT5G26751, AT1G10940, AT5G66880, AT3G17980	34	2309	25147	3.524	1.28E-02	3.21E-03	4.29E-01
1	GO:0005634~nucleus	25	73.53	8.60E-05	AT1G72770, AT2G26040, AT1G60940, AT5G66880, AT1G32230, AT3G61890, AT3G46930, AT2G33700, AT4G26080, AT5G46790, AT5G63650, AT3G50500, AT3G17510, AT3G17980, AT5G08590, AT1G06040, AT5G35410, AT4G33950, AT5G26751, AT1G10940, AT4G01420, AT4G17870, AT4G40010, AT5G57630, AT4G23650	34	9796	25147	1.888	2.06E-03	6.88E-04	6.90E-02
1	GO:0005737~cytoplasm	18	52.94	8.95E-06	AT1G16540, AT5G08590, AT1G72770, AT4G33950, AT2G26040, AT4G17615, AT4G01420, AT5G66880, AT1G32230, AT4G17870, AT2G33700, AT4G40010, AT5G57630, AT5G63650, AT5G46790, AT4G26080, AT3G50500, AT3G17510	34	4407	25147	3.021	2.15E-04	1.07E-04	7.18E-03
1	GO:0005886~plasma membrane	18	52.94	7.53E-07	AT5G35410, AT4G10310, AT2G26040, AT1G60940, AT4G17615, AT4G01420, AT3G47950, AT2G26650, AT4G17870, AT4G23650, AT5G27150, AT2G01980, AT1G15690, AT5G46790, AT4G26080, AT3G50500, AT3G17510, AT3G17980	34	3702	25147	3.596	1.81E-05	1.81E-05	6.04E-04
4	GO:0005634~nucleus	6	100.00	8.96E-03	AT1G66350, AT2G01570, AT3G05120, AT1G60220, AT3G03450, AT1G14920	6	9796	25147	2.567	2.67E-02	2.67E-02	2.79E+00
Molecular Function												
1	GO:0004872~receptor activity	3	8.82	2.39E-03	AT4G17870, AT5G46790, AT2G26040	34	40	18171	40.083	1.06E-01	1.40E-02	2.25E+00

1	GO:0004722~protein serine/threonine phosphatase activity	4	11.76	2.51E-03	AT5G57050, AT2G33700, AT1G72770, AT4G26080	34	150	18171	14.252	1.11E-01	1.30E-02	2.36E+00
1	GO:0010427~abscisic acid binding	3	8.82	5.36E-04	AT4G17870, AT5G46790, AT2G26040	34	19	18171	84.385	2.49E-02	3.60E-03	5.08E-01
1	GO:0004864~protein phosphatase inhibitor activity	3	8.82	3.78E-04	AT4G17870, AT5G46790, AT2G26040	34	16	18171	100.208	1.76E-02	2.96E-03	3.58E-01
1	GO:0005524~ATP binding	15	44.12	2.88E-05	AT5G08590, AT5G35410, AT4G33950, AT1G60940, AT5G26751, AT1G10940, AT5G66880, AT3G47950, AT3G46930, AT4G23650, AT4G40010, AT5G57630, AT5G63650, AT3G50500, AT3G17510	34	2372	18171	3.380	1.35E-03	2.71E-04	2.73E-02
1	GO:0004672~protein kinase activity	9	26.47	1.79E-06	AT5G08590, AT4G23650, AT5G35410, AT4G33950, AT1G60940, AT3G50500, AT5G26751, AT1G10940, AT5G66880	34	482	18171	9.979	8.42E-05	2.10E-05	1.70E-03
1	GO:0016301~kinase activity	13	38.24	1.33E-07	AT5G08590, AT5G35410, AT4G33950, AT1G60940, AT1G10940, AT5G66880, AT3G46930, AT4G23650, AT4G40010, AT5G57630, AT5G63650, AT3G50500, AT3G17510	34	1039	18171	6.687	6.24E-06	2.08E-06	1.26E-04
1	GO:0005515~protein binding	18	52.94	4.54E-09	AT1G06040, AT1G72770, AT5G35410, AT4G33950, AT2G26040, AT5G66880, AT1G32230, AT5G57050, AT4G17870, AT2G26650, AT5G27150, AT5G57630, AT2G01980, AT5G46790, AT4G26080, AT3G50500, AT3G17510, AT3G17980	34	1901	18171	5.060	2.13E-07	2.13E-07	4.31E-06
1	GO:0004674~protein serine/threonine kinase activity	13	38.24	7.80E-09	AT5G08590, AT5G35410, AT4G33950, AT1G60940, AT5G26751, AT1G10940, AT5G66880, AT4G23650, AT4G40010, AT5G57630, AT5G63650, AT3G50500, AT3G17510	34	804	18171	8.641	3.67E-07	1.83E-07	7.41E-06
2	GO:0004707~MAP kinase activity	2	14.29	1.38E-02	AT4G01370, AT2G43790	12	23	18171	131.674	2.54E-01	4.76E-02	1.02E+01
2	GO:0044212~transcription regulatory region DNA binding	3	21.43	1.52E-02	AT3G23250, AT1G27730, AT2G38470	12	319	18171	14.241	2.75E-01	4.50E-02	1.12E+01
2	GO:0004708~MAP kinase kinase activity	2	14.29	6.64E-03	AT4G29810, AT1G73500	12	11	18171	275.318	1.31E-01	3.44E-02	5.01E+00
2	GO:0043565~sequence-specific DNA binding	4	28.57	6.76E-03	AT3G23250, AT2G30250, AT1G27730, AT2G38470	12	676	18171	8.960	1.33E-01	2.81E-02	5.10E+00
2	GO:0016301~kinase activity	5	35.71	2.54E-03	AT4G01370, AT4G29810, AT1G73500, AT2G43790, AT3G50310	12	1039	18171	7.287	5.19E-02	2.63E-02	1.94E+00
2	GO:0005515~protein binding	6	42.86	3.35E-03	AT4G01370, AT4G29810, AT2G30250, AT2G38470, AT2G43790, AT2G41010	12	1901	18171	4.779	6.81E-02	2.32E-02	2.56E+00
2	GO:0004702~receptor signaling protein serine/threonine kinase activity	3	21.43	4.35E-04	AT4G29810, AT1G73500, AT3G50310	12	52	18171	87.361	9.09E-03	9.09E-03	3.35E-01
3	GO:0005524~ATP binding	5	62.50	3.49E-03	AT3G44110, AT5G56030, AT2G04030, AT4G24190, AT3G50310	7	2372	18171	5.472	3.43E-02	1.73E-02	2.08E+00
3	GO:0051082~unfolded protein binding	4	50.00	2.10E-06	AT3G44110, AT5G56030, AT2G04030, AT4G24190	7	87	18171	119.350	2.10E-05	2.10E-05	1.26E-03
4	GO:0005515~protein binding	4	66.67	9.72E-03	AT2G01570, AT3G05120, AT3G03450, AT1G14920	6	1901	18171	6.372	8.41E-02	2.17E-02	5.46E+00
4	GO:0003700~transcription factor activity, sequence-specific DNA binding	4	66.67	7.20E-03	AT1G66350, AT2G01570, AT3G03450, AT1G14920	6	1711	18171	7.080	6.30E-02	2.15E-02	4.08E+00
4	GO:0043565~sequence-specific DNA binding	4	66.67	4.85E-04	AT1G66350, AT2G01570, AT3G03450, AT1G14920	6	676	18171	17.920	4.35E-03	2.18E-03	2.79E-01
4	GO:0000989~transcription factor activity, transcription factor binding	4	66.67	2.86E-09	AT1G66350, AT2G01570, AT3G03450, AT1G14920	6	13	18171	931.846	2.57E-08	2.57E-08	1.64E-06
5	GO:0008792~arginine decarboxylase activity	2	40.00	4.40E-04	AT2G16500, AT4G34710	5	2	18171	3634.200	5.71E-03	5.71E-03	2.91E-01

Table S13. Gene ontology terms associated with specific gene clusters in the water stress associated gene network obtained using DAVID server. The description of the columns as in Table S7.

Cluster	Term	Count	%	PValue	Genes	List Total	Pop Hits	Pop Total	Fold Enrichment	Bonferroni	Benjamini	FDR
Biological Process												
1	GO:0045893~positive regulation of transcription, DNA-templated	5	9.09	3.68E-03	AT2G40220, AT4G27410, AT1G69600, AT5G05410, AT1G45249	55	217	18499	7.749895	4.89E-01	3.66E-02	4.45E+00
1	GO:0006470~protein dephosphorylation	4	7.27	3.38E-03	AT3G16800, AT5G27930, AT1G72770, AT4G26080	55	103	18499	13.06196	4.60E-01	3.56E-02	4.10E+00
1	GO:0080163~regulation of protein serine/threonine phosphatase activity	3	5.45	1.39E-03	AT4G27920, AT4G17870, AT2G26040	55	19	18499	53.10718	2.23E-01	1.56E-02	1.70E+00
1	GO:0006950~response to stress	4	7.27	1.08E-03	AT1G20450, AT1G20440, AT2G04030, AT4G24190	55	69	18499	19.49829	1.78E-01	1.30E-02	1.32E+00
1	GO:0009688~abscisic acid biosynthetic process	3	5.45	8.57E-04	AT1G30100, AT3G14440, AT1G52340	55	15	18499	67.26909	1.44E-01	1.11E-02	1.05E+00
1	GO:0042538~hyperosmotic salinity response	4	7.27	6.15E-04	AT2G39800, AT3G14080, AT3G14440, AT1G19120	55	57	18499	23.60319	1.06E-01	8.58E-03	7.57E-01
1	GO:0042542~response to hydrogen peroxide	4	7.27	5.25E-04	AT2G43350, AT4G26070, AT5G05410, AT1G32230	55	54	18499	24.91448	9.11E-02	7.93E-03	6.46E-01
1	GO:0010119~regulation of stomatal movement	4	7.27	3.06E-04	AT2G18960, AT2G40220, AT4G26080, AT4G33950	55	45	18499	29.89737	5.42E-02	5.05E-03	3.77E-01
1	GO:0010107~potassium ion import	3	5.45	8.32E-05	AT2G26650, AT1G30270, AT4G17615	55	5	18499	201.8073	1.50E-02	1.51E-03	1.03E-01
1	GO:0009788~negative regulation of abscisic acid-activated signaling pathway	5	9.09	4.94E-06	AT2G39550, AT1G08720, AT4G26080, AT5G40280, AT3G59380	55	39	18499	43.12121	8.99E-04	1.00E-04	6.10E-03
1	GO:0009409~response to cold	9	16.36	2.31E-06	AT1G20450, AT4G25480, AT1G78080, AT1G20440, AT4G26080, AT4G24190, AT3G50310, AT4G17615, AT2G42540	55	299	18499	10.12411	4.21E-04	5.27E-05	2.86E-03
1	GO:0010118~stomatal movement	5	9.09	1.46E-06	AT1G30270, AT1G12480, AT4G24020, AT4G33950, AT4G17615	55	29	18499	57.9906	2.65E-04	3.79E-05	1.80E-03
1	GO:0009631~cold acclimation	6	10.91	3.72E-07	AT1G20450, AT4G25480, AT3G14080, AT1G20440, AT1G19120, AT2G42540	55	51	18499	39.57005	6.77E-05	1.13E-05	4.59E-04
1	GO:0009737~response to abscisic acid	13	23.64	1.13E-09	AT1G20450, AT2G18960, AT2G39800, AT2G39550, AT1G12480, AT1G20440, AT4G26080, AT4G27410, AT4G33950, AT5G40280, AT3G50500, AT2G42540, AT1G45249	55	394	18499	11.09769	2.06E-07	4.11E-08	1.39E-06
1	GO:0006970~response to osmotic stress	10	18.18	7.25E-11	AT2G40220, AT1G78080, AT1G20440, AT3G14440, AT4G33950, AT3G50500, AT4G17615, AT4G01420, AT2G42540, AT1G32230	55	122	18499	27.5693	1.32E-08	3.30E-09	8.95E-08
1	GO:0009651~response to salt stress	16	29.09	5.04E-12	AT1G74920, AT2G39800, AT1G78080, AT2G04030, AT4G33950, AT4G17615, AT4G01420, AT2G42540, AT1G32230, AT1G45249, AT2G26650, AT1G15690, AT4G24190, AT3G50310, AT3G50500, AT1G10370	55	484	18499	11.11886	9.18E-10	3.06E-10	6.22E-09
1	GO:0009738~abscisic acid-activated signaling pathway	13	23.64	3.12E-13	AT2G43350, AT1G72770, AT4G33950, AT2G26040, AT4G17615, AT1G45249, AT4G27920, AT4G17870, AT2G40220, AT1G12480, AT1G08720, AT4G26080, AT3G50500	55	195	18499	22.42303	5.68E-11	2.84E-11	3.85E-10
1	GO:0009414~response to water deprivation	42	76.36	9.21E-65	AT1G74920, AT1G20450, AT3G14080, AT1G11755, AT3G15500, AT4G26070, AT4G24020, AT2G04030, AT1G52890, AT1G45249, AT1G32230, AT2G26650, AT4G25480, AT1G30100, AT4G27410, AT5G40280, AT3G50500, AT3G50310, AT3G59380, AT2G18960, AT2G30580, AT2G39800, AT1G30270, AT1G78080, AT2G39550, AT1G20440, AT4G33950, AT4G17615, AT4G01420, AT2G42540, AT1G06770, AT1G15690, AT2G40220, AT3G30775, AT1G08720, AT3G14440, AT1G12110, AT1G69600, AT1G19120, AT4G24190, AT5G05410, AT1G52340	55	279	18499	50.63265	1.68E-62	1.68E-62	1.14E-61
2	GO:0002679~respiratory burst involved in defense response	2	20.00	2.43E-03	AT3G52450, AT2G35930	10	5	18499	739.96	7.94E-02	1.17E-02	2.12E+00
2	GO:0000209~protein polyubiquitination	3	30.00	1.34E-03	AT5G59550, AT3G46620, AT3G56580	10	115	18499	48.25826	4.46E-02	7.57E-03	1.17E+00
2	GO:0043161~proteasome-mediated ubiquitin-dependent protein catabolic process	4	40.00	2.07E-04	AT5G59550, AT3G46620, AT3G56580, AT3G13672	10	256	18499	28.90469	7.01E-03	1.41E-03	1.82E-01
2	GO:0042787~protein ubiquitination involved in ubiquitin-dependent protein catabolic process	4	40.00	6.93E-05	AT5G59550, AT3G46620, AT3G56580, AT3G13672	10	177	18499	41.80565	2.35E-03	5.89E-04	6.10E-02
2	GO:0009414~response to water deprivation	6	60.00	9.03E-08	AT5G59550, AT3G46620, AT3G52450, AT2G35930, AT1G27730, AT3G56580	10	279	18499	39.7828	3.07E-06	1.02E-06	7.94E-05
2	GO:0051865~protein autoubiquitination	4	40.00	2.89E-08	AT5G59550, AT3G46620, AT3G52450, AT2G35930	10	14	18499	528.5429	9.83E-07	4.91E-07	2.54E-05
2	GO:0010200~response to chitin	6	60.00	2.19E-09	AT5G59550, AT3G23250, AT3G46620, AT3G52450, AT2G35930, AT1G27730	10	133	18499	83.45414	7.46E-08	7.46E-08	1.93E-06
3	GO:0051301~cell division	4	50.00	5.46E-05	AT1G18040, AT5G60410, AT5G27620, AT1G66750	8	218	18499	42.4289	2.72E-03	1.36E-03	5.25E-02
3	GO:0007049~cell cycle	4	50.00	9.61E-06	AT1G18040, AT5G27620, AT1G66750, AT4G30820	8	122	18499	75.81557	4.80E-04	4.80E-04	9.25E-03
4	GO:0080170~hydrogen peroxide transmembrane transport	2	20.00	2.43E-03	AT3G53420, AT2G36830	10	5	18499	739.96	3.35E-02	4.86E-03	1.64E+00

4	GO:0015840~urea transport	2	20.00	1.94E-03	AT3G16240, AT2G36830	10	4	18499	924.95	2.69E-02	4.53E-03	1.31E+00
4	GO:0009414~response to water deprivation	6	60.00	9.03E-08	AT3G53420, AT2G37180, AT3G61430, AT1G01620, AT2G45960, AT4G00430	10	279	18499	39.7828	1.26E-06	2.53E-07	6.13E-05
4	GO:0006810~transport	10	100.00	2.73E-16	AT3G53420, AT2G37180, AT3G54820, AT3G16240, AT3G61430, AT2G36830, AT4G35100, AT1G01620, AT2G45960, AT4G00430	10	349	18499	53.00573	3.11E-15	7.77E-16	1.55E-13
4	GO:0006833~water transport	8	80.00	8.08E-23	AT3G53420, AT3G54820, AT3G16240, AT3G61430, AT2G36830, AT1G01620, AT2G45960, AT4G00430	10	11	18499	1345.382	1.13E-21	5.66E-22	5.49E-20
4	GO:0034220~ion transmembrane transport	9	90.00	8.23E-23	AT3G53420, AT2G37180, AT3G54820, AT3G61430, AT2G36830, AT4G35100, AT1G01620, AT2G45960, AT4G00430	10	28	18499	594.6107	1.15E-21	3.84E-22	5.59E-20
4	GO:0009992~cellular water homeostasis	10	100.00	1.78E-25	AT3G53420, AT2G37180, AT3G54820, AT3G16240, AT3G61430, AT2G36830, AT4G35100, AT1G01620, AT2G45960, AT4G00430	10	37	18499	499.973	2.50E-24	2.50E-24	1.21E-22
Cellular Component												
1	GO:0005737~cytoplasm	20	36.36	1.10E-03	AT1G20450, AT3G16800, AT5G27930, AT2G43350, AT1G30270, AT2G39800, AT1G72770, AT4G26070, AT4G33950, AT2G26040, AT4G17615, AT4G01420, AT1G32230, AT4G27920, AT1G06770, AT4G17870, AT4G26080, AT3G50310, AT3G50500, AT1G10370	54	4407	25147	2.113389	4.30E-02	2.17E-02	1.00E+00
1	GO:0005634~nucleus	34	61.82	4.97E-04	AT1G20450, AT3G14080, AT1G72770, AT3G15500, AT4G24020, AT2G26040, AT1G52890, AT1G45249, AT1G32230, AT1G18390, AT4G25480, AT4G26080, AT4G27410, AT3G50500, AT3G59380, AT2G18960, AT3G16800, AT2G30580, AT1G30270, AT1G78080, AT2G39550, AT1G20440, AT4G33950, AT4G01420, AT4G27920, AT1G06770, AT4G17870, AT2G40220, AT3G53600, AT1G08720, AT1G69600, AT1G19120, AT4G24190, AT5G05410	54	9796	25147	1.616302	1.97E-02	1.97E-02	4.54E-01
2	GO:0000145~exocyst	2	20.00	1.11E-02	AT5G58430, AT1G07000	9	35	25147	159.6635	7.50E-02	2.57E-02	5.58E+00
2	GO:0070062~extracellular exosome	2	20.00	1.59E-03	AT5G58430, AT1G07000	9	5	25147	1117.644	1.11E-02	5.55E-03	8.17E-01
2	GO:0005829~cytosol	6	60.00	2.87E-04	AT5G59550, AT5G58430, AT1G07000, AT3G46620, AT3G52450, AT2G35930	9	2309	25147	7.260575	2.01E-03	2.01E-03	1.48E-01
3	GO:0005634~nucleus	8	100.00	1.36E-03	AT1G18040, AT1G03190, AT5G60410, AT3G19600, AT5G27620, AT1G66750, AT5G64960, AT4G30820	8	9796	25147	2.567068	1.22E-02	1.22E-02	7.80E-01
4	GO:0009941~chloroplast envelope	3	30.00	1.52E-02	AT3G16240, AT3G61430, AT2G36830	10	543	25147	13.89337	2.52E-01	3.56E-02	1.08E+01
4	GO:0000326~protein storage vacuole	2	20.00	5.71E-03	AT3G16240, AT2G36830	10	16	25147	314.3375	1.03E-01	1.54E-02	4.20E+00
4	GO:0042807~central vacuole	2	20.00	3.57E-03	AT3G16240, AT2G36830	10	10	25147	502.94	6.58E-02	1.13E-02	2.65E+00
4	GO:0005886~plasma membrane	9	90.00	1.71E-06	AT3G53420, AT2G37180, AT3G54820, AT3G16240, AT3G61430, AT4G35100, AT1G01620, AT2G45960, AT4G00430	10	3702	25147	6.113533	3.26E-05	6.52E-06	1.29E-03
4	GO:0016020~membrane	10	100.00	4.77E-11	AT3G53420, AT2G37180, AT3G54820, AT3G16240, AT3G61430, AT2G36830, AT4G35100, AT1G01620, AT2G45960, AT4G00430	10	1797	25147	13.99388	9.06E-10	3.02E-10	3.57E-08
4	GO:0009506~plasmodesma	9	90.00	5.63E-11	AT3G53420, AT2G37180, AT3G54820, AT3G16240, AT3G61430, AT4G35100, AT1G01620, AT2G45960, AT4G00430	10	1008	25147	22.45268	1.07E-09	2.68E-10	4.22E-08
4	GO:0005773~vacuole	10	100.00	3.62E-15	AT3G53420, AT2G37180, AT3G54820, AT3G16240, AT3G61430, AT2G36830, AT4G35100, AT1G01620, AT2G45960, AT4G00430	10	629	25147	39.97933	6.96E-14	3.49E-14	2.74E-12
4	GO:0005887~integral component of plasma membrane	10	100.00	1.96E-18	AT3G53420, AT2G37180, AT3G54820, AT3G16240, AT3G61430, AT2G36830, AT4G35100, AT1G01620, AT2G45960, AT4G00430	10	275	25147	91.44364	3.73E-17	3.73E-17	1.47E-15
Molecular Function												
1	GO:0004864~protein phosphatase inhibitor activity	3	5.45	9.76E-04	AT4G27920, AT4G17870, AT2G26040	54	16	18171	63.09375	7.15E-02	3.64E-02	1.02E+00
1	GO:0010427~abscisic acid binding	3	5.45	1.38E-03	AT4G27920, AT4G17870, AT2G26040	54	19	18171	53.13158	9.98E-02	3.44E-02	1.45E+00
1	GO:0042803~protein homodimerization activity	5	9.09	1.83E-03	AT4G27920, AT4G17870, AT1G69600, AT2G04030, AT2G26040	54	179	18171	9.399441	1.30E-01	3.42E-02	1.91E+00
1	GO:0005515~protein binding	26	47.27	1.30E-11	AT3G14080, AT3G15500, AT1G72770, AT4G26070, AT2G04030, AT2G26040, AT1G52890, AT1G32230, AT1G45249, AT2G26650, AT4G26080, AT5G40280, AT3G50500, AT3G59380, AT2G18960, AT2G30580, AT1G30270, AT1G78080, AT4G33950, AT4G27920, AT4G17870, AT2G40220, AT1G08720, AT1G69600, AT1G19120, AT5G05410	54	1901	18171	4.602315	9.87E-10	9.87E-10	1.37E-08
2	GO:0008270~zinc ion binding	5	50.00	9.97E-04	AT5G59550, AT3G46620, AT1G27730, AT3G56580, AT3G13672	8	1393	18171	8.152818	1.09E-02	2.74E-03	6.19E-01
2	GO:0061630~ubiquitin protein ligase activity	4	40.00	9.38E-05	AT5G59550, AT3G46620, AT3G56580, AT3G13672	8	257	18171	35.35214	1.03E-03	3.44E-04	5.84E-02
2	GO:0004842~ubiquitin-protein transferase activity	6	60.00	2.40E-07	AT5G59550, AT3G46620, AT3G52450, AT2G35930, AT3G56580, AT3G13672	8	475	18171	28.69105	2.64E-06	1.32E-06	1.50E-04

2	GO:0016874~ligase activity	6	60.00	1.18E-08	AT5G59550, AT3G46620, AT3G52450, AT2G35930, AT3G56580, AT3G13672	8	260	18171	52.41635	1.30E-07	1.30E-07	7.38E-06
3	GO:0004672~protein kinase activity	3	37.50	1.35E-02	AT1G18040, AT5G27620, AT5G64960	8	482	18171	14.13719	1.95E-01	4.26E-02	9.20E+00
3	GO:0016301~kinase activity	4	50.00	5.48E-03	AT1G18040, AT1G66750, AT5G64960, AT4G30820	8	1039	18171	8.744466	8.42E-02	2.18E-02	3.83E+00
3	GO:0005515~protein binding	7	87.50	8.30E-06	AT1G18040, AT1G03190, AT5G60410, AT5G27620, AT1G66750, AT5G64960, AT4G30820	8	1901	18171	8.363822	1.33E-04	6.64E-05	5.89E-03
3	GO:0008353~RNA polymerase II carboxy-terminal domain kinase activity	3	37.50	8.38E-06	AT1G18040, AT1G66750, AT5G64960	8	12	18171	567.8438	1.34E-04	4.47E-05	5.95E-03
3	GO:0004693~cyclin-dependent protein serine/threonine kinase activity	4	50.00	3.99E-07	AT1G18040, AT5G27620, AT1G66750, AT5G64960	8	42	18171	216.3214	6.39E-06	6.39E-06	2.84E-04
4	GO:0015204~urea transmembrane transporter activity	2	20.00	2.97E-03	AT3G16240, AT2G36830	10	6	18171	605.7	2.35E-02	5.93E-03	1.61E+00
4	GO:0005215~transporter activity	6	60.00	2.80E-07	AT3G53420, AT3G61430, AT4G35100, AT1G01620, AT2G45960, AT4G00430	10	344	18171	31.6936	2.24E-06	7.46E-07	1.53E-04
4	GO:0015250~water channel activity	10	100.00	3.57E-25	AT3G53420, AT2G37180, AT3G54820, AT3G16240, AT3G61430, AT2G36830, AT4G35100, AT1G01620, AT2G45960, AT4G00430	10	39	18171	465.9231	2.85E-24	1.43E-24	1.95E-22
4	GO:0015254~glycerol channel activity	10	100.00	1.58E-25	AT3G53420, AT2G37180, AT3G54820, AT3G16240, AT3G61430, AT2G36830, AT4G35100, AT1G01620, AT2G45960, AT4G00430	10	36	18171	504.75	1.27E-24	1.27E-24	8.68E-23

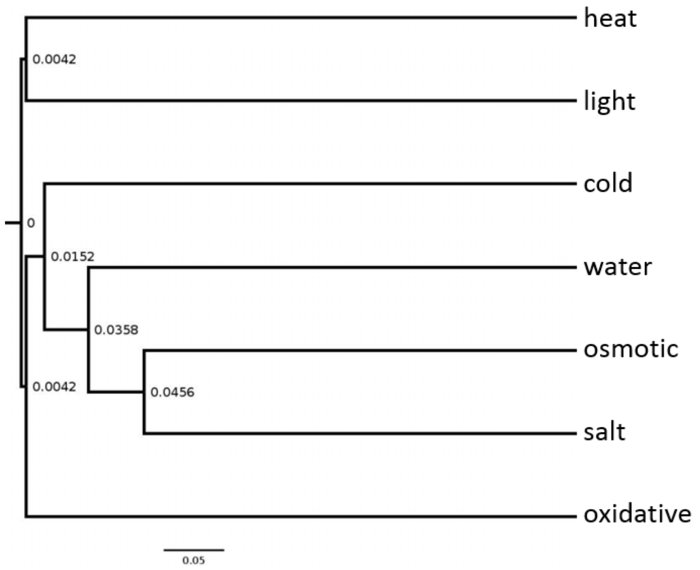

Figure S1. The similarity between sets of genes associated with different types of stress is shown in the form of a tree constructed by UPGMA using distances calculated on the basis of the Ochiai coefficients. The distance scale is shown in the lower left corner. The length of the branches is indicated next to them [Ochiai, A. (1957). Zoogeographic studies on the soleoid fishes found in Japan and its neighbouring regions. Bulletin of Japanese Society of Scientific Fisheries, 22, 526-530.]

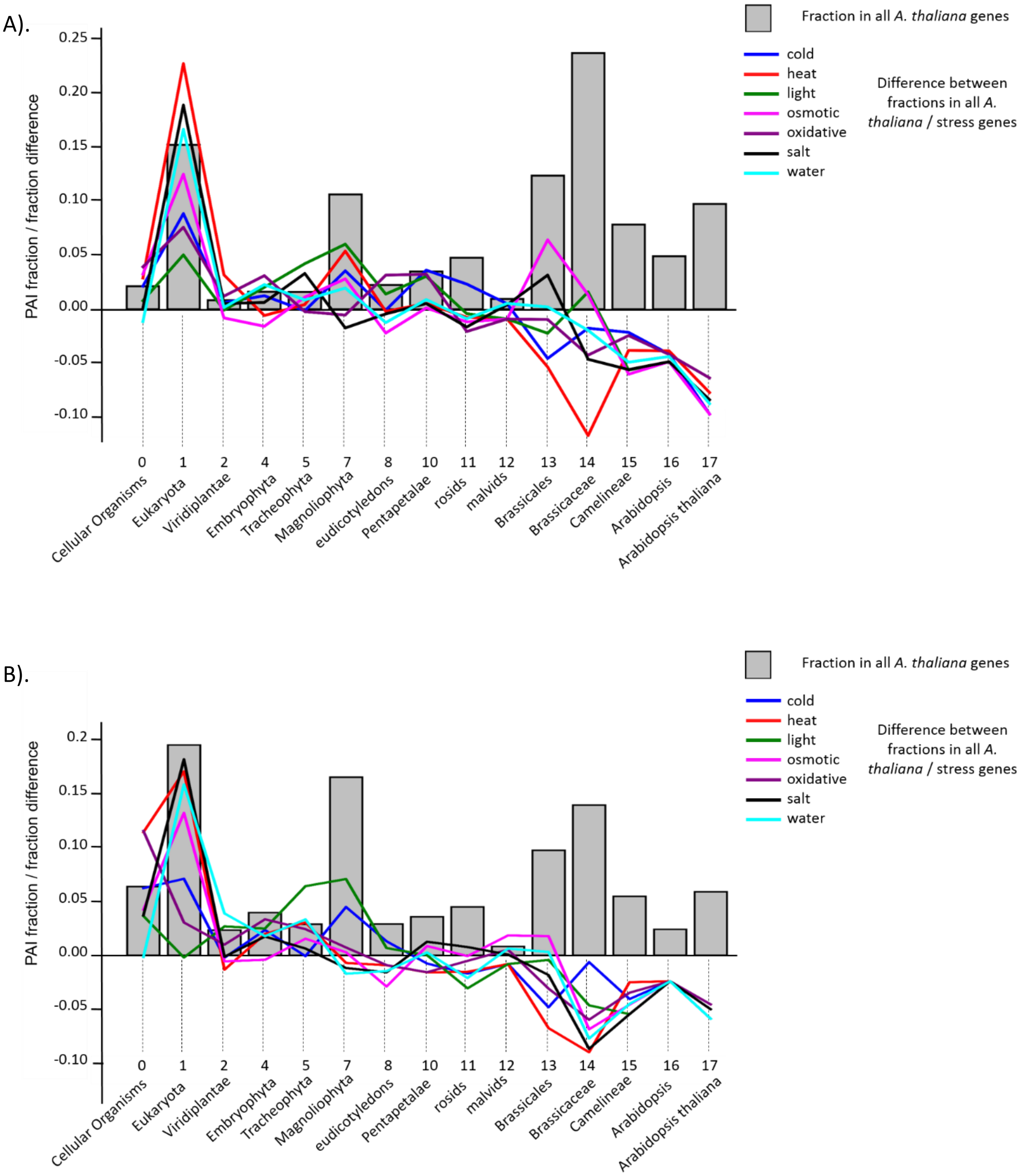

Figure S2. The distribution of frequencies of *A. thaliana* protein-coding genes (y-axis) by PAI (X-axis) is shown by grey bars. Solid lines indicate the values of the difference between the frequencies of occurrence of PAI values in stress dataset and all *A.thaliana* genes (dfPAI$_i$). Correspondence of the line color and stress type is shown in the box in the upper right corner. (A) distributions and frequency differences obtained by Orthoscape in the identification of homologues with sequence identity of 0.7; (B) distributions and frequency differences obtained by Orthoscape in the identification of homologues with sequence identity of 0.6;

A).

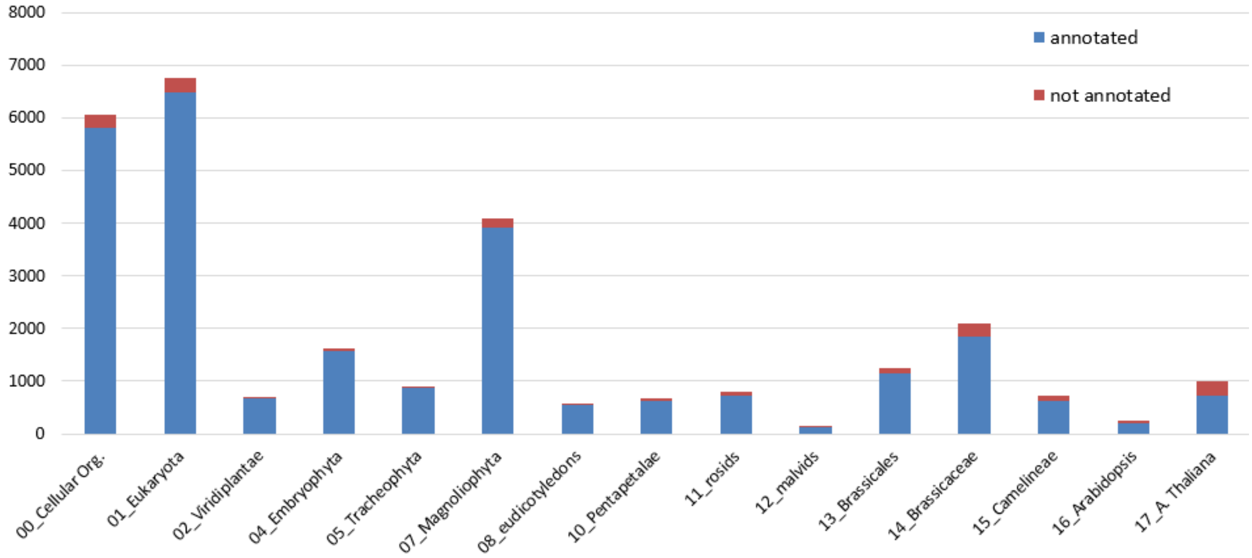

B).

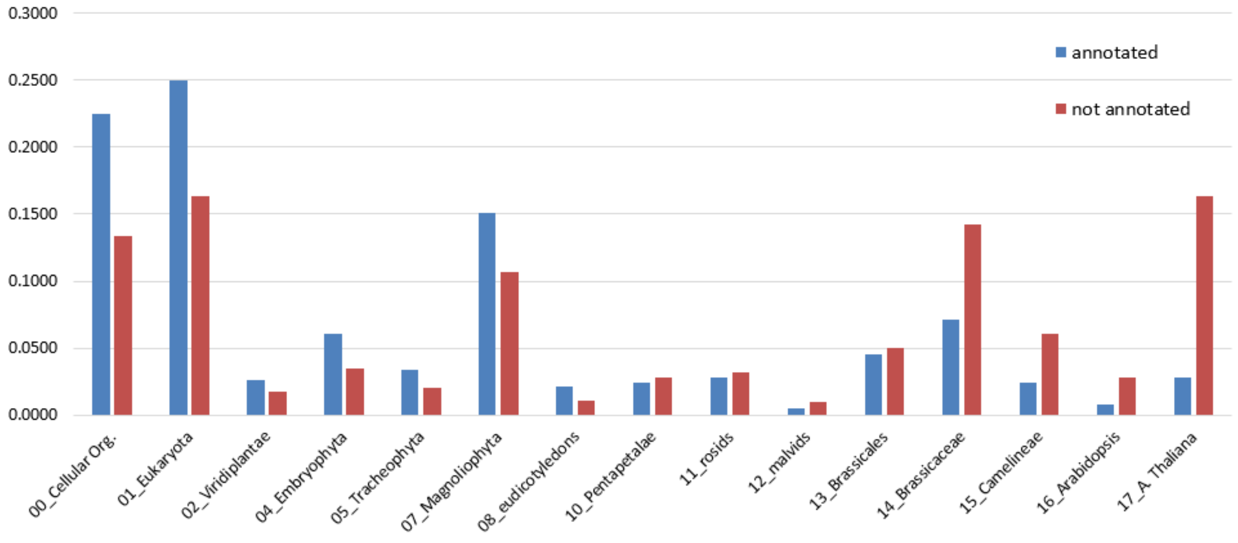

Figure S3. The PAI distribution for *A.thaliana* gene sets with and without GO annotation. A) Stacked column chart for gene numbers of GO annotated (blue bars) and non-annotated (red bars) gene numbers. B) The PAI frequency of occurrence distribution for GO annotated (blue bars) and non-annotated (red bars) genes.

Cold stress gene network

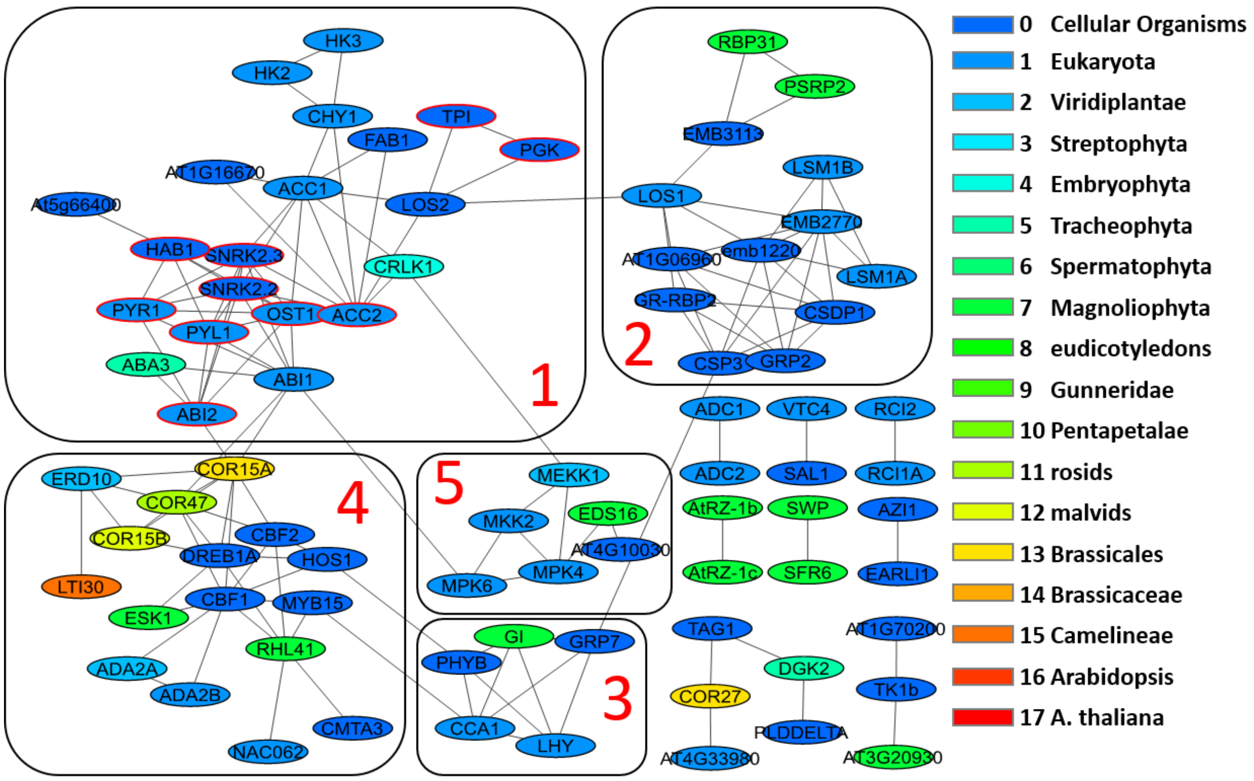

Figure S4. Gene network reconstructed for cold stress associated genes using STRING tool. Node color correspond to the PAI index of the gene is shown in the right panel. Nodes that added to the gene set by STRING procedure of network reconstruction outlined by red color. Clusters of genes shown by rectangles and numbered.

Light stress gene network

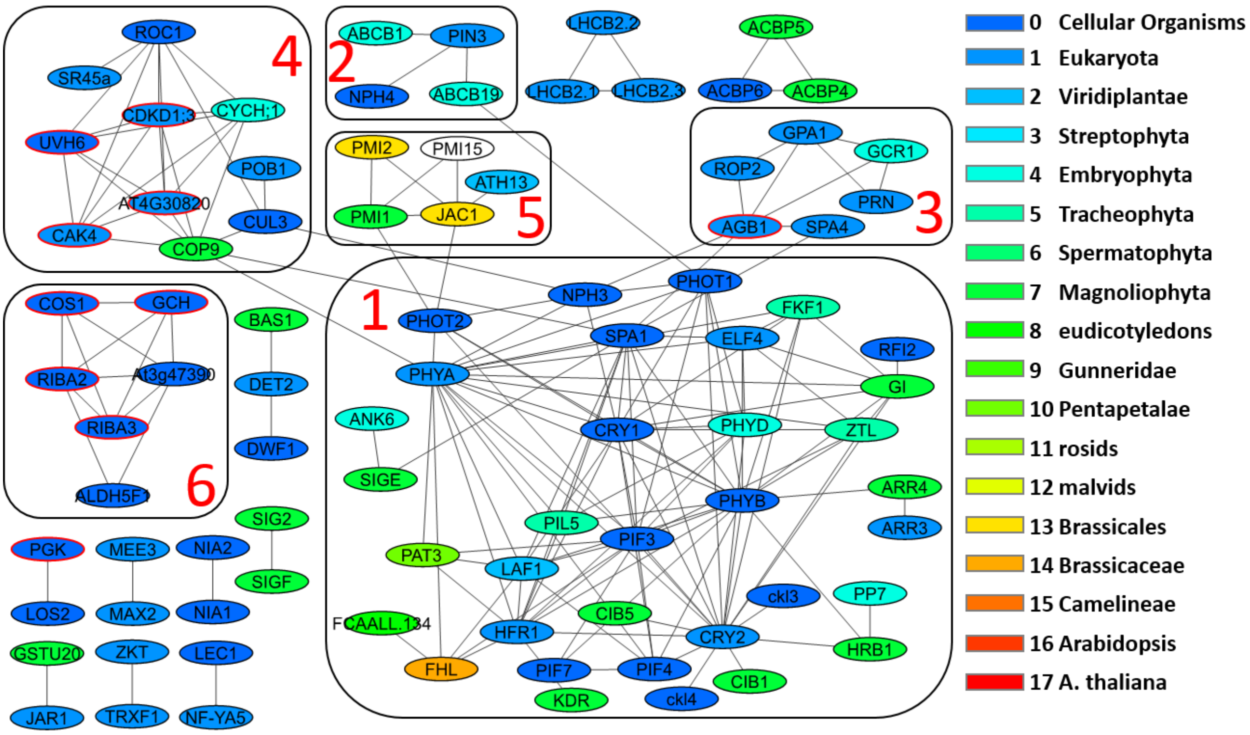

Figure S5. Gene network reconstructed for light stress associated genes using STRING tool. Node color correspond to the PAI index of the gene is shown in the right panel. Nodes that added to the gene set by STRING procedure of network reconstruction outlined by red color. Clusters of genes shown by rectangles and numbered. There are no data found for PMI15 gene.

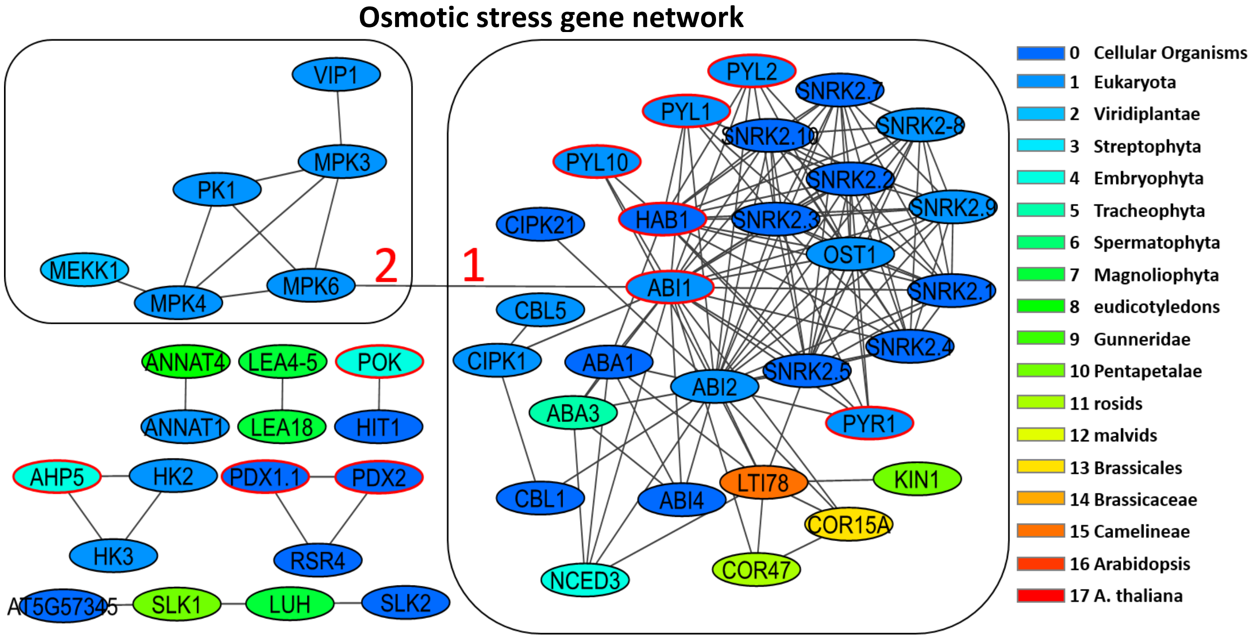

Figure S6. Gene network reconstructed for osmotic stress associated genes using STRING tool. Node color correspond to the PAI index of the gene is shown in the right panel. Nodes that added to the gene set by STRING procedure of network reconstruction outlined by red color. Clusters of genes shown by rectangles and numbered.

Oxidative stress gene network

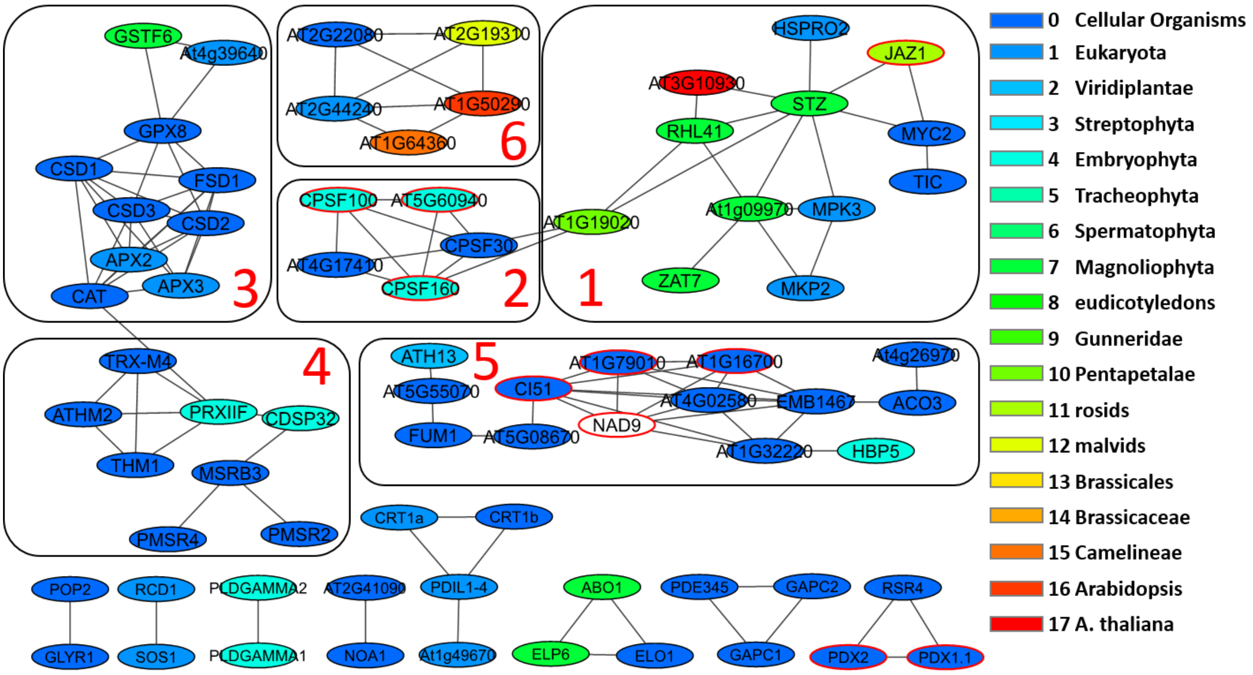

Figure S7. Gene network reconstructed for oxidative stress associated genes using STRING tool. Node color correspond to the PAI index of the gene is shown in the right panel. Nodes that added to the gene set by STRING procedure of network reconstruction outlined by red color. Clusters of genes shown by rectangles and numbered. There are no data found for NAD9 gene.

Salt stress gene network

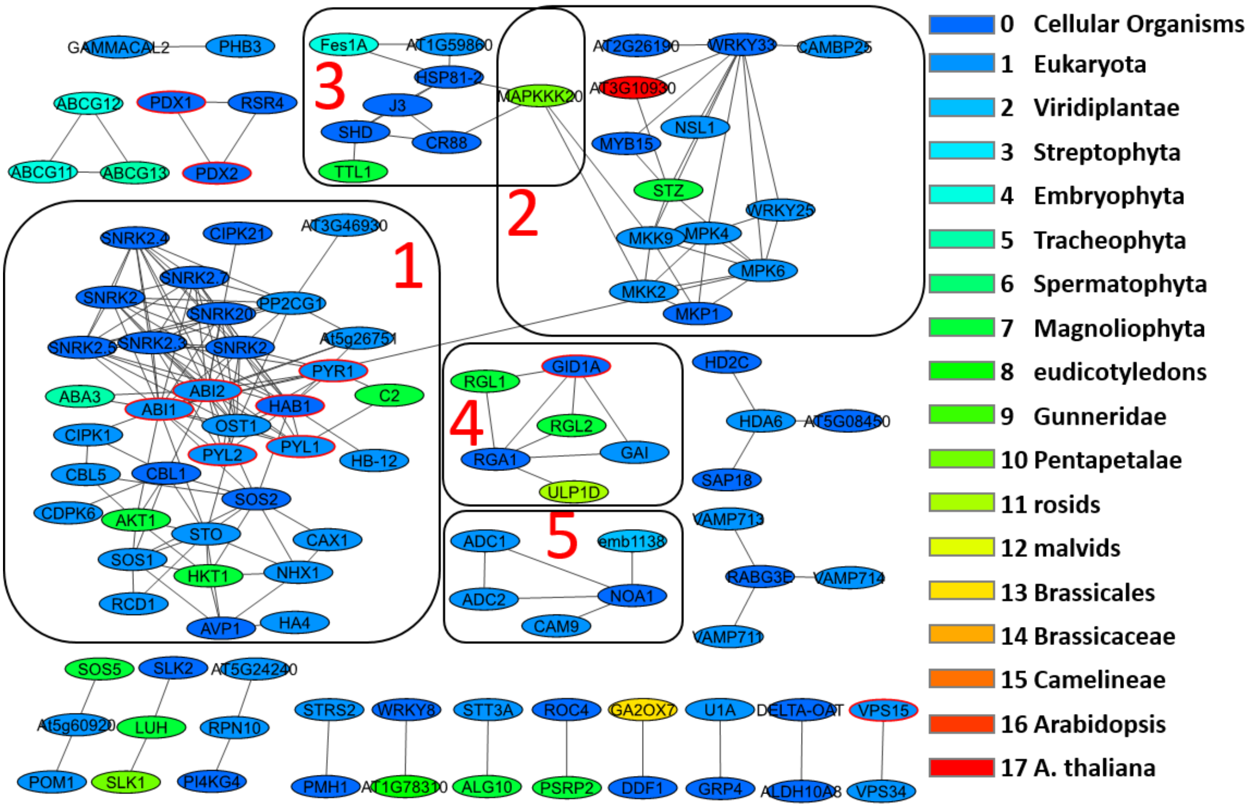

Figure S8. Gene network reconstructed for salt stress associated genes using STRING tool. Node color correspond to the PAI index of the gene is shown in the right panel. Nodes that added to the gene set by STRING procedure of network reconstruction outlined by red color. Clusters of genes shown by rectangles and numbered.

Water stress gene network

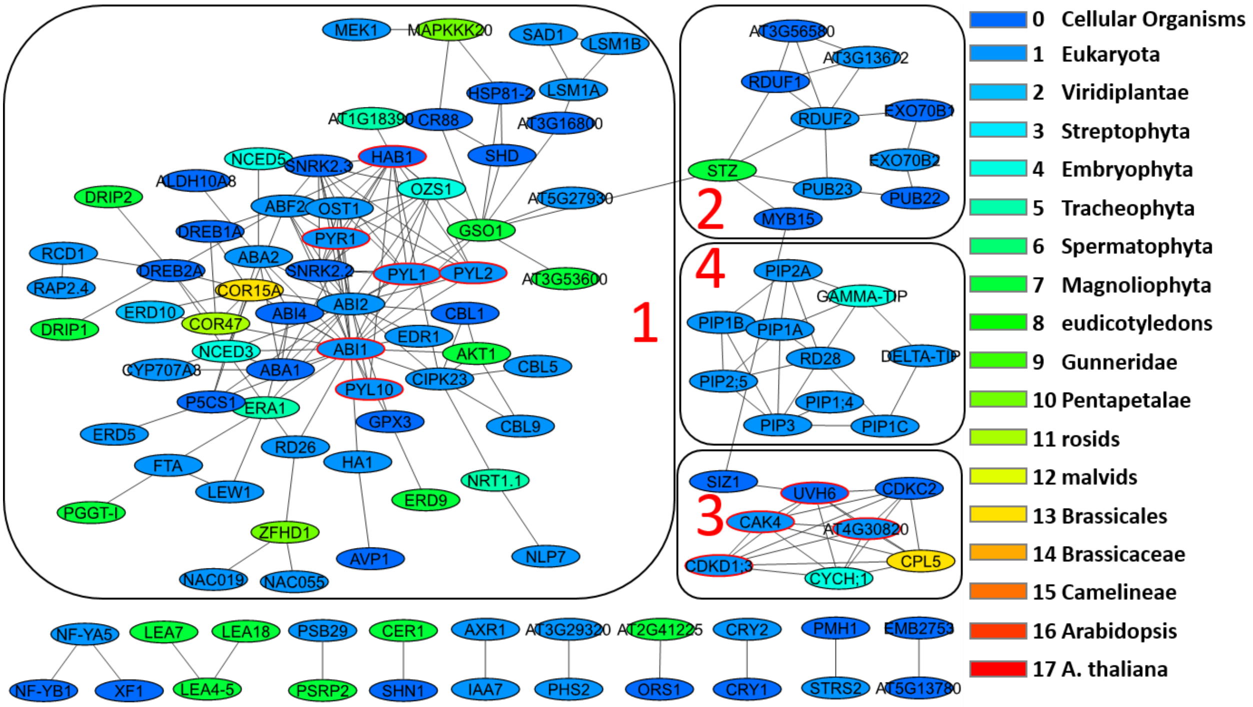

Figure S9. Gene network reconstructed for water stress associated genes using STRING tool. Node color correspond to the PAI index of the gene is shown in the right panel. Nodes that added to the gene set by STRING procedure of network reconstruction outlined by red color. Clusters of genes shown by rectangles and numbered.

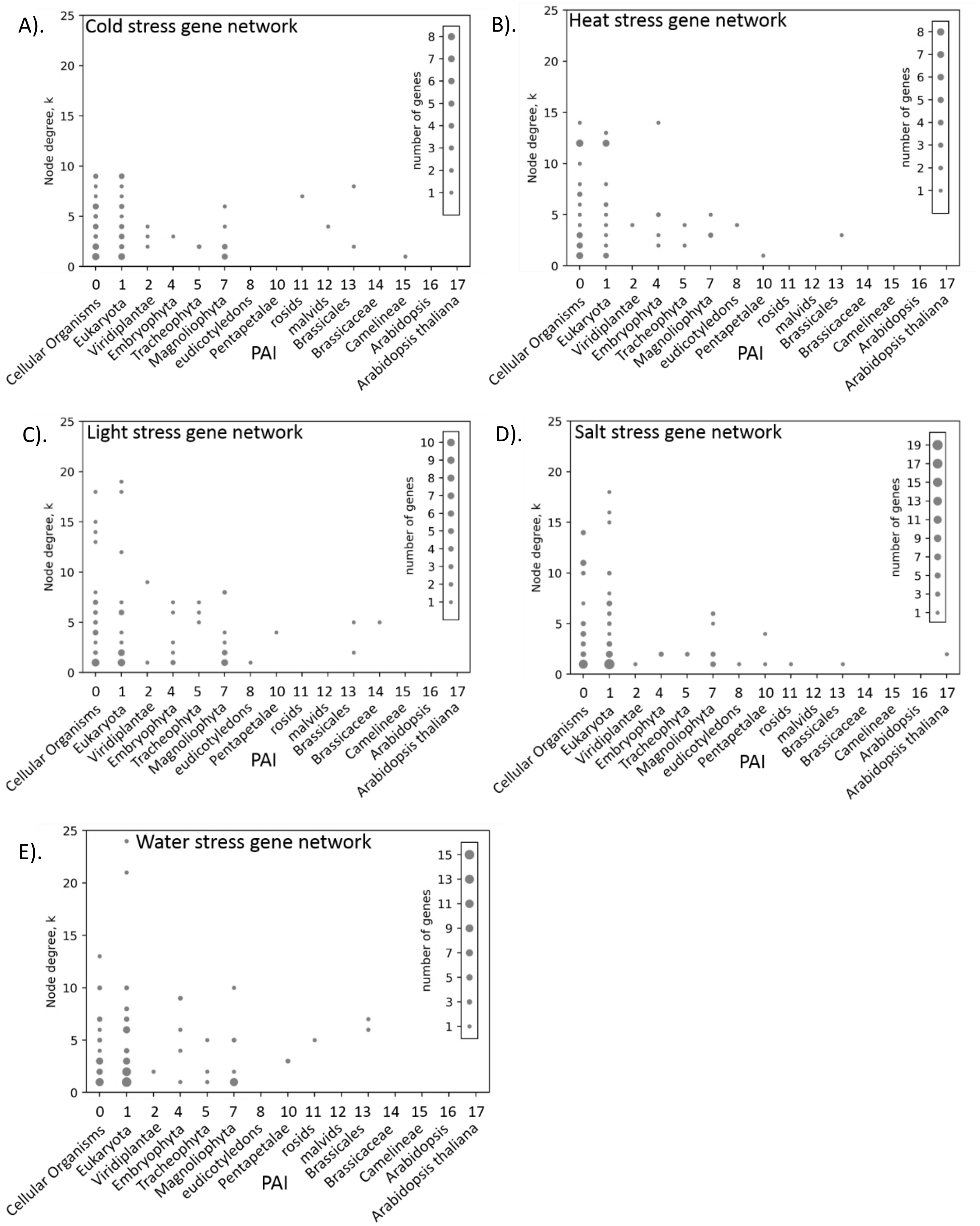

Figure S10. The PAI versus *k* scatterplots for cold (A), heat (B), light (C), salt (D), and water (E) stress gene networks. The X-axis represents the PAI, the Y-axis shows node degree *k*. Genes are indicated by circles. The size of the circle reflects the number of genes having these values *k* and PAI.

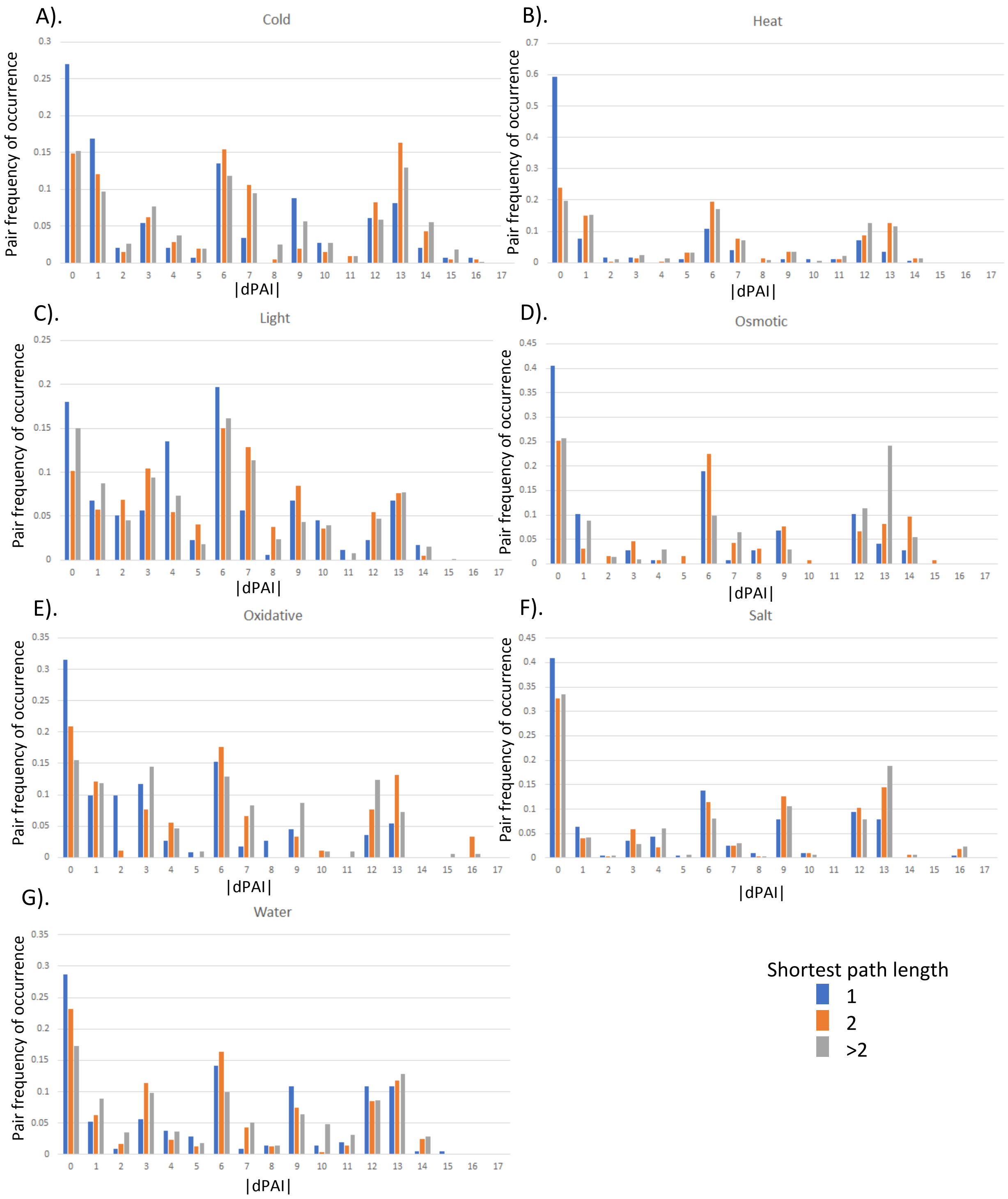

Figure S11. Distribution of |dPAI|, the absolute difference between the values of PAI in the node pairs of gene networks, separated by the different distances of the shortest path for different types of stress. The colors of the histograms for the different lengths of the shortest path between the graph vertices shown in the lower right panel of the figure.

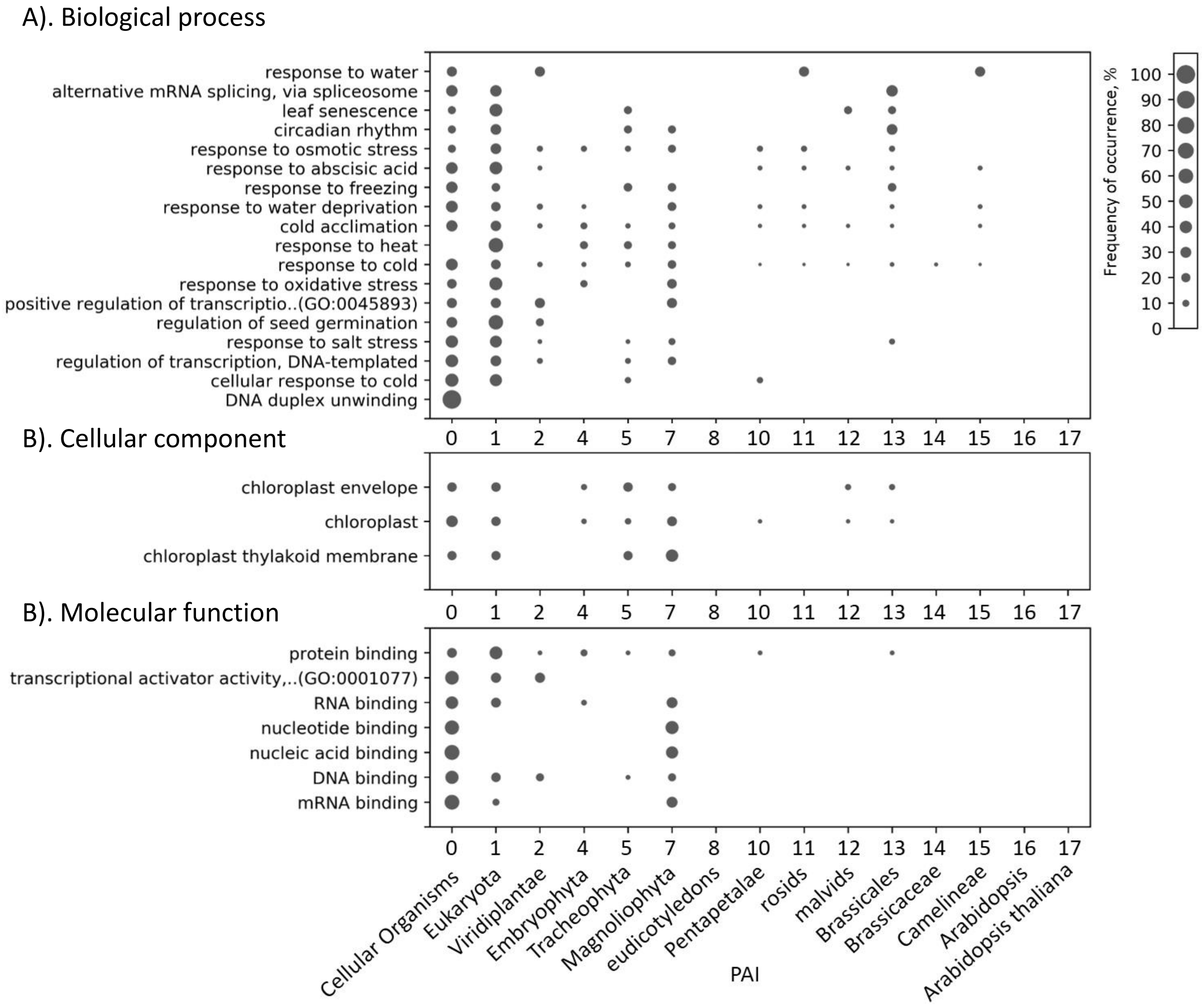

Figure S12. Distribution of genes associated with cold stress response and annotated with different GO terms by PAI values: (A) “biological process” GO terms; (B) “cellular component” GO terms; (C) “molecular function” GO terms.

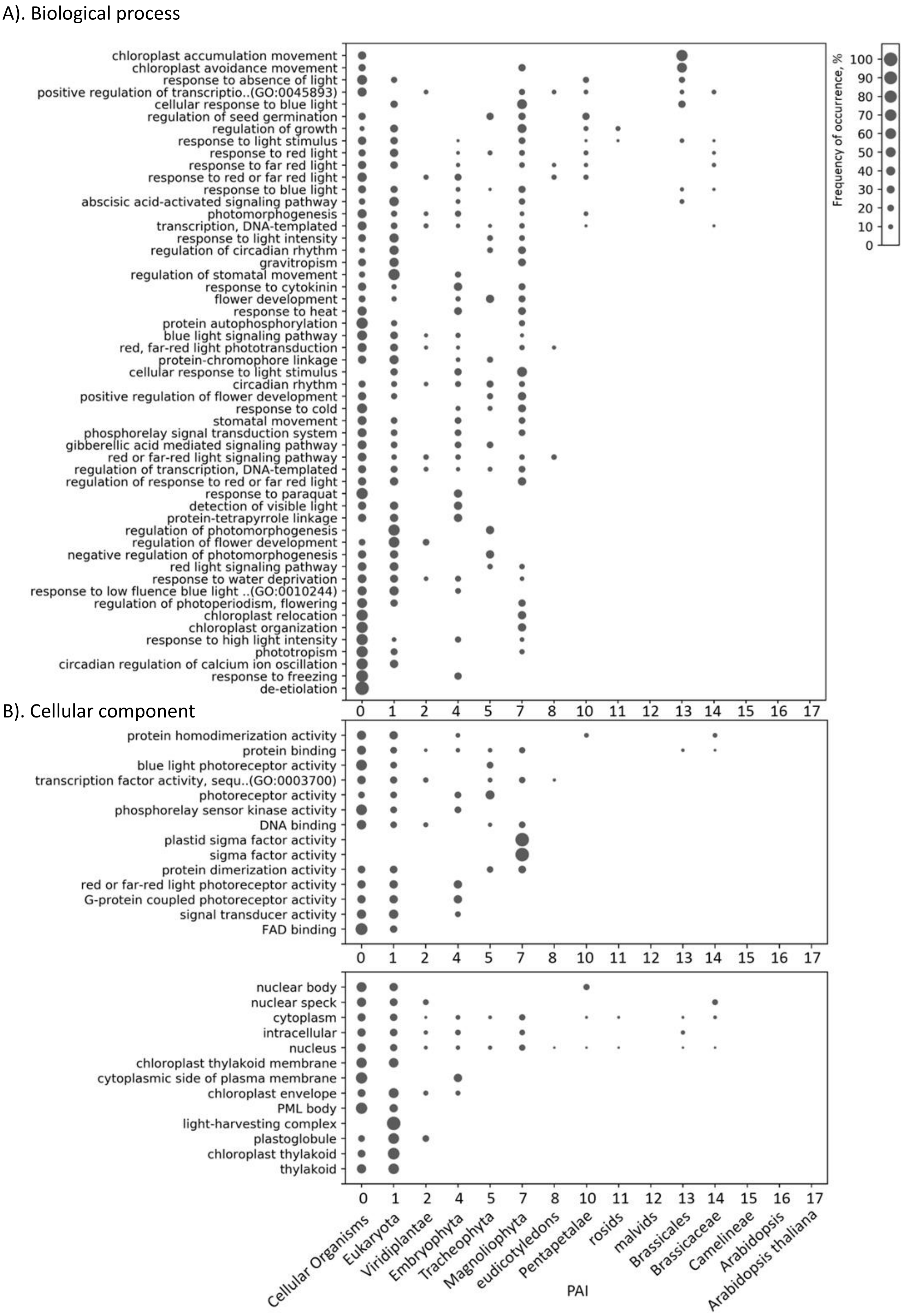

Figure S13. Distribution of genes associated with light stress response and annotated with different GO terms by PAI values: (A) "biological process" GO terms; (B) "cellular component" GO terms; (C) "molecular function" GO terms.

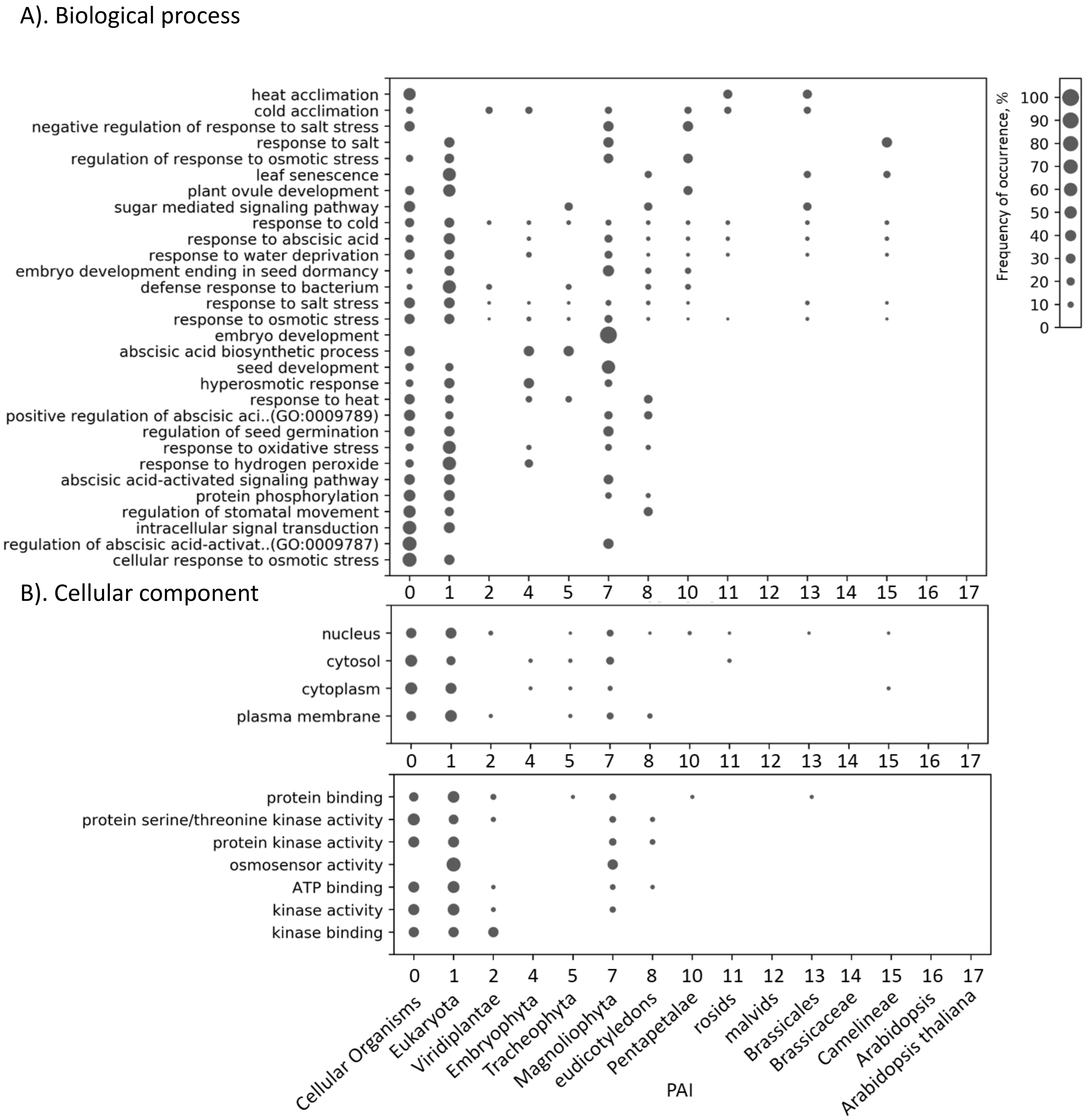

Figure S14. Distribution of genes associated with osmotic stress response and annotated with different GO terms by PAI values: (A) "biological process" GO terms; (B) "cellular component" GO terms; (C) "molecular function" GO terms.

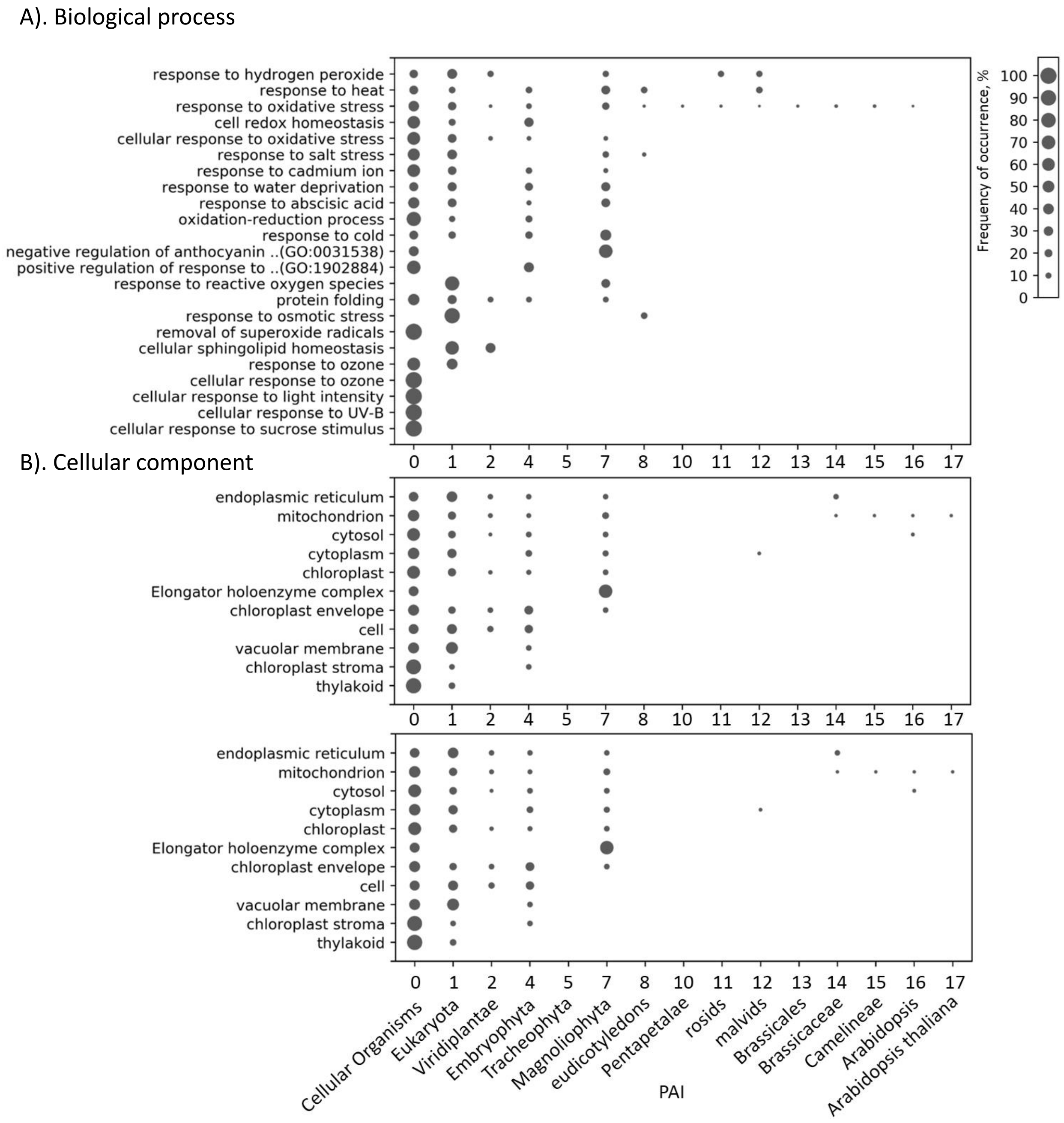

Figure S15. Distribution of genes associated with oxidative stress response and annotated with different GO terms by PAI values: (A) "biological process" GO terms; (B) "cellular component" GO terms; (C) "molecular function" GO terms.

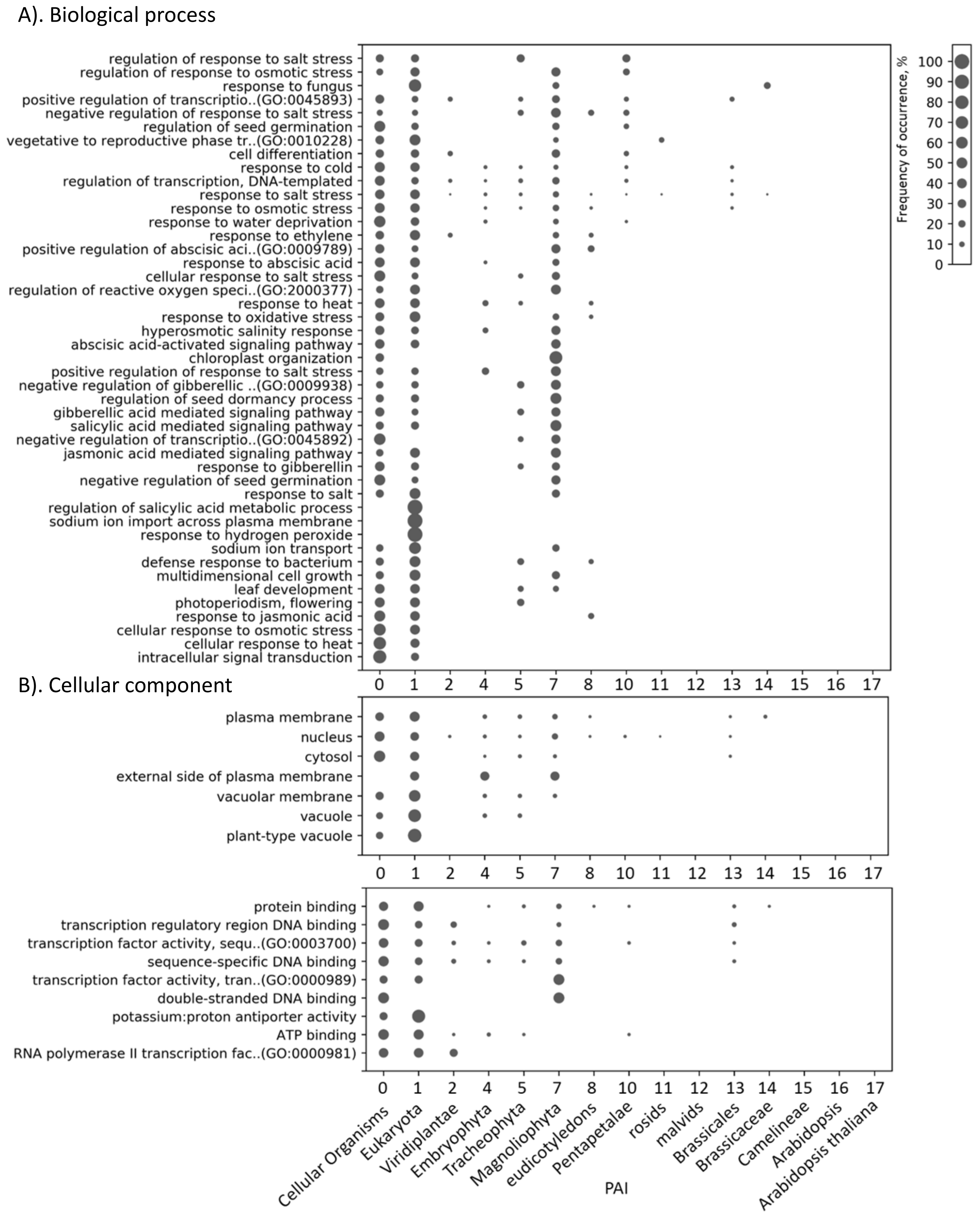

Figure S16. Distribution of genes associated with salt stress response and annotated with different GO terms by PAI values: (A) "biological process" GO terms; (B) "cellular component" GO terms; (C) "molecular function" GO terms.

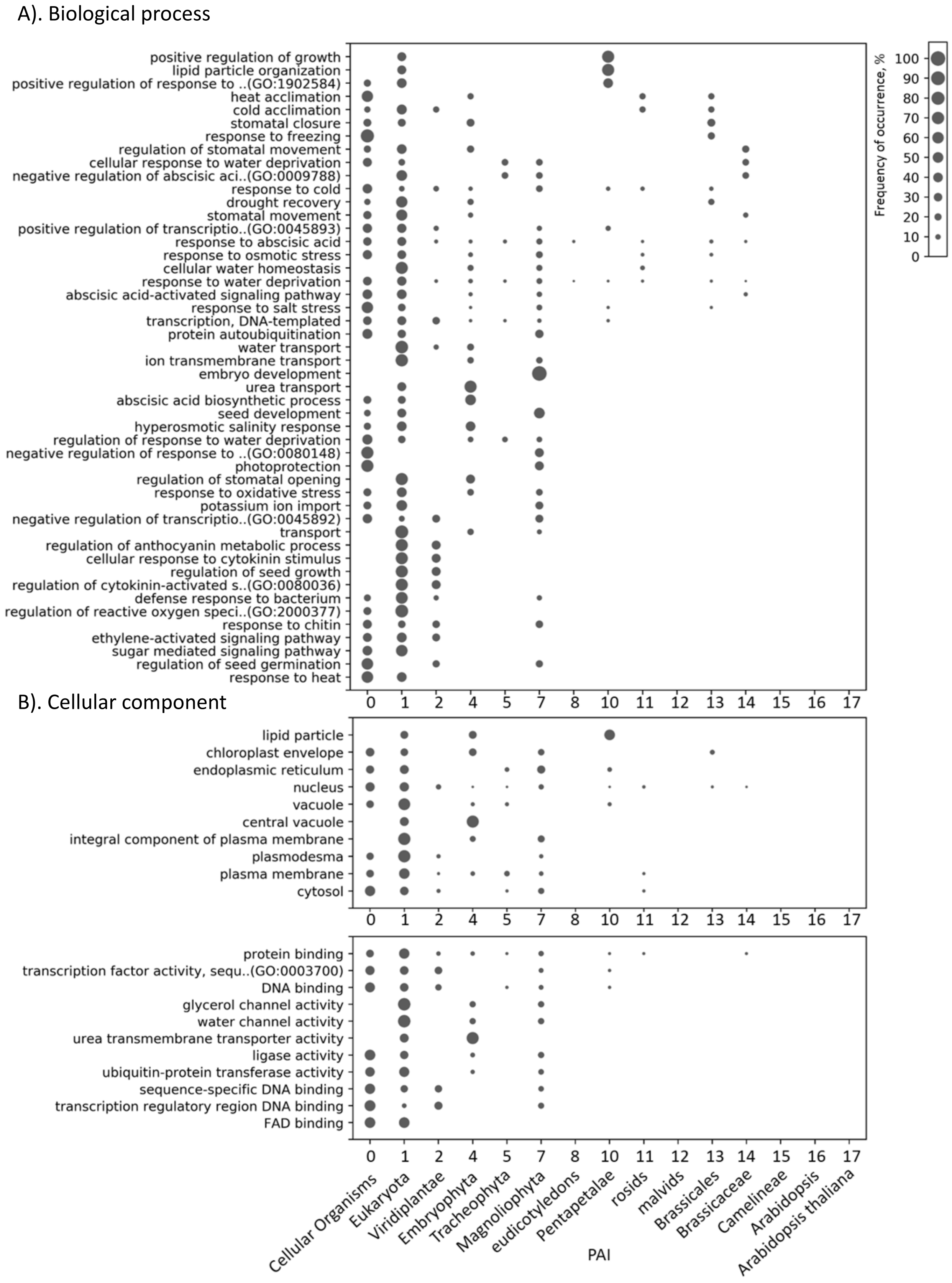

Figure S17. Distribution of genes associated with water stress response and annotated with different GO terms by PAI values: (A) "biological process" GO terms; (B) "cellular component" GO terms; (C) "molecular function" GO terms.
