# Supplementary material for: Phylostratigraphic Analysis Shows the Earliest Origination of the Abiotic Stress Associated Genes in A. thaliana
Source: Genes (Basel). 2019 Nov 22;10(12):963. doi: 10.3390/genes10120963 (PMC6947294; doi:10.3390/genes10120963)
Supplement: Supplementary file 1 [file genes-10-00963-s001.zip › Supplementary file 5_final.pdf]

Supplementary materials for Mustafin et al. “Phylostratigraphic Analysis Shows the Earliest Origination of the Abiotic Stress Associated Genes in A. thaliana”

Gene ontology terms associated with stress-associated genes obtained using DAVID server.

Columns explanation:

Stress – the name of the stress genes associated with.

Term – the ID and the name of term (separated by ~).

Count – the number of genes associated with term.

% - the proportion (in %) of genes associated with this GO compared to total number genes in cluster.

PValue - The threshold of EASE Score, a modified Fisher Exact P-Value, for gene-enrichment analysis. It ranges from 0 to 1. Fisher Exact P-Value = 0 represents perfect enrichment. Usually P-Value is equal or smaller than 0.05 to be considered strongly enriched in the annotation categories.

Genes – the list of genes associated with GO term.

List Total – the number of genes in stress dataset.

Pop Hits – how many have the function name in gene list of interest.

Pop Total – how many genes in GO dictionary (on the top of table) has that function name in the background genome.

Fold Enrichment – measure the magnitude of enrichment. Fold enrichment along with EASE score could rank the enriched terms in a more comprehensive way.

Bonferroni – one of the correction’s of the enrichment’s P-values to control family-wide false discovery rate.

Benjamini – one of the correction’s of the enrichment’s P-values to control family-wide false discovery rate.

FDR (False discovery rate) - represents the percentages of test which might be false positive.

Stress	Term	Count	%	PValue	Genes	List Total	Pop Hits	Pop Total	Fold Enrichment	Bonferroni	Benjamini	FDR
Biological process												
cold	GO:0006979~response to oxidative stress	9	6.25	1.78E-03	AT5G59820, AT5G20230, AT2G47180, AT3G12490, AT2G43790, AT1G56600, AT2G16500, AT4G34710, AT2G40880	142	291	18499	4.029113	4.73E-01	3.50E-02	2.41E+00
cold	GO:0045893~positive regulation of transcription, DNA-templated	8	5.56	1.39E-03	AT5G09410, AT3G04740, AT4G25490, AT5G64220, AT1G28520, AT1G46768, AT3G55990, AT2G46830	142	217	18499	4.802752	3.95E-01	3.09E-02	1.89E+00
cold	GO:0009408~response to heat	7	4.86	1.42E-03	AT5G59820, AT1G16540, AT3G53110, AT2G47180, AT4G26080, AT1G21760, AT1G28520	142	160	18499	5.699516	4.02E-01	2.98E-02	1.94E+00
cold	GO:0000380~alternative mRNA splicing, via spliceosome	3	2.08	8.48E-04	AT1G54380, AT4G24500, AT2G21660	142	6	18499	65.13732	2.64E-01	2.02E-02	1.16E+00
cold	GO:0006355~regulation of transcription, DNA-templated	31	21.53	5.63E-04	AT5G59820, AT2G22300, AT4G36020, AT3G02790, AT1G22770, AT5G62470, AT5G65050, AT4G25480, AT5G10450, AT4G36930, AT5G64220, AT4G38680, AT2G18790, AT2G46830, AT3G61950, AT5G09410, AT4G31120, AT2G46810, AT2G40970, AT4G31690, AT2G46590, AT1G01060, AT2G36530, AT2G17870, AT3G23250, AT4G25470, AT3G49530, AT4G04920, AT1G11760, AT4G16150, AT1G46768	142	2119	18499	1.905858	1.84E-01	1.55E-02	7.70E-01
cold	GO:0032508~DNA duplex unwinding	4	2.78	6.00E-04	AT2G21660, AT4G38680, AT4G36020, AT2G17870	142	22	18499	23.6863	1.95E-01	1.54E-02	8.22E-01
cold	GO:0009415~response to water	4	2.78	9.07E-05	AT1G20450, AT1G20440, AT5G66400, AT3G50970	142	12	18499	43.42488	3.22E-02	2.97E-03	1.25E-01
cold	GO:0007623~circadian rhythm	7	4.86	9.86E-05	AT1G22770, AT4G24500, AT4G36930, AT2G21660, AT1G01060, AT2G42540, AT2G46830	142	97	18499	9.401263	3.50E-02	2.96E-03	1.35E-01
cold	GO:0010150~leaf senescence	7	4.86	7.34E-05	AT2G22300, AT1G27320, AT2G43790, AT2G42530, AT5G35750, AT1G11760, AT2G42540	142	92	18499	9.912201	2.61E-02	2.65E-03	1.01E-01
cold	GO:0010029~regulation of seed germination	7	4.86	5.46E-07	AT1G20450, AT5G15090, AT2G19450, AT5G53470, AT1G27320, AT5G35750, AT2G18790	142	40	18499	22.79806	1.97E-04	2.19E-05	7.51E-04
cold	GO:0050826~response to freezing	6	4.17	4.42E-07	AT5G63980, AT5G09410, AT5G38480, AT3G06510, AT2G42540, AT3G55990	142	21	18499	37.22133	1.60E-04	1.99E-05	6.07E-04
cold	GO:0006970~response to osmotic stress	14	9.72	9.04E-12	AT1G16540, AT5G67590, AT2G21660, AT5G15960, AT1G20440, AT4G13850, AT1G27320, AT2G43790, AT5G35750,	142	122	18499	14.94955	3.26E-09	4.66E-10	1.24E-08

					AT4G04920, AT4G34710, AT4G08500, AT2G42540, AT5G06760							
cold	GO:0009737~response to abscisic acid	26	18.06	2.60E-16	AT1G20450, AT3G53110, AT5G15960, AT2G47180, AT2G19450, AT1G56600, AT2G42530, AT3G05890, AT5G62470, AT4G26080, AT5G53470, AT4G38680, AT3G50970, AT2G46830, AT1G20440, AT5G66400, AT1G01060, AT1G29395, AT4G34710, AT2G42540, AT2G36530, AT4G12480, AT4G01370, AT3G05880, AT2G43790, AT5G35750	142	394	18499	8.596804	8.02E-14	1.33E-14	3.00E-13
cold	GO:0009651~response to salt stress	30	20.83	3.72E-18	AT2G47180, AT2G19450, AT4G13850, AT1G27320, AT2G16500, AT1G56600, AT4G36020, AT5G63980, AT5G62470, AT3G05890, AT2G21660, AT4G29810, AT5G50950, AT2G46830, AT1G16540, AT4G24500, AT2G38170, AT5G04280, AT1G01060, AT4G34710, AT2G42540, AT2G36530, AT4G12480, AT4G01370, AT1G60650, AT3G23250, AT2G43790, AT5G35750, AT4G08500, AT3G55990	142	484	18499	8.074875	1.34E-15	2.69E-16	5.11E-15
cold	GO:0070417~cellular response to cold	13	9.03	2.95E-18	AT1G54380, AT4G12470, AT5G20230, AT3G61580, AT2G46210, AT5G62390, AT1G20823, AT1G60170, AT1G27320, AT1G29395, AT5G35750, AT3G49530, AT1G29390	142	31	18499	54.6313	1.06E-15	2.66E-16	4.05E-15
cold	GO:0009414~response to water deprivation	28	19.44	2.60E-22	AT1G20450, AT3G14080, AT5G15960, AT2G47180, AT4G13850, AT1G27320, AT1G56600, AT1G28520, AT4G36020, AT2G40880, AT5G63980, AT5G62470, AT4G25480, AT2G21660, AT4G38680, AT3G50970, AT5G04280, AT1G20440, AT5G66400, AT2G42540, AT5G06760, AT1G60650, AT4G25490, AT3G12490, AT1G19120, AT5G35750, AT3G55990, AT1G46768	142	279	18499	13.07416	9.40E-20	3.13E-20	3.58E-19
cold	GO:0009631~cold acclimation	41	28.47	1.94E-77	AT1G20450, AT5G59820, AT3G14080, AT5G54590, AT5G15960, AT4G13850, AT2G42530, AT1G28520, AT4G36020, AT3G53460, AT4G12470, AT1G74960, AT4G25480, AT5G38480, AT4G29810, AT1G56070, AT1G77490, AT4G38680, AT3G50970, AT3G26420, AT4G16420, AT3G55580, AT2G38170, AT3G04740, AT1G10760, AT1G20440, AT5G66400, AT1G29395, AT2G42540, AT1G29390, AT2G17870, AT5G67590, AT4G25470, AT4G04800, AT4G25490, AT1G36160, AT1G19120, AT4G24770, AT4G04920, AT1G11760, AT4G08500	142	51	18499	104.7306	7.01E-75	3.50E-75	2.67E-74
cold	GO:0009409~response to cold	96	66.67	8.18E-141	AT1G20450, AT5G63770, AT3G02870, AT3G08920, AT4G10030, AT5G15960, AT2G22300, AT4G13850, AT3G21300, AT1G60170, AT3G07740, AT4G36020, AT5G65940, AT2G40880, AT1G22770, AT5G63980, AT3G11170, AT4G25480, AT4G36930, AT5G52440, AT1G56070, AT4G03430, AT5G53470, AT4G38680, AT3G50970, AT2G18790, AT1G16540, AT2G40970, AT4G24500, AT2G46590, AT4G31690, AT3G22370, AT2G36530, AT2G17870, AT1G60650, AT5G15090, AT4G25490, AT5G42900, AT3G12490, AT1G21760, AT2G43790, AT1G33410, AT4G24770, AT4G08500, AT2G01918, AT1G46768, AT5G59820, AT2G39810, AT5G54590, AT3G53110, AT2G33800, AT4G28210, AT2G47180, AT1G01860, AT4G35790, AT2G19450, AT2G20990, AT1G27320, AT1G70200, AT2G42530, AT2G16500, AT1G56600, AT2G46090, AT3G53460, AT2G37230, AT1G05140, AT3G05890, AT5G17890, AT2G21660, AT4G29810, AT5G38480, AT5G67320, AT4G26080, AT1G74710, AT5G64220, AT3G26420, AT5G52370, AT2G46830, AT5G09410, AT1G17610, AT5G04280, AT1G20440, AT5G23070, AT1G01060, AT3G06510, AT4G34710, AT2G42540, AT5G06760, AT4G12480, AT4G01370, AT4G25470, AT3G05880, AT4G19330, AT3G22690, AT3G20930, AT3G55990	142	299	18499	41.82731	2.95E-138	2.95E-138	1.12E-137
heat	GO:0043248~proteasome assembly	3	2.94	3.38E-03	AT4G29040, AT1G64520, AT4G38630	96	17	18499	34.00551	6.87E-01	4.37E-02	4.51E+00
heat	GO:0072593~reactive oxygen species metabolic process	3	2.94	2.99E-03	AT4G04950, AT1G79440, AT5G47910	96	16	18499	36.13086	6.43E-01	4.03E-02	4.00E+00
heat	GO:0009688~abscisic acid biosynthetic process	3	2.94	2.62E-03	AT1G16540, AT5G67030, AT1G52340	96	15	18499	38.53958	5.95E-01	3.85E-02	3.52E+00
heat	GO:0050832~defense response to fungus	9	8.82	2.67E-03	AT4G11260, AT2G26330, AT4G23100, AT5G42980, AT5G47910, AT2G38470, AT5G03280, AT1G64280, AT5G02500	96	464	18499	3.737675	6.01E-01	3.76E-02	3.58E+00
heat	GO:0009873~ethylene-activated signaling pathway	6	5.88	2.30E-03	AT1G66340, AT2G20880, AT1G54490, AT5G03280, AT1G05850, AT3G16770	96	179	18499	6.459148	5.48E-01	3.54E-02	3.10E+00
heat	GO:0008219~cell death	4	3.92	1.49E-03	AT5G43940, AT5G03280, AT1G64280, AT3G16770	96	44	18499	17.51799	4.02E-01	2.42E-02	2.02E+00
heat	GO:0061077~chaperone-mediated protein folding	4	3.92	5.84E-04	AT3G53990, AT3G25230, AT5G48570, AT2G42540	96	32	18499	24.08724	1.82E-01	1.00E-02	7.94E-01

heat	GO:0051259~protein oligomerization	3	2.94	3.86E-04	AT3G17880, AT5G42980, AT2G42540	96	6	18499	96.34896	1.24E-01	6.97E-03	5.26E-01
heat	GO:0001944~vasculature development	4	3.92	3.13E-04	AT1G66340, AT2G26330, AT1G54490, AT5G03280	96	26	18499	29.64583	1.02E-01	5.97E-03	4.26E-01
heat	GO:0050826~response to freezing	4	3.92	1.63E-04	AT5G60410, AT5G58070, AT1G12610, AT2G42540	96	21	18499	36.70437	5.46E-02	3.30E-03	2.22E-01
heat	GO:0042742~defense response to bacterium	9	8.82	8.79E-05	AT1G66340, AT1G16540, AT2G26330, AT4G23100, AT4G26850, AT2G39770, AT2G38470, AT5G03280, AT5G02500	96	276	18499	6.283628	2.98E-02	1.89E-03	1.20E-01
heat	GO:0051788~response to misfolded protein	3	2.94	7.80E-05	AT4G29040, AT1G64520, AT4G38630	96	3	18499	192.6979	2.65E-02	1.79E-03	1.06E-01
heat	GO:0052544~defense response by callose deposition in cell wall	4	3.92	7.00E-05	AT1G66340, AT4G23100, AT4G26850, AT5G03280	96	16	18499	48.17448	2.38E-02	1.72E-03	9.54E-02
heat	GO:0006402~mRNA catabolic process	4	3.92	7.00E-05	AT5G21160, AT5G61780, AT1G54490, AT5G07350	96	16	18499	48.17448	2.38E-02	1.72E-03	9.54E-02
heat	GO:0010182~sugar mediated signaling pathway	5	4.90	1.61E-05	AT1G66340, AT1G16540, AT5G67030, AT5G03280, AT1G52340	96	30	18499	32.11632	5.54E-03	4.27E-04	2.20E-02
heat	GO:0042542~response to hydrogen peroxide	6	5.88	8.32E-06	AT2G26150, AT4G27670, AT5G42980, AT1G74310, AT5G05410, AT4G12400	96	54	18499	21.41088	2.86E-03	2.39E-04	1.14E-02
heat	GO:0009735~response to cytokinin	9	8.82	4.59E-06	AT5G42020, AT5G53400, AT5G42980, AT5G58070, AT1G64520, AT4G38630, AT3G25230, AT1G05850, AT3G16770	96	183	18499	9.476947	1.58E-03	1.44E-04	6.27E-03
heat	GO:0009409~response to cold	12	11.76	3.83E-07	AT5G59820, AT1G16540, AT3G53110, AT3G53990, AT5G58070, AT2G30250, AT4G26080, AT2G38470, AT1G21760, AT2G03440, AT2G42540, AT5G02500	96	299	18499	7.733696	1.32E-04	1.32E-05	5.23E-04
heat	GO:0006457~protein folding	12	11.76	2.64E-07	AT5G56010, AT5G42020, AT4G26780, AT5G53400, AT5G62390, AT3G17880, AT1G09080, AT5G42980, AT3G08970, AT3G25230, AT1G56260, AT5G02500	96	288	18499	8.02908	9.07E-05	1.01E-05	3.60E-04
heat	GO:0009651~response to salt stress	16	15.69	2.40E-08	AT1G16540, AT2G20880, AT1G12610, AT2G30250, AT3G06010, AT2G03440, AT5G03280, AT2G42540, AT3G09350, AT1G66340, AT5G61780, AT2G39770, AT2G38470, AT5G07350, AT4G38630, AT1G05850	96	484	18499	6.370179	8.27E-06	1.03E-06	3.28E-05
heat	GO:0009644~response to high light intensity	8	7.84	1.25E-08	AT2G26150, AT4G27670, AT5G58070, AT1G74310, AT3G47860, AT5G53170, AT2G42540, AT4G12400	96	55	18499	28.02879	4.30E-06	7.16E-07	1.70E-05
heat	GO:0006970~response to osmotic stress	10	9.80	1.28E-08	AT5G57050, AT1G16540, AT1G50500, AT2G30250, AT5G67030, AT2G38470, AT5G53060, AT5G03280, AT3G25230, AT2G42540	96	122	18499	15.79491	4.40E-06	6.28E-07	1.74E-05
heat	GO:0009414~response to water deprivation	14	13.73	1.83E-09	AT1G12610, AT3G06010, AT1G28520, AT2G42540, AT5G57050, AT5G60410, AT5G44650, AT5G58070, AT5G67030, AT3G47860, AT2G38470, AT1G05850, AT5G05410, AT1G52340	96	279	18499	9.66943	6.30E-07	1.26E-07	2.50E-06
heat	GO:0034605~cellular response to heat	10	9.80	4.86E-15	AT2G26150, AT5G62390, AT2G30250, AT4G19020, AT5G61780, AT3G08970, AT2G38470, AT5G07350, AT5G27660, AT5G07100	96	26	18499	74.11458	1.68E-12	4.20E-13	6.66E-12
heat	GO:0070370~cellular heat acclimation	10	9.80	1.68E-20	AT5G21160, AT2G26330, AT2G30250, AT2G38470, AT4G15802, AT2G21320, AT1G54490, AT3G25230, AT5G48570, AT5G07100	96	10	18499	192.6979	5.77E-18	1.92E-18	2.29E-17
heat	GO:0010286~heat acclimation	24	23.53	1.79E-44	AT4G26840, AT3G02885, AT5G43940, AT1G54490, AT1G79350, AT2G42540, AT4G12400, AT5G21160, AT2G26150, AT4G26780, AT5G60410, AT1G50500, AT5G53400, AT3G17880, AT5G44650, AT4G21320, AT5G58070, AT5G42980, AT3G47220, AT5G53060, AT5G05410, AT5G10010, AT2G41140, AT3G16770	96	38	18499	121.7039	6.16E-42	3.08E-42	2.44E-41
heat	GO:0009408~response to heat	65	63.73	3.57E-114	AT4G27670, AT4G11260, AT4G29770, AT5G47910, AT4G26850, AT5G63870, AT1G64280, AT4G04950, AT3G09350, AT1G66340, AT1G03190, AT3G10140, AT3G53990, AT5G42980, AT3G08970, AT1G09090, AT5G16820, AT1G16540, AT3G02885, AT1G12610, AT4G26780, AT5G57050, AT1G79440, AT1G50500, AT4G21320, AT5G58070, AT1G08550, AT4G15802, AT1G21760, AT4G38630, AT5G05410, AT1G71790, AT1G52340, AT5G59820, AT3G24320, AT3G53110, AT3G28030, AT1G64520, AT2G03440, AT1G28520, AT3G25230, AT5G03280, AT4G12400, AT2G26150, AT4G29040, AT4G26080, AT3G47860, AT2G38470, AT1G05850, AT5G53170, AT5G02500, AT5G56010, AT4G26840, AT5G17020, AT3G06010, AT5G42020, AT4G23100, AT5G53400, AT1G09080, AT1G74310, AT5G05630, AT5G67030, AT2G39770, AT3G10800, AT1G56260	96	160	18499	78.28353	1.23E-111	1.23E-111	4.87E-111
light	GO:0046777~protein autophosphorylation	6	4.23	3.56E-03	AT1G10470, AT4G08920, AT3G45780, AT4G28860, AT5G58140, AT1G04400	140	135	18499	5.872698	7.70E-01	2.96E-02	4.88E+00
light	GO:0010029~regulation of seed germination	4	2.82	3.35E-03	AT2G37678, AT2G20180, AT1G42550, AT2G18790	140	40	18499	13.21357	7.49E-01	2.84E-02	4.59E+00
light	GO:0010099~regulation of photomorphogenesis	3	2.11	2.95E-03	AT2G21150, AT2G20180, AT2G24790	140	11	18499	36.03701	7.04E-01	2.56E-02	4.05E+00
light	GO:0010100~negative regulation of photomorphogenesis	3	2.11	2.95E-03	AT2G20180, AT2G21320, AT2G46340	140	11	18499	36.03701	7.04E-01	2.56E-02	4.05E+00
light	GO:0009658~chloroplast organization	6	4.23	2.54E-03	AT5G24120, AT3G47390, AT4G22260, AT1G08540, AT2G46340, AT5G55280	140	125	18499	6.342514	6.50E-01	2.25E-02	3.50E+00

light	GO:0009909~regulation of flower development	5	3.52	2.34E-03	AT3G21150, AT2G24790, AT1G25540, AT1G04400, AT2G40080	140	74	18499	8.928089	6.19E-01	2.12E-02	3.23E+00
light	GO:0009740~gibberellic acid mediated signaling pathway	5	3.52	2.22E-03	AT5G39760, AT2G26300, AT2G20180, AT1G48270, AT1G09530	140	73	18499	9.050391	6.01E-01	2.06E-02	3.07E+00
light	GO:0009408~response to heat	7	4.93	1.32E-03	AT5G59820, AT4G27670, AT1G79440, AT5G58070, AT3G47860, AT5G58140, AT5G63870	140	160	18499	5.780938	4.21E-01	1.26E-02	1.84E+00
light	GO:0009902~chloroplast relocation	3	2.11	8.24E-04	AT1G42550, AT5G58140, AT5G55280	140	6	18499	66.06786	2.88E-01	8.06E-03	1.15E+00
light	GO:0000160~phosphorelay signal transduction system	5	3.52	5.80E-04	AT1G10470, AT3G45780, AT3G48100, AT1G59940, AT5G58140	140	51	18499	12.95448	2.13E-01	5.81E-03	8.10E-01
light	GO:0017006~protein-tetrapyrrole linkage	3	2.11	5.52E-04	AT4G16250, AT2G18790, AT1G09570	140	5	18499	79.28143	2.04E-01	5.67E-03	7.71E-01
light	GO:0009584~detection of visible light	3	2.11	5.52E-04	AT4G16250, AT2G18790, AT1G09570	140	5	18499	79.28143	2.04E-01	5.67E-03	7.71E-01
light	GO:0050826~response to freezing	4	2.82	5.00E-04	AT5G63980, AT1G31812, AT5G58070, AT5G58140	140	21	18499	25.16871	1.86E-01	5.27E-03	6.98E-01
light	GO:0009904~chloroplast accumulation movement	4	2.82	4.31E-04	AT3G45780, AT5G38150, AT1G75100, AT1G66840	140	20	18499	26.42714	1.63E-01	4.79E-03	6.02E-01
light	GO:2000028~regulation of photoperiodism, flowering	4	2.82	4.31E-04	AT1G22770, AT2G46340, AT2G18790, AT1G04400	140	20	18499	26.42714	1.63E-01	4.79E-03	6.02E-01
light	GO:0009409~response to cold	10	7.04	4.37E-04	AT1G22770, AT5G63980, AT5G59820, AT1G31812, AT2G46590, AT4G36930, AT5G58070, AT3G22690, AT2G18790, AT2G36530	140	299	18499	4.419255	1.65E-01	4.73E-03	6.10E-01
light	GO:1901562~response to paraquat	3	2.11	3.33E-04	AT5G58070, AT3G47860, AT5G58140	140	4	18499	99.10179	1.28E-01	3.80E-03	4.65E-01
light	GO:0010617~circadian regulation of calcium ion oscillation	3	2.11	1.67E-04	AT4G08920, AT2G18790, AT1G04400	140	3	18499	132.1357	6.66E-02	1.97E-03	2.34E-01
light	GO:2000030~regulation of response to red or far red light	3	2.11	1.67E-04	AT2G46370, AT1G78370, AT4G04770	140	3	18499	132.1357	6.66E-02	1.97E-03	2.34E-01
light	GO:0071482~cellular response to light stimulus	4	2.82	1.42E-04	AT3G08550, AT1G08540, AT2G30170, AT2G36990	140	14	18499	37.75306	5.69E-02	1.77E-03	1.99E-01
light	GO:0009908~flower development	9	6.34	1.46E-04	AT1G22770, AT1G68050, AT5G57360, AT4G36930, AT1G48270, AT3G54610, AT1G25540, AT4G34530, AT2G40080	140	202	18499	5.887235	5.84E-02	1.77E-03	2.04E-01
light	GO:0009735~response to cytokinin	9	6.34	7.36E-05	AT1G10470, AT3G62030, AT4G38740, AT5G58070, AT3G48100, AT1G59940, AT1G48270, AT1G78370, AT5G58140	140	183	18499	6.498478	2.99E-02	9.47E-04	1.03E-01
light	GO:0009704~de-etiolation	4	2.82	6.55E-05	AT4G38740, AT5G61270, AT2G43010, AT1G09530	140	11	18499	48.04935	2.66E-02	8.71E-04	9.18E-02
light	GO:0010118~stomatal movement	5	3.52	6.27E-05	AT4G18290, AT4G08920, AT5G58140, AT1G04400, AT2G17260	140	29	18499	22.78202	2.55E-02	8.60E-04	8.77E-02
light	GO:0009903~chloroplast avoidance movement	5	3.52	2.89E-05	AT3G45780, AT1G42550, AT5G38150, AT1G75100, AT1G66840	140	24	18499	27.52827	1.18E-02	4.10E-04	4.04E-02
light	GO:0010119~regulation of stomatal movement	6	4.23	2.14E-05	AT2G46370, AT1G20090, AT2G26300, AT3G45780, AT5G27620, AT4G40100	140	45	18499	17.6181	8.77E-03	3.15E-04	2.99E-02
light	GO:0009911~positive regulation of flower development	6	4.23	1.04E-05	AT1G68050, AT1G26260, AT1G26830, AT1G25540, AT4G34530, AT1G04400	140	39	18499	20.32857	4.29E-03	1.59E-04	1.46E-02
light	GO:0071483~cellular response to blue light	4	2.82	8.17E-06	AT5G24120, AT2G21320, AT1G75100, AT2G36990	140	6	18499	88.09048	3.36E-03	1.29E-04	1.14E-02
light	GO:0009738~abscisic acid-activated signaling pathway	11	7.75	2.21E-06	AT5G63980, AT3G08550, AT1G21970, AT2G26300, AT1G70800, AT1G02340, AT1G42550, AT3G04110, AT3G59220, AT1G48270, AT1G54160	140	195	18499	7.45381	9.08E-04	3.64E-05	3.09E-03
light	GO:0009646~response to absence of light	6	4.23	1.29E-06	AT2G37678, AT1G31812, AT4G08920, AT3G47340, AT5G43470, AT1G04400	140	26	18499	30.49286	5.32E-04	2.31E-05	1.81E-03
light	GO:0009414~response to water deprivation	13	9.15	1.33E-06	AT4G08920, AT1G11755, AT5G27620, AT2G42620, AT5G63980, AT5G58070, AT3G47860, AT2G05620, AT5G58140, AT5G07690, AT1G54160, AT1G04400, AT5G49230	140	279	18499	6.156861	5.48E-04	2.28E-05	1.86E-03
light	GO:0045893~positive regulation of transcription, DNA-templated	12	8.45	7.55E-07	AT5G24120, AT2G37678, AT1G21970, AT4G27430, AT5G20730, AT3G54610, AT4G40060, AT2G36990, AT1G25540, AT4G15090, AT5G02200, AT4G25560	140	217	18499	7.307044	3.11E-04	1.41E-05	1.06E-03
light	GO:0009630~gravitropism	7	4.93	3.10E-07	AT1G70800, AT5G20730, AT1G05630, AT1G70940, AT2G18790, AT1G09570, AT2G21650	140	37	18499	24.99865	1.28E-04	6.08E-06	4.34E-04
light	GO:0040008~regulation of growth	10	7.04	6.84E-08	AT1G26945, AT1G26260, AT3G08550, AT4G31500, AT5G20730, AT1G10120, AT1G17060, AT2G26710, AT1G78370, AT4G28556	140	101	18499	13.08274	2.82E-05	1.41E-06	9.58E-05
light	GO:0042752~regulation of circadian rhythm	7	4.93	1.94E-08	AT2G21150, AT1G22770, AT1G10470, AT5G57360, AT4G08920, AT1G59940, AT1G04400	140	24	18499	38.53958	8.01E-06	4.22E-07	2.72E-05
light	GO:0006351~transcription, DNA-templated	40	28.17	2.66E-09	AT1G26945, AT5G59820, AT1G26260, AT1G21970, AT2G20180, AT4G16250, AT3G21150, AT2G36890, AT5G27620, AT1G10120, AT2G43010, AT2G21320, AT4G01120, AT1G09570, AT2G21650, AT3G61850, AT4G36930, AT5G48560, AT1G59940, AT4G40060, AT3G54610, AT5G07690, AT2G18790, AT2G37678, AT2G46590, AT1G02340, AT1G09530, AT1G25540, AT5G02200, AT4G34530, AT2G36530, AT4G25560, AT1G10470, AT5G39760, AT5G61270, AT5G20730, AT3G48100, AT4G28610, AT4G17490, AT1G54160	140	1886	18499	2.802454	1.10E-06	6.09E-08	3.73E-06
light	GO:0006355~regulation of transcription, DNA-templated	44	30.99	5.34E-10	AT5G59820, AT1G26945, AT1G26260, AT2G20180, AT4G16250, AT3G21150, AT2G36890, AT5G43630, AT5G27620, AT1G10120, AT2G43010, AT2G21320, AT2G36990, AT4G01120, AT3G02790, AT1G09570, AT2G21650, AT1G22770,	140	2119	18499	2.743734	2.20E-07	1.29E-08	7.48E-07

					AT5G24120, AT1G68050, AT3G61850, AT4G36930, AT5G48560, AT1G59940, AT4G40060, AT1G10170, AT5G07690, AT2G18790, AT2G46590, AT1G02340, AT1G08540, AT1G09530, AT4G34530, AT2G36530, AT4G25560, AT1G10470, AT5G39760, AT5G28490, AT5G61270, AT5G20730, AT3G48100, AT4G28610, AT4G17490, AT1G54160							
light	GO:0010017~red or far-red light signaling pathway	8	5.63	4.99E-10	AT1G10470, AT3G21150, AT4G16250, AT2G43010, AT1G09530, AT4G15090, AT2G46340, AT2G40080	140	25	18499	42.28343	2.05E-07	1.28E-08	6.99E-07
light	GO:0009639~response to red or far red light	9	6.34	2.10E-10	AT2G36910, AT2G37678, AT3G61850, AT5G58960, AT1G09530, AT1G26830, AT3G28860, AT4G15090, AT4G25560	140	36	18499	33.03393	8.66E-08	5.77E-09	2.94E-07
light	GO:0009642~response to light intensity	7	4.93	7.23E-11	AT1G10522, AT5G16400, AT3G62030, AT4G37270, AT1G02340, AT1G10170, AT3G02730	140	11	18499	84.08636	2.98E-08	2.13E-09	1.01E-07
light	GO:0010244~response to low fluence blue light stimulus by blue low-fluence system	7	4.93	1.33E-11	AT4G08920, AT2G26300, AT3G59220, AT2G43010, AT1G48270, AT2G18790, AT1G04400	140	9	18499	102.7722	5.49E-09	4.22E-10	1.86E-08
light	GO:0007623~circadian rhythm	13	9.15	8.28E-12	AT4G18290, AT5G57360, AT4G08920, AT1G10470, AT1G22770, AT1G68050, AT3G45780, AT4G36930, AT3G48100, AT1G59940, AT4G28610, AT5G58140, AT1G04400	140	97	18499	17.70891	3.41E-09	2.84E-10	1.16E-08
light	GO:0009644~response to high light intensity	12	8.45	2.60E-13	AT4G18290, AT4G27670, AT3G47390, AT4G08920, AT4G22260, AT5G58070, AT3G47860, AT2G05620, AT1G07350, AT4G17490, AT5G58140, AT2G42080	140	55	18499	28.82961	1.07E-10	9.76E-12	3.65E-10
light	GO:0009638~phototropism	9	6.34	1.90E-13	AT5G64330, AT4G08920, AT3G45780, AT1G70800, AT5G20730, AT5G58140, AT2G18790, AT1G09570, AT1G04400	140	17	18499	69.9542	7.84E-11	8.71E-12	2.67E-10
light	GO:0009640~photomorphogenesis	12	8.45	2.10E-13	AT2G46370, AT4G28880, AT2G36910, AT2G37678, AT4G08920, AT3G21150, AT4G28860, AT4G14110, AT3G28860, AT2G46340, AT2G18790, AT1G09570	140	54	18499	29.36349	8.64E-11	8.64E-12	2.94E-10
light	GO:0010161~red light signaling pathway	8	5.63	4.13E-14	AT2G39840, AT2G20180, AT2G24790, AT2G43010, AT1G59940, AT2G18790, AT1G09570, AT5G49230	140	9	18499	117.454	1.70E-11	2.13E-12	5.79E-11
light	GO:0018298~protein-chromophore linkage	12	8.45	2.35E-14	AT1G68050, AT2G05100, AT5G57360, AT4G08920, AT3G45780, AT2G05070, AT4G16250, AT3G27690, AT5G58140, AT2G18790, AT1G09570, AT1G04400	140	45	18499	35.23619	9.70E-12	1.39E-12	3.30E-11
light	GO:0010218~response to far red light	15	10.56	4.97E-19	AT2G37678, AT4G08920, AT1G02340, AT3G27690, AT1G25540, AT5G02200, AT1G09570, AT5G24120, AT1G22770, AT2G05100, AT5G28490, AT3G28860, AT2G46340, AT4G15090, AT2G18790	140	49	18499	40.44971	2.05E-16	3.41E-17	6.96E-16
light	GO:0009585~red, far-red light phototransduction	17	11.97	8.51E-21	AT1G53090, AT1G02340, AT4G16250, AT2G43010, AT1G09530, AT4G14110, AT1G25540, AT1G09570, AT4G25560, AT2G46370, AT1G22770, AT4G38740, AT5G61270, AT2G46340, AT4G15090, AT2G18790, AT2G40080	140	62	18499	36.23076	3.51E-18	7.02E-19	1.19E-17
light	GO:0010114~response to red light	21	14.79	1.14E-28	AT2G21150, AT2G37678, AT3G61600, AT5G57360, AT4G08920, AT1G08540, AT3G27690, AT1G25540, AT2G21650, AT1G10470, AT5G24120, AT5G17880, AT2G05100, AT4G31500, AT5G28490, AT4G36930, AT3G48100, AT1G59940, AT2G46260, AT2G46340, AT2G40080	140	58	18499	47.84224	4.71E-26	1.18E-26	1.60E-25
light	GO:0009785~blue light signaling pathway	18	12.68	5.04E-36	AT4G28880, AT1G21970, AT4G08920, AT3G21150, AT1G02340, AT2G36890, AT4G28860, AT1G48270, AT5G63870, AT5G64330, AT3G45780, AT2G26300, AT4G38740, AT5G20730, AT3G59220, AT1G54160, AT1G04400, AT5G49230	140	18	18499	132.1357	2.08E-33	6.92E-34	7.06E-33
light	GO:0009416~response to light stimulus	38	26.76	1.33E-42	AT1G26945, AT5G59820, AT4G08920, AT1G55480, AT1G17060, AT4G14110, AT2G42620, AT5G63980, AT1G20090, AT3G05420, AT4G27430, AT4G38740, AT2G05070, AT1G42550, AT3G04110, AT2G26710, AT1G77760, AT3G54610, AT1G37130, AT4G28556, AT2G38050, AT1G04400, AT2G17260, AT2G37678, AT3G08550, AT2G46590, AT5G27630, AT1G70940, AT2G36530, AT5G64330, AT3G19820, AT5G17880, AT1G79440, AT5G28490, AT5G61270, AT5G58070, AT5G08560, AT5G43470	140	188	18499	26.70828	5.46E-40	2.73E-40	1.86E-39
light	GO:0009637~response to blue light	34	23.94	9.03E-59	AT2G21150, AT4G28880, AT1G26260, AT4G08920, AT4G28860, AT3G27690, AT1G10120, AT5G27620, AT1G05630, AT5G55280, AT4G01120, AT5G24120, AT1G22770, AT1G68050, AT2G36910, AT3G45780, AT1G42550, AT5G48560, AT4G40060, AT5G58140, AT2G46340, AT1G66840, AT1G04400, AT1G53090, AT1G70800, AT1G08540, AT4G40100, AT5G02200, AT4G34530, AT2G05100, AT5G28490, AT5G38150, AT3G28860, AT5G43470	140	52	18499	86.39643	3.72E-56	3.72E-56	1.26E-55
osmotic	GO:0009688~abscisic acid biosynthetic process	3	2.63	3.62E-03	AT1G16540, AT5G67030, AT3G14440	113	15	18499	32.74159	7.58E-01	4.78E-02	4.92E+00
osmotic	GO:0010119~regulation of stomatal movement	4	3.51	2.55E-03	AT2G40220, AT2G21660, AT4G33950, AT5G03280	113	45	18499	14.55182	6.31E-01	3.50E-02	3.48E+00

osmotic	GO:0048481~plant ovule development	4	3.51	2.55E-03	AT3G45640, AT5G62090, AT2G43790, AT4G25520	113	45	18499	14.55182	6.31E-01	3.50E-02	3.48E+00
osmotic	GO:0009793~embryo development ending in seed dormancy	10	8.77	1.90E-03	AT1G32560, AT2G32700, AT2G35300, AT5G13170, AT5G45800, AT5G62090, AT4G25520, AT2G35510, AT5G06760, AT1G32230	113	459	18499	3.566622	5.24E-01	2.71E-02	2.61E+00
osmotic	GO:0010286~heat acclimation	4	3.51	1.56E-03	AT1G50500, AT1G20440, AT5G53060, AT2G42540	113	38	18499	17.23242	4.57E-01	2.42E-02	2.15E+00
osmotic	GO:0009787~regulation of abscisic acid-activated signaling pathway	3	2.63	1.58E-03	AT4G16830, AT2G22660, AT1G42550	113	10	18499	49.11239	4.62E-01	2.36E-02	2.18E+00
osmotic	GO:1902074~response to salt	3	2.63	1.27E-03	AT2G32700, AT5G52310, AT1G65690	113	9	18499	54.56932	3.92E-01	2.05E-02	1.75E+00
osmotic	GO:1901001~negative regulation of response to salt stress	3	2.63	9.93E-04	AT2G32700, AT5G62090, AT4G25520	113	8	18499	61.39049	3.22E-01	1.68E-02	1.37E+00
osmotic	GO:0048316~seed development	5	4.39	7.70E-04	AT1G32560, AT2G35300, AT2G40220, AT4G34710, AT5G06760	113	68	18499	12.03735	2.60E-01	1.36E-02	1.06E+00
osmotic	GO:0042542~response to hydrogen peroxide	5	4.39	3.19E-04	AT5G12030, AT1G77120, AT2G47900, AT2G43790, AT1G32230	113	54	18499	15.15814	1.17E-01	5.92E-03	4.42E-01
osmotic	GO:0009790~embryo development	3	2.63	1.09E-04	AT1G32560, AT2G35300, AT5G06760	113	3	18499	163.708	4.16E-02	2.12E-03	1.51E-01
osmotic	GO:0009789~positive regulation of abscisic acid-activated signaling pathway	5	4.39	5.13E-05	AT1G53300, AT5G03280, AT3G50500, AT5G66880, AT1G15100	113	34	18499	24.0747	1.99E-02	1.06E-03	7.13E-02
osmotic	GO:0010182~sugar mediated signaling pathway	5	4.39	3.09E-05	AT1G16540, AT2G40220, AT5G67030, AT5G03280, AT5G49450	113	30	18499	27.28466	1.20E-02	6.71E-04	4.29E-02
osmotic	GO:0010150~leaf senescence	7	6.14	2.01E-05	AT5G52310, AT5G13170, AT1G27320, AT2G43790, AT5G03280, AT5G35750, AT2G42540	113	92	18499	12.45604	7.83E-03	4.62E-04	2.79E-02
osmotic	GO:0006468~protein phosphorylation	18	15.79	1.85E-05	AT3G08730, AT5G08590, AT4G33950, AT1G60940, AT1G10940, AT5G66880, AT4G01370, AT3G46930, AT1G72180, AT2G43850, AT4G40010, AT5G57630, AT5G45800, AT2G23030, AT5G63650, AT3G50500, AT3G17510, AT1G78290	113	870	18499	3.387061	7.20E-03	4.51E-04	2.57E-02
osmotic	GO:0009408~response to heat	9	7.89	5.83E-06	AT5G57050, AT1G16540, AT1G50500, AT5G12030, AT2G38750, AT5G67030, AT5G03280, AT3G25230, AT1G35720	113	160	18499	9.208573	2.28E-03	1.52E-04	8.11E-03
osmotic	GO:0010029~regulation of seed germination	6	5.26	4.13E-06	AT1G42550, AT3G63060, AT1G27320, AT3G50500, AT5G35750, AT5G66880	113	40	18499	24.55619	1.61E-03	1.15E-04	5.74E-03
osmotic	GO:0042742~defense response to bacterium	12	10.53	9.25E-07	AT4G39090, AT3G54560, AT1G16540, AT3G46930, AT3G56400, AT5G24660, AT1G27320, AT4G33950, AT2G43790, AT5G03280, AT4G18780, AT2G41560	113	276	18499	7.117738	3.62E-04	2.78E-05	1.29E-03
osmotic	GO:0009631~cold acclimation	7	6.14	6.21E-07	AT5G67590, AT5G15960, AT1G20440, AT4G13850, AT4G04920, AT4G08500, AT2G42540	113	51	18499	22.46972	2.43E-04	2.02E-05	8.63E-04
osmotic	GO:0006972~hyperosmotic response	6	5.26	3.34E-09	AT4G38240, AT4G01370, AT5G12030, AT3G12490, AT5G19660, AT2G40880	113	11	18499	89.29525	1.30E-06	1.19E-07	4.64E-06
osmotic	GO:0035556~intracellular signal transduction	13	11.40	1.65E-09	AT3G08730, AT5G08590, AT4G33950, AT1G60940, AT1G10940, AT5G66880, AT5G57630, AT4G40010, AT2G23030, AT5G63650, AT3G50500, AT3G17510, AT1G78290	113	189	18499	11.26034	6.46E-07	6.46E-08	2.30E-06
osmotic	GO:0006979~response to oxidative stress	16	14.04	2.49E-10	AT2G22660, AT4G37900, AT5G03280, AT4G34710, AT2G40880, AT1G32230, AT2G32700, AT3G46930, AT3G45640, AT5G01410, AT3G12490, AT2G43790, AT1G35910, AT2G35510, AT2G31870, AT1G35720	113	291	18499	9.001125	9.74E-08	1.08E-08	3.46E-07
osmotic	GO:0009738~abscisic acid-activated signaling pathway	14	12.28	1.81E-10	AT2G17820, AT2G22660, AT2G01150, AT1G53300, AT3G63060, AT4G33950, AT4G17615, AT5G66880, AT5G57050, AT2G40220, AT3G45640, AT1G42550, AT3G50500, AT1G15100	113	195	18499	11.75339	7.08E-08	8.85E-09	2.52E-07
osmotic	GO:0047484~regulation of response to osmotic stress	7	6.14	3.00E-13	AT2G32700, AT5G19690, AT5G24660, AT5G62090, AT1G29060, AT4G25520, AT1G15100	113	7	18499	163.708	1.17E-10	1.67E-11	4.17E-10
osmotic	GO:0071470~cellular response to osmotic stress	9	7.89	6.23E-17	AT5G58580, AT4G16830, AT2G22660, AT5G57630, AT5G62460, AT5G13170, AT4G37900, AT2G47900, AT5G57345	113	10	18499	147.3372	4.34E-14	7.22E-15	1.55E-13
osmotic	GO:0009409~response to cold	23	20.18	5.37E-18	AT3G08730, AT1G16540, AT5G15960, AT1G20440, AT4G13850, AT1G27320, AT4G17615, AT4G34710, AT2G42540, AT2G40880, AT3G59770, AT5G06760, AT4G01370, AT5G52310, AT2G38750, AT2G21660, AT3G45640, AT3G23830, AT1G77120, AT3G12490, AT2G43790, AT4G08500, AT1G35720	113	299	18499	12.59292	2.10E-15	4.20E-16	7.47E-15
osmotic	GO:0009737~response to abscisic acid	27	23.68	5.38E-20	AT5G15960, AT5G66880, AT5G52310, AT3G46930, AT1G65690, AT2G38750, AT1G64670, AT1G77120, AT3G50500, AT3G17510, AT1G55870, AT4G16830, AT1G20440, AT1G53300, AT3G63060, AT4G33950, AT1G10940, AT4G34710, AT2G42540, AT5G57050, AT4G01370, AT2G40190, AT2G43790, AT5G08560, AT4G14605, AT5G35750, AT1G35720	113	394	18499	11.21857	2.10E-17	5.26E-18	7.48E-17
osmotic	GO:0009414~response to water deprivation	39	34.21	1.93E-41	AT5G15960, AT4G13850, AT1G27320, AT4G18780, AT5G66880, AT2G40880, AT1G32230, AT1G32560, AT5G52310, AT1G02730, AT1G69310, AT3G46930, AT2G38750, AT2G21660, AT1G77120, AT3G50500, AT2G31870, AT2G17820, AT2G37180, AT1G20440, AT3G63060, AT3G12630, AT4G33950, AT4G17615, AT4G01420, AT2G42540, AT5G06760, AT4G39090, AT5G57050, AT2G32700,	113	279	18499	22.88391	7.54E-39	2.51E-39	2.68E-38

					AT2G35300, AT3G23830, AT2G40220, AT5G67030, AT3G14440, AT3G12490, AT3G56580, AT5G35750, AT1G35720							
osmotic	GO:0009651~response to salt stress	49	42.98	2.89E-46	AT4G13850, AT1G60940, AT5G52310, AT2G38750, AT1G77120, AT3G50500, AT1G16540, AT4G16830, AT5G08590, AT2G47900, AT2G18250, AT1G10940, AT4G17615, AT4G39090, AT3G23830, AT2G43790, AT4G08500, AT1G35720, AT1G27320, AT5G03280, AT5G49450, AT5G66880, AT1G32230, AT1G02730, AT1G69310, AT3G46930, AT2G21660, AT5G01410, AT5G63650, AT3G17510, AT1G55870, AT3G08730, AT1G53300, AT5G24660, AT3G63060, AT4G33950, AT4G34710, AT4G01420, AT2G42540, AT2G41560, AT4G01370, AT3G20250, AT4G40010, AT5G19690, AT4G14605, AT5G35750, AT1G35910, AT2G35510, AT1G15100	113	484	18499	16.57374	1.13E-43	5.65E-44	4.02E-43
osmotic	GO:0006970~response to osmotic stress	81	71.05	3.45E-162	AT1G55180, AT5G15960, AT4G13850, AT4G18780, AT1G60940, AT5G52310, AT2G38750, AT1G64670, AT1G77120, AT1G42550, AT3G58620, AT5G01270, AT3G50500, AT1G78290, AT4G16830, AT1G16540, AT2G37180, AT5G08590, AT4G04340, AT2G18250, AT4G17615, AT1G10940, AT4G39090, AT5G57050, AT1G50500, AT2G43850, AT5G67590, AT2G35300, AT2G40220, AT2G40190, AT3G23830, AT1G58200, AT2G43790, AT4G04920, AT4G08500, AT1G35720, AT1G43700, AT1G27320, AT5G49450, AT3G25230, AT5G03280, AT5G66880, AT3G59770, AT1G32230, AT1G32560, AT3G46930, AT1G69310, AT1G02730, AT1G65690, AT3G45640, AT2G21660, AT2G23030, AT5G63650, AT3G17510, AT1G55870, AT2G31870, AT5G35550, AT3G08730, AT2G17820, AT2G22660, AT1G20440, AT1G53300, AT3G63060, AT3G12630, AT4G33950, AT2G42580, AT4G34710, AT4G01420, AT2G42540, AT2G41560, AT5G06760, AT4G40010, AT3G20250, AT5G67030, AT3G14440, AT4G14605, AT5G53060, AT5G08560, AT5G35750, AT1G35910, AT2G35510	113	122	18499	108.6914	1.35E-159	1.35E-159	4.80E-159
oxidative	GO:0000302~response to reactive oxygen species	4	2.65	2.34E-03	AT4G35000, AT2G01980, AT3G09640, AT4G02380	150	33	18499	14.94869	5.74E-01	4.18E-02	3.17E+00
oxidative	GO:0045454~cell redox homeostasis	7	4.64	1.61E-03	AT1G76080, AT4G35090, AT5G60640, AT3G06050, AT4G03520, AT1G03680, AT3G15360	150	155	18499	5.569591	4.45E-01	3.05E-02	2.20E+00
oxidative	GO:0071329~cellular response to sucrose stimulus	3	1.99	1.32E-03	AT3G11220, AT2G28190, AT1G08830	150	7	18499	52.85429	3.81E-01	2.63E-02	1.80E+00
oxidative	GO:0071493~cellular response to UV-B	3	1.99	1.32E-03	AT5G18100, AT2G28190, AT1G08830	150	7	18499	52.85429	3.81E-01	2.63E-02	1.80E+00
oxidative	GO:0071484~cellular response to light intensity	3	1.99	6.34E-04	AT5G18100, AT2G28190, AT1G08830	150	5	18499	73.996	2.06E-01	1.35E-02	8.69E-01
oxidative	GO:0031538~negative regulation of anthocyanin metabolic process	3	1.99	3.83E-04	AT3G11220, AT5G13680, AT4G10090	150	4	18499	92.495	1.30E-01	8.67E-03	5.25E-01
oxidative	GO:1902884~positive regulation of response to oxidative stress	3	1.99	3.83E-04	AT5G58070, AT3G06930, AT4G17410	150	4	18499	92.495	1.30E-01	8.67E-03	5.25E-01
oxidative	GO:0009408~response to heat	8	5.30	3.18E-04	AT5G59820, AT3G53990, AT5G58070, AT3G47860, AT3G04120, AT5G03280, AT2G19310, AT1G35720	150	160	18499	6.166333	1.09E-01	7.68E-03	4.36E-01
oxidative	GO:0090156~cellular sphingolipid homeostasis	3	1.99	1.92E-04	AT5G42000, AT1G01230, AT1G07380	150	3	18499	123.3267	6.76E-02	4.99E-03	2.64E-01
oxidative	GO:0071457~cellular response to ozone	3	1.99	1.92E-04	AT5G18100, AT2G28190, AT1G08830	150	3	18499	123.3267	6.76E-02	4.99E-03	2.64E-01
oxidative	GO:0010193~response to ozone	5	3.31	1.38E-04	AT4G25100, AT3G47450, AT1G08830, AT3G06110, AT1G32230	150	33	18499	18.68586	4.90E-02	3.85E-03	1.90E-01
oxidative	GO:0006457~protein folding	11	7.28	1.17E-04	AT1G76080, AT3G62030, AT5G60640, AT1G09210, AT5G42000, AT1G56340, AT4G17070, AT1G01230, AT4G03520, AT1G03680, AT3G15360	150	288	18499	4.710394	4.15E-02	3.53E-03	1.60E-01
oxidative	GO:0019430~removal of superoxide radicals	4	2.65	1.07E-04	AT4G25100, AT5G18100, AT2G28190, AT1G08830	150	12	18499	41.10889	3.81E-02	3.53E-03	1.47E-01
oxidative	GO:0006970~response to osmotic stress	8	5.30	5.90E-05	AT3G46930, AT3G45640, AT5G03280, AT1G35910, AT2G35510, AT1G35720, AT2G31870, AT1G32230	150	122	18499	8.086995	2.13E-02	2.15E-03	8.12E-02
oxidative	GO:0009409~response to cold	12	7.95	3.13E-05	AT5G63980, AT4G35090, AT5G59820, AT3G53990, AT3G45640, AT5G58070, AT1G27730, AT3G12490, AT4G02380, AT1G03680, AT1G35720, AT2G40880	150	299	18499	4.949565	1.13E-02	1.27E-03	4.31E-02
oxidative	GO:0055114~oxidation-reduction process	28	18.54	7.17E-06	AT4G25100, AT1G75280, AT1G08830, AT1G03680, AT1G76080, AT4G35090, AT3G06050, AT1G31170, AT4G35000, AT5G18100, AT3G09640, AT3G47450, AT3G11050, AT2G28190, AT5G25620, AT5G07460, AT1G63460, AT5G20230, AT2G39800, AT5G16990, AT4G25130, AT4G03520, AT5G16970, AT3G15360, AT2G14170, AT1G13440, AT4G04800, AT3G04120	150	1330	18499	2.596351	2.61E-03	3.26E-04	9.87E-03
oxidative	GO:0009737~response to abscisic acid	16	10.60	7.05E-07	AT3G62030, AT3G01420, AT2G39800, AT5G39610, AT3G22380, AT2G05710, AT5G13680, AT3G16890, AT3G46930,	150	394	18499	5.00819	2.57E-04	3.67E-05	9.70E-04

					AT3G11220, AT3G47860, AT1G27730, AT4G02380, AT3G11050, AT1G32640, AT1G35720							
oxidative	GO:0009414~response to water deprivation	15	9.93	6.00E-08	AT1G76080, AT5G63980, AT3G46930, AT2G39800, AT5G58070, AT1G27730, AT3G47860, AT1G02930, AT3G12490, AT3G22380, AT4G02380, AT2G40880, AT1G35720, AT2G31870, AT1G32230	150	279	18499	6.630466	2.18E-05	3.64E-06	8.25E-05
oxidative	GO:0046686~response to cadmium ion	17	11.26	1.74E-08	AT2G01140, AT4G25100, AT1G56340, AT1G52760, AT1G02930, AT2G05710, AT5G56550, AT3G22200, AT1G75280, AT5G16970, AT4G26970, AT1G13440, AT3G06050, AT3G04120, AT5G59880, AT3G14990, AT1G35720	150	342	18499	6.130273	6.32E-06	1.26E-06	2.39E-05
oxidative	GO:0042542~response to hydrogen peroxide	9	5.96	1.12E-08	AT2G01980, AT1G52760, AT5G39610, AT3G04120, AT4G02380, AT4G03240, AT2G19310, AT1G16420, AT1G32230	150	54	18499	20.55444	4.06E-06	1.01E-06	1.53E-05
oxidative	GO:0009651~response to salt stress	25	16.56	9.52E-13	AT3G62030, AT1G09210, AT2G39800, AT1G56340, AT5G39210, AT4G25130, AT1G02930, AT2G05710, AT5G03280, AT3G22200, AT1G08830, AT1G32230, AT3G16890, AT5G63980, AT3G46930, AT1G49300, AT2G01980, AT5G01410, AT1G27730, AT3G47450, AT2G47510, AT3G04120, AT1G35910, AT2G35510, AT1G35720	150	484	18499	6.370179	3.47E-10	1.16E-10	1.31E-09
oxidative	GO:0034599~cellular response to oxidative stress	20	13.25	3.17E-25	AT3G07700, AT2G41090, AT5G07460, AT4G25130, AT1G07380, AT4G03520, AT5G57345, AT4G00290, AT1G03680, AT1G08830, AT3G15360, AT1G76080, AT1G66330, AT1G31170, AT5G64940, AT4G35000, AT4G04800, AT3G09640, AT2G28190, AT3G06110	150	64	18499	38.53958	1.15E-22	5.76E-23	4.36E-22
oxidative	GO:0006979~response to oxidative stress	130	86.09	2.03E-223	AT4G12000, AT3G20340, AT1G72060, AT2G21195, AT5G50350, AT2G19310, AT1G30460, AT1G03680, AT1G69190, AT3G46090, AT4G35090, AT3G11220, AT4G35000, AT3G53990, AT4G17070, AT1G27730, AT4G11850, AT2G28190, AT4G03240, AT5G59080, AT2G01140, AT1G63460, AT1G80130, AT4G39640, AT5G41150, AT5G16990, AT3G55460, AT4G03520, AT5G13680, AT2G21640, AT1G21520, AT2G14170, AT2G01980, AT1G52200, AT3G01420, AT3G10020, AT3G16670, AT5G03280, AT5G56550, AT3G22200, AT1G09970, AT1G08830, AT1G75280, AT4G26970, AT1G76080, AT3G46930, AT1G31170, AT1G49300, AT5G01410, AT1G14870, AT3G47860, AT4G11830, AT2G31870, AT5G07460, AT2G40000, AT2G39800, AT2G05710, AT1G64360, AT5G16970, AT3G15360, AT1G09000, AT3G60980, AT1G73120, AT1G13340, AT5G37510, AT3G23910, AT1G11210, AT1G35910, AT3G06110, AT1G50170, AT4G25100, AT5G60640, AT3G53030, AT3G22380, AT1G78410, AT2G24150, AT1G49670, AT2G40880, AT2G22080, AT3G16890, AT5G08670, AT5G27830, AT3G06050, AT2G04795, AT4G08940, AT5G18100, AT3G09640, AT2G47510, AT4G02380, AT5G59880, AT1G52500, AT5G47650, AT4G02580, AT1G32220, AT3G25530, AT1G52760, AT5G39610, AT3G12490, AT3G51610, AT1G19020, AT1G35720, AT5G59820, AT3G62030, AT2G44240, AT1G56340, AT4G11010, AT2G15560, AT1G50290, AT2G19810, AT5G20140, AT1G32230, AT5G19875, AT3G45640, AT5G09830, AT3G47450, AT3G14430, AT3G11050, AT5G43750, AT5G18040, AT5G20230, AT1G09210, AT1G02930, AT1G27330, AT1G16420, AT1G13440, AT3G08670, AT3G04120, AT5G55070, AT4G10090, AT2G35510	150	291	18499	55.09439	7.39E-221	7.39E-221	2.79E-220
salt	GO:0042542~response to hydrogen peroxide	5	2.16	4.06E-03	AT2G01980, AT2G47180, AT2G47900, AT2G43790, AT1G32230	224	54	18499	7.646743	8.90E-01	5.00E-02	5.75E+00
salt	GO:0009658~chloroplast organization	7	3.03	4.10E-03	AT5G24120, AT5G55580, AT1G43160, AT3G18870, AT3G57180, AT4G14605, AT2G34620	224	125	18499	4.62475	8.92E-01	4.93E-02	5.80E+00
salt	GO:0098719~sodium ion import across plasma membrane	3	1.30	3.86E-03	AT5G27150, AT2G01980, AT3G06370	224	8	18499	30.96931	8.77E-01	4.87E-02	5.48E+00
salt	GO:0010337~regulation of salicylic acid metabolic process	3	1.30	3.86E-03	AT1G28380, AT1G05850, AT2G41010	224	8	18499	30.96931	8.77E-01	4.87E-02	5.48E+00
salt	GO:0009863~salicylic acid mediated signaling pathway	4	1.73	3.66E-03	AT1G66350, AT2G01570, AT3G03450, AT1G14920	224	26	18499	12.70536	8.63E-01	4.73E-02	5.20E+00
salt	GO:0009867~jasmonic acid mediated signaling pathway	5	2.16	3.54E-03	AT1G66350, AT2G01570, AT3G03450, AT1G14920, AT1G32230	224	52	18499	7.940848	8.54E-01	4.69E-02	5.03E+00
salt	GO:0030154~cell differentiation	11	4.76	3.26E-03	AT2G32700, AT4G37260, AT1G66350, AT5G67300, AT3G23250, AT5G62090, AT3G03450, AT2G47460, AT4G25520, AT1G17950, AT1G66230	224	296	18499	3.069031	8.30E-01	4.44E-02	4.65E+00
salt	GO:0009753~response to jasmonic acid	8	3.46	2.87E-03	AT4G37260, AT5G67300, AT3G23250, AT1G43160, AT2G39770, AT5G13330, AT5G03280, AT4G34710	224	156	18499	4.235119	7.89E-01	4.02E-02	4.10E+00

salt	GO:0009740~gibberellic acid mediated signaling pathway	6	2.60	1.88E-03	AT2G27300, AT5G14920, AT1G66350, AT2G01570, AT3G03450, AT1G14920	224	73	18499	6.787794	6.39E-01	2.72E-02	2.70E+00
salt	GO:0009620~response to fungus	6	2.60	1.77E-03	AT4G01370, AT2G32700, AT3G05360, AT2G47900, AT1G73500, AT2G43790	224	72	18499	6.882068	6.17E-01	2.63E-02	2.54E+00
salt	GO:0009825~multidimensional cell growth	4	1.73	1.69E-03	AT1G03060, AT5G60920, AT3G46550, AT1G05850	224	20	18499	16.51696	6.01E-01	2.59E-02	2.44E+00
salt	GO:0048573~photoperiodism, flowering	5	2.16	1.47E-03	AT5G63980, AT2G27300, AT3G44110, AT1G06040, AT2G35510	224	41	18499	10.07132	5.49E-01	2.31E-02	2.12E+00
salt	GO:0048366~leaf development	8	3.46	8.06E-04	AT4G24560, AT5G27150, AT1G15690, AT3G47450, AT4G33950, AT5G56030, AT4G38630, AT5G02410	224	125	18499	5.285429	3.54E-01	1.32E-02	1.17E+00
salt	GO:0042742~defense response to bacterium	12	5.19	5.46E-04	AT5G46350, AT1G16540, AT3G62030, AT3G46930, AT5G67300, AT2G39770, AT2G38470, AT4G33950, AT2G43790, AT5G03280, AT1G10170, AT2G41560	224	276	18499	3.590644	2.56E-01	9.21E-03	7.92E-01
salt	GO:0006814~sodium ion transport	5	2.16	5.01E-04	AT5G27150, AT2G01980, AT4G10310, AT3G06370, AT3G19490	224	31	18499	13.32013	2.38E-01	8.72E-03	7.27E-01
salt	GO:0045892~negative regulation of transcription, DNA-templated	9	3.90	2.23E-04	AT3G61050, AT5G63980, AT5G03740, AT2G32700, AT4G26630, AT5G05660, AT4G06634, AT1G27730, AT4G21670	224	134	18499	5.546742	1.14E-01	4.02E-03	3.24E-01
salt	GO:0006355~regulation of transcription, DNA-templated	45	19.48	2.12E-04	AT3G57180, AT3G06930, AT1G16060, AT5G49450, AT1G14920, AT1G17950, AT5G24120, AT3G61890, AT1G69310, AT1G43160, AT3G18870, AT5G67300, AT5G04760, AT2G45640, AT5G62090, AT2G38470, AT2G47460, AT4G25520, AT1G10170, AT1G14350, AT2G26430, AT5G46350, AT2G01570, AT5G05660, AT5G63110, AT1G06040, AT1G12610, AT2G30250, AT5G13330, AT2G47900, AT3G06590, AT2G34620, AT1G66230, AT2G27300, AT5G55580, AT2G32700, AT4G37260, AT1G66350, AT2G01430, AT3G23250, AT3G51960, AT2G38340, AT5G08520, AT4G14605, AT3G03450	224	2119	18499	1.753807	1.08E-01	3.95E-03	3.08E-01
salt	GO:0009739~response to gibberellin	8	3.46	1.96E-04	AT2G27300, AT5G14920, AT1G66350, AT5G67300, AT3G50500, AT3G03450, AT1G14920, AT5G66880	224	99	18499	6.673521	1.01E-01	3.78E-03	2.85E-01
salt	GO:2000033~regulation of seed dormancy process	4	1.73	1.38E-04	AT1G66350, AT2G01570, AT3G03450, AT1G14920	224	9	18499	36.70437	7.19E-02	2.76E-03	2.00E-01
salt	GO:0009789~positive regulation of abscisic acid-activated signaling pathway	6	2.60	5.09E-05	AT1G53300, AT5G03280, AT3G50500, AT5G66880, AT3G17980, AT1G15100	224	34	18499	14.57379	2.72E-02	1.06E-03	7.41E-02
salt	GO:0035556~intracellular signal transduction	12	5.19	1.91E-05	AT5G08590, AT5G57630, AT4G40010, AT4G23650, AT5G63650, AT5G35410, AT4G33950, AT1G60940, AT3G50500, AT1G10940, AT3G17510, AT5G66880	224	189	18499	5.243481	1.03E-02	4.14E-04	2.78E-02
salt	GO:0034605~cellular response to heat	6	2.60	1.30E-05	AT1G43160, AT2G38340, AT2G30250, AT5G61780, AT2G38470, AT5G07350	224	26	18499	19.05804	7.03E-03	3.07E-04	1.90E-02
salt	GO:0045893~positive regulation of transcription, DNA-templated	13	5.63	1.33E-05	AT2G37678, AT5G05660, AT4G06634, AT5G13330, AT5G49450, AT5G24120, AT2G32700, AT3G61890, AT3G05700, AT2G38340, AT1G43160, AT1G73500, AT2G47460	224	217	18499	4.947478	7.21E-03	3.01E-04	1.94E-02
salt	GO:1901000~regulation of response to salt stress	4	1.73	6.85E-06	AT5G58580, AT2G37678, AT4G24560, AT4G14300	224	4	18499	82.58482	3.71E-03	1.69E-04	9.98E-03
salt	GO:0009938~negative regulation of gibberellic acid mediated signaling pathway	5	2.16	6.35E-06	AT2G27300, AT1G66350, AT2G01570, AT3G03450, AT1G14920	224	11	18499	37.53856	3.43E-03	1.64E-04	9.24E-03
salt	GO:0010228~vegetative to reproductive phase transition of meristem	10	4.33	3.97E-06	AT3G48680, AT3G51780, AT5G63110, AT1G06040, AT1G10570, AT1G60220, AT4G14300, AT5G63510, AT3G06930, AT3G63060	224	102	18499	8.096551	2.15E-03	1.19E-04	5.78E-03
salt	GO:0071470~cellular response to osmotic stress	5	2.16	4.08E-06	AT5G58580, AT4G16830, AT5G57630, AT5G62460, AT2G47900	224	10	18499	41.29241	2.21E-03	1.16E-04	5.94E-03
salt	GO:0010187~negative regulation of seed germination	6	2.60	4.23E-06	AT1G66350, AT2G01570, AT1G16060, AT5G56030, AT3G03450, AT1G14920	224	21	18499	23.59566	2.29E-03	1.15E-04	6.16E-03
salt	GO:1902074~response to salt	5	2.16	2.47E-06	AT2G32700, AT1G65690, AT5G08450, AT5G24240, AT2G34208	224	9	18499	45.88046	1.34E-03	7.88E-05	3.60E-03
salt	GO:1901002~positive regulation of response to salt stress	5	2.16	6.99E-07	AT5G46350, AT5G58070, AT1G29060, AT5G26751, AT3G17980	224	7	18499	58.98916	3.79E-04	2.53E-05	1.02E-03
salt	GO:0009738~abscisic acid-activated signaling pathway	14	6.06	7.29E-07	AT5G63980, AT4G23650, AT5G67300, AT2G38340, AT1G53300, AT3G63060, AT4G33950, AT3G46550, AT3G50500, AT4G17615, AT4G21670, AT5G66880, AT3G17980, AT1G15100	224	195	18499	5.929167	3.95E-04	2.47E-05	1.06E-03
salt	GO:0042538~hyperosmotic salinity response	9	3.90	3.91E-07	AT1G66350, AT2G01570, AT5G46180, AT3G51960, AT5G01410, AT5G58070, AT3G03450, AT5G26751, AT1G14920	224	57	18499	13.03971	2.12E-04	1.51E-05	5.70E-04
salt	GO:0006979~response to oxidative stress	19	8.23	1.63E-08	AT3G62030, AT2G47180, AT2G16500, AT1G56600, AT5G03280, AT3G22200, AT4G34710, AT1G32230, AT3G16890, AT2G32700, AT3G46930, AT1G49300, AT2G01980, AT5G01410, AT1G27730, AT3G47450, AT2G43790, AT1G35910, AT2G35510	224	291	18499	5.392136	8.86E-06	6.81E-07	2.38E-05
salt	GO:0047484~regulation of response to osmotic stress	6	2.60	5.01E-09	AT2G32700, AT5G19690, AT5G62090, AT1G29060, AT4G25520, AT1G15100	224	7	18499	70.78699	2.72E-06	2.26E-07	7.30E-06
salt	GO:0009723~response to ethylene	14	6.06	3.38E-09	AT2G01570, AT4G24800, AT5G13330, AT5G40770, AT5G03280, AT1G14920, AT1G32230, AT4G37260, AT1G66350, AT3G23250, AT5G67300, AT2G43790, AT2G47460, AT3G03450	224	124	18499	9.324093	1.83E-06	1.66E-07	4.92E-06
salt	GO:0010029~regulation of seed germination	10	4.33	9.04E-10	AT2G37678, AT2G01570, AT2G19450, AT3G63060, AT3G50500, AT3G03450,	224	40	18499	20.64621	4.90E-07	5.45E-08	1.32E-06

					AT4G38630, AT1G14920, AT3G54770, AT5G66880							
salt	GO:0009408~response to heat	16	6.93	9.84E-10	AT1G16540, AT3G44110, AT1G12610, AT2G47180, AT3G06010, AT2G04030, AT5G56030, AT5G03280, AT3G09350, AT1G59860, AT3G51780, AT5G58070, AT2G39770, AT2G38470, AT4G38630, AT1G05850	224	160	18499	8.258482	5.34E-07	5.34E-08	1.43E-06
salt	GO:2000377~regulation of reactive oxygen species metabolic process	10	4.33	1.19E-12	AT2G32010, AT5G14920, AT1G66350, AT2G01570, AT2G01980, AT2G01900, AT4G33950, AT3G03450, AT1G14920, AT1G32230	224	21	18499	39.32611	6.43E-10	8.03E-11	1.73E-09
salt	GO:1901001~negative regulation of response to salt stress	8	3.46	2.67E-13	AT2G32700, AT3G51960, AT4G33730, AT5G62090, AT1G30580, AT4G25520, AT1G78310, AT3G49810	224	8	18499	82.58482	1.45E-10	2.06E-11	3.88E-10
salt	GO:0071472~cellular response to salt stress	12	5.19	9.36E-14	AT1G53210, AT1G03060, AT4G16830, AT2G27300, AT2G32010, AT5G57630, AT5G62460, AT3G45680, AT2G01900, AT3G49810, AT2G03680, AT1G67580	224	33	18499	30.03084	5.07E-11	8.45E-12	1.36E-10
salt	GO:0009409~response to cold	26	11.26	2.78E-14	AT2G47180, AT2G19450, AT4G13850, AT1G56600, AT2G16500, AT5G63980, AT1G43160, AT3G22310, AT4G29810, AT1G27730, AT2G38470, AT3G50310, AT1G16540, AT5G08620, AT4G24500, AT2G30250, AT4G17615, AT4G34710, AT4G12480, AT4G01370, AT3G51780, AT3G51960, AT3G23830, AT5G58070, AT2G43790, AT4G24190	224	299	18499	7.181289	1.50E-11	3.01E-12	4.04E-11
salt	GO:0009414~response to water deprivation	47	20.35	4.55E-39	AT1G74920, AT2G47180, AT1G16060, AT4G13850, AT2G04030, AT2G45960, AT1G56600, AT5G66880, AT1G32230, AT3G61050, AT5G63980, AT5G03740, AT3G61890, AT3G46930, AT2G26650, AT1G02730, AT1G69310, AT3G05700, AT2G33700, AT1G43160, AT5G67300, AT3G22310, AT2G34208, AT1G27730, AT2G38470, AT3G50500, AT3G50310, AT1G05850, AT5G08620, AT1G12610, AT5G13330, AT3G06010, AT3G63060, AT4G33950, AT5G56030, AT3G51920, AT4G17615, AT3G54770, AT4G01420, AT2G32700, AT5G25370, AT1G15690, AT3G23830, AT2G38340, AT5G58070, AT4G24190, AT2G41010	224	279	18499	13.91214	2.47E-36	6.16E-37	6.62E-36
salt	GO:0009737~response to abscisic acid	57	24.68	4.59E-44	AT1G61210, AT4G32150, AT1G16060, AT3G16890, AT1G03060, AT5G03740, AT3G05700, AT5G14920, AT1G43160, AT5G67300, AT2G34208, AT1G27730, AT3G50500, AT5G46350, AT4G16830, AT5G13330, AT3G51920, AT1G10940, AT2G43790, AT4G38630, AT3G03450, AT4G27710, AT3G62030, AT5G08450, AT2G47180, AT2G19450, AT5G63510, AT3G48330, AT1G51500, AT1G56600, AT1G14920, AT1G17950, AT5G66880, AT3G48680, AT1G17840, AT1G65690, AT3G46930, AT3G61890, AT2G33700, AT5G24240, AT2G45640, AT3G17510, AT1G55870, AT2G01570, AT5G63110, AT1G53300, AT3G63060, AT4G33950, AT4G34710, AT3G54770, AT4G12480, AT4G01370, AT4G37260, AT5G25370, AT1G66350, AT1G56570, AT4G14605	224	394	18499	11.94755	2.49E-41	8.28E-42	6.68E-41
salt	GO:0006970~response to osmotic stress	40	17.32	2.37E-45	AT4G13850, AT4G10310, AT5G03280, AT1G60940, AT5G49450, AT5G66880, AT1G32230, AT1G65690, AT3G61890, AT1G02730, AT1G69310, AT3G46930, AT1G59860, AT1G43160, AT5G63650, AT2G38470, AT3G50500, AT3G17510, AT1G55870, AT4G16830, AT1G16540, AT5G08590, AT2G30250, AT1G53300, AT3G63060, AT4G33950, AT2G18250, AT4G34710, AT1G10940, AT4G17615, AT4G01420, AT2G41560, AT4G40010, AT3G20250, AT3G23830, AT3G51960, AT4G14605, AT2G43790, AT1G35910, AT2G35510	224	122	18499	27.07699	1.29E-42	6.43E-43	3.46E-42
salt	GO:0009651~response to salt stress	173	74.89	3.80E-237	AT1G74920, AT1G61210, AT4G24800, AT4G32150, AT1G16060, AT1G51460, AT3G46550, AT3G12360, AT1G60940, AT5G63980, AT3G61050, AT1G43160, AT1G27730, AT1G73500, AT3G50500, AT2G47460, AT3G50310, AT1G10170, AT3G19490, AT3G17980, AT2G26430, AT4G16830, AT5G60920, AT1G04120, AT1G73660, AT4G24500, AT3G55270, AT2G18250, AT4G17615, AT1G10940, AT5G22360, AT2G01980, AT3G51960, AT1G60490, AT2G43790, AT3G03450, AT4G38630, AT2G15390, AT3G06370, AT3G48850, AT1G10570, AT3G57180, AT2G19450, AT3G48330, AT4G10310, AT5G03280, AT2G16500, AT1G51500, AT1G14920, AT3G22200, AT5G66880, AT5G14040, AT5G11150, AT1G17840, AT1G13930, AT3G46930, AT1G49300, AT3G61890, AT1G59860, AT5G01410, AT1G05850, AT3G17510, AT1G55870,	224	484	18499	29.51895	2.06E-234	2.06E-234	5.53E-234

					AT2G43430, AT3G27460, AT5G05660, AT2G30250, AT1G53300, AT3G63060, AT1G31470, AT4G33950, AT5G26751, AT1G10880, AT2G27300, AT5G55580, AT4G01370, AT4G40010, AT3G20250, AT1G66350, AT3G51780, AT1G15690, AT1G56570, AT1G16850, AT1G60220, AT2G39770, AT4G14605, AT4G24190, AT2G17270, AT1G35910, AT1G10370, AT1G15100, AT4G13850, AT2G04030, AT5G40770, AT1G50960, AT3G47950, AT5G37370, AT3G09350, AT3G16890, AT1G28380, AT5G03740, AT4G26630, AT3G05700, AT5G14920, AT5G67300, AT1G24460, AT4G22330, AT5G02410, AT1G16540, AT2G47580, AT5G08590, AT2G38170, AT1G06040, AT1G12610, AT5G13330, AT2G47900, AT3G51920, AT5G56030, AT4G21670, AT1G14080, AT5G27150, AT3G23250, AT3G23830, AT1G27760, AT1G30580, AT5G07350, AT2G16005, AT4G27710, AT3G44110, AT3G62030, AT5G35080, AT2G47180, AT5G63510, AT2G45960, AT5G49450, AT1G56600, AT1G32230, AT2G46500, AT3G48680, AT5G44440, AT2G26650, AT1G69310, AT1G02730, AT2G33700, AT3G22310, AT4G29810, AT5G40550, AT5G63650, AT3G47450, AT2G45640, AT2G38470, AT3G23940, AT5G02020, AT5G08620, AT4G24560, AT2G01570, AT5G46180, AT5G63110, AT3G06010, AT5G35410, AT4G34710, AT3G54770, AT4G01420, AT2G41560, AT1G64460, AT4G12480, AT5G25370, AT4G23650, AT5G19690, AT2G38340, AT5G61780, AT2G35510, AT2G41010							
water	GO:0080036~regulation of cytokinin-activated signaling pathway	3	1.42	4.26E-03	AT4G31920, AT3G16857, AT2G25180	208	9	18499	29.64583	8.82E-01	4.96E-02	5.96E+00
water	GO:0034389~lipid particle organization	3	1.42	4.26E-03	AT1G67360, AT3G05500, AT2G47780	208	9	18499	29.64583	8.82E-01	4.96E-02	5.96E+00
water	GO:0042538~hyperosmotic salinity response	5	2.37	3.79E-03	AT2G39800, AT3G14080, AT5G44650, AT3G14440, AT1G19120	208	57	18499	7.801535	8.50E-01	4.53E-02	5.32E+00
water	GO:1902456~regulation of stomatal opening	3	1.42	3.34E-03	AT5G57050, AT1G12480, AT4G33950	208	8	18499	33.35156	8.13E-01	4.10E-02	4.70E+00
water	GO:0080113~regulation of seed growth	3	1.42	3.34E-03	AT4G31920, AT3G16857, AT2G25180	208	8	18499	33.35156	8.13E-01	4.10E-02	4.70E+00
water	GO:0045927~positive regulation of growth	3	1.42	2.52E-03	AT1G67360, AT3G05500, AT2G47780	208	7	18499	38.11607	7.18E-01	3.19E-02	3.57E+00
water	GO:0010117~photoprotection	3	1.42	2.52E-03	AT4G08920, AT1G27730, AT2G05620	208	7	18499	38.11607	7.18E-01	3.19E-02	3.57E+00
water	GO:0071368~cellular response to cytokinin stimulus	3	1.42	2.52E-03	AT4G31920, AT3G16857, AT2G25180	208	7	18499	38.11607	7.18E-01	3.19E-02	3.57E+00
water	GO:0006351~transcription, DNA-templated	36	17.06	1.90E-03	AT4G31920, AT1G22810, AT1G15360, AT3G15500, AT5G27620, AT1G16060, AT4G24020, AT1G28520, AT1G52890, AT1G45249, AT1G18390, AT5G03740, AT1G69310, AT2G46400, AT1G33240, AT3G16857, AT4G25480, AT2G25180, AT3G10500, AT1G27730, AT3G23050, AT4G27410, AT5G07690, AT3G11020, AT3G20310, AT1G78080, AT3G56400, AT1G12610, AT2G38880, AT2G40750, AT1G36060, AT2G40220, AT1G69600, AT1G54160, AT5G05410, AT1G46768	208	1886	18499	1.697641	6.15E-01	2.48E-02	2.71E+00
water	GO:2000377~regulation of reactive oxygen species metabolic process	4	1.90	1.58E-03	AT4G08920, AT3G10500, AT4G33950, AT1G32230	208	21	18499	16.94048	5.48E-01	2.18E-02	2.26E+00
water	GO:0010119~regulation of stomatal movement	5	2.37	1.58E-03	AT2G18960, AT2G40220, AT2G31470, AT5G27620, AT4G33950	208	45	18499	9.881944	5.48E-01	2.12E-02	2.26E+00
water	GO:0042742~defense response to bacterium	11	5.21	1.13E-03	AT4G39090, AT3G46930, AT1G33560, AT3G30775, AT3G56400, AT4G26070, AT1G02205, AT5G11270, AT3G16640, AT4G33950, AT4G18780	208	276	18499	3.544611	4.34E-01	1.61E-02	1.62E+00
water	GO:0010029~regulation of seed germination	5	2.37	1.02E-03	AT1G20450, AT3G63060, AT3G50500, AT3G54770, AT5G66880	208	40	18499	11.11719	3.99E-01	1.49E-02	1.45E+00
water	GO:0048316~seed development	6	2.84	9.84E-04	AT1G32560, AT5G05860, AT2G35300, AT2G40220, AT1G54160, AT5G06760	208	68	18499	7.847426	3.89E-01	1.48E-02	1.41E+00
water	GO:0045892~negative regulation of transcription, DNA-templated	8	3.79	7.86E-04	AT3G61050, AT5G63980, AT5G03740, AT1G33240, AT3G20310, AT3G56400, AT1G27730, AT1G54160	208	134	18499	5.309701	3.25E-01	1.22E-02	1.13E+00
water	GO:0015840~urea transport	3	1.42	7.37E-04	AT3G16240, AT2G36830, AT4G01470	208	4	18499	66.70313	3.09E-01	1.18E-02	1.06E+00
water	GO:0009688~abscisic acid biosynthetic process	4	1.90	5.69E-04	AT1G30100, AT5G67030, AT3G14440, AT1G52340	208	15	18499	23.71667	2.48E-01	9.78E-03	8.16E-01
water	GO:0006810~transport	13	6.16	5.89E-04	AT2G37170, AT2G37180, AT3G54820, AT3G16240, AT4G35100, AT2G45960, AT4G00430, AT3G53420, AT3G61430, AT2G36830, AT3G04090, AT1G01620, AT4G01470	208	349	18499	3.312858	2.55E-01	9.78E-03	8.44E-01
water	GO:0006979~response to oxidative stress	12	5.69	4.55E-04	AT3G62770, AT1G76080, AT3G46930, AT2G39800, AT3G30775, AT2G37040, AT1G27730, AT3G12490, AT3G22380, AT2G31870, AT2G40880, AT1G32230	208	291	18499	3.667526	2.04E-01	8.41E-03	6.54E-01
water	GO:0090332~stomatal closure	4	1.90	4.59E-04	AT5G64010, AT1G12480, AT5G56030, AT2G31870	208	14	18499	25.41071	2.05E-01	8.18E-03	6.59E-01

water	GO:0031537~regulation of anthocyanin metabolic process	3	1.42	3.71E-04	AT4G31920, AT3G16857, AT2G25180	208	3	18499	88.9375	1.70E-01	7.13E-03	5.33E-01
water	GO:0009790~embryo development	3	1.42	3.71E-04	AT1G32560, AT2G35300, AT5G06760	208	3	18499	88.9375	1.70E-01	7.13E-03	5.33E-01
water	GO:0080148~negative regulation of response to water deprivation	3	1.42	3.71E-04	AT3G01650, AT5G14420, AT1G10370	208	3	18499	88.9375	1.70E-01	7.13E-03	5.33E-01
water	GO:0010182~sugar mediated signaling pathway	5	2.37	3.32E-04	AT2G40220, AT3G26090, AT5G67030, AT1G52340, AT2G20890	208	30	18499	14.82292	1.53E-01	6.64E-03	4.77E-01
water	GO:0045893~positive regulation of transcription, DNA-templated	11	5.21	1.72E-04	AT5G09410, AT3G11020, AT3G05700, AT2G40220, AT3G10500, AT4G27410, AT1G69600, AT1G28520, AT5G05410, AT1G46768, AT1G45249	208	217	18499	4.508353	8.26E-02	3.58E-03	2.47E-01
water	GO:0010200~response to chitin	9	4.27	1.27E-04	AT5G59550, AT2G46400, AT3G23250, AT3G53600, AT3G46620, AT3G56400, AT3G52450, AT2G35930, AT1G27730	208	133	18499	6.018327	6.18E-02	2.77E-03	1.83E-01
water	GO:0050826~response to freezing	5	2.37	7.85E-05	AT5G63980, AT5G09410, AT5G60410, AT1G12610, AT2G42540	208	21	18499	21.1756	3.86E-02	1.87E-03	1.13E-01
water	GO:0009408~response to heat	10	4.74	8.12E-05	AT5G57050, AT1G12610, AT5G67030, AT3G06010, AT5G56030, AT2G04030, AT1G28520, AT1G05850, AT5G05410, AT1G52340	208	160	18499	5.558594	3.99E-02	1.85E-03	1.17E-01
water	GO:0009788~negative regulation of abscisic acid-activated signaling pathway	6	2.84	7.06E-05	AT5G57050, AT2G39550, AT2G31470, AT1G08720, AT5G40280, AT3G59380	208	39	18499	13.68269	3.48E-02	1.77E-03	1.02E-01
water	GO:0009631~cold acclimation	7	3.32	2.16E-05	AT1G20450, AT4G25480, AT3G14080, AT1G20440, AT1G19120, AT1G28520, AT2G42540	208	51	18499	12.20711	1.08E-02	5.70E-04	3.11E-02
water	GO:0010107~potassium ion import	4	1.90	1.36E-05	AT5G47100, AT2G26650, AT1G30270, AT4G17615	208	5	18499	71.15	6.78E-03	3.78E-04	1.96E-02
water	GO:0042631~cellular response to water deprivation	6	2.84	1.10E-05	AT1G18390, AT1G33240, AT5G05860, AT2G43350, AT2G31470, AT3G28270	208	27	18499	19.76389	5.52E-03	3.25E-04	1.59E-02
water	GO:0009873~ethylene-activated signaling pathway	12	5.69	5.66E-06	AT1G36060, AT1G22810, AT3G20310, AT2G40220, AT1G78080, AT1G15360, AT1G08720, AT1G16060, AT4G18780, AT1G05850, AT1G46768, AT1G32230	208	179	18499	5.962291	2.83E-03	1.77E-04	8.15E-03
water	GO:0010286~heat acclimation	7	3.32	3.74E-06	AT3G11020, AT5G60410, AT5G44650, AT1G20440, AT5G56030, AT5G05410, AT2G42540	208	38	18499	16.38322	1.87E-03	1.25E-04	5.39E-03
water	GO:1902584~positive regulation of response to water deprivation	5	2.37	1.03E-06	AT5G58787, AT1G67360, AT2G42620, AT3G05500, AT2G47780	208	8	18499	55.58594	5.16E-04	3.68E-05	1.48E-03
water	GO:0009409~response to cold	18	8.53	4.73E-08	AT1G20450, AT5G08620, AT5G09410, AT1G78080, AT1G20440, AT4G17615, AT2G42540, AT2G40880, AT5G06760, AT5G63980, AT4G25480, AT3G22310, AT5G52440, AT1G27730, AT3G12490, AT4G24190, AT3G50310, AT1G46768	208	299	18499	5.354097	2.37E-05	1.82E-06	6.81E-05
water	GO:0051865~protein autoubiquitination	7	3.32	5.09E-09	AT1G06770, AT5G59550, AT2G30580, AT3G46620, AT3G52450, AT2G35930, AT3G12630	208	14	18499	44.46875	2.55E-06	2.12E-07	7.33E-06
water	GO:0009819~drought recovery	7	3.32	1.27E-11	AT3G20250, AT3G10500, AT2G37040, AT1G70670, AT3G16640, AT1G07240, AT2G42540	208	7	18499	88.9375	6.34E-09	5.77E-10	1.82E-08
water	GO:0009738~abscisic acid-activated signaling pathway	20	9.48	7.06E-13	AT3G29320, AT2G17820, AT2G43350, AT2G31470, AT5G11270, AT3G63060, AT4G33950, AT4G17615, AT5G66880, AT1G45249, AT5G59550, AT5G63980, AT5G57050, AT5G47100, AT2G40220, AT1G12480, AT3G46620, AT1G08720, AT3G50500, AT1G54160	208	195	18499	9.121795	3.54E-10	3.54E-11	1.02E-09
water	GO:0010118~stomatal movement	11	5.21	4.12E-13	AT5G47100, AT4G08920, AT1G30270, AT2G31470, AT1G12480, AT5G11270, AT4G24020, AT2G47800, AT4G33950, AT4G17615, AT1G04400	208	29	18499	33.73491	2.06E-10	2.29E-11	5.93E-10
water	GO:2000070~regulation of response to water deprivation	9	4.27	3.44E-14	AT5G52050, AT5G60410, AT2G41225, AT5G52440, AT4G14300, AT5G27620, AT2G41230, AT4G05590, AT2G20890	208	11	18499	72.76705	1.72E-11	2.16E-12	4.96E-11
water	GO:0034220~ion transmembrane transport	13	6.16	7.17E-17	AT2G37170, AT2G37180, AT3G54820, AT4G35100, AT2G45960, AT5G18290, AT4G00430, AT3G53420, AT3G61430, AT2G36830, AT3G04090, AT1G01620, AT4G01470	208	28	18499	41.29241	5.56E-14	7.99E-15	1.55E-13
water	GO:0009992~cellular water homeostasis	15	7.11	1.48E-18	AT2G37170, AT2G37180, AT3G54820, AT3G16240, AT4G35100, AT2G45960, AT5G18290, AT4G00430, AT3G53420, AT1G21270, AT3G61430, AT2G36830, AT3G04090, AT1G01620, AT4G01470	208	37	18499	36.05574	7.43E-16	1.24E-16	2.14E-15
water	GO:0006833~water transport	11	5.21	2.69E-19	AT2G37170, AT3G53420, AT3G54820, AT3G16240, AT3G61430, AT2G36830, AT1G01620, AT2G45960, AT3G24715, AT4G01470, AT4G00430	208	11	18499	88.9375	1.35E-16	2.70E-17	3.88E-16
water	GO:0009651~response to salt stress	43	20.38	1.60E-25	AT1G74920, AT3G62770, AT1G16060, AT2G04030, AT2G45960, AT5G66880, AT1G45249, AT1G32230, AT3G61050, AT5G63980, AT5G03740, AT5G62470, AT3G46930, AT2G26650, AT1G02730, AT1G69310, AT3G05700, AT3G22310, AT1G27730, AT3G50500, AT3G50310, AT1G05850, AT5G08620, AT2G39800, AT1G78080, AT1G12610, AT3G06010, AT3G63060, AT4G33950, AT5G56030, AT3G51920, AT4G17615, AT3G54770, AT4G01420, AT2G42540, AT4G39090, AT5G25370, AT3G20250, AT1G15690, AT3G23250, AT3G47600, AT4G24190, AT1G10370	208	484	18499	7.901472	8.04E-23	2.01E-23	2.31E-22
water	GO:0006970~response to osmotic stress	28	13.27	1.24E-27	AT3G62770, AT4G18780, AT5G66880, AT1G32230, AT1G32560, AT1G02730,	208	122	18499	20.41189	6.23E-25	2.08E-25	1.79E-24

					AT1G69310, AT3G46930, AT3G50500, AT2G31870, AT2G17820, AT2G37180, AT1G78080, AT1G20440, AT3G12630, AT3G63060, AT4G33950, AT4G17615, AT4G01420, AT2G42540, AT5G06760, AT4G39090, AT5G57050, AT2G35300, AT3G20250, AT2G40220, AT5G67030, AT3G14440							
water	GO:0009737~response to abscisic acid	45	21.33	2.30E-31	AT1G20450, AT2G37170, AT5G48870, AT2G31470, AT3G26090, AT4G35100, AT5G11270, AT1G16060, AT3G22380, AT5G66880, AT1G17950, AT1G45249, AT5G59550, AT3G53420, AT5G03740, AT5G62470, AT3G46930, AT3G19600, AT3G05700, AT3G46620, AT5G08490, AT1G27730, AT4G27410, AT5G40280, AT3G18490, AT3G50500, AT2G18960, AT2G39800, AT3G20310, AT2G39550, AT1G20440, AT1G70670, AT3G63060, AT4G33950, AT3G51920, AT3G54770, AT2G42540, AT4G23450, AT5G57050, AT5G52050, AT5G25370, AT1G13740, AT1G12480, AT3G47600, AT1G80710	208	394	18499	10.15784	1.15E-28	5.77E-29	3.32E-28
water	GO:0009414~response to water deprivation	142	67.30	2.16E-221	AT1G74920, AT1G20450, AT2G37170, AT4G31920, AT1G15360, AT3G52450, AT1G11755, AT1G16060, AT1G52890, AT1G45249, AT5G63980, AT3G61050, AT5G47100, AT4G25480, AT3G16857, AT5G45340, AT1G27730, AT3G18490, AT3G50310, AT3G50500, AT5G07690, AT1G04400, AT4G24275, AT3G29320, AT2G37180, AT3G11020, AT2G30580, AT1G78080, AT2G39550, AT5G13780, AT4G17615, AT4G39090, AT1G06770, AT3G61430, AT3G30775, AT1G52340, AT4G34890, AT4G08920, AT2G31470, AT1G28520, AT5G66880, AT1G76080, AT3G45140, AT3G46930, AT5G08490, AT1G01620, AT5G40280, AT1G05850, AT3G59380, AT2G31870, AT2G33230, AT5G64010, AT2G17820, AT3G20310, AT2G39800, AT1G20440, AT3G63060, AT2G38880, AT4G33950, AT1G47128, AT5G08120, AT5G60410, AT1G15690, AT5G67030, AT1G69600, AT3G56580, AT4G24190, AT1G58440, AT3G14080, AT5G27620, AT5G11270, AT3G22380, AT2G04030, AT4G18780, AT2G40880, AT4G00430, AT5G06530, AT5G03740, AT5G13750, AT5G62470, AT3G05700, AT1G33240, AT2G25180, AT3G46620, AT3G23050, AT2G18960, AT2G29130, AT1G12610, AT2G35930, AT2G47800, AT5G56030, AT3G51920, AT5G57050, AT2G35300, AT1G13740, AT2G40220, AT1G08720, AT3G12490, AT1G19120, AT1G80710, AT1G80410, AT5G05410, AT1G46768, AT3G62770, AT5G48870, AT3G26090, AT1G33560, AT3G15500, AT4G26070, AT4G24020, AT2G45960, AT2G42620, AT1G32230, AT5G59550, AT3G53420, AT1G32560, AT1G69310, AT1G02730, AT2G26650, AT3G19600, AT5G44650, AT3G22310, AT1G30100, AT4G27410, AT1G05180, AT3G46970, AT5G08620, AT1G30270, AT4G34100, AT3G06010, AT1G02205, AT3G12630, AT3G54770, AT4G01420, AT2G42540, AT4G23450, AT5G06760, AT5G25370, AT3G14440, AT1G12110, AT2G05620, AT1G54160	208	279	18499	45.26568	1.08E-218	1.08E-218	3.12E-218
Cellular component												
cold	GO:0009535~chloroplast thylakoid membrane	10	6.94	4.26E-04	AT3G52150, AT5G52440, AT2G33800, AT1G77490, AT1G29395, AT4G24770, AT1G29390, AT2G37230, AT2G01918, AT4G39730	139	407	25147	4.445053	3.60E-02	1.22E-02	4.59E-01
cold	GO:0009507~chloroplast	38	26.39	3.54E-04	AT3G08920, AT3G52150, AT2G33800, AT4G28210, AT1G01860, AT1G70200, AT2G42530, AT3G53460, AT1G05140, AT5G63980, AT3G11170, AT1G74960, AT5G62390, AT5G10450, AT5G52440, AT2G21660, AT5G38480, AT1G56070, AT5G50950, AT1G74710, AT1G77490, AT5G65430, AT5G52370, AT2G38170, AT1G10760, AT1G20823, AT5G23070, AT1G29395, AT3G06510, AT2G42540, AT2G36530, AT5G15090, AT4G24770, AT3G22690, AT3G20930, AT3G55160, AT4G39730, AT2G01918	139	3855	25147	1.783325	3.00E-02	1.51E-02	3.82E-01
cold	GO:0009941~chloroplast envelope	14	9.72	9.71E-06	AT3G08920, AT3G52150, AT4G31690, AT1G10760, AT2G19450, AT1G29395, AT3G06510, AT2G42530, AT2G42540, AT1G29390, AT3G11170, AT5G15090, AT5G52440, AT4G24770	139	543	25147	4.664441	8.35E-04	8.35E-04	1.05E-02
heat	GO:0005635~nuclear envelope	4	3.92	3.35E-03	AT3G53110, AT5G61780, AT5G07350, AT3G10800	101	75	25147	13.27894	2.30E-01	4.27E-02	3.49E+00

heat	GO:0005634~nucleus	55	53.92	1.59E-03	AT4G11260, AT4G29770, AT5G47910, AT4G26850, AT2G16575, AT1G64280, AT5G63870, AT5G07100, AT5G21160, AT3G09350, AT3G06400, AT1G03190, AT5G16820, AT2G20880, AT1G12610, AT1G79350, AT4G21320, AT3G47220, AT4G15802, AT1G21760, AT4G38630, AT5G05410, AT5G59820, AT3G53110, AT3G24320, AT1G64520, AT3G28030, AT1G17780, AT1G54490, AT2G21320, AT5G03280, AT1G28520, AT3G25230, AT4G12400, AT2G26150, AT4G29040, AT1G73130, AT2G17690, AT4G26080, AT2G38470, AT5G02500, AT3G16770, AT4G26840, AT2G30250, AT5G17020, AT3G06010, AT2G46020, AT5G42020, AT5G60410, AT1G74310, AT4G19020, AT2G39770, AT5G53060, AT5G48570, AT3G10800	101	9796	25147	1.397908	1.17E-01	2.45E-02	1.67E+00
heat	GO:0000932~cytoplasmic mRNA processing body	4	3.92	1.11E-03	AT5G21160, AT5G61780, AT1G54490, AT5G07350	101	51	25147	19.52786	8.27E-02	2.14E-02	1.16E+00
heat	GO:0010494~cytoplasmic stress granule	4	3.92	7.70E-05	AT5G21160, AT5G61780, AT1G54490, AT5G07350	101	21	25147	47.4248	5.99E-03	2.00E-03	8.15E-02
heat	GO:0005737~cytoplasm	39	38.24	9.43E-07	AT4G11260, AT3G53110, AT4G29770, AT4G26850, AT5G43940, AT1G28520, AT3G25230, AT5G63870, AT1G64280, AT4G12400, AT5G21160, AT2G26150, AT3G09350, AT4G04950, AT4G29040, AT3G17880, AT3G53990, AT2G17690, AT5G42980, AT4G26080, AT5G16820, AT5G02500, AT3G16770, AT5G56010, AT1G16540, AT4G26840, AT2G20880, AT5G17020, AT5G53400, AT5G58070, AT1G74310, AT2G39770, AT5G61780, AT5G07350, AT1G21760, AT4G38630, AT5G10010, AT3G10800, AT1G71790	101	4407	25147	2.203365	7.36E-05	3.68E-05	9.98E-04
heat	GO:0005829~cytosol	29	28.43	5.46E-08	AT4G11260, AT3G53110, AT1G64520, AT5G43940, AT1G54490, AT3G25230, AT5G21160, AT4G04950, AT3G06400, AT4G29040, AT5G62390, AT5G42980, AT5G02500, AT5G56010, AT4G26840, AT5G17020, AT1G79350, AT2G46020, AT5G42020, AT1G50500, AT5G53400, AT5G58070, AT1G74310, AT5G61780, AT2G39770, AT4G15802, AT5G07350, AT4G38630, AT1G52340	101	2309	25147	3.127079	4.26E-06	4.26E-06	5.78E-05
light	GO:0030076~light-harvesting complex	3	2.11	5.40E-03	AT2G05100, AT2G05070, AT3G27690	140	20	25147	26.94321	3.37E-01	3.37E-02	5.54E+00
light	GO:0009941~chloroplast envelope	10	7.04	3.29E-03	AT2G05100, AT3G62030, AT4G37270, AT5G64940, AT2G05070, AT5G58070, AT3G27690, AT1G55480, AT4G13670, AT5G58140	140	543	25147	3.307945	2.21E-01	2.25E-02	3.40E+00
light	GO:0009534~chloroplast thylakoid	7	4.93	1.02E-03	AT5G16400, AT2G05070, AT1G55480, AT4G13670, AT2G05620, AT1G03600, AT3G02730	140	206	25147	6.103641	7.48E-02	7.75E-03	1.07E+00
light	GO:0009579~thylakoid	7	4.93	1.02E-03	AT2G05100, AT3G62030, AT4G22260, AT2G05070, AT3G27690, AT1G55480, AT1G03600	140	206	25147	6.103641	7.48E-02	7.75E-03	1.07E+00
light	GO:0010287~plastoglobule	5	3.52	8.02E-04	AT2G05100, AT4G31390, AT2G05070, AT3G27690, AT1G79600	140	75	25147	11.97476	5.91E-02	7.59E-03	8.40E-01
light	GO:0009898~cytoplasmic side of plasma membrane	3	2.11	8.31E-04	AT3G45780, AT5G58070, AT5G58140	140	8	25147	67.35804	6.12E-02	7.00E-03	8.71E-01
light	GO:0016605~PML body	3	2.11	1.81E-04	AT4G08920, AT2G46340, AT1G04400	140	4	25147	134.7161	1.36E-02	1.96E-03	1.90E-01
light	GO:0009535~chloroplast thylakoid membrane	11	7.75	9.22E-05	AT2G05100, AT3G62030, AT4G22260, AT2G05070, AT3G27690, AT3G47860, AT1G55480, AT4G13670, AT2G05620, AT1G03600, AT5G55280	140	407	25147	4.854633	6.98E-03	1.17E-03	9.70E-02
light	GO:0005622~intracellular	15	10.56	1.18E-05	AT3G21150, AT4G16250, AT1G09570, AT1G10470, AT5G63980, AT1G20090, AT3G45780, AT2G24790, AT1G42550, AT3G04110, AT3G48100, AT1G59940, AT5G58140, AT4G17490, AT2G18790	140	634	25147	4.249718	8.94E-04	1.79E-04	1.24E-02
light	GO:0016607~nuclear speck	7	4.93	2.51E-06	AT5G61270, AT1G07350, AT2G46340, AT5G02200, AT2G18790, AT1G09570, AT4G25560	140	70	25147	17.96214	1.91E-04	6.36E-05	2.64E-03
light	GO:0005634~nucleus	83	58.45	1.32E-06	AT2G21150, AT4G18290, AT1G26260, AT1G21970, AT3G21150, AT4G16250, AT2G36890, AT5G43630, AT4G28860, AT1G05630, AT1G10120, AT5G27620, AT4G14110, AT2G36990, AT5G63870, AT1G09570, AT2G21650, AT1G22770, AT5G63980, AT1G68050, AT1G20090, AT4G27430, AT4G36930, AT3G59220, AT5G48560, AT1G59940, AT2G46260, AT5G58140, AT1G10170, AT5G07690, AT2G18790, AT1G04400, AT5G49230, AT2G37678, AT1G53090, AT5G57360, AT2G46590, AT1G09530, AT1G25540, AT4G34530, AT2G36530, AT4G25560, AT5G64330, AT5G28490, AT5G20730, AT4G28610, AT4G15090, AT5G59820, AT1G26945, AT4G28880, AT2G20180, AT4G08920, AT2G43010, AT2G21320, AT2G42620, AT3G02790, AT4G01120, AT3G05420, AT3G45780, AT3G61850, AT2G24790, AT4G40060, AT1G78370, AT3G54610, AT1G26830, AT2G46340,	140	9796	25147	1.521905	1.01E-04	5.03E-05	1.39E-03

					AT4G28556, AT2G40080, AT3G44450, AT2G39840, AT3G61600, AT1G70800, AT1G02340, AT5G02200, AT2G46370, AT1G10470, AT3G19820, AT5G39760, AT5G61270, AT3G48100, AT5G61230, AT4G17490, AT1G54160							
light	GO:0005737~cytoplasm	48	33.80	2.63E-06	AT4G28880, AT1G26945, AT4G08920, AT3G21150, AT4G28860, AT5G27620, AT1G05630, AT4G14110, AT1G75100, AT5G55280, AT5G63870, AT4G01120, AT3G02790, AT1G09570, AT1G22770, AT1G68050, AT5G63980, AT1G20090, AT3G05420, AT2G26300, AT3G45780, AT4G38740, AT1G59940, AT2G46260, AT1G78370, AT5G58140, AT4G28556, AT2G38050, AT1G04400, AT2G37678, AT2G39840, AT3G61600, AT3G08550, AT5G57360, AT1G31812, AT2G47700, AT5G27630, AT4G40100, AT5G02200, AT2G36530, AT2G46370, AT5G17880, AT1G10470, AT5G58070, AT5G38150, AT3G48100, AT5G08560, AT3G47340	140	4407	25147	1.956394	2.00E-04	5.00E-05	2.77E-03
light	GO:0016604~nuclear body	6	4.23	2.82E-08	AT2G37678, AT4G08920, AT2G46340, AT2G18790, AT1G09570, AT1G04400	140	17	25147	63.3958	2.14E-06	2.14E-06	2.96E-05
osmotic	GO:0005886~plasma membrane	31	27.19	5.41E-04	AT1G55180, AT1G27320, AT5G03280, AT1G60940, AT4G18780, AT1G65690, AT1G72180, AT5G01410, AT1G42550, AT5G45800, AT1G77120, AT3G50500, AT3G17510, AT2G17820, AT2G37180, AT2G22660, AT5G13170, AT4G04340, AT2G47900, AT4G17615, AT4G01420, AT5G58580, AT1G50500, AT5G62460, AT2G43850, AT5G19690, AT1G58200, AT5G35750, AT4G08500, AT1G15100, AT1G35720	112	3702	25147	1.880154	3.24E-02	8.21E-03	5.42E-01
osmotic	GO:0005634~nucleus	63	55.26	2.23E-04	AT1G01510, AT2G01150, AT1G60940, AT5G52310, AT4G11230, AT3G58620, AT5G62090, AT3G50500, AT5G01270, AT4G25520, AT1G78290, AT4G16830, AT5G08590, AT3G56400, AT2G47900, AT2G40750, AT2G18250, AT1G10940, AT4G39090, AT2G32700, AT5G57630, AT2G40220, AT2G43790, AT4G04920, AT4G08500, AT1G35720, AT3G33520, AT1G43700, AT5G03280, AT3G25230, AT5G49450, AT5G66880, AT3G59770, AT1G32230, AT3G46930, AT1G69310, AT3G45640, AT2G21660, AT2G23030, AT5G63650, AT3G17510, AT1G55870, AT2G31870, AT3G24860, AT5G35550, AT3G54560, AT3G08730, AT2G22660, AT1G20440, AT5G24660, AT1G53300, AT3G63060, AT3G12630, AT4G33950, AT2G42580, AT4G34710, AT4G01420, AT5G58580, AT4G01370, AT4G40010, AT5G53060, AT2G35510, AT1G15100	112	9796	25147	1.443976	1.35E-02	6.77E-03	2.24E-01
osmotic	GO:0005737~cytoplasm	36	31.58	2.29E-04	AT3G33520, AT2G01150, AT5G12030, AT4G37900, AT3G25230, AT5G66880, AT3G59770, AT1G32230, AT5G52310, AT5G01410, AT2G21660, AT3G45640, AT1G64670, AT2G23030, AT1G77120, AT5G63650, AT3G50500, AT5G01270, AT3G17510, AT1G78290, AT3G08730, AT4G16830, AT1G16540, AT5G08590, AT2G22660, AT4G33950, AT4G17615, AT4G01420, AT4G01370, AT5G62460, AT4G40010, AT5G57630, AT3G20250, AT5G08560, AT1G35910, AT1G15100	112	4407	25147	1.834119	1.39E-02	4.64E-03	2.30E-01
osmotic	GO:0005829~cytosol	28	24.56	1.99E-06	AT1G01510, AT5G12030, AT1G43700, AT1G60940, AT3G25230, AT5G66880, AT1G32560, AT1G65690, AT5G01410, AT2G21660, AT1G42550, AT2G23030, AT1G77120, AT3G50500, AT1G20440, AT2G47900, AT4G33950, AT2G18250, AT1G10940, AT5G06760, AT4G01370, AT1G50500, AT2G35300, AT5G57630, AT3G20250, AT4G40010, AT3G12490, AT1G35720	112	2309	25147	2.722715	1.22E-04	1.22E-04	2.00E-03
oxidative	GO:0005737~cytoplasm	39	25.83	7.16E-03	AT2G41090, AT4G25100, AT3G06930, AT5G20140, AT3G22200, AT1G49670, AT2G19310, AT1G30460, AT1G75280, AT1G08830, AT1G32230, AT5G63980, AT3G11220, AT4G17070, AT5G01410, AT3G53990, AT3G45640, AT5G09830, AT1G14870, AT3G09640, AT4G11850, AT5G28030, AT3G11050, AT5G59880, AT5G25620, AT2G40000, AT2G39800, AT5G16990, AT1G02930, AT5G16970, AT1G09000, AT2G14170, AT1G13440, AT1G13340, AT5G58070, AT3G04120, AT1G35910, AT4G10090, AT3G06110	148	4407	25147	1.503647	3.82E-01	4.28E-02	7.11E+00
oxidative	GO:0009570~chloroplast stroma	12	7.95	1.63E-03	AT1G76080, AT5G63980, AT2G01140, AT3G62030, AT4G25100, AT4G25130, AT2G05710, AT4G03520, AT2G28190, AT1G03680, AT3G15360, AT1G35720	148	653	25147	3.122429	1.03E-01	1.09E-02	1.66E+00
oxidative	GO:0005783~endoplasmic reticulum	13	8.61	8.97E-04	AT1G21520, AT5G60640, AT1G09210, AT5G42000, AT1G56340, AT5G58070, AT4G04800, AT1G52760, AT1G01230,	148	703	25147	3.142046	5.83E-02	6.66E-03	9.16E-01

					AT3G12490, AT1G27330, AT1G07380, AT5G57345							
oxidative	GO:0033588~Elongator holoenzyme complex	3	1.99	6.99E-04	AT3G11220, AT5G13680, AT4G10090	148	7	25147	72.8195	4.58E-02	5.84E-03	7.15E-01
oxidative	GO:0005774~vacuolar membrane	12	7.95	3.25E-04	AT5G08670, AT1G49300, AT5G60640, AT4G35000, AT1G56340, AT5G58070, AT2G05710, AT1G07380, AT3G04120, AT3G22200, AT1G35720, AT3G14990	148	536	25147	3.804004	2.15E-02	3.62E-03	3.33E-01
oxidative	GO:0009941~chloroplast envelope	12	7.95	3.62E-04	AT1G76080, AT3G62030, AT4G25100, AT5G64940, AT4G35000, AT2G01980, AT5G58070, AT4G25130, AT5G20140, AT4G00290, AT1G03680, AT3G15360	148	543	25147	3.754965	2.40E-02	3.46E-03	3.71E-01
oxidative	GO:0009579~thylakoid	8	5.30	2.16E-04	AT3G62030, AT4G25100, AT1G32220, AT4G03520, AT2G28190, AT1G03680, AT3G15360, AT1G35720	148	206	25147	6.598531	1.44E-02	2.89E-03	2.22E-01
oxidative	GO:0005623~cell	9	5.96	1.80E-05	AT1G76080, AT5G60640, AT3G06050, AT5G42000, AT1G01230, AT1G07380, AT4G03520, AT1G03680, AT3G15360	148	192	25147	7.964633	1.21E-03	3.02E-04	1.85E-02
oxidative	GO:0009507~chloroplast	47	31.13	8.52E-07	AT1G50170, AT3G62030, AT5G60640, AT4G25100, AT1G56340, AT4G11010, AT5G20140, AT1G03680, AT4G26970, AT5G08670, AT1G76080, AT4G35090, AT5G63980, AT1G31170, AT4G35000, AT4G08940, AT5G18100, AT3G09640, AT3G47860, AT3G47450, AT4G02380, AT3G11050, AT2G28190, AT4G11830, AT5G59880, AT4G03240, AT5G43750, AT3G07700, AT5G07460, AT2G01140, AT1G09210, AT2G39800, AT1G32220, AT1G52760, AT4G25130, AT2G05710, AT4G03520, AT4G00290, AT3G15360, AT1G66330, AT1G13440, AT5G64940, AT5G37510, AT3G04120, AT1G35910, AT3G14990, AT1G35720	148	3855	25147	2.071562	5.71E-05	2.85E-05	8.74E-04
oxidative	GO:0005829~cytosol	34	22.52	1.09E-06	AT4G25100, AT1G56340, AT4G11010, AT3G06930, AT1G50290, AT3G22200, AT1G08830, AT4G26970, AT1G69190, AT5G08670, AT4G35090, AT5G63980, AT5G01410, AT4G35000, AT5G09830, AT3G09640, AT2G47510, AT5G59880, AT5G47650, AT5G07460, AT1G63460, AT3G25530, AT5G16990, AT1G02930, AT2G05710, AT4G03520, AT5G13680, AT5G16970, AT1G13440, AT5G58070, AT3G12490, AT3G04120, AT1G35720, AT3G14990	148	2309	25147	2.501955	7.33E-05	2.44E-05	1.12E-03
oxidative	GO:0005739~mitochondrion	52	34.44	5.99E-11	AT5G60640, AT4G25100, AT2G21195, AT1G49670, AT2G24150, AT1G69190, AT5G08670, AT3G16890, AT4G35090, AT5G63980, AT3G06050, AT4G35000, AT2G47510, AT4G02380, AT4G03240, AT5G59880, AT2G01140, AT4G02580, AT3G25530, AT1G32220, AT1G01230, AT2G21640, AT2G14170, AT5G58070, AT1G35720, AT3G10930, AT3G62030, AT1G56340, AT5G42000, AT4G11010, AT1G50290, AT3G22200, AT4G26970, AT1G31170, AT5G19875, AT3G47450, AT3G14430, AT5G18040, AT2G40000, AT1G09210, AT2G39800, AT4G25130, AT1G27330, AT1G02930, AT2G05710, AT4G00290, AT3G60980, AT1G13440, AT5G37510, AT3G04120, AT5G55070, AT3G14990	148	3406	25147	2.594079	4.02E-09	4.02E-09	6.15E-08
salt	GO:0005886~plasma membrane	51	22.08	2.18E-03	AT4G32150, AT1G51460, AT3G12360, AT5G40770, AT3G46550, AT1G60940, AT3G47950, AT3G61050, AT2G32010, AT5G14920, AT3G50500, AT3G17980, AT5G60920, AT1G04120, AT4G24500, AT3G05360, AT2G47900, AT3G51920, AT4G17615, AT5G27150, AT2G01980, AT5G58070, AT5G07350, AT3G06370, AT3G44110, AT2G45960, AT4G10310, AT5G59010, AT1G51500, AT5G03280, AT1G17840, AT1G13930, AT1G65690, AT1G49300, AT2G26650, AT5G01410, AT4G29810, AT2G33080, AT3G17510, AT3G45680, AT5G35410, AT4G01420, AT2G27300, AT5G58580, AT4G23650, AT5G62460, AT1G15690, AT5G19690, AT4G24190, AT1G15100, AT4G39730	229	3702	25147	1.51281	1.89E-01	2.95E-02	2.38E+00
salt	GO:0000325~plant-type vacuole	5	2.16	4.66E-04	AT1G53210, AT1G04120, AT5G27150, AT1G15690, AT2G41560	229	40	25147	13.72653	4.37E-02	8.90E-03	5.12E-01
salt	GO:0009897~external side of plasma membrane	3	1.30	4.85E-04	AT5G60920, AT1G17840, AT3G46550	229	4	25147	82.35917	4.55E-02	7.74E-03	5.33E-01
salt	GO:0005773~vacuole	17	7.36	2.18E-04	AT1G53210, AT1G04120, AT2G38170, AT4G32150, AT2G45960, AT5G40770, AT2G41560, AT1G64460, AT5G11150, AT5G22360, AT4G23650, AT5G27150, AT3G20250, AT1G15690, AT5G58070, AT4G24190, AT4G39730	229	629	25147	2.967898	2.07E-02	5.21E-03	2.39E-01
salt	GO:0005774~vacuolar membrane	19	8.23	2.17E-06	AT1G53210, AT1G04120, AT2G38170, AT3G51390, AT4G32150, AT5G40770, AT2G04030, AT3G22200, AT2G41560, AT5G14040, AT5G11150, AT3G48680, AT1G49300, AT5G27150, AT1G15690, AT5G58070, AT4G24190, AT3G06370, AT4G39730	229	536	25147	3.892598	2.09E-04	6.95E-05	2.39E-03

salt	GO:0005634~nucleus	127	54.98	4.08E-07	AT4G06634, AT1G16060, AT3G06930, AT3G12360, AT1G60940, AT5G26742, AT3G49810, AT5G63980, AT1G43160, AT1G27730, AT1G73500, AT5G62090, AT2G01900, AT2G47460, AT3G50500, AT4G25520, AT1G10170, AT1G78310, AT3G17980, AT2G26430, AT5G46350, AT4G16830, AT2G37678, AT1G73660, AT4G24500, AT3G55270, AT2G18250, AT1G10940, AT3G51960, AT2G43790, AT3G03450, AT4G38630, AT5G01520, AT5G08450, AT1G10570, AT2G19450, AT3G57180, AT5G03280, AT1G51500, AT1G14920, AT5G66880, AT3G46930, AT3G61890, AT3G17510, AT1G55870, AT1G14350, AT3G27460, AT5G05660, AT2G30250, AT1G53300, AT3G63060, AT1G31470, AT4G33950, AT5G26751, AT1G10880, AT2G27300, AT4G01370, AT4G40010, AT3G57480, AT1G66350, AT3G51780, AT2G01430, AT5G08520, AT2G39770, AT1G60220, AT4G14300, AT4G24190, AT1G67580, AT1G15100, AT1G01510, AT3G09350, AT5G37370, AT1G03060, AT2G32010, AT5G03740, AT1G28380, AT4G26630, AT3G05700, AT5G67300, AT4G22330, AT2G47580, AT5G08590, AT1G06040, AT1G12610, AT2G47900, AT5G13330, AT5G56030, AT3G51920, AT2G26190, AT4G21670, AT1G66230, AT2G32700, AT5G57630, AT3G23250, AT1G27760, AT3G51390, AT5G49450, AT1G56600, AT1G17950, AT1G32230, AT1G69310, AT2G33700, AT3G22310, AT5G40550, AT5G24240, AT5G63650, AT2G38470, AT2G45640, AT5G04760, AT1G21610, AT5G08620, AT5G02020, AT2G01570, AT5G63110, AT3G06010, AT5G35410, AT3G06590, AT4G34710, AT3G54770, AT4G01420, AT1G64460, AT5G58580, AT4G37260, AT4G23650, AT2G38340, AT2G35510, AT2G41010	229	9796	25147	1.423658	3.92E-05	1.96E-05	4.49E-04
salt	GO:0005829~cytosol	48	20.78	1.04E-07	AT1G74920, AT1G01510, AT3G44110, AT4G24800, AT3G48330, AT3G06930, AT2G04030, AT1G60940, AT3G22200, AT5G66880, AT2G46500, AT5G63980, AT4G26630, AT1G65690, AT5G01410, AT5G24240, AT3G50500, AT3G17980, AT3G23940, AT2G47580, AT4G24500, AT3G55270, AT2G47900, AT4G33950, AT5G56030, AT3G51920, AT2G18250, AT5G26751, AT1G10940, AT4G01370, AT4G23650, AT4G40010, AT5G57630, AT3G20250, AT3G51780, AT1G15690, AT5G58070, AT4G14300, AT2G39770, AT5G61780, AT1G30580, AT5G07350, AT4G24190, AT4G38630, AT1G10370, AT1G67580, AT5G01520, AT4G39730	229	2309	25147	2.282801	9.97E-06	9.97E-06	1.14E-04
water	GO:0009506~plasmodesma	19	9.00	1.77E-03	AT2G37170, AT1G20450, AT2G37180, AT2G18960, AT3G54820, AT3G16240, AT4G35100, AT2G47800, AT3G16640, AT2G45960, AT5G56030, AT1G47128, AT4G00430, AT3G53420, AT1G32560, AT3G61430, AT1G80410, AT1G01620, AT4G24190	208	1008	25147	2.278851	1.55E-01	2.77E-02	1.93E+00
water	GO:0042807~central vacuole	3	1.42	2.91E-03	AT3G16240, AT2G36830, AT4G01470	208	10	25147	36.26971	2.42E-01	2.73E-02	3.15E+00
water	GO:0009941~chloroplast envelope	13	6.16	1.90E-03	AT3G16240, AT3G52150, AT2G04030, AT2G42540, AT2G20890, AT1G76080, AT3G45140, AT1G32080, AT3G61430, AT5G52440, AT1G15690, AT5G67030, AT2G36830	208	543	25147	2.894452	1.66E-01	2.55E-02	2.07E+00
water	GO:0005886~plasma membrane	47	22.27	2.43E-03	AT2G37170, AT3G26090, AT4G35100, AT2G45960, AT4G18780, AT4G00430, AT1G18390, AT3G61050, AT5G06530, AT3G53420, AT5G47100, AT5G13750, AT2G41225, AT2G26650, AT1G21270, AT1G32080, AT1G07000, AT5G52440, AT3G10500, AT5G14420, AT1G01620, AT3G50500, AT1G05180, AT5G58430, AT2G18960, AT2G17820, AT2G37180, AT3G54820, AT3G16240, AT1G30270, AT4G31390, AT2G47800, AT3G51920, AT3G16640, AT4G17615, AT4G01420, AT5G52050, AT3G61430, AT1G15690, AT1G12480, AT3G01650, AT1G08720, AT1G12110, AT4G24190, AT1G79600, AT1G60200, AT4G39730	208	3702	25147	1.534915	2.07E-01	2.54E-02	2.64E+00
water	GO:0005783~endoplasmic reticulum	15	7.11	2.15E-03	AT2G17820, AT5G50430, AT1G58440, AT3G17000, AT2G41230, AT1G67360, AT5G18290, AT3G61050, AT1G08720, AT3G04090, AT3G12490, AT4G24190, AT3G18490, AT1G17280, AT4G39730	208	703	25147	2.579638	1.85E-01	2.53E-02	2.34E+00
water	GO:0005811~lipid particle	4	1.90	4.09E-04	AT1G70670, AT1G67360, AT3G05500, AT2G47780	208	18	25147	26.86645	3.82E-02	7.75E-03	4.49E-01
water	GO:0005634~nucleus	108	51.18	1.28E-04	AT1G20450, AT4G31920, AT2G28625, AT1G15360, AT1G16060, AT1G52890,	208	9796	25147	1.332901	1.21E-02	3.04E-03	1.41E-01

					AT1G45249, AT1G18390, AT5G63980, AT3G16857, AT4G25480, AT1G07000, AT1G27730, AT3G50500, AT5G07690, AT1G04400, AT4G24275, AT2G30580, AT3G11020, AT1G78080, AT2G39550, AT3G56400, AT3G17000, AT2G41230, AT1G06770, AT4G39090, AT3G47600, AT5G18320, AT4G08920, AT2G31470, AT1G28520, AT5G66880, AT3G46930, AT2G46400, AT3G10500, AT3G13672, AT3G59380, AT1G14350, AT2G31870, AT2G33230, AT4G03260, AT5G64010, AT3G16800, AT3G20310, AT1G20440, AT3G63060, AT2G38880, AT4G33950, AT5G08120, AT5G60410, AT1G36060, AT4G14300, AT4G24190, AT1G69600, AT3G14080, AT4G35100, AT5G27620, AT5G11270, AT3G22380, AT5G64960, AT5G03740, AT5G62470, AT3G05700, AT1G33240, AT2G25180, AT3G46620, AT3G23050, AT2G18960, AT1G12610, AT2G40750, AT5G56030, AT3G51920, AT1G13740, AT3G23250, AT2G40220, AT1G08720, AT1G80710, AT1G80410, AT1G19120, AT5G05410, AT1G46768, AT3G62770, AT1G22810, AT5G48870, AT3G26090, AT3G15500, AT4G24020, AT2G42620, AT1G17950, AT1G32230, AT5G59550, AT1G69310, AT3G19600, AT3G22310, AT4G27410, AT5G14420, AT1G05180, AT5G08620, AT5G09410, AT1G30270, AT3G06010, AT3G12630, AT3G16640, AT3G54770, AT4G01420, AT4G23450, AT3G53600, AT1G54160							
water	GO:0005829~cytosol	43	20.38	7.55E-07	AT1G74920, AT1G20450, AT3G62770, AT4G34890, AT3G52450, AT2G04030, AT5G64960, AT1G52690, AT5G66880, AT5G59550, AT1G32560, AT5G63980, AT1G07000, AT3G46620, AT3G50500, AT1G05180, AT3G46970, AT3G29320, AT5G58430, AT1G30270, AT1G20440, AT2G35930, AT5G13780, AT4G33950, AT5G56030, AT3G51920, AT3G16640, AT5G13030, AT1G47128, AT5G06760, AT5G58787, AT2G35300, AT3G20250, AT1G15690, AT1G08720, AT4G14300, AT3G12490, AT1G80410, AT4G24190, AT1G07240, AT1G10370, AT4G39730, AT1G52340	208	2309	25147	2.251476	7.17E-05	2.39E-05	8.30E-04
water	GO:0005773~vacuole	22	10.43	6.39E-08	AT2G37170, AT2G18960, AT2G37180, AT3G54820, AT3G16240, AT4G35100, AT2G47800, AT2G45960, AT1G67360, AT1G47128, AT4G00430, AT4G39090, AT3G53420, AT3G61430, AT3G20250, AT1G15690, AT2G36830, AT1G01620, AT4G24190, AT3G05500, AT1G04400, AT4G39730	208	629	25147	4.228583	6.07E-06	3.03E-06	7.02E-05
water	GO:0005887~integral component of plasma membrane	16	7.58	1.00E-08	AT2G37170, AT2G37180, AT2G18960, AT3G54820, AT3G16240, AT4G35100, AT2G45960, AT5G18290, AT4G00430, AT3G53420, AT2G26650, AT3G61430, AT2G36830, AT3G04090, AT1G01620, AT4G01470	208	275	25147	7.034126	9.54E-07	9.54E-07	1.10E-05
**Molecular function**												
cold	GO:0000166~nucleotide binding	11	7.64	2.00E-03	AT1G06960, AT3G52150, AT1G60650, AT2G21660, AT5G04280, AT4G13850, AT1G70200, AT4G24770, AT3G20930, AT3G26420, AT3G53460	122	504	18171	3.250732	2.62E-01	4.25E-02	2.37E+00
cold	GO:0003676~nucleic acid binding	13	9.03	8.26E-04	AT5G59820, AT2G33835, AT3G52150, AT5G04280, AT4G13850, AT1G70200, AT4G36020, AT3G53460, AT2G17870, AT1G60650, AT2G21660, AT4G38680, AT4G24770	122	615	18171	3.148381	1.18E-01	2.07E-02	9.84E-01
cold	GO:0003729~mRNA binding	10	6.94	5.72E-04	AT2G21660, AT1G56070, AT5G04280, AT4G38680, AT4G24770, AT4G36020, AT3G26420, AT2G37230, AT3G53460, AT2G17870	122	351	18171	4.24338	8.33E-02	1.72E-02	6.83E-01
cold	GO:0003677~DNA binding	28	19.44	3.66E-04	AT3G07740, AT5G62470, AT4G25480, AT4G36930, AT5G64220, AT4G38680, AT3G26420, AT2G46830, AT4G16420, AT3G61950, AT5G09410, AT2G33835, AT2G46810, AT2G40970, AT4G31690, AT2G46590, AT5G04280, AT1G01060, AT2G36530, AT2G17870, AT1G60650, AT3G23250, AT4G25470, AT4G25490, AT3G49530, AT4G08500, AT4G16150, AT1G46768	122	2047	18171	2.03732	5.41E-02	1.38E-02	4.37E-01
cold	GO:0003723~RNA binding	17	11.81	5.11E-05	AT3G52150, AT3G53110, AT3G14080, AT5G04280, AT1G01860, AT4G13850, AT3G21300, AT4G36020, AT3G53460, AT2G17870, AT1G06960, AT1G60650, AT2G21660, AT1G19120, AT4G24770, AT3G20930, AT3G26420	122	769	18171	3.29262	7.73E-03	2.58E-03	6.11E-02
cold	GO:0005515~protein binding	30	20.83	1.49E-05	AT5G54590, AT3G14080, AT2G39810, AT1G60170, AT1G27320, AT1G22770, AT4G12470, AT5G62390, AT5G10450, AT4G36930, AT4G29810, AT5G50950,	122	1901	18171	2.350489	2.27E-03	1.13E-03	1.79E-02

					AT4G26080, AT5G53470, AT2G18790, AT2G46830, AT4G16420, AT3G55580, AT5G20230, AT4G24500, AT1G10760, AT3G04740, AT2G17870, AT4G01370, AT5G15090, AT1G19120, AT2G43790, AT5G35750, AT1G33410, AT4G08500							
cold	GO:0001077~transcriptional activator activity, RNA polymerase II core promoter proximal region sequence-specific binding	4	2.78	5.68E-06	AT5G09410, AT2G22300, AT5G64220, AT4G16150	122	6	18171	99.29508	8.63E-04	8.63E-04	6.79E-03
heat	GO:0043565~sequence-specific DNA binding	12	11.76	6.06E-04	AT2G26150, AT5G59820, AT2G20880, AT2G30250, AT1G12610, AT2G38470, AT5G16820, AT1G28520, AT3G10800, AT5G05410, AT5G07100, AT3G16770	93	676	18171	3.46841	9.30E-02	4.76E-02	7.30E-01
heat	GO:0008094~DNA-dependent ATPase activity	4	3.92	7.31E-04	AT3G06400, AT3G10140, AT3G24320, AT2G46020	93	35	18171	22.32995	1.11E-01	3.85E-02	8.80E-01
heat	GO:0005515~protein binding	36	35.29	2.70E-12	AT4G11260, AT4G29770, AT5G47910, AT3G28030, AT1G64520, AT1G54490, AT1G64280, AT5G07100, AT5G21160, AT2G26150, AT4G04950, AT3G06400, AT1G66340, AT1G03190, AT5G62390, AT3G17880, AT5G44650, AT4G26080, AT2G38470, AT5G02500, AT3G16770, AT5G56010, AT4G26840, AT2G30250, AT5G17020, AT2G46020, AT5G57050, AT5G60410, AT1G50500, AT1G74310, AT2G39770, AT5G53060, AT4G38630, AT1G56260, AT5G05410, AT3G10800	93	1901	18171	3.700124	4.34E-10	4.34E-10	3.26E-09
light	GO:0004871~signal transducer activity	7	4.93	6.52E-04	AT5G64330, AT1G53090, AT2G26300, AT4G16250, AT2G46340, AT2G18790, AT1G09570	128	150	18171	6.624844	9.96E-02	9.50E-03	7.85E-01
light	GO:0071949~FAD binding	4	2.82	6.85E-04	AT4G08920, AT1G77760, AT1G37130, AT1G04400	128	25	18171	22.71375	1.04E-01	9.16E-03	8.25E-01
light	GO:0016987~sigma factor activity	3	2.11	7.14E-04	AT5G24120, AT1G08540, AT2G36990	128	6	18171	70.98047	1.09E-01	8.80E-03	8.59E-01
light	GO:0001053~plastid sigma factor activity	3	2.11	7.14E-04	AT5G24120, AT1G08540, AT2G36990	128	6	18171	70.98047	1.09E-01	8.80E-03	8.59E-01
light	GO:0008020~G-protein coupled photoreceptor activity	3	2.11	2.88E-04	AT4G16250, AT2G18790, AT1G09570	128	4	18171	106.4707	4.53E-02	4.63E-03	3.48E-01
light	GO:0009883~red or far-red light photoreceptor activity	3	2.11	1.45E-04	AT4G16250, AT2G18790, AT1G09570	128	3	18171	141.9609	2.30E-02	2.59E-03	1.75E-01
light	GO:0046983~protein dimerization activity	11	7.75	9.61E-05	AT1G26945, AT1G26260, AT2G20180, AT4G36930, AT1G02340, AT5G61270, AT5G48560, AT1G10120, AT2G43010, AT1G09530, AT4G34530	128	325	18171	4.804832	1.54E-02	1.93E-03	1.16E-01
light	GO:0003677~DNA binding	31	21.83	5.93E-05	AT1G26260, AT2G20180, AT5G43630, AT2G36890, AT1G10120, AT2G43010, AT2G36990, AT4G01120, AT2G21650, AT5G24120, AT3G61850, AT4G36930, AT5G48560, AT3G54610, AT5G07690, AT2G46590, AT1G02340, AT1G08540, AT1G09530, AT1G25540, AT4G34530, AT2G36530, AT4G25560, AT5G39760, AT1G10522, AT5G28490, AT5G20730, AT5G61270, AT4G28610, AT4G17490, AT1G54160	128	2047	18171	2.149873	9.51E-03	1.36E-03	7.17E-02
light	GO:0000155~phosphorelay sensor kinase activity	5	3.52	8.13E-06	AT3G45780, AT4G16250, AT5G58140, AT2G18790, AT1G09570	128	19	18171	37.35814	1.31E-03	2.18E-04	9.83E-03
light	GO:0009881~photoreceptor activity	5	3.52	1.08E-06	AT1G68050, AT5G57360, AT4G16250, AT2G18790, AT1G09570	128	12	18171	59.15039	1.74E-04	3.47E-05	1.30E-03
light	GO:0003700~transcription factor activity, sequence-specific DNA binding	34	23.94	4.87E-08	AT5G59820, AT1G26260, AT1G21970, AT2G20180, AT3G21150, AT2G36890, AT1G10120, AT2G43010, AT2G21320, AT2G36990, AT4G01120, AT3G02790, AT2G21650, AT5G24120, AT3G61850, AT4G36930, AT2G24790, AT5G48560, AT4G40060, AT1G10170, AT5G07690, AT2G46590, AT1G02340, AT1G08540, AT1G09530, AT4G34530, AT4G25560, AT5G39760, AT5G61270, AT5G20730, AT4G28610, AT4G17490, AT4G15090, AT1G54160	128	1711	18171	2.820965	7.85E-06	2.62E-06	5.89E-05
light	GO:0042803~protein homodimerization activity	12	8.45	5.15E-08	AT5G39760, AT2G37678, AT3G61600, AT4G08920, AT4G16250, AT2G46260, AT5G02200, AT3G47340, AT2G18790, AT1G09570, AT1G04400, AT2G40080	128	179	18171	9.516934	8.29E-06	2.07E-06	6.23E-05
light	GO:0009882~blue light photoreceptor activity	5	3.52	1.13E-08	AT5G57360, AT4G08920, AT3G45780, AT5G58140, AT1G04400	128	5	18171	141.9609	1.82E-06	9.11E-07	1.37E-05
light	GO:0005515~protein binding	55	38.73	6.49E-21	AT1G26260, AT1G21970, AT3G21150, AT4G16250, AT4G28860, AT1G10120, AT5G27620, AT4G14110, AT2G36990, AT5G55280, AT1G09570, AT1G22770, AT1G68050, AT1G20090, AT4G36930, AT5G48560, AT3G59220, AT2G46260, AT2G18790, AT1G04400, AT1G53090, AT5G57360, AT1G09530, AT1G25540, AT4G34530, AT4G25560, AT5G64330, AT1G10522, AT3G28860, AT4G28880, AT4G08920, AT2G20180, AT2G43010, AT1G48270, AT4G01120, AT3G05420, AT2G26300, AT3G45780, AT2G24790, AT1G77760, AT3G54610, AT1G26830, AT2G46340, AT1G66840, AT2G39840, AT3G61600, AT1G02340, AT1G70800, AT5G02200, AT1G10470, AT5G39760, AT2G05620, AT4G17490, AT5G43470, AT4G04770	128	1901	18171	4.107234	1.04E-18	1.04E-18	7.84E-18

osmotic	GO:0019900~kinase binding	3	2.63	1.26E-03	AT5G35750, AT4G17615, AT4G08500	99	10	18171	55.06364	1.54E-01	2.37E-02	1.46E+00
osmotic	GO:0005524~ATP binding	26	22.81	6.37E-04	AT4G13850, AT1G60940, AT5G66880, AT1G72180, AT3G46930, AT3G45640, AT2G23030, AT5G45800, AT5G63650, AT3G50500, AT3G17510, AT1G78290, AT3G08730, AT5G35550, AT5G08590, AT4G33950, AT2G18250, AT1G10940, AT2G41560, AT4G01370, AT5G57630, AT2G43850, AT4G40010, AT2G43790, AT4G08500, AT1G35720	99	2372	18171	2.011881	8.13E-02	1.40E-02	7.43E-01
osmotic	GO:0005034~osmosensor activity	3	2.63	1.72E-04	AT2G17820, AT1G27320, AT5G35750	99	4	18171	137.6591	2.26E-02	4.55E-03	2.00E-01
osmotic	GO:0004672~protein kinase activity	13	11.40	1.09E-05	AT3G08730, AT5G08590, AT4G33950, AT1G60940, AT1G10940, AT5G66880, AT4G01370, AT1G72180, AT2G43850, AT3G45640, AT5G45800, AT2G43790, AT3G50500	99	482	18171	4.950396	1.45E-03	3.62E-04	1.27E-02
osmotic	GO:0004674~protein serine/threonine kinase activity	17	14.91	5.89E-06	AT3G08730, AT5G08590, AT4G33950, AT1G60940, AT1G10940, AT5G66880, AT1G72180, AT2G43850, AT4G40010, AT5G57630, AT5G45800, AT2G23030, AT5G63650, AT3G50500, AT3G17510, AT4G08500, AT1G78290	99	804	18171	3.880936	7.83E-04	2.61E-04	6.89E-03
osmotic	GO:0016301~kinase activity	20	17.54	2.42E-06	AT3G08730, AT5G08590, AT4G33950, AT1G60940, AT1G10940, AT5G66880, AT4G01370, AT1G72180, AT3G46930, AT2G43850, AT4G40010, AT5G57630, AT3G45640, AT2G23030, AT5G63650, AT2G43790, AT3G50500, AT3G17510, AT4G08500, AT1G78290	99	1039	18171	3.533118	3.22E-04	1.61E-04	2.83E-03
osmotic	GO:0005515~protein binding	34	29.82	4.90E-10	AT1G01510, AT3G33520, AT1G43700, AT1G27320, AT4G18780, AT5G49450, AT5G66880, AT1G32230, AT1G72180, AT3G45640, AT5G01410, AT5G19660, AT5G62090, AT3G50500, AT4G25520, AT3G17510, AT3G08730, AT2G17820, AT5G13170, AT3G56400, AT4G33950, AT2G40750, AT5G57050, AT2G32700, AT4G01370, AT1G50500, AT2G43850, AT5G57630, AT2G40220, AT5G53060, AT2G43790, AT5G35750, AT4G08500, AT1G15100	99	1901	18171	3.28277	6.52E-08	6.52E-08	5.73E-07
oxidative	GO:0005515~protein binding	23	15.23	1.64E-03	AT2G41090, AT5G20230, AT5G39610, AT5G20140, AT4G03520, AT1G78410, AT1G30460, AT1G08830, AT1G32230, AT1G76080, AT4G35090, AT3G11220, AT2G01980, AT5G01410, AT4G35000, AT3G45640, AT5G09830, AT4G11850, AT2G47510, AT4G10090, AT1G32640, AT3G06110, AT3G14990	109	1901	18171	2.016964	2.40E-01	4.46E-02	1.98E+00
oxidative	GO:0008113~peptide-methionine (S)-S-oxide reductase activity	3	1.99	1.23E-03	AT5G07460, AT4G04800, AT4G25130	109	9	18171	55.56881	1.85E-01	4.02E-02	1.48E+00
oxidative	GO:0047134~protein-disulfide reductase activity	4	2.65	6.67E-04	AT1G76080, AT4G03520, AT1G03680, AT3G15360	109	29	18171	22.99399	1.05E-01	2.75E-02	8.08E-01
oxidative	GO:0050897~cobalt ion binding	6	3.97	8.50E-06	AT5G08670, AT4G35090, AT4G11010, AT3G12490, AT1G02930, AT3G22200	109	47	18171	21.28167	1.42E-03	7.09E-04	1.03E-02
oxidative	GO:0004784~superoxide dismutase activity	4	2.65	1.12E-05	AT4G25100, AT5G18100, AT2G28190, AT1G08830	109	8	18171	83.35321	1.87E-03	6.23E-04	1.36E-02
oxidative	GO:0005507~copper ion binding	13	8.61	2.04E-08	AT5G08670, AT2G14170, AT5G20230, AT1G13440, AT4G25100, AT5G18100, AT1G02930, AT2G05710, AT3G04120, AT2G28190, AT1G08830, AT1G35720, AT4G26970	109	241	18171	8.992463	3.41E-06	3.41E-06	2.48E-05
salt	GO:0000981~RNA polymerase II transcription factor activity, sequence-specific DNA binding	8	3.46	2.09E-03	AT4G37260, AT5G67300, AT3G23250, AT1G16060, AT2G47460, AT1G17950, AT1G14350, AT1G66230	204	159	18171	4.481687	3.68E-01	4.96E-02	2.63E+00
salt	GO:0003700~transcription factor activity, sequence-specific DNA binding	34	14.72	1.34E-03	AT1G16060, AT5G49450, AT1G14920, AT1G17950, AT5G24120, AT1G69310, AT3G61890, AT1G43160, AT5G67300, AT1G27730, AT5G04760, AT2G38470, AT2G47460, AT1G10170, AT1G14350, AT5G46350, AT2G01570, AT5G05660, AT1G06040, AT2G30250, AT1G12610, AT5G13330, AT2G47900, AT3G06590, AT1G66230, AT2G27300, AT4G37260, AT1G66350, AT2G01430, AT3G23250, AT3G51960, AT2G38340, AT5G08520, AT3G03450	204	1711	18171	1.770018	2.54E-01	4.77E-02	1.69E+00
salt	GO:0003690~double-stranded DNA binding	6	2.60	1.34E-03	AT5G55580, AT3G23830, AT3G18870, AT4G13850, AT4G14605, AT2G34620	204	73	18171	7.321112	2.55E-01	4.12E-02	1.70E+00
salt	GO:0005524~ATP binding	43	18.61	1.52E-03	AT3G44110, AT1G51460, AT4G13850, AT2G04030, AT5G59010, AT1G51500, AT1G60940, AT5G26742, AT5G66880, AT3G47950, AT2G46500, AT1G17840, AT3G46930, AT3G22310, AT4G29810, AT5G24240, AT5G63650, AT1G73500, AT3G50500, AT3G50310, AT3G17510, AT5G08620, AT1G04120, AT5G08590, AT1G73660, AT3G06010, AT5G35410, AT4G33950, AT5G56030, AT2G18250, AT5G26751, AT1G10940, AT2G41560, AT1G64460, AT4G01370, AT4G23650, AT4G40010, AT5G57630, AT1G60490, AT1G30580, AT2G43790, AT4G24190, AT1G67580	204	2372	18171	1.614739	2.83E-01	4.07E-02	1.91E+00

salt	GO:0015386~potassium:proton antiporter activity	4	1.73	6.91E-04	AT5G27150, AT2G01980, AT3G06370, AT3G19490	204	16	18171	22.26838	1.40E-01	2.98E-02	8.75E-01
salt	GO:0000989~transcription factor activity, transcription factor binding	4	1.73	3.62E-04	AT1G66350, AT2G01570, AT3G03450, AT1G14920	204	13	18171	27.40724	7.62E-02	1.96E-02	4.59E-01
salt	GO:0044212~transcription regulatory region DNA binding	14	6.06	6.43E-05	AT2G01570, AT1G12610, AT5G49450, AT1G17950, AT1G66230, AT4G37260, AT1G69310, AT2G38340, AT3G23250, AT5G67300, AT1G27730, AT2G38470, AT2G47460, AT1G14350	204	319	18171	3.909183	1.40E-02	4.68E-03	8.17E-02
salt	GO:0043565~sequence-specific DNA binding	27	11.69	3.95E-08	AT4G06634, AT5G49450, AT1G14920, AT1G17950, AT3G61050, AT5G03740, AT1G69310, AT3G61890, AT5G67300, AT1G27730, AT2G38470, AT2G47460, AT1G14350, AT5G46350, AT2G01570, AT2G30250, AT1G12610, AT5G13330, AT1G66230, AT2G27300, AT4G37260, AT1G66350, AT2G01430, AT2G38340, AT3G51960, AT3G23250, AT3G03450	204	676	18171	3.557671	8.65E-06	4.33E-06	5.02E-05
salt	GO:0005515~protein binding	53	22.94	6.24E-10	AT1G01510, AT3G12360, AT2G04030, AT1G03060, AT4G26630, AT5G62090, AT3G50500, AT4G25520, AT1G78310, AT3G17980, AT5G46350, AT1G73660, AT4G24500, AT1G06040, AT5G56030, AT3G51920, AT4G21670, AT2G32700, AT5G27150, AT5G57630, AT2G01980, AT1G30580, AT2G43790, AT4G38630, AT3G03450, AT2G16005, AT5G35080, AT5G08450, AT5G63510, AT5G49450, AT1G14920, AT5G66880, AT1G32230, AT3G48680, AT1G17840, AT2G26650, AT1G59860, AT5G01410, AT4G29810, AT2G45640, AT2G38470, AT3G17510, AT4G24560, AT2G01570, AT5G63110, AT2G30250, AT5G35410, AT4G33950, AT4G01370, AT2G39770, AT2G41010, AT1G67580, AT1G15100	204	1901	18171	2.483376	1.37E-07	1.37E-07	7.94E-07
water	GO:0071949~FAD binding	4	1.90	2.12E-03	AT4G08920, AT3G30775, AT5G67030, AT1G04400	189	25	18171	15.38286	3.42E-01	3.73E-02	2.62E+00
water	GO:0015204~urea transmembrane transporter activity	3	1.42	1.55E-03	AT3G16240, AT2G36830, AT4G01470	189	6	18171	48.07143	2.64E-01	3.02E-02	1.93E+00
water	GO:0003677~DNA binding	40	18.96	1.13E-04	AT4G31920, AT1G22810, AT1G15360, AT3G15500, AT5G11270, AT4G24020, AT1G16060, AT1G17950, AT1G52890, AT1G45249, AT1G18390, AT5G62470, AT3G05700, AT4G25480, AT1G33240, AT3G16857, AT2G25180, AT3G22310, AT3G10500, AT4G27410, AT3G18490, AT5G07690, AT1G14350, AT5G09410, AT3G11020, AT3G20310, AT1G78080, AT1G12610, AT3G06010, AT3G12630, AT5G60410, AT1G36060, AT2G40220, AT3G23250, AT3G47600, AT1G69600, AT1G80710, AT1G54160, AT5G05410, AT1G46768	189	2047	18171	1.878708	2.21E-02	2.48E-03	1.42E-01
water	GO:0004842~ubiquitin-protein transferase activity	18	8.53	9.38E-06	AT5G18320, AT2G30580, AT5G50430, AT4G34100, AT3G52450, AT2G35930, AT3G12630, AT3G17000, AT2G42620, AT4G23450, AT5G59550, AT1G06770, AT3G01650, AT3G46620, AT5G14420, AT3G13672, AT3G56580, AT1G17280	189	475	18171	3.643308	1.85E-03	2.31E-04	1.17E-02
water	GO:0044212~transcription regulatory region DNA binding	15	7.11	6.16E-06	AT3G11020, AT3G20310, AT1G12610, AT1G17950, AT5G62470, AT1G69310, AT2G46400, AT2G40220, AT3G23250, AT3G53600, AT3G47600, AT1G27730, AT5G07690, AT5G05410, AT1G14350	189	319	18171	4.520824	1.21E-03	1.73E-04	7.70E-03
water	GO:0003700~transcription factor activity, sequence-specific DNA binding	39	18.48	4.77E-06	AT4G31920, AT1G22810, AT1G15360, AT3G15500, AT5G11270, AT4G24020, AT1G16060, AT1G17950, AT1G52890, AT1G45249, AT5G62470, AT1G69310, AT2G46400, AT4G25480, AT1G33240, AT3G16857, AT2G25180, AT3G10500, AT1G27730, AT3G23050, AT4G27410, AT5G07690, AT1G14350, AT3G11020, AT3G20310, AT1G78080, AT3G56400, AT1G12610, AT2G38880, AT2G40750, AT1G36060, AT2G40220, AT3G23250, AT3G53600, AT3G47600, AT1G69600, AT1G54160, AT5G05410, AT1G46768	189	1711	18171	2.19145	9.39E-04	1.57E-04	5.96E-03
water	GO:0016874~ligase activity	14	6.64	3.26E-06	AT5G18320, AT2G30580, AT4G34100, AT3G52450, AT2G35930, AT4G23450, AT1G06770, AT5G59550, AT5G60410, AT3G01650, AT3G46620, AT5G14420, AT3G13672, AT3G56580	189	260	18171	5.176923	6.42E-04	1.28E-04	4.08E-03
water	GO:0043565~sequence-specific DNA binding	28	13.27	1.75E-09	AT1G15360, AT1G28520, AT1G17950, AT1G45249, AT3G61050, AT5G03740, AT5G62470, AT1G69310, AT2G46400, AT1G33240, AT3G10500, AT1G27730, AT4G27410, AT5G07690, AT1G14350, AT5G09410, AT3G11020, AT3G20310, AT1G78080, AT3G56400, AT1G12610, AT2G38880, AT2G40750, AT2G40220, AT3G23250, AT3G53600, AT3G47600, AT5G05410	189	676	18171	3.982249	3.46E-07	8.64E-08	2.19E-06
water	GO:0005515~protein binding	51	24.17	3.24E-10	AT3G14080, AT4G35100, AT5G27620, AT2G04030, AT5G64960, AT4G18780, AT1G52890, AT1G45249, AT5G47100,	189	1901	18171	2.579319	6.38E-08	2.13E-08	4.05E-07

					AT3G23050, AT3G50500, AT1G04400, AT2G18960, AT2G30580, AT1G78080, AT3G56400, AT5G56030, AT2G40750, AT3G51920, AT5G57050, AT3G28270, AT2G40220, AT1G13740, AT1G08720, AT1G19120, AT5G05410, AT4G08920, AT2G31470, AT3G26090, AT3G15500, AT4G26070, AT5G66880, AT1G32230, AT1G76080, AT3G45140, AT2G26650, AT5G44650, AT2G37040, AT1G01620, AT5G40280, AT3G59380, AT2G17820, AT3G54820, AT3G20310, AT1G30270, AT2G38880, AT4G33950, AT5G08120, AT5G60410, AT2G05620, AT1G69600							
water	GO:0015250~water channel activity	14	6.64	6.46E-17	AT2G37170, AT2G37180, AT3G54820, AT3G16240, AT4G35100, AT2G45960, AT5G18290, AT4G00430, AT3G53420, AT3G61430, AT2G36830, AT3G04090, AT1G01620, AT4G01470	189	39	18171	34.51282	2.19E-14	1.09E-14	1.44E-13
water	GO:0015254~glycerol channel activity	14	6.64	1.89E-17	AT2G37170, AT2G37180, AT3G54820, AT3G16240, AT4G35100, AT2G45960, AT5G18290, AT4G00430, AT3G53420, AT3G61430, AT2G36830, AT3G04090, AT1G01620, AT4G01470	189	36	18171	37.38889	3.72E-15	3.72E-15	2.36E-14
